# Borate-catalysed direct amidation reactions of coordinating substrates†

**DOI:** 10.1039/d4sc07744j

**Published:** 2025-02-04

**Authors:** Richard J. Procter, Carla Alamillo-Ferrer, Usman Shabbir, Phyllida Britton, Dejan-Krešimir Bučar, Alexandre S. Dumon, Henry S. Rzepa, Jordi Burés, Andrew Whiting, Tom D. Sheppard

**Affiliations:** a Department of Chemistry, Christopher Ingold Laboratories, University College London 20 Gordon St London WC1H 0AJ UK tom.sheppard@ucl.ac.uk; b Department of Chemistry, The University of Manchester Manchester M13 9PL UK jordi.bures@manchester.ac.uk; c Department of Chemistry, Molecular Sciences Research Hub, Imperial College London White City Campus, Wood Lane London W12 OBZ UK rzepa@imperial.ac.uk; d Centre for Sustainable Chemical Processes, Department of Chemistry, Science Laboratories, Durham University South Road Durham DH1 3LE UK andy.whiting@durham.ac.uk

## Abstract

The catalytic activity of different classes of boron catalysts was studied in amidation reactions with 4-phenylbutylamine/benzoic acid, and with 2-aminopyridine/phenylacetic acid. Whilst a simple boronic acid catalyst showed high catalytic activity with the former substrates, it was completely inactive in the latter reaction. In contrast, a borate ester catalyst was able to mediate the amidation of both substrate pairs with moderate activity. By screening a range of borate esters we were able to identify a novel borate catalyst that shows high reactivity with a range of challenging carboxylic acids/amine pairs, enabling catalystic amidation reactions to be achieved effectively with these industrially relevant compounds. The reactions can be performed on multigram scale with high levels of efficiency, and *in situ* catalyst generation from commercially available reagents renders the process readily accessible for everyday laboratory use. Further experiments showed that the deactivating effect of 2-aminopyridine on boronic acid catalysts was due to its ability to stabilise catalytically inactive boroxines.

## Abstract 2

A new borate ester amidation catalyst was developed, that shows higher reactivity with challenging carboxylic acids and amines. Reactions could be performed on multigram scale with the catalyst generated in situ from commercial reagents.

## Graphical abstract

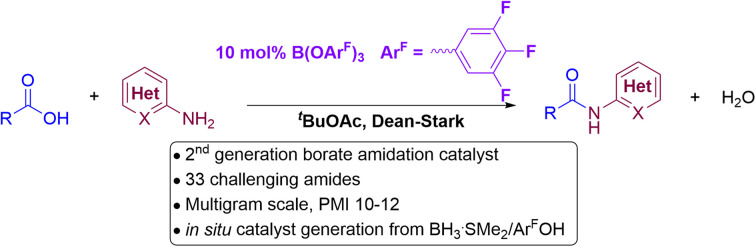

## Introduction

The synthesis of amides is perhaps the most common process in organic chemistry, with amides forming key linkages in a vast array of useful molecules, including pharmaceuticals, agrochemicals, and organic polymers.^1–3^ Amides are also common synthetic intermediates used in the preparation of other important functional groups such as amines and heterocycles. Amide formation is typically a highly inefficient process which leads to the generation of significant quantities of waste, and there is considerable interest in rendering the process more efficient.^4^ The most common readily available precursors to the amide unit, carboxylic acids and amines, must undergo a formal dehydration during the amide coupling reaction, and this is typically achieved either *via* pre-activation of the carboxylic acid or *via* the use of a stoichiometric dehydrating agent which provides activation *in situ*. In both cases the byproducts obtained are typically of high molecular weight, while the reagents used are often hazardous or toxic. Catalytic methods for amide formation from carboxylic acids and amines are gaining prominence,^5^ and in such reactions the only stoichiometric byproduct is water. However, these reactions are still not widely employed in synthetic chemistry laboratories for a variety of reasons, notably a relatively narrow substrate scope and a lack of accessibility (*e.g.*, slow reaction rates; catalysts that are not commercially available or are too expensive). In many cases the reaction scope is quite limited to the preparation of largely unfunctionalized amides. The application of most catalytic reactions to polar substrates and/or those containing coordinating functional groups is often low yielding or unreported.^5^ However, these classes of amides are typically the ones most widely in demand for the many applications outlined above. Similarly, catalytic amidation reactions often fail with poorly nucleophilic amines (electron-deficient anilines, heterocyclic amines), and lower yields are frequently obtained from less-reactive carboxylic acids (benzoic acids, heterocyclic carboxylic acids, fluorinated carboxylic acids). This latter factor often correlates with acidity, with carboxylic acids of low p*K*_a_ typically showing lower reactivity in catalytic amidation processes.^6^

Reported catalysts include group (IV) metals,^7–10^ polyoxometallates,^11–15^ silanes/silanols,^16^ and boron compounds,^17–38^ with the latter class of catalysts being the most widely studied. Boronic acids have been widely explored as amidation catalysts since the key report using 3,4,5-trifluorophenylboronic acid by Yamamoto in 1996,^17^ with catalysts since reported by a number of research groups around the world. Key developments have included bifunctional boronic acid catalysts,^18^ and 5-methoxy-2-iodophenylboronic acid,^20^ the latter an active catalyst at room temperature using molecular sieves as a drying agent. However, for coordinating amidation partners (*e.g.* aromatic heterocyclic acids + heterocyclic anilines) these catalysts remain ineffective. More recent developments have included diboronic acids^23–25^ and a boron-containing heterocyclic catalyst^26–29^ that is effective with a range of hindered/challenging substrates. Boric acid itself is well-known as a very low-cost catalyst for amidation reactions of relatively reactive substrates,^33^ while borate esters have been reported as highly effective catalysts for amidation reactions of a wide-range of substrates,^34,35^ including many acids or amines that were previously considered unsuitable for catalytic amidation reactions (*e.g.* unprotected amino acids^36^). We envisaged that by studying the effectiveness of different boron-based amidation catalysts with coordinating substrates we could obtain an understanding of how catalyst deactivation takes place, and which catalysts were more suitable for mediating these challenging catalytic amidation reactions. This in turn should enable the identification of new catalysts that are widely applicable to amidation reactions of coordinating substrates.

## Results and discussion

**Scheme 1:**
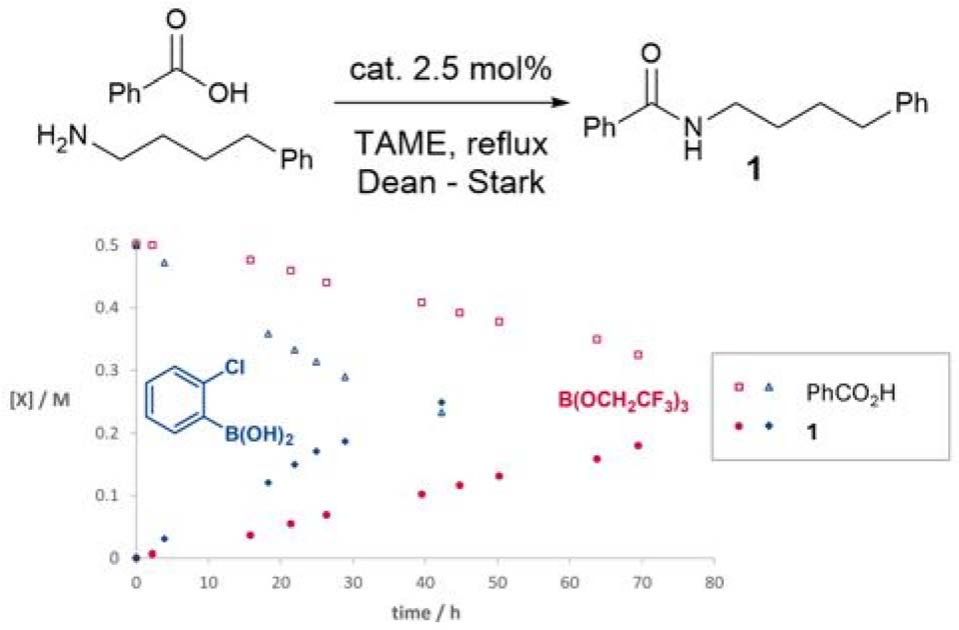
Time course experiments of boron catalysts in the reaction of 4-phenylbutylamine and benzoic acid.

In preliminary experiments (Scheme 1), we studied a simple amidation reaction of ‘easy’ (*i.e.* non-functionalised) substrates, 4-phenylbutylamine and benzoic acid in tertiary amyl methyl ether (TAME) under Dean–Stark conditions. A simple boronic acid catalyst (2-chlorophenylboronic acid) led to a considerably faster amidation rate than a reactive borate ester, B(OCH_2_CF_3_)_3_. As we have previously reported, 2-chlorophenylborinic acid [Ar_2_B(OH)] was catalytically inactive under the same conditions.^39^

**Scheme 2:**
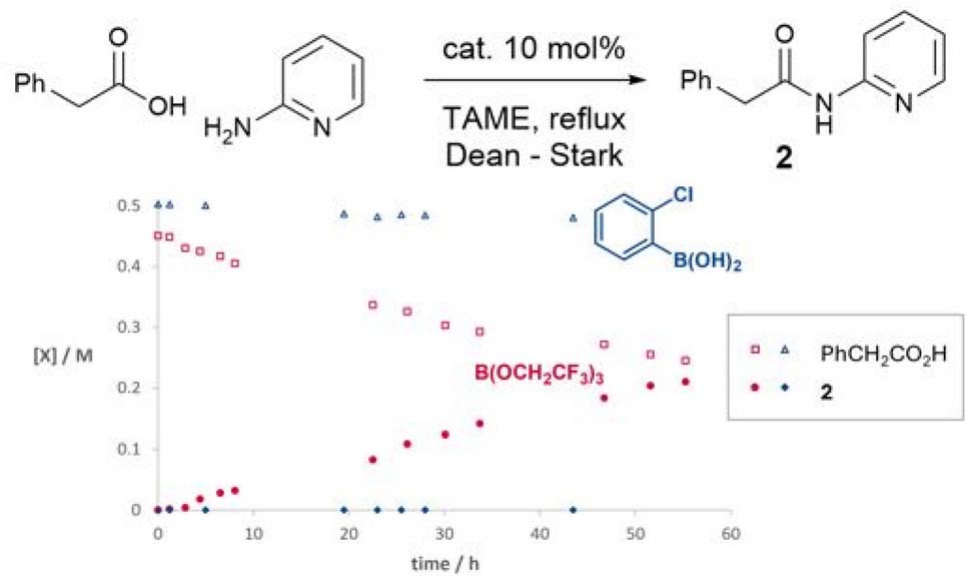
Time course experiments of boron catalysts in the reaction of 2-aminopyridine and phenylacetic acid.

We have previously observed that 2-aminopyridine is a particularly challenging amine for amidation reactions,^40^ perhaps because it is poorly nucleophilic and also contains the adjacent coordinating pyridine nitrogen which is potentially able to chelate to Lewis acids and hence inhibit their ability to mediate amidation. We therefore examined the reactivity of the same boron catalysts on the more challenging substrate combination of phenylacetic acid and 2-aminopyridine to give amide 2 (Scheme 2). A starkly different outcome was observed from the formation of simple amide 1, with the boronic acid showing no catalytic activity at all, whereas the borate ester catalyst continued to mediate the reaction effectively. However, the reaction rate was relatively slow with only a 63% isolated yield of amide after 40 hours.

**Fig. 1:**
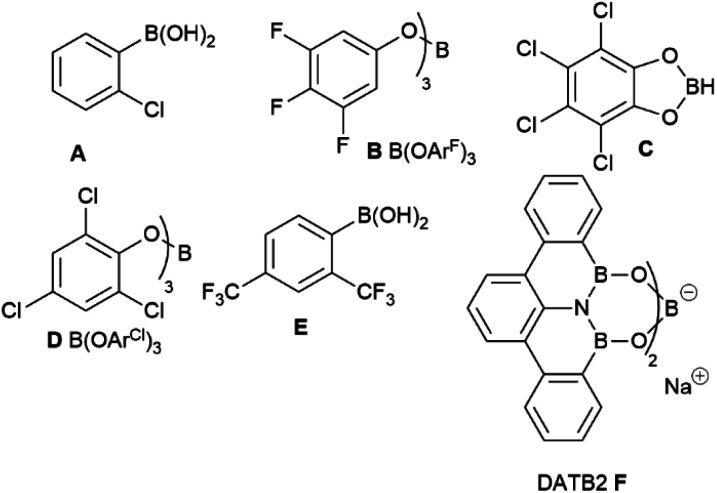
Additional structures of catalysts examined in Tables 1 and 2.

Although B(OCH_2_CF_3_)_3_ has previously worked well for catalytic amidation of a range of challenging substrates, this is often limited to reactions of a challenging amine with a simple carboxylic acid (or *vice versa*), while reactions of substrate pairs where both partners are deactivated are typically low-yielding.^34^ We therefore set out to screen a range of borates to identify higher activity catalysts that may be applicable to these more challenging combinations (Fig. 1). Borates were purchased from commercial suppliers or synthesized from the respective alcohols and BH_3_·SMe_2_ or BCl_3_,^41–46^ as described in the ESI.† Initial screening (Table 1) was performed using the reaction of benzoic acid with benzylamine under Dean–Stark dehydration conditions in *tert*-amyl methyl ether (TAME) as previously employed in our initial work with B(OCH_2_CF_3_)_3_.^34^

**Table 1:** Screening of borate ester catalysts in the reaction of benzoic acid and benzylamine

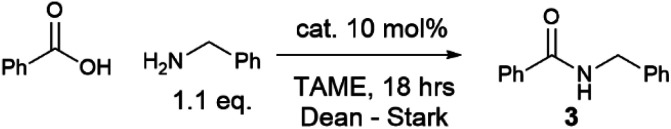				
	Catalyst	Yield	Alcohol p*K*_a_	Lewis acidity^a^
1	B(OH)_3_	20	14	
2	B(OMe)_3_	36	15.3	23.1
3	B(OCH_2_CF_3_)_3_	58	12.4	66.4
4	B(OCH(CF_3_)_2_)_3_	60	9.3	79.4
5	B(OC(CF_3_)_3_)_3_	51	5.4	92.7
6	B(OCH_2_(CF_2_)_3_CF_2_H)_3_	65		
7	B(OCH_2_Cl_3_)_3_	64	12.2	
8	B(OPh)_3_	52	10	62.9
9	B(OAr^F^)_3_B	**71**	8.2	88.8
10	B(OC_6_Cl_5_)_3_	51	4.7	
11	Cl_4_CatBH C	57		
12	ArB(OH)_2_A	**78**		

a Determined using the Gutmann–Beckett acceptor method.^47,48^

This reaction was chosen as, although it will go to completion with B(OCH_2_CF_3_)_3_, it is relatively slow, enabling differences in catalyst reactivity to be readily identified. After 18 hours, B(OCH_2_CF_3_)_3_ gave a 58% yield of amide, with boric acid and trimethyl borate giving significantly lower yields as expected (entries 1–3). An obvious strategy to try and increase the reactivity of the borate ester was to replace 2,2,2-trifluoroethanol with more acidic analogues such as 1,1,1,3,3,3-hexafluoroisopropanol (entry 4) or perfluoro-*tert*-butanol (entry 5), which should lead to a more Lewis-acidic borate with potentially higher reactivity. Our initial screen revealed, however, that these borates offered little improvement in reactivity (60% and 51% yield respectively, entries 4–5) with the catalytic reactivity showing little correlation to the Lewis acidity of the borates (as measured by the Gutmann–Beckett acceptor number^47,48^). It is likely that in many cases the increased Lewis acidity of the borate is balanced by increased steric hindrance or increased volatility of the alcohol (and therefore a reduced catalyst stability¶¶We have previously observed that the catalytic activity of B(OCH_2_CF_3_)_3_ is reduced significantly in higher boiling point solvents were larger quantities of CF_3_CH_2_OH are removed into the Dean–Stark trap.^35^). A borate derived from a larger polyfluorinated alcohol offered a moderate improvement in yield (65%, entry 6), perhaps indicating that alcohol volatility was a significant factor affecting the performance of these catalysts.¶ Similarly, the borate derived from 2,2,2-trichloroethanol gave a slightly improved yield (64%, entry 7). We then switched to phenol-derived borates,^37^ and although the unsubstituted phenyl borate offered little advantage (entry 8), the 3,4,5-trifluorophenol derivative B [B(OAr^F^)_3_] showed a significantly improved reactivity (71%, entry 9). Pentachlorophenyl borate and tetrachlorocatecholborane^21^ were only moderately effective in this reaction (entries 10–11). Notably, 2-chlorophenylboronic acid performed more effectively than B(OAr^F^)_3_, giving a 78% yield of amide in 18 hours (entry 12).

Given the moderate reactivity improvements observed with this amidation reaction of unfunctionalized substrates and our desire to find catalysts for more difficult reactions, we then screened the catalysts against a more challenging amide formation reaction between 3-fluorophenylacetic acid and 2-aminopyridine (Table 2) both of which we have found to be relatively demanding substrates.^40^ Again, the reaction was stopped after 18 h to provide effective differentiation between catalysts; increased yields could be obtained if desired by running the reactions for longer. This reaction was much lower yielding, with all of the simple halogenated alkyl borate catalysts performing poorly (15–24% yield, entries 1–5). Two results were notable – the boronic acid catalyst A in this case was completely inactive (entry 6), in distinct contrast to the benzoic acid/benzylamine reaction above (Table 1). Other catalysts containing boronic acids such as 2,4-bis(trifluoromethyl)phenyl boronic acid E, where deleterious co-ordination of amines is reported to be hindered by the ortho substituent,^38^ and the BNO heterocyclic catalyst F (DATB2)^29^ were also low-yielding (entries 7–8). 3,4,5-Trifluorophenyl borate B [B(OAr^F^)_3_, entry 9] was demonstrably the most reactive catalyst for this amidation, giving much higher conversions than other aryl borates (entries 10–12). A moderate isolated yield of the amide 4 (57%) could be obtained using B as a catalyst, if the reaction time was extended to 66 h.

**Table 2:** Screening of catalysts in the amidation reaction of 2-aminopyridine with 3-fluorophenylacetic acid

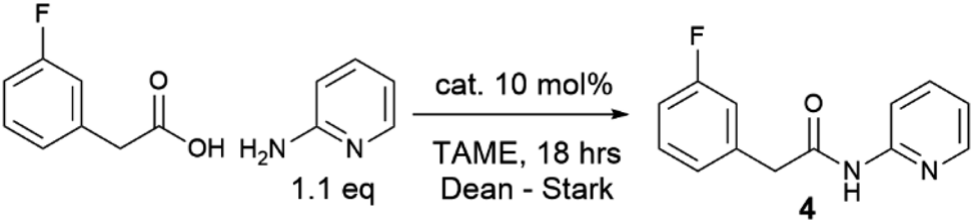		
	Catalyst	Yield
1	B(OCH_2_CF_3_)_3_	20
2	B(OCH_2_Cl_3_)_3_	16
3	B(OCH(CF_3_)_2_)_3_	24
4	B(OC(CF_3_)_3_)_3_	22
5	B(OCH_2_(CF_2_)_3_CF_2_H)_3_	15
6	*o*-ClC_6_H_4_B(OH)_2_A	0
7	*o*,*p*-(CF_3_)_2_C_6_H_3_B(OH)_2_E	15
8	DATB2 F	20
9	B(OAr^F^)_3_B	**43**
10	B(OPh)_3_	28
11	B(OAr^cl^)_3_D	29
12	B(OC_6_Cl_5_)_3_	26

**Scheme 3:**
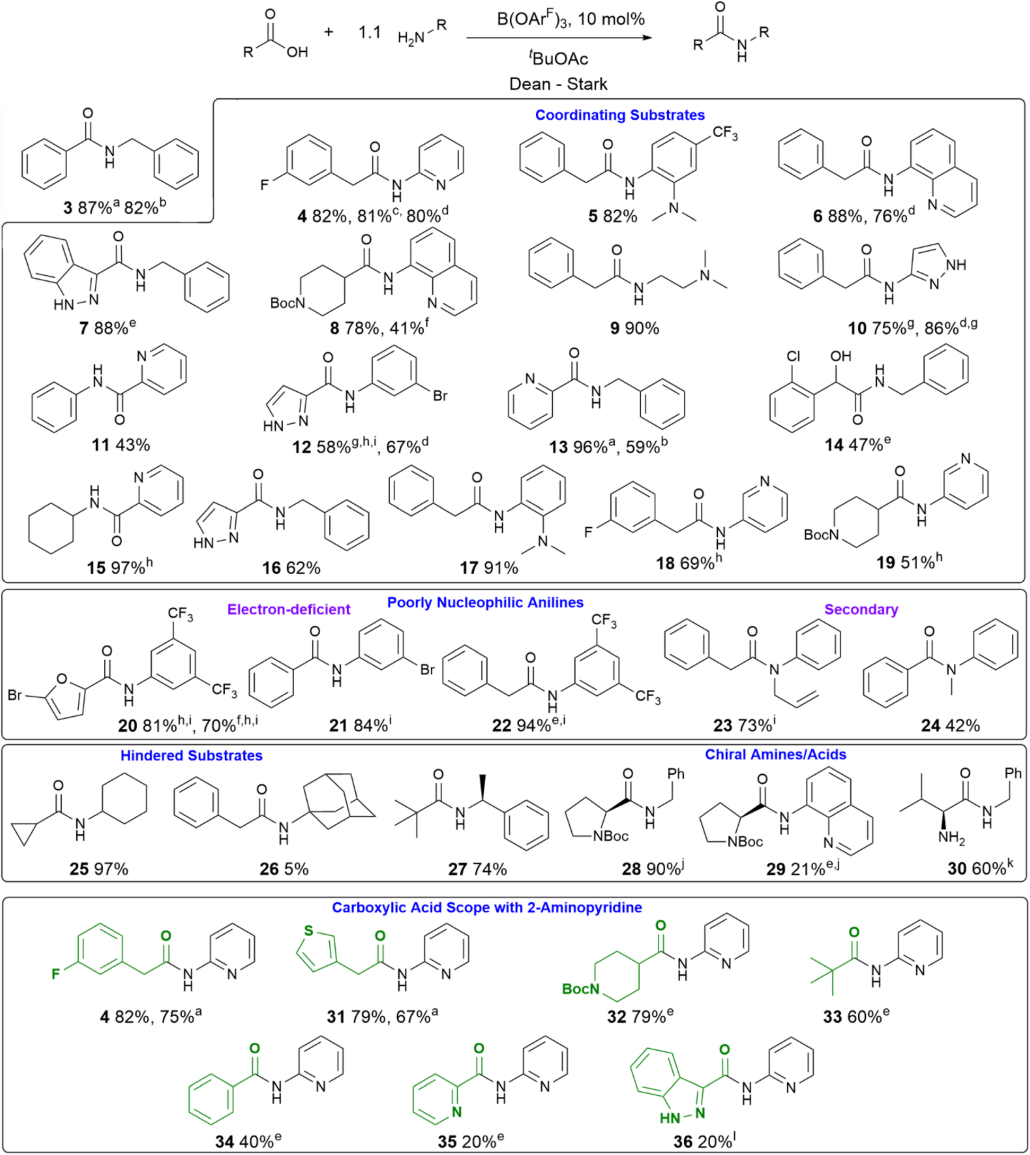
Reaction scope of B(OAr^F^)_3_-catalysed amidation reactions with challenging substrates ^a^ 5% catalyst; ^b^ 2% catalyst; ^c^ catalyst generated *in situ* from 10 mol% BH_3_·SMe_2_ and 30 mol% Ar^F^OH; ^d^ catalyst generated *in situ* from 10 mol% BH_3_·SMe_2_ and 20 mol% Ar^F^OH; ^e^ reaction time 40–45 h; ^f^ using 10% A as catalyst; ^g^ purified by recrystallisation; ^h^ reaction time 24 h; ^i^ toluene used as reaction solvent; ^j^ no epimerisation observed; ^k^ catalyst generated *in situ* from 15 mol% BH_3_·SMe_2_ and 30 mol% Ar^F^OH, 90 : 10 enantiomeric ratio; ^l^ reaction time 72 h.

With a new borate catalyst identified, we sought to explore its scope for the preparation of a range of challenging amides (Scheme 3). By switching the solvent to *tert*-butyl acetate^35^ we were able to improve the isolated yields of amides 3 and 4 to >80% after 20 h. Electron-poor and potentially co-ordinating amines and acids were tolerated (4–17), including 8-aminoquinoline, a common directing group for C–H activation (6, 8). A selection of poorly nucleophilic anilines including electron-deficient examples (20–22) and hindered secondary anilines (23–24) underwent amidation effectively. For certain anilines, toluene was selected as the reaction solvent due to side reactions observed in ^*t*^BuOAc. Reactions with 3,4-bis-trifluoromethyl aniline required the use of toluene, as azeotropic water-removal did not take place effectively in ^*t*^BuOAc. Notably, boronic acid catalyst A was also fairly effective for the amidation of non-coordinating electron-deficient anilines (*e.g.*20). With the borate catalyst B, moderately hindered acids/amines could be used (*e.g.*25, 27), but 1-adamantylamine showed low reactivity (26), perhaps unsurprisingly as it is poorly soluble in the reaction mixture. Proline amides (28–29) could be prepared, although when using a poorly reactive amine the yield was low (29). The catalyst could also be used to directly form an amide from the hindered amine acid l-valine (30). We then sought to further examine the limits of the new catalyst by screening the reaction of 2-aminopyridine with a range of carboxylic acids (4, 31–36). Primary alkyl carboxylic acids were coupled effectively (4, 31), more hindered *N*-Boc piperidine 4-carboxylic acid (32) and pivalic acid (33) reacted in good yield with prolonged reaction times. Aromatic carboxylic acids (34–36) proved less reactive, giving low yields even after prolonged reaction times, with the heterocyclic aromatic carboxylic acids (35–36) proving particularly challenging.

**Scheme 4:**
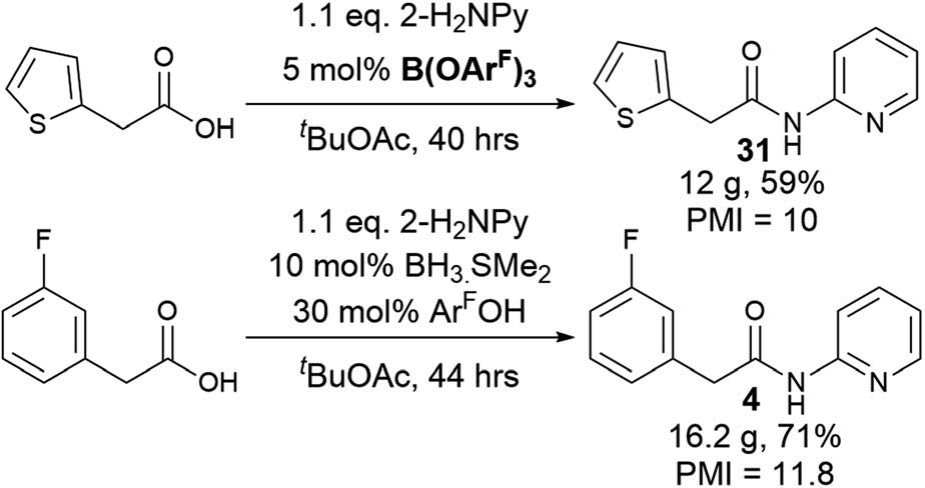
Multigram scale amidation reactions and *in situ* catalyst generation.

To explore a more accessible procedure, we devised conditions for *in situ* formation of the catalyst *via* reaction of BH_3_·SMe_2_ and 3,4,5-trifluorophenol for direct use in an amidation reaction. This approach gave comparable or better yields for amides 4, 6, 10 and 12, and circumvents the need to prepare and isolate the catalyst, enabling rapid evaluation of this novel catalytic system for challenging amidation substrates. Notably the *in situ* catalyst formation could be performed with only 2 eq. of phenol with respect to BH_3_·SMe_2_. We then examined multigram scale reactions to explore the efficiency of the reaction (Scheme 4), with the products isolated by crystallisation from the reaction mixture. Process mass intensity (PMI) is a widely used and convenient metric for measuring the efficiency of a chemical process.^49^ It is calculated by dividing the total mass of input materials used in a reaction (reagents, solvents, catalysts) by the quantity of product obtained. Twelve grams of amide 30 could be obtained in 59% yield using only 5 mol% catalyst, with a PMI of 10 demonstrating the efficiency of the method.^5^ Scaling up the synthesis of 4 with *in situ* catalyst generation gave 16.2 g of amide 4 in 71% yield, with a PMI of 11.8.

Finally, we sought to explore the reasons why 2-aminopyridine was a particularly challenging substrate for catalytic amidation reactions using boron catalysts (Table 3). Direct amidation of benzylamine/benzoic acid proceeds efficiently with 10 mol% 2-chorophenyl boronic acid catalyst, giving a 79% yield of amide (entry 1). Addition of increasing quantities of 2-aminopyridine to this reaction leads to significant lowering of the yield (entries 2–5), demonstrating that 2-aminopyridine is not simply a poor substrate for the reaction, it actively inhibits the process. 4-Aminopyridine or 4-dimethylaminopyridine (4-DMAP) also inhibit the reaction but to a significantly lesser extent (entries 6–7). Interestingly, addition of tributylamine leads to a small enhancement of the amidation yield (entry 8); its addition to other slower reactions provided only very minor improvements in yield, however. Notably, our borate ester catalyst B(OAr^F^)_3_ is less significantly inhibited by the addition of 2-aminopyridine, with a 55% yield of amide being obtained even with 1 eq. of 2-aminopyridine present (10 eq. with respect to catalyst).

**Table 3:** Inhibition of a catalytic amidation reaction by 2-aminopyridine

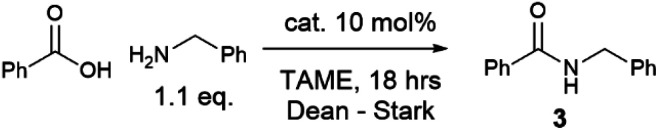				
	Additive	Eq.	Yield (A)/%	Yield B (OAr^F^)_3_/%
1	None	—	78	71
2	2-NH_2_Py	0.1	67	—
3		1	41	55
4		2	21	32
5		4	10	23
6	4-NH_2_Py	1	70	
7	4-DMAP	1	68	
8	NBu_3_	1	89	

**Scheme 5:**
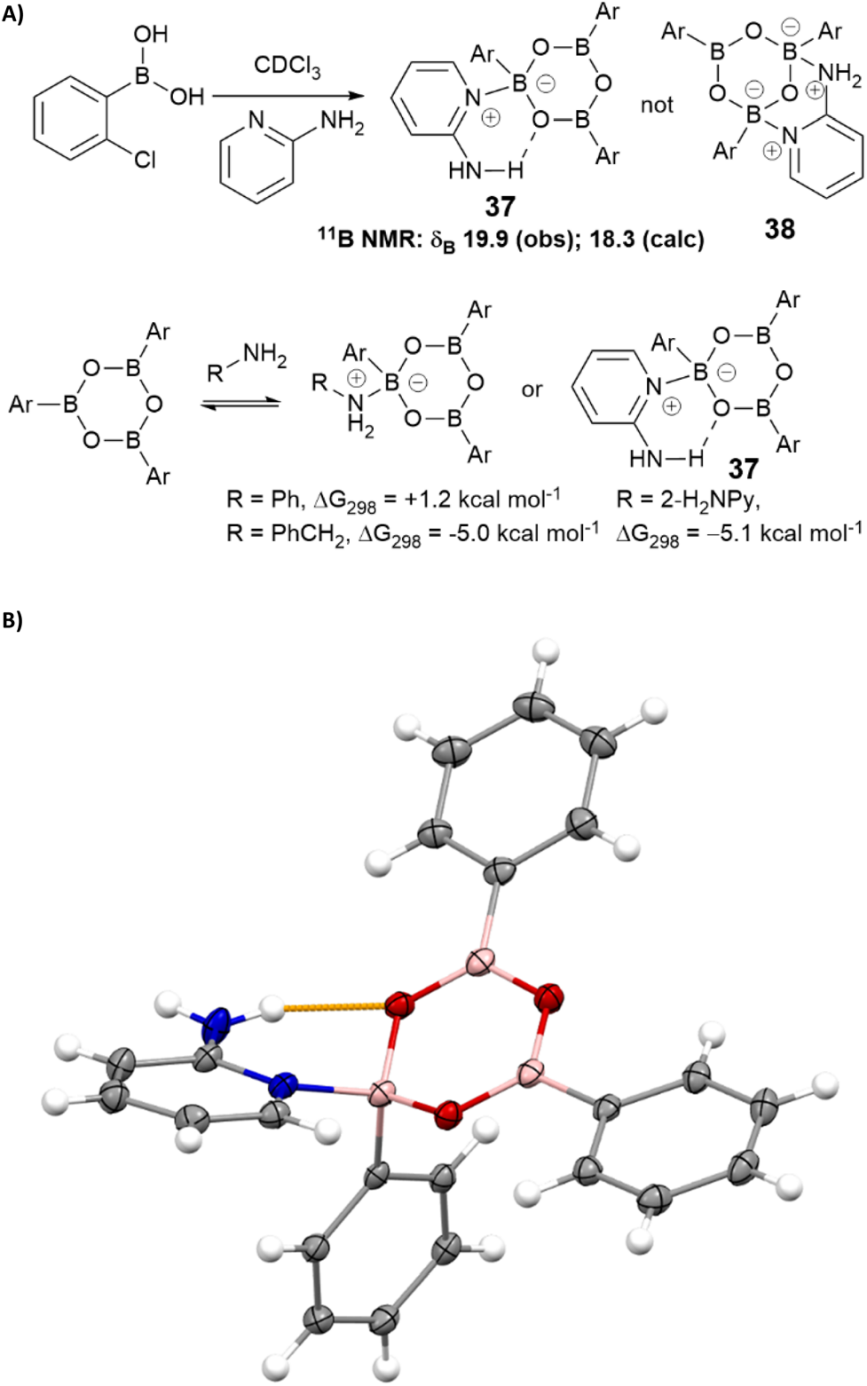
(A) Complexation of boroxines with 2-aminopyridine and other amines; (B) X-ray crystal structure of the complex between phenylboroxine and 2-aminopyridine showing the key hydrogen bonding interaction (yellow).

We hypothesised that 2-aminopyridine may inhibit catalysis by binding/stabilising catalytically inactive species in the solution. Treatment of 2-chlorophenylboronic acid with 2-aminopyridine led to the formation of a stable boroxine complex (Scheme 5) with an NMR spectrum consistent with NMR shift predictions for the structure 37 (rather than chelated isomer 38 which was predicted to be thermodynamically unstable with respect to 37).^50,51^ Calculations at the B3LYP+GD3+BJ/Def2-TZVPP/SCRF level^52^ suggest this complex is 5.1 kcal mol^−1^ lower in free energy (Δ*G*_298_) than the boroxine/amine components using chloroform as a continuum solvent. The corresponding values for complexes with benzylamine and aniline are respectively −5.0 and +1.2 kcal mol^−1^. The complex between phenyl boroxine and 2-aminopyridine was successfully characterised by single crystal X-ray diffraction, confirming the hypothesised hydrogen bonding interaction between the pyridyl amine and the boroxine oxygen (Scheme 5B).

Taken together, these observations suggest that the reactivity of amines in boronic acid-catalysed amidation reactions is affected not only by the nucleophilicity of the amine, but also its ability to stabilise off-cycle inactive species derived from the catalyst. This latter effect becomes particularly important when the amine has low reactivity in the desired amidation reaction and leads to ‘negative feedback’ as significant quantities of catalyst are sequestered in the off-cycle complex, further suppressing the amidation rate. Although benzylamine is capable of forming a relatively stable off-cycle species, its high nucleophilicity means that amidation still readily occurs. In contrast, whilst aniline has relatively low nucleophilicity it is less able to form a stable off-cycle complex with the catalyst, so direct amidation reactions of aniline are usually straightforward. Although 2-aminopyridine also coordinates to borate ester catalysts to form some tetrahedral boron species,||||See ESI† for further details.^,^^50,51^ it does not seem to significantly inhibit the amidation reaction.

## Conclusions

2-Aminopyridine was found to inhibit boron-catalysed amidation reactions, with a particularly significant effect on catalysts incorporating boronic acid functionality; borate ester catalysts are inhibited to a lesser degree. This catalyst inhibition is probably due to stabilisation of catalytically inactive off-cycle boroxines. We have discovered that 3,4,5-trifluorophenol borate is highly effective for catalytic amidations using previously unreactive substrates, in particular working with very electron poor amines and substrates with competitive coordinating functionality. The catalyst is readily synthesized on multigram scale and can be generated directly in the reaction from BH_3_·SMe_2_/Ar^F^OH without additional purification. After reaction, amides can be purified by crystallization or base washing leading to highly efficient processes for the preparation of amides in multigram quantities. We anticipate that by increasing the generality and accessibility of catalytic amidation reactions they may become more prevalent in day-to-day laboratory organic synthesis.

## Data availability

Data supporting this manuscript is available within the ESI.† Full raw NMR data, X-ray data, and computational data can be found on an open access data repository at DOI: https://doi.org/10.14469/hpc/12218.^52^ An IUPAC FAIRSpec Finding Aid for the NMR spectroscopic data is available at DOI: https://doi.org/10.14469/hpc/14884. A selection of data discovery searches can be found at DOI: https://doi.org/10.14469/hpc/14822.

## Author contributions

Conceptualization: RJP, CAF, HSR, JB, AW, TDS. Data curation: HSR. Formal analysis: all. Funding acquisition: HSR, JB, AW, TDS. Investigation: RJP, CAF, US, PB, DKB, ASD. Methodology: RJP, CAF, US, HSR, JB, AW, TDS. Project Administration: RJP, CAF, HSR, JB, AW, TDS. Supervision: HSR, JB, AW, TDS. Validation: RJP, CAF, US, HSR, JB, AW, TDS. Visualisation: RJP, CAF, PB, DKB, HSR, JB, TDS. Writing – original draft: RJP, TDS. Writing – review & editing: RJP, CAF, HSR, JB, AW, TDS.

## Conflicts of interest

There are no conflicts to declare.

## Supplementary Material

SC-016-D4SC07744J-s001

SC-016-D4SC07744J-s002
