# Supplementary material for: Borate-catalysed direct amidation reactions of coordinating substrates
Source: Chem Sci. 2025 Feb 4;16(11):4718–24. doi: 10.1039/d4sc07744j (PMC11831689; doi:10.1039/d4sc07744j)
Supplement: SC-016-D4SC07744J-s001 [file SC-016-D4SC07744J-s001.pdf]

Supplementary Information (SI) for Chemical Science.
This journal is © The Royal Society of Chemistry 2025

Borate-Catalysed Direct Amidation Reactions of Coordinating Substrates - Supporting Information

Richard J. Procter, Carla Alamillo-Ferrer, Usman Shabbir, Phyllida Britton, Dejan-Krešimir Bučar, Alexandre S. Dumon, Henry S. Rzepa,* Jordi Bures,* Andrew Whiting,* and Tom D. Sheppard*

Raw data files can be found in an online data repository associated with this paper, DOI: 10.14469/hpc/12218

Contents

General Information 2
Preliminary time course experiments 2
Optimisation of Catalysis 4
Synthesis of Borates 6
Synthesis of Amides 10
In-Situ generation of catalyst 27
Varying the ratio of phenol to borane 29
Borane dimethylsulfide catalyst control reaction 29
Larger scale amidation reactions 30
Inhibition with 2 -amino pyridine 31
Adduct formation between 2-amino pyridine and phenylboroxine 32
Interaction of 2-amino pyridine with 2-chlorophenylboroxine 33
Synthesis of 2-Chlorophenylboroxine 33
Reaction of 2-amino pyridine with 2-chlorophenylboroxine 33
Reaction of 2-amino pyridine with 2-chlorophenylboronic acid 34
Interaction of 2-amino pyridine with catalysts in ^{t}BuOAc 36
Analysis of reaction mixtures for catalyst protodeborylation 41
Characterisation data 46
Chiral HPLC traces 92
Enantiopurity determination with Marfey's reagent 97
Single crystal x-ray diffraction studies 98
Computational methods 100
References 101

General Information

All reagents were purchased from chemical suppliers and used as received unless otherwise stated. Reactions were carried out open to ambient air unless otherwise specified. ^{1}H, ^{13}C, ^{11}B and ^{19}F NMR spectra were recorded on Bruker Avance Neo 700, Avance III 600, Avance Neo 500 or Avance III 400 spectrometers. Chemical shifts are reported in parts per million (ppm) and referenced against residual solvent signals ($CDCl_3$ = 7.26 (^{1}H), 77.1 (^{13}C); DMSO = 2.49 (^{1}H), 39.5 (^{13}C); MeCN-d_3 = 1.97 (^{1}H), 1.32 (^{13}C)), ^{11}B and ^{19}F were referenced externally. Peaks are assigned as singlet(s), doublet(d), triplet(t), quartet(q), quintet(qn), septet(sept) or multiplet(m). Infrared spectra were obtained on a Bruker Alpha II compact FTIR Spectrometer operating in ATR mode. High resolution mass spectrometry was performed using a Thermo Vanquish LC connected to Q Exactive Plus Hybrid Quadrupole-Orbitrap mass spectrometer operating in ESI mode. Melting points were measured on a Gallenkamp heating block and are uncorrected**.** Column chromatography was performed on a Biotage Isolera one system using pre-packed flashpure cartridges.

Preliminary time course experiments

Data is available at 10.14469/hpc/14832

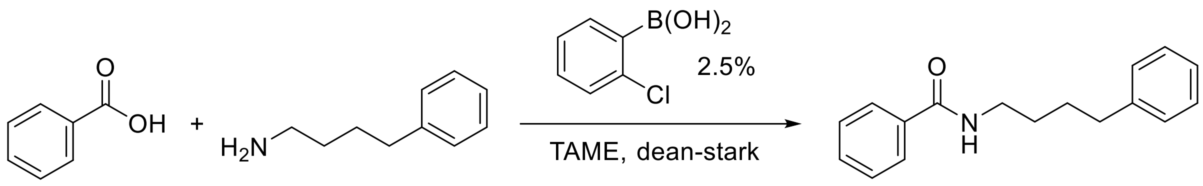

Stock solutions, made up to the prescribed volume with TAME, were used in the preparation of this reaction.

SL	Component	Volume / mL	Mass / mg	Concentration / M
**A**	BzOH	5.0	764.0	1.25
**B**	4-Phenylbutylamine	5.0	935.2	1.25
**C**	TMB	2.0	360.2	1.07
**D**	*o*-$ClC_6H_4B(OH)_2$	2.0	39.4	0.13

To a 25 mL three-neck flask containing a magnetic stirrer and fitted with a Dean-Stark condenser side-arm filled with TAME and wrapped with a heating tape (grounded silicone rubber heating tape BS0-G, BS0102040LG), was charged with stock solutions **A** (4.0 mL, 5.00 mmol, 1.0 equiv), **B** (4.0 mL, 5.01 mmol, 1.0 equiv) and **C** (1.0 mL, 1.07 mmol). The reaction mixture was heated to reflux (86 °C) before the addition of the catalyst stock solution **D** (1.0 mL, 0.13 mmol, 2.5 mol%). The reaction was monitored by offline sampling and analysing the data by quantitative ^{1}H NMR using TMB as the internal standard.

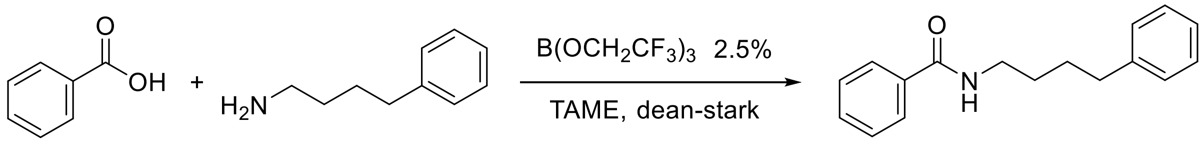

Stock solutions, made up to the prescribed volume with TAME, were used in the preparation of this reaction.

SL	Component	Volume / mL	Mass / mg	Concentration / M
**A**	BzOH	5.0	694.7	1.14
**B**	4-Phenylbutylamine	5.0	812.3	1.09
**C**	TMB	2.0	303.3	0.90

To a 25 mL three-neck flask containing a magnetic stirrer and fitted with a Dean-Stark condenser side-arm filled with TAME and wrapped with a heating tape (grounded silicone rubber heating tape BS0-G, BS0102040LG), was charged with stock solutions **A** (4.4 mL, 5.01 mmol, 1.0 equiv), **B** (4.6 mL, 5.01 mmol, 1.0 equiv) and **C** (1.0 mL, 0.90 mmol). The reaction mixture was heated to reflux (86 °C) before the addition of $B(OCH_2CF_3)_3$ (27 µL, 0.12 mmol, 2.5 mol%) in one portion due to low stability in open air of the catalyst at room temperature. The reaction was monitored by offline sampling and analysing the data by quantitative ^{1}H NMR using TMB as the internal standard.

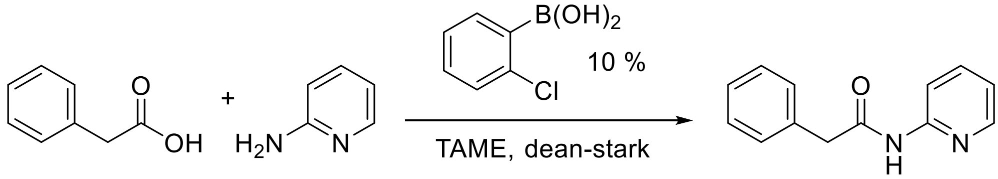

Stock solutions, made up to the prescribed volume with TAME, were used in the preparation of this reaction.

SL	Component	Volume / mL	Mass / mg	Concentration / M
**A**	Phenyl acetic acid	5.0	850.4	1.25
**B**	2-aminopyridine	5.0	588.4	1.25
**C**	TMB	2.0	297.0	0.88
**D**	(o-$ClC_6H_4)_2B(OH)$	2.0	156.4	0.50

To a 25 mL three-neck flask containing a magnetic stirrer and fitted with a Dean-Stark condenser side-arm filled with TAME and wrapped with a heating tape (grounded silicone rubber heating tape BS0-G, BS0102040LG), was charged with stock solutions **A** (4.0 mL, 5.00 mmol, 1.0 equiv), **B** (4.0 mL, 5.03 mmol, 1.0 equiv) and **C** (1.0 mL, 0.88 mmol). The reaction mixture was heated to reflux (86 °C) before the addition of the catalyst stock solution **D** (1.0 mL, 0.50 mmol, 10 mol%). The reaction was monitored by offline sampling and analysing the data by quantitative ^{1}H NMR using TMB as the internal standard.

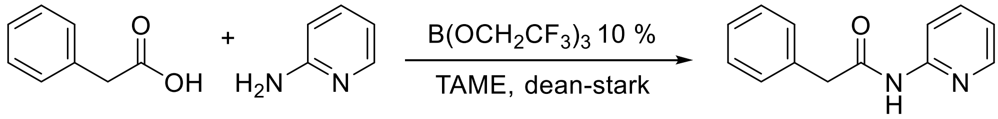

Stock solutions, made up to the prescribed volume with TAME, were used in the preparation of this reaction.

SL	Component	Volume / mL	Mass / mg	Concentration / M
**A**	Phenylacetic acid	5.0	775.6	1.14
**B**	2-aminopyridine	5.0	537.9	1.14
**C**	TMB	2.0	286.1	0.80

To a 25 mL three-neck flask containing a magnetic stirrer and fitted with a Dean-Stark condenser side-arm filled with TAME and wrapped with a heating tape (grounded silicone rubber heating tape BS0-G, BS0102040LG), was charged with stock solutions **A** (4.4 mL, 5.01 mmol, 1.0 equiv), **B** (4.4 mL, 5.03 mmol, 1.0 equiv) and **C** (1.1 mL, 0.94 mmol). The reaction mixture was heated to reflux (86 °C) before the addition of $B(OCH_2CF_3)_3$ (100 µL, 0.46 mmol, 10 mol%) in one portion due to low stability in open air of the catalyst at room temperature. The reaction was monitored by offline sampling and analysing the data by quantitative ^{1}H NMR using TMB as the internal standard.

Optimisation of Catalysis

General procedure

In a 50 ml round bottomed flask, benzoic acid (610 mg, 5 mmol) and 1,3,5-trimethoxybenzene (for use as internal standard, 84.1 mg, 0.5 mmol) were dissolved in 10 ml *tert*-Amyl methyl ether (TAME). Benzylamine (600 µl, 5.5 mmol) was added, and the reaction mixture was heated to 70 °C when the respective catalyst (0.5 mmol, either as neat liquid, solid or as a solution in TAME) was added. The flask was equipped with a Dean-Stark distillation apparatus with a filled side arm, and the reaction was brought to reflux for 18 hours. A sample of the homogenous reaction mixture was quenched with 1M HCl solution, extracted into DCM, dried under reduced pressure, redissolved in $CDCl_3$ and analysed by ^1H NMR spectroscopy.

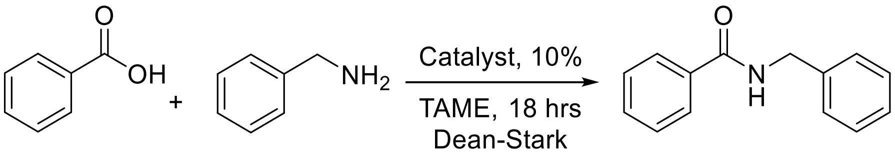

Catalyst	Yield[a] / %
$B(OCH_2CF_3)_3$	56
$B(OMe)_3$	36
$[-B(OMe)O-]_3$	38
$B(OiPr)_3$	27
$B(OHFIP)_3$	60
$B(OC(CF_3)_3)_3$	51
$B(OCH_2CCl_3)_3$	64
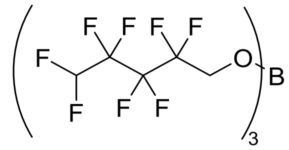	65
$B(OPh)_3$	52
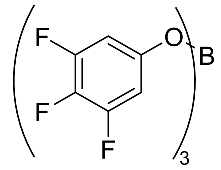	71
$B(OC_6Cl_5)_3$	51
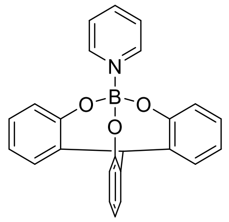	0
CatBH	34
Cl_4CatBH	57
$B(OH)_3$	20
PinBH	22
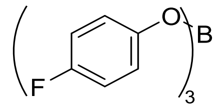	62

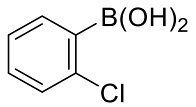	78

[a]Measured by ^{1}H NMR vs. trimethoxybenzene internal standard

In a 50 ml round bottomed flask, 3-fluorophenylacetic acid (771mg, 5 mmol) and 2-amino pyridine (518 mg, 5.5 mmol) were dissolved in 10 ml *tert*-Amyl methyl ether (TAME). The reaction mixture was heated to 70 °C, (becomes homogenous ~ 65°C) and the respective catalyst was added (0.5 mmol, either as neat liquid, solid or as a solution in TAME (0.25M)). The flask was then equipped with a Dean-Stark distillation apparatus with a filled side arm, and the reaction was brought to reflux for 18 hours. The reaction was quenched with saturated $NaHCO_3$, and acetanilide (135 mg, 1 mmol) was added to use as an internal standard. The mixture was extracted into DCM, dried under reduced pressure, redissolved in $CDCl_3$ and analysed by ^{1}H NMR spectroscopy.

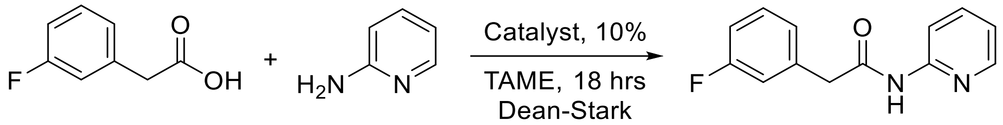

Catalyst	Yield / %
$B(OCH_2CF_3)_3$	20
$B(OiPr)_3$	26
$B(OHFIP)_3$	27
$B(OC(CF_3)_3)_3$	22
$B(OCH_2CCl_3)_3$	16
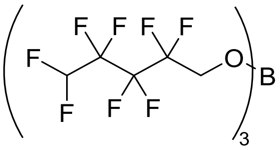	15
$B(OPh)_3$	27
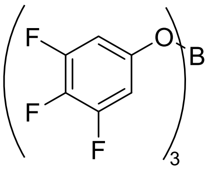	46
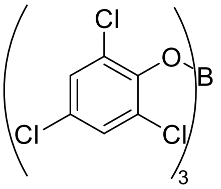	29
$B(OC_6Cl_5)_3$	26
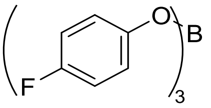	29
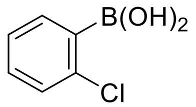	0
$B(OC_6F_5)_3$*	26

*Generated *in-situ* from $BH_3.SMe_2$ + 3 C_6F_5OH, according to the general procedure described for $B(OAr^F)_3$ *vide infra*.

Synthesis of Borates

Commercially available borates, boranes, and boronic acids were purchased from chemical suppliers and used as received. $B(OCH_2CF_3)_3$,[1] $B(OHFiP)_3$,[2, 3] Chlorocatechol borane[4], and DATB2 (F)[5] were synthesised according to literature procedures. 3,4,5 trifluorophenol was stored over 3Å molecular sieves prior to use in borate synthesis. Melting points for borates are not recorded due to their sensitivity to moisture in air leading to unreliable melting point measurements.

NMR data for $B(OHFiP)_3$ is available from 10.14469/hpc/14837, and for DATB2(F) is available from 10.14469/hpc/14842

***Tris*-(3,4,5-trifluorophenyl) borate (B)**

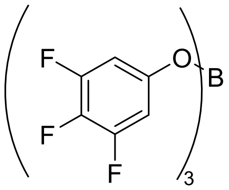

Procedure A (from BCl_3): In a flame dried Schlenk flask, under an atmosphere of argon, 3,4,5-trifluorophenol (7.26 g, 49 mmol) was dissolved in 30 ml anhydrous dichloromethane and cooled to -40 °C (precipitates). Boron trichloride solution (0.82 M in DCM, 20 ml, 16.333 mmol) (care, HCl evolution) was added dropwise and the reaction became homogenous. After 30 minutes the reaction was warmed to 0 °C with an ice/water bath (HCl evolves on warming), stirred for 30 minutes and warmed to room temperature. After 1 hour the reaction was concentrated under vacuum and the residue redissolved by warming in a mix of anhydrous pentane (10 ml) and DCM (4 ml), then cooled to -20 °C overnight, producing a crop of white crystals. The crystals were washed with pentane (1 ml) and dried under reduced pressure. Yield = 4.8 g, 10.6 mmol, 65%.

Procedure B (from $BH_3.SMe_2$): Under an atmosphere of argon in a flame dried 2 neck round bottom flask equipped with a reflux condenser, 3,4,5-trifluorophenol (10 g, 68 mmol), was dissolved in 15 ml anhydrous diethyl ether. The solution was cooled to 0 °C, and borane dimethylsulfide (2.6 ml, 2.6 mmol) was added dropwise and stirred for 30 minutes, after which the reaction was heated to reflux overnight. The solution was transferred to a Schlenk flask and concentrated under reduced pressure. Recrystallisation of the residue is typically more difficult than using procedure A, presumably due to residual dimethyl sulfide or borane species, and works most effectively using pentane/ether mixes (2:1). Yield 5.5g, 12.2 mmol, 47%.

Raw NMR data: https://doi.org/10.14469/hpc/14727

^{1}H NMR (500 MHz, $CDCl_3$) δ (ppm) = 6.77 (m, 6H)

^{13}C{^{1}H}NMR (126 MHz, $CDCl_3$) δ (ppm) = 151.3 (ddd, $J_{C\text{-}F}$ = 250.1, 10.5, 5.5 Hz), 146.9 (dt, $J_{C\text{-}F}$ = 11.9, 4.0 Hz), 137.4 (dt, $J_{C\text{-}F}$ = 248.6, 15.3, Hz), 105.3 (m)

^{11}B NMR (128 MHz, $CDCl_3$) δ (ppm) = 15.6 (s)

^{19}F NMR (376 MHz, $CDCl_3$) δ (ppm) = −132.66 (d, J = 21 Hz), −165.51 (t, J = 21 Hz).

HRMS: $[C_{18}H_6BO_3F_9 + OMe]^-$: Expect 483.05 observe 483.05

ν_{max} (solid/cm^{-1}): 3090, 1625, 1520, 1453

Tris-(2,2,3,3,4,4,5,5-octafluoropentyl) borate

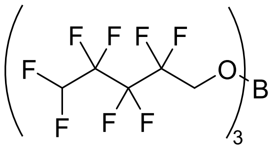

A flame dried two neck round bottomed flask was charged with borane dimethylsulfide (2.1 ml, 21 mmol), and cooled to 0 °C. 1H,1H,5H-octafluoro-1-pentanol (9 ml, 15.1g, 65 mmol) was added dropwise, and the reaction stirred overnight. The solution was then transferred to a distillation apparatus with the aid of 5 ml anhydrous pentane and volatiles were removed under reduced pressure. The product was distilled (111-115 °C, 2 mbar) to give a colourless oil. Yield = 13.27g, 18.85 mmol, 87%.

Raw NMR data: https://doi.org/10.14469/hpc/14728

^{1}H NMR (400 MHz, $CDCl_3$) δ (ppm) = 6.01 (tt, *J* = 52.0, 5.4 Hz, 3H), 4.34 (t, *J* = 13.3 Hz, 6H).

^{13}C{^{1}H} NMR (176 MHz, $CDCl_3$) δ (ppm) = 114.6 (tt, *J* = 256.5, 30.6 Hz), 111.0 (ttt, *J* = 264.4, 33.8, 30.9 Hz), 110.2 (ttt, *J* = 263.7, 31.3, 26.8Hz), 107.7 (tt, *J* = 253.9, 30.9 Hz).

^{11}B NMR (128 MHz, $CDCl_3$) δ (ppm) = 17.7

^{19}F{^{1}H} NMR (376 MHz, $CDCl_3$) δ (ppm) = −122.04 (m, 6F), −125.83 (m, 6F), −130.63 (m, 6F), −137.75 (m, 6F).

HRMS $[C_{15}H_9BF_{24}O_3]^-$: Calculated 704.0267, observed 704.0273

Tris-(2,2,2-trichloroethyl) borate

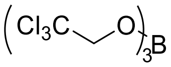

A flame dried two neck round bottomed flask was charged with borane dimethylsulfide (2 ml, 20 mmol), and cooled to 0 °C. Trichloroethanol (5.75 ml, 60 mmol) was added dropwise, and after 30 minutes the reaction was allowed to warm to room temperature. After stirring at this temperature for 30 minutes, the reaction was heated to 40 °C, and over a period of 1 hour a white solid formed. The reaction was allowed to stir at 40 °C overnight. The solid was recrystallised from hot anhydrous dichloromethane, producing 5.2 g white crystals (11.4 mmol, 57 %).

Raw NMR data: https://doi.org/10.14469/hpc/14729

^{1}H NMR (500 MHz, $CDCl_3$) δ (ppm) = 4.52 (s, 6H)

^{13}C{^{1}H} NMR (126 MHz, $CDCl_3$) δ (ppm) = 97.0, 75.9.

^{11}B NMR (160 MHz, $CDCl_3$) δ (ppm) = 17.7.

HRMS $[C_6H_6B^{35}Cl_9O_3+C_2H_5O]^-$: Calculated 496.7947, experimental 496.7954

***Tris*-(2,4,6-trichlorophenyl) borate (D)**

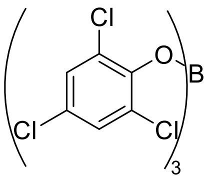

Tris(2,4,6-trichlorophenyl) borate was prepared by a modified literature procedure[6]. In a flame dried Schlenk tube, 2,4,6-trichlorophenol (5.33 g, 27 mmol) was dissolved in anhydrous dichloromethane (40 ml). The solution was cooled with a dry ice/acetonitrile bath (-41 °C), causing the phenol to precipitate, and neat boron tribromide (690 µl, 9 mmol) was added dropwise (HBr is evolved, and should be trapped by bubbling through NaOH solution. A homogenous solution was formed on addition of the BBr_3). After 45 minutes reaction, the cold bath was replaced with an ice/water bath (further HBr evolution on warming), and after a further 45 minutes reaction this was removed and the flask allowed to warm to room temperature. After 45 minutes at room temperature, the solvent was removed under reduced pressure, and the product was isolated by recrystallisation from a saturated solution in DCM. Yield = 2.02 g white crystals, 37%.

Raw NMR data: https://doi.org/10.14469/hpc/14730

^{1}H NMR (500 MHz, $CDCl_3$) δ (ppm) = 7.35 (s, 6H)

^{13}C{^{1}H} NMR (126 MHz, $CDCl_3$) δ (ppm) = 144.4, 130.1, 128.6, 127.9.

^{11}B NMR (160 MHz, $CDCl_3$) δ (ppm) = 15.5

HRMS $[C_{18}H_6B^{35}Cl_7{}^{35}Cl_2O_3]^-$: Calculated 599.7542, observed 599.7542

***Tris*-(pentachlorophenyl) borate**

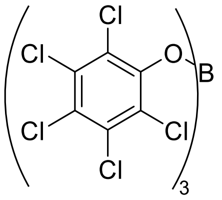

In a Schlenk flask under argon, pentachlorophenol (7.99 g, 30 mmol, 2.72 eq) was suspended in 20 ml anhydrous dichloromethane. The mixture was cooled to -78 °C and boron trichloride (1M in DCM, 1.1 ml, 11 mmol, 1 eq.) was added dropwise (care HCl evolution). After 30 minutes the cold bath was removed and the reaction was stirred at room temperature overnight. A further 20 ml anhydrous dichloromethane was added and the suspension filtered by cannula filtration, and the solid was washed with anhydrous THF (2 * 20ml). Due to poor solubility in standard NMR solvents, the product was characterised by mass spec only.

HRMS $[C_{18}B^{35}Cl_{12}{}^{37}Cl_3O_3]^-$: Expected 805.5185, observed 805.5187

***Tris*-(perfluoro-*tert*-butyl) borate**

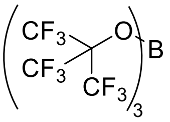

Under an atmosphere of argon, a flame dried 2-necked round bottom flask equipped with a condenser was charged with perfluoro-tert-butanol (7.5 ml, 12.75g, 54 mmol, 3.15 eq.) and cooled to 0 °C. Borane dimethylsulfide (1.7 ml, 17 mmol, 1 eq) was added dropwise, and after 30 minutes the cold bath was removed and the reaction heated to reflux overnight. After this time 1 ml further perfluoro-tert-butanol was added and reflux was continued for 2 hours. After this time the borate was purified by distillation (100 °C, 40 mbar) and collected as a white solid. The solid was melted by gentle heating and solid impurities were removed by filtration. Yield = 5.75 g colourless liquid, 47%. Due to poor solubility in typical NMR solvents, analysis was carried out in anhydrous diethyl ether. NMR data is in accordance with literature reports[7].

NMR data available from 10.14469/hpc/14833

$^{13}C\{^1H\}$NMR (126 MHz, $(C_2H_5)_2O$) δ (ppm) = 119.7 (q, $J_{C\text{-}F}$ = 290.3 Hz), 81.0–80.0 (m*, *J* = 35 Hz).

^{11}B NMR (160 MHz, $(C_2H_5)_2O$) δ (ppm) = 14.1 ppm

^{19}F NMR (376 MHz, $(C_2H_5)_2O$) δ (ppm) = −72.3 ppm

*This signal is presumably a decet but due to the low intensities not all of the peaks can be clearly seen in the spectrum.

Synthesis of Amides

General Procedure

A 50 ml round bottomed flask was charged with carboxylic acid (5 mmol), amine (5.5 mmol), and *tert*-butyl acetate (4 ml), and heated to 80 °C. Once this temperature was reached $B(OAr^F)_3$ (0.5 mmol, in solution in 1 ml tBuOAc) was added, the flask equipped with a Dean-Stark distillation apparatus with a filled side arm, and the reaction was brought to reflux for the indicated time. Reactions were quenched with a saturated $NaHCO_3$ solution, extracted into DCM, and washed with 1M NaOH followed by 1M HCl (for basic substrates this step was omitted). The organic phase was dried over Na_2SO_4, filtered, dried under reduced pressure and purified by column chromatography.

***N*-Benzylbenzamide (3)**

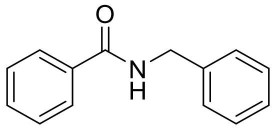

Synthesised according to the general procedure, using 5 mol% or 2 mol% catalyst loading. Purified by flash column chromatography (EtOAc:Cyclohexane 30% → 60%). Analytical data in accordance with literature reports.[8] Yield = 923 mg white solid, 4.37 mmol, 87%

Raw NMR data: https://doi.org/10.14469/hpc/14636

^1H NMR (700 MHz, $CDCl_3$): δ (ppm) = 7.79 (d, *J* = 7.6 Hz, 2H), 7.50 (t, *J* = 7.3 Hz, 1H), 7.43 (t, *J* = 7.6 Hz, 2H), 7.36 (m, 4H), 7.33-7.28 (m, 1H), 6.46 (s, 1H), 4.65 (d, *J* = 5.6 Hz, 2H).

^{13}C NMR (176 MHz, $CDCl_3$): δ (ppm) = 167.48, 138.31, 134.52, 131.70, 128.94, 128.74, 128.07, 127.78, 127.09, 44.28.

HRMS $[C_{14}H_{13}NO+H]^+$ Expected 212.1081 Observed 212.1068

Melting point: 92–93 °C

2-(3-Fluorophenyl)-N-(pyridin-2-yl)acetamide (4)

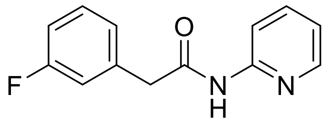

Synthesised according to the general procedure, purified by flash column Chromatography (EtOAc : cyclohexane, 15% → 50%). Yield = 940 mg white solid, 82%.

Raw NMR data: https://doi.org/10.14469/hpc/14710

^1H NMR (700 MHz, $CDCl_3$) δ (ppm) = 8.43 (s, 1H), 8.22 (m, 2H), 7.71 (ddd, *J* = 8.4, 7.4, 1.9, Hz, 1H), 7.32 (td, *J* = 8.0, 6.0 Hz, 1H), 7.09 (d, *J* = 7.6 Hz, 1H), 7.04 (m, 2H), 7.00 (td, *J* = 8.5, 2.5 Hz, 1H), 3.74 (s, 1H).

^{13}C NMR (176 MHz, $CDCl_3$) δ (ppm) = 169.0 163.2 (d, $J(^1CF)$ = 247.0 Hz), 151.3, 147.6, 138.8, 136.4 (d, $J(^3CF)$ = 7.6 Hz), 130.7 (d, $J(^3CF)$ = 8.3 Hz), 125.2 (d, $J(^4CF)$ = 2.9 Hz), 120.2, 116.6 (d, $J(^2CF)$ = 21.6 Hz), 114.8 (d, $J(^2CF)$ = 21.0 Hz), 114.4), 44.5.

^{19}F NMR (659 MHz, $CDCl_3$) δ (ppm) = −112.1 (m, 1F).

HRMS $[C_{13}H_{11}FN_2O+H]^+$; Expected: 231.0928, observed: 231.0924

ν_{max} (solid/cm^{-1}): 3241, 3188, 3114, 3081, 3033, 1687, 1615, 1578, 1524, 1487, 1460, 1436

Melting Point: 108–111 °C

***N*-(2-(Dimethylamino)-5-(trifluoromethyl)phenyl)-2-phenylacetamide (5)**

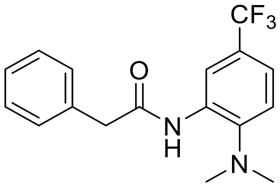

Synthesised according to the general procedure, purified by flash column chromatography (EtOAc:Cyclohexane 5% → 50%). Yield = 1.32 g pale pink solid, 4.09 mmol, 82%

Raw NMR data: https://doi.org/10.14469/hpc/14711

^{1}H NMR (700 MHz, $CDCl_3$): δ (ppm) = 8.69 (d, *J* = 1.6 Hz, 1H), 8.34 (s, 1H), 7.41 (m, 2H), 7.35 (m, 3 H), 7.25 (dd, *J* = 7.9, 1.3 Hz, 1H), 7.10 (d, *J* = 8.2 Hz, 1H), 3.79 (s, 2H), 2.35 (s, 6H).

^{13}C NMR (176MHz, $CDCl_3$): δ (ppm) = 169.3, 145.8, 134.6, 133.5, 129.8, 129.3, 127.8, 126.8 (q, J_{C-F} = 32.5 Hz), 124.3 (q, J_{C-F} = 271.8 Hz), 120.7 (q, J_{C-F} = 3.8 Hz), 120.0, 116.2 (q, J_{C-F} = 3.8 Hz), 45.5, 44.0.

^{19}F NMR (659 MHz, $CDCl_3$) δ (ppm) = −62.15 (s).

HRMS $[C_{17}H_{17}F_3N_2O + H]^+$: Expect 323.1371 observe 323.1360

ν_{max} (solid/cm^{-1}): 3285, 3075, 3038, 2985, 2945, 2870, 2840, 2795, 1664, 1614, 1585, 1531, 1481, 1468, 1425.

Melting point: 86–88 °C

2-Phenyl-*N*-(quinolin-8-yl)acetamide (6)

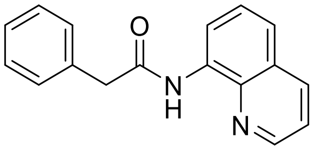

Synthesised according to the general procedure. Purified by flash column chromatography (EtOAc : Cyclohexane 10% → 30%). Yield = 1.16g off-white solid, 4.42 mmol, 88%. Analytical data is in accordance with literature reports.[9]

Raw NMR data: https://doi.org/10.14469/hpc/14712

^{1}H NMR (700 MHz, $CDCl_3$) δ (ppm) = 9.92 (s, 1H), 8.76 (d, *J* = 7.5 Hz, 1H), 8.69 (dd, *J* = 4.2, 1.5 Hz, 1H), 8.11 (dd, *J* = 8.2, 1.4 Hz, 1H), 7.51 (t, *J* = 7.9 Hz, 1H), 7.47 (dd, *J* = 8.1, 1 Hz, 1H), 7.45 (d, *J* = 7.5 Hz, 2H), 7.40 (m, 3H), 7.33 (t, *J* = 7.3 Hz, 1H), 3.90 (s, 2H).

^{13}C NMR (176 MHz, $CDCl_3$) δ (ppm) = 169.7, 148.2, 138.5, 136.5, 134.8, 134.59, 129.7, 129.1, 128.0, 127.5, 127.5, 121.75, 121.7, 116.6, 45.5

HRMS $[C_{17}H_{14}N_2O+H]^+$: Expected 263.1179, Observed 263.1178

Melting point : 71–73 °C

***N*-Benzyl-1H-indazole-3-carboxamide (7)**

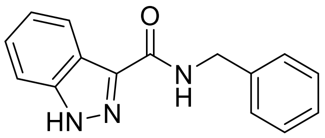

Synthesised according to the general procedure, reaction time 44 hrs. Purified by flash column chromatography (MeOH in DCM, 0% → 4%), isolated as a white solid. Yield = 1.11 g, 4.4 mmol, 88%.

Raw NMR data: https://doi.org/10.14469/hpc/14713

^{1}H NMR (700 MHz, DMSO) δ (ppm) = 13.59 (s, 1H), 8.96 (t, *J* = 6.3 Hz, 1H), 8.17 (d, *J* = 8.1 Hz, 1H), 7.61 (d, *J* = 8.5 Hz, 1H), 7.40 (t, *J* = 7.3 Hz, 1H), 7.35 (d, *J* = 7.5 Hz, 2H), 7.31 (t, *J* = 7.6 Hz, 2H), 7.22 (m, 2H), 4.49 (d, *J* = 6.3 Hz, 2H)

^{13}C NMR (176 MHz, DMSO) δ (ppm) = 162.4, 141.1, 140.0, 138.2, 128.3, 127.3, 126.7, 126.5, 122.1, 121.6, 121.6, 110.7, 41.9.

HRMS $[C_{15}H_{13}N_3O+H]^+$: Expected 252.1131, observed 252.1135

ν_{max} (solid/cm^{-1}): 3406, 3160, 3125, 3083, 3053, 3026, 2916, 1644, 1535, 1505, 1469, 1452, 1429

Melting point: 169–170 °C

***tert*-Butyl 4-(quinolin-8-ylcarbamoyl)piperidine-1-carboxylate (8)**

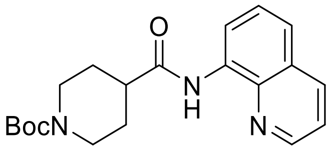

Synthesised according to the general procedure. Purified by flash column chromatography (EtOAc : Cyclohexane 10% → 50%). Yield = 1.38g, brown solid, 3.88 mmol, 78%. Analytical data is in accordance with literature reports.[10]

Raw NMR data: https://doi.org/10.14469/hpc/14714

^{1}H NMR (700 MHz, $CDCl_3$) δ (ppm) = 9.94 (s, 1H), 8.80 (dd, *J* = 4.2, 1.4 Hz, 1H), 8.77 (dd, *J* = 7.4, 0.9 Hz, 1H), 8.16 (dd, *J* = 8.2, 1.4 Hz, 1H), 7.53 (t, *J* = 7.8 Hz, 1H), 7.50 (dd, *J* = 8.1, 1.2 Hz, 1H), 7.46 (dd, *J* = 8.3, 4.2 Hz, 1H), 4.22 (br s, 2H), 2.86 (br s, 2H), 2.62 (tt, *J* = 11.5, 3.7 Hz, 1H), 2.03 (d, *J* = 11.3 Hz, 2H), 1.83 (qd, *J* = 12.2, 3.6 Hz, 2H), 1.48 (s, 9H)

^{13}C NMR (176 MHz, $CDCl_3$) δ (ppm) = 173.1, 154.9, 148.3, 138.6, 136.6, 134.5, 128.1, 127.6, 121.8, 121.7, 116.7, 79.8, 44.9, 43.8, 43.1, 28.9, 28.6.

HRMS $[C_{20}H_{25}N_3O_3+H]^+$: Calculated 356.1969, Observed 356.1964

Melting point: 103–105 °C

N-(2-(Dimethylamino)ethyl)-2-phenylacetamide (9)

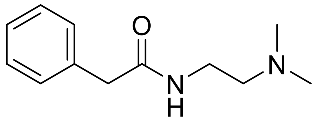

Synthesised according to the general procedure. Purified by flash column chromatography (MeOH in DCM, 5 → 25 %, plus 1% NEt_3). Yield = 926 mg, cream coloured hygroscopic solid, 4.49 mmol, 90%.

Raw NMR data: https://doi.org/10.14469/hpc/14715

^{1}H NMR (600 MHz, $CDCl_3$) δ (ppm) = 7.33 (m, 2H), 7.27 (m, 3 H), 6.03 (s, 1H), 3.55 (s, 2H), 3.28 (q, J = 5.8 Hz, 2H), 2.33 (t, J = 6.0 Hz, 2H), 2.15 (s, 6 H).

^{13}C NMR (151 MHz, $CDCl_3$) δ (ppm) = 171.1, 135.2, 129.4, 128.9, 127.2, 57.5, 45.2, 43.8, 37.1.

HRMS $[C_{12}H_{18}N_2O + H]^+$; Calculated 207.1492, observed 207.1488

ν_{max} (solid/cm^{-1}): 3921, 3080, 3060, 3027, 2982, 2976, 2941, 2815, 2761, 1636, 1550, 1493, 1466, 1452, 1446

2-Phenyl-*N*-(1H-pyrazol-3-yl)acetamide (10)

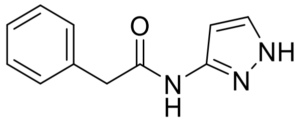

Synthesised according to the general procedure. Purified by recrystallisation from hot methanol/water (2:1, 21 ml). Yield = 753 mg white needle crystals, 3.75 mmol, 75%. Analytical data is in accordance with literature reports.[11]

Raw NMR data: https://doi.org/10.14469/hpc/14716

^{1}H NMR (700 MHz, DMSO) δ(ppm) = 12.30 (s, 1H), 10.59 (s, 1H), 7.56 (s, 1H), 7.30 (m, 4H), 7.22 (t, *J* = 6.7 Hz, 1H), 6.46 (s, 1H), 3.59 (s, 2H).

^{13}C NMR (176 MHz, DMSO) δ(ppm) = 168.2, 147.4, 136.2, 129.1, 128.6, 128.3, 126.5, 95.9, 42.5.

HRMS $[C_{11}H_{11}N_3O+H]^+$: Expected 202.0975 Observed 202.0971

Melting point: 150–152 °C

***N*-Phenylpicolinamide (11)**

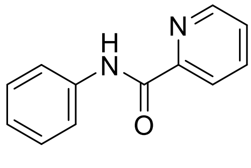

Synthesised according to the general procedure. After aqueous work-up, the crude oil was triturated with pentane and dried under vacuum to yield an orange/brown crystalline solid. Yield = 427 mg, 43%. Analytical data is in accordance with literature reports.[12]

Raw NMR data: https://doi.org/10.14469/hpc/14717

^1H NMR (700 MHz, $CDCl_3$) δ(ppm) = 10.04 (s, 1H), 8.61 (d, *J* = 4.1 Hz, 1H), 8.31 (d, *J* = 7.8 Hz, 1H), 7.91 (t, *J* = 7.6 Hz, 1H), 7.79 (d, *J* = 7.9 Hz, 2H), 7.48 (dd, *J* = 7.7, 5.1 Hz, 1H), 7.39 (t, *J* = 7.7 Hz, 2H), 7.15 (t, *J* = 7.4 Hz, 1H).

^{13}C NMR (176 MHz, $CDCl_3$) δ(ppm) = 162.1, 149.9, 148.0, 137.9, 137.9, 129.2, 126.6, 124.5, 122.6, 119.8.

HRMS $[C_{12}H_{10}N_2O+H]^+$: Expected 199.0866 Observed 199.0866

Melting point: 71–73 °C

***N*-(3-Bromophenyl)-1H-pyrazole-3-carboxamide (12)**

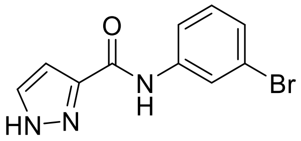

Synthesised according to the general procedure, using toluene as the reaction solvent with 24 hours reaction time. Purified by recrystallisation on cooling of the reaction mixture, followed by washing with toluene (2 x 10 ml) and trituration with Et_2O (2 x 10 ml). Further pure material is isolated from the mother liquor by recrystallisation from hot toluene. Yield = 857 mg, white solid, 3.22 mmol, 64%.

Raw NMR data: https://doi.org/10.14469/hpc/14718

^1H NMR (700 MHz, DMSO-d_6) (major component) δ(ppm) = 13.45 (s, 1H), 10.24 (s, 1H), 8.15 (s, 1H), 7.90 (s, 1H), 7.81 (d, *J* = 7.5 Hz, 1H), 7.25 (m, 2H), 6.77 (s, 1H).

^{13}C NMR (176 MHz, DMSO-d_6) δ(ppm) = 160.8, 146.4, 140.6, 130.6 (2C, overlapping peaks), 125.9, 122.4, 121.4, 119.0, 105.9

HRMS $[C_{10}H_9ON_3{}^{79}Br]^+$: Expect 265.9924, Observed 265.9927

ν_{max} (solid/cm^{-1}): 3368, 3283, 1676, 1585, 1532, 1478, 1450, 1433

Melting Point: 146–149 °C

***N*-Benzylpicolinamide (13)**

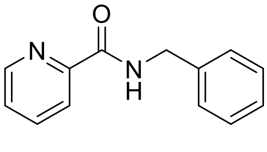

Synthesised according to the general procedure using 5 mol % borate catalyst. Purified by flash column chromatography (EtOAc : Cyclohexane 20% → 40%). Yield = 1.022 g, white solid 4.82 mmol, 96%. Analytical data is in accordance with literature reports.[13]

Raw NMR data: https://doi.org/10.14469/hpc/14719

^1H NMR (700 MHz, $CDCl_3$) δ (ppm) = 8.52 (d, *J* = 4.7 Hz, 1H), 8.40 (s, 1H), 8.24 (d, *J* = 7.8 Hz, 1H), 7.85 (td, *J* = 7.7, 1.7 Hz, 1H), 7.42 (ddd, *J* = 7.6, 4.7, 1.1 Hz, 1H), 7.35 (m, 4H), 7.28 (tt, *J* = 7.2, 1.4 Hz, 1H), 4.67 (d, *J* = 6.2 Hz, 2H).

^{13}C NMR (176 MHz, $CDCl_3$) δ (ppm) = 164.3, 149.9, 148.2, 138.4, 137.6, 128.8, 128.0, 127.6, 126.4, 122.5, 43.6.

HRMS $[C_{13}H_{12}N_2O+H]^+$; expected : 213.1022, observed : 213.1021

Melting point: 75 – 76 °C

_N_-Benzyl-2-(2-chlorophenyl)-2-hydroxyacetamide (14)

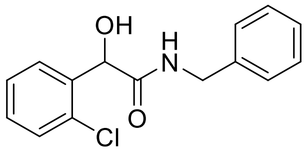

Synthesised according to the general procedure, with 40 hours reaction time. Purified by flash column chromatography (EtOAc : Hexanes 30 → 70 %). Yield = 638 mg, white solid, 2.32 mmol, 46%. Analytical data is in accordance with literature values.[14]

Raw NMR data: https://doi.org/10.14469/hpc/14720

^{1}H NMR (700 MHz, $CDCl_3$): δ (ppm) = 7.47 (dd, *J* = 7.7, 1.8 Hz, 1H), 7.38 (dd, *J* = 7.8, 1.3 Hz, 1H), 7.30 (m, 3H), 7.26 (m, 2H), 7.17 (d, *J* = 7.8 Hz, 2H), 6.55 (br s, 1H), 5.55 (d, *J* = 4.6 Hz, 1H), 4.51 (dd, *J* = 14.9, 5.9 Hz, 1H), 4.42 (dd, *J* = 14.9, 5.8 Hz, 1H), 4.10 (d, *J* = 4.6 Hz, 1H).

^{13}C NMR (176 MHz, DMSO-d_6): δ (ppm) = 171.3, 139.6, 139.1, 132.7, 129.3, 129.2, 129.0, 128.2, 127.3, 127.1, 126.7, 70.5, 42.0.

HRMS $[C_{15}H_{14}ClNO_2 + H^+]$: Calculated 276.0786, observed 276.0789

Melting point: 81–84 °C

_N_-Cyclohexylpicolinamide (15)

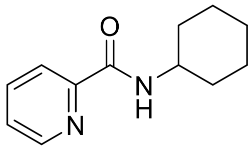

Synthesised according to the general procedure, reaction for 24 hours. Purified by flash column chromatography (EtOAc in Cyclohexane 30 → 60 %). Yield = 993 mg, white solid, 4.86 mmol, 97%. Data is in accordance with reported values.[15]

Raw NMR data: https://doi.org/10.14469/hpc/14721

^{1}H NMR (700 MHz, $CDCl_3$) δ (ppm) = 8.53 (d, *J* = 4.3 Hz, 1H), 8.19 (d, *J* = 7.8 Hz, 1H), 7.94 (br s, 1H), 7.82 (t, *J* = 7.6 Hz, 1H), 7.40 (dd, *J* = 6.8, 5.3 Hz, 1H), 3.96 (m, 1H), 2.00 (dd, *J* = 12.3, 2.6 Hz, 2H), 1.76 (dt, *J* = 13.4, 3.5 Hz, 2H), 1.64 (m, 1H), 1.42 (qt, J = 11.8, 3.5 Hz, 2H), 1.31 (m, 2H), 1.23 (m, 1H).

^{13}C NMR (176 MHz, $CDCl_3$) δ (ppm) = 163.4, 150.4, 148.1, 137.5, 126.1, 122.3, 48.3, 33.2, 25.7, 25.0.

Melting point: 59–61 °C

N-Benzyl-1H-pyrazole-3-carboxamide (16)

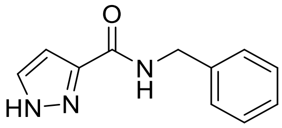

Synthesised according to the general procedure, purified by flash column chromatography (Acetone : cyclohexane 20% → 80%). Yield 623 mg, white powder, 62%. Data is in accordance with reported values.[16]

Raw NMR data: https://doi.org/10.14469/hpc/14722

^{1}H NMR (400 MHz, DMSO) δ (ppm) = 13.30 (br s, 1H), 8.75 (br s, 1H), 7.78 (br s, 1H), 7.34 – 7.28 (m, 4H), 7.28 – 7.20 (m, 1H), 6.69 (br s, 1H), 4.43 (d, *J* = 6.3 Hz, 2H).

^{13}C NMR (151 MHz, DMSO) δ (ppm) = 161.6, 146.5, 139.9, 130.1, 128.3 (2C, overlapping peaks), 127.3 (2C, overlapping peaks), 126.7, 105.1, 41.9.

HRMS $[C_{11}H_{11}N_3O+H]^+$: Expect 202.0975, Observe 202.0978

Melting point: 140–145 °C

N-(2-(Dimethylamino)phenyl)-2-phenylacetamide (17)

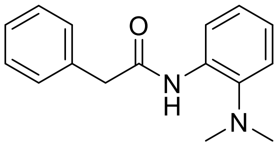

Synthesised according to the general procedure. Purified by flash column chromatography (EtOAc : cyclohexane 10 % → 30%). Yield = 1.16 g, pale pink solid 4.46 mmol, 91%.

Raw NMR data: https://doi.org/10.14469/hpc/14723

^{1}H NMR (700 MHz, $CDCl_3$) δ (ppm) = 8.48 (s, 1H), 8.35 (d, *J* = 8.1 Hz, 1H), 7.40 (t, *J* = 7.5 Hz, 2H), 7.35 (m, 3H), 7.07 (m, 2H), 6.99 (t, *J* = 7.5 Hz, 1H), 3.77 (s, 2H), 2.33 (s, 6H).

^{13}C NMR (176 MHz, $CDCl_3$) δ (ppm) = 169.1, 142.9, 135.0, 133.5, 129.8, 129.2, 127.6, 125.1, 123.8, 120.0, 119.1, 45.6, 44.5.

HRMS $[C_{16}H_{18}N_2O+H]^+$; Expected = 255.1492, Observed = 255.1496

ν_{max} (solid/cm^{-1}): 3266, 3029, 2977, 2940, 2854, 2825, 2785, 1665, 1591, 1509, 1492, 1474, 1452, 1416

Melting point: 46–48 °C

2-(3-Fluorophenyl)-*N*-(pyridin-3-yl)acetamide (18)

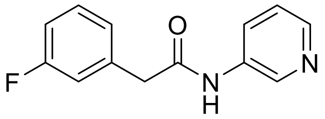

Synthesised according to the general procedure, reaction for 24 hours. Purified by flash column chromatography (EtOAc in cyclohexane, 60 → 100%). Yield = 791mg, white solid, 69%.

Raw NMR data: https://doi.org/10.14469/hpc/14724

^{1}H NMR (600 MHz, $CDCl_3$) δ (ppm) = 8.46 (d, *J* = 2.5 Hz, 1H), 8.34 (d, *J* = 4.6 Hz, 1H), 8.13 (d, *J* = 8.3 Hz, 1H), 7.38 (dd, *J* = 13.8, 7.9 Hz, 2H), 7.27 (dd, *J* = 7.2, 3.4 Hz, 1H), 7.13 (d, *J* = 7.6 Hz, 1H), 7.09 – 7.02 (m, 2H), 3.76 (s, 2H).

^{13}C NMR (151 MHz, $CDCl_3$) δ (ppm) = 168.9, 163.1 (d, *J*(^{1}CF) = 247.7 Hz), 145.6, 141.1, 136.2 (d, *J*(^{3}CF) = 7.5 Hz), 134.4, 130.9 (d, *J*(^{3}CF) = 8.4 Hz), 127.4, 125.2 (d, *J*(^{3}CF) = 2.9 Hz), 123.8, 116.6 (d, *J*(^{2}CF) = 21.6 Hz), 114.9 (d, *J*(^{2}CF) = 20.9 Hz), 44.4.

^{19}F NMR (659 MHz, $CDCl_3$) δ (ppm) = −111.7 (m, 1F).

HRMS $[C_{13}H_{11}FN_2O+H]^+$: Expected 231.0928, observed 231.0921.

ν_{max} (solid/cm^{-1}): 3102, 3051, 1658, 1604, 1581, 1502, 1485, 1449, 1417.

Melting point: 105–107 °C.

***tert*-Butyl 4-(pyridin-3-ylcarbamoyl)piperidine-1-carboxylate (19)**

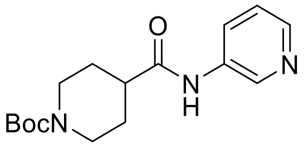

Synthesised according to the general procedure, reaction for 24 hours. Purified by flash column chromatography (EtOAc in cyclohexane, 0 → 20%). Yield = 784 mg, off-white solid, 51%. Data is in accordance with literature reports.[17]

Raw NMR data: https://doi.org/10.14469/hpc/14725

^{1}H NMR (700 MHz, $CDCl_3$) δ (ppm) = 8.54 (d, *J* = 2.2 Hz, 1H), 8.35 (d, *J* = 4.5 Hz, 1H), 8.19 (d, *J* = 8.2 Hz, 1H), 7.32 (br s, 1H), 7.28 (dd, *J* = 8.3, 4.7 Hz, 1H), 4.21 (br s, 2H), 2.79 (br s, 2H), 2.43 (tt, *J* = 11.6, 3.6 Hz, 1H), 1.91 (d, *J* = 12.3 Hz, 2H), 1.75 (qd, *J* = 12.4, 4.3 Hz, 2H), 1.46 (s, 9H).

^{13}C NMR (176 MHz, $CDCl_3$) δ (ppm) = 173.2, 154.8, 145.6, 141.2, 134.7, 127.4, 123.9, 79.9, 44.4, 28.7, 28.6.

HRMS $[C_{16}H_{23}N_3O_3+H]^+$: Expected 306.1812, observed 306.1808

ν_{max} (solid/cm^{-1}): 3243, 3178, 2961, 2847, 1688, 1611, 1581, 1550, 1477, 1450, 1424.

Melting point: 164–166 °C.

N-(3,5-Bis(trifluoromethyl)phenyl)-5-bromofuran-2-carboxamide (20)

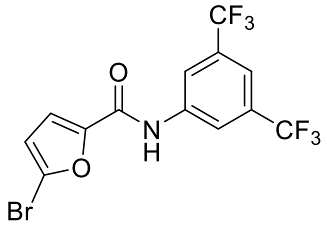

Synthesised according to the general procedure using toluene as the reaction solvent, reaction for 24 hours. Purified by flash column chromatography (EtOAc 5% → 25% in cyclohexane) Yield = 1.62 g cream solid, 81%.

Raw NMR data: https://doi.org/10.14469/hpc/14726

^{1}H NMR (700 MHz, $CDCl_3$) δ (ppm) = 8.25 (s, 1H), 8.18 (s, 2H), 7.65 (s, 1H), 7.25 (d, J = 3.6 Hz, 1H), 6.55 (d, J = 3.6 Hz, 1H).

^{13}C NMR (176 MHz, $CDCl_3$) δ (ppm) = 155.1, 148.4, 138.7, 132.7 (q, $J_{C\text{-}F}$ = 33.6 Hz), 126.0, 123.1 (q, $J_{C\text{-}F}$ = 272.9 Hz), 119.8 (q, $J_{C\text{-}F}$ = 3.7 Hz), 119.0, 118.1 (sept, $J_{C\text{-}F}$ = 3.7 Hz), 115.2.

^{19}F NMR (659 MHz, $CDCl_3$) δ (ppm) = −63.04 (s, 1F)

HRMS $[C_{13}H_6F_6NO_2{}^{79}Br + H]^+$: Expect 401.9559, observed 401.9570

ν_{max} (solid/cm^{-1}): 3297, 3095, 1657, 1628, 1586, 1547, 1467, 1439

Melting point: 121–122 °C

N-(3-Bromophenyl)benzamide (21)

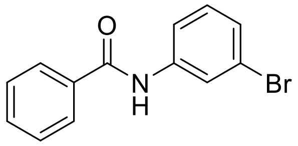

Synthesised according to the general procedure, using toluene as the reaction solvent. Purified by flash column chromatography (EtOAc:Cyclohexane 5 → 30 %). Yield = 1.16 g, white solid, 84%. Analytical data is in accordance with literature reports.[18]

Raw NMR data: https://doi.org/10.14469/hpc/14731

^{1}H NMR (700 MHz, $CDCl_3$) δ (ppm) = 7.99 (s, 1H), 7.90 (t, J = 1.9 Hz, 1H), 7.83 (m, 2H), 7.54 (m, 2H), 7.46 (t, J = 7.8 Hz, 2H), 7.26 (m, 1H), 7.20 (t, J = 8.0 Hz, 1H).

^{13}C NMR (176 MHz, $CDCl_3$) δ (ppm) = 166.0, 139.3, 134.6, 132.2, 130.5, 129.0, 127.7, 127.2, 123.3, 122.8, 118.9.

HRMS $[C_{13}H_{10}NO^{79}Br+H]^+$: Expected 276.0019, Observed 276.0019

Melting point: 133–135 °C

N-(3,5-Bis(trifluoromethyl)phenyl)-2-phenylacetamide (22)

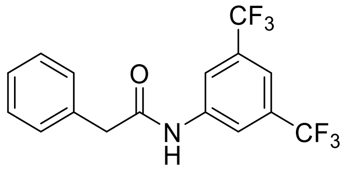

Synthesised according to the general procedure using toluene as reaction solvent, with 46 hours reaction time. Purified by flash column chromatography (EtOAc : Cyclohexane 5 → 50%). Yield = 1.625 g, white solid, 94%. Analytical data is in accordance with literature reports.[15]

Raw NMR data: https://doi.org/10.14469/hpc/14732

^{1}H NMR (700 MHz, $CDCl_3$) δ (ppm) = 7.93 (s, 2H), 7.58 (s, 1H), 7.43 (t, J = 7.4 Hz, 2H), 7.38 (m, 2H), 7.33 (d, J = 7.6 Hz, 2H), 3.78 (s, 2H).

^{13}C NMR (176 MHz, $CDCl_3$) δ (ppm) = 169.6, 139.1, 133.6, 132.5 (q, $J_{C\text{-}F}$ = 33.6 Hz), 129.6, 129.6, 128.3, 123.12 (q, $J_{C\text{-}F}$ = 272.8 Hz), 119.6 (m), 117.9 (sept, $J_{C\text{-}F}$ = 3.6 Hz), 44.9

^{19}F NMR (659 MHz, $CDCl_3$) δ (ppm) = −63.04.

HRMS $[C_{16}H_{10}NOF_6]^-$: Expected 346.0672, observed 346.0668

Melting point: 126–127 °C

N-Allyl-N-phenyl,2-phenylacetamide (23)

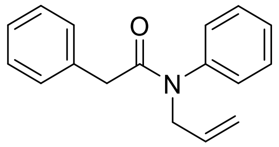

Synthesised according to the general procedure, using toluene as the reaction solvent, at 0.5 M concentration. Purified by flash column chromatography (EtOAc : Cyclohexane 10 → 65%). Yield = 903 mg, yellow oil, 72%. Analytical data is in accordance with literature reports.[1]

Raw NMR data: https://doi.org/10.14469/hpc/14733

^{1}H NMR (700 MHz, $CDCl_3$) δ (ppm) = 7.37 (m, 3H), 7.23 (t, J = 7.3 Hz, 2H), 7.19 (t, J = 7.2 Hz, 1H), 7.08 (d, J = 7.3 Hz, 2H), 7.05 (d, J = 7.3 Hz, 2H), 5.86 (ddt, J = 17.2, 10.2, 6.4 Hz, 1H), 5.09 (d, J = 10.1 Hz, 1H), 5.04 (d, J = 17.1 Hz, 1H), 4.30 (d, J = 6.3 Hz, 2H), 3.45 (s, 2H).

^{13}C NMR (176 MHz, $CDCl_3$) δ 170.7, 142.5, 135.5, 133.2, 129.6, 129.2, 128.8, 128.4, 128.2, 126.7, 118.1, 52.6, 41.4.

HRMS $[C_{17}H_{17}NO+H]^+$: Expected 252.1383 Observed 252.1377

_N_-Methyl-_N_-phenylbenzamide (24)

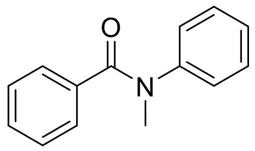

Synthesised according to the general procedure using toluene as reaction solvent for 23 hours. Purified by flash column chromatography (EtOAc 20% → 60% in cyclohexane) Yield = 440 mg brown oil, 42%. Analytical data is in accordance with reported literature values.[19]

Raw NMR data: https://doi.org/10.14469/hpc/14734

^{1}H NMR (700 MHz, $CDCl_3$) δ (ppm) = 7.29 (d, J = 7.5 Hz, 2H), 7.24-7.20 (m, 3H), 7.16-7.12 (m, 3H), 7.03 (d, J = 7.9Hz, 2H), 3.50 (s, 3H).

^{13}C NMR (176 MHz, $CDCl_3$) δ (ppm) = 170.8, 145.0, 136.0, 129.7, 129.3, 128.8, 127.9, 127.0, 126.6, 38.5.

_N_-Cyclohexylcyclopropanecarboxamide (25)

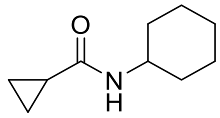

Synthesised according to the general procedure. Purified by flash column chromatography (EtOAc : cyclohexane, 25% → 85%). Yield = 812 mg white solid, 97%. Data in accordance with previous literature reports.[20]

Raw NMR data: https://doi.org/10.14469/hpc/14735

^{1}H NMR (700 MHz, $CDCl_3$): δ (ppm) = 5.54 (s, 1H), 3.77 (tdt, J = 12 Hz, 11 Hz, 4 Hz, 1H), 1.91 (dddd, J = 12.5 Hz, 4 Hz, 3.9 Hz, 3.5 Hz, 2H), 1.69 (dtt, J = 13.5, 3.9, 3.9 Hz, 2H), 1.60 (dtt, J = 12.0, 3.9, 3.9 Hz, 1 H), 1.34 (qt, J = 12.1 Hz, 3.5 Hz, 2H), 1.27 (tt, J = 7.9 Hz, 4.4 Hz, 1H), 1.08-1.18 (m, 3H), 0.93 (dtd, J = 4.4 Hz, 3.9 Hz, 3.1 Hz, 2H), 0.69 (dtd, J = 7.9 Hz, 3.9 Hz, 3.1 Hz, 2H).

^{13}C NMR (176 MHz, $CDCl_3$): δ (ppm) = 172.6, 48.4, 33.5, 25.7, 25.1, 15.0, 7.1

HRMS : $[C_{10}H_{17}ON + H]^+$: Expected = 168.1383, found 168.1388

Melting point: 137–138 °C

_N_-(Adamantan-1-yl)-2-phenylacetamide (26)

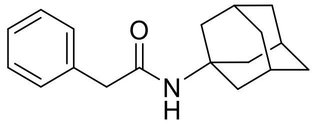

Synthesised according to the general procedure. Purified by flash column chromatography (EtOAc 10% → 50% in cyclohexane) Yield = 67 mg white solid, 5%. Data is in accordance with previous literature values.[21]

Raw NMR data: https://doi.org/10.14469/hpc/14736

^{1}H NMR (700 MHz, $CDCl_3$) δ (ppm) = 7.34 (t, *J* = 7.4 Hz, 1H), 7.27 (t, *J* = 7.4 Hz, 2H), 7.24 (d, *J* = 7.5 Hz, 2H), 5.02 (s, 1H), 3.47 (s, 2H), 2.00 – 2.05 (m, 3H), 1.88 – 1.92 (m, 6H), 1.64 (t, *J* = 3.0 Hz, 6H).

^{13}C NMR (176 MHz, $CDCl_3$) δ (ppm) = 170.2, 135.7, 129.4, 129.0, 127.2, 52.0, 45.2, 41.6, 36.4, 29.5.

HRMS: $[C_{18}H_{23}ON + H]^+$: Expected = 270.1852, found 270.1855

(S)-*N*-(1-phenylethyl)pivalamide (27)

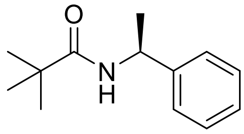

Synthesised according to the general procedure. Purified by flash column chromatography (EtOAc 15 % → 60 % in cyclohexane). Yield = 763 mg white solid, 74%. Analytical data is in accordance with literature reports.[22]

Raw NMR data: https://doi.org/10.14469/hpc/14737

^{1}H NMR (700 MHz, $CDCl_3$) δ (ppm) = 7.33 (t, *J* = 7.6 Hz, 2H), 7.29 (d, *J* = 7.4 Hz, 2H), 7.25 (t, *J* = 7.2 Hz, 1H), 5.82 (s, 1H), 5.10 (qn, *J* = 7.1 Hz, 1H), 1.48 (d, *J* = 6.9 Hz, 3H), 1.20 (s, 9H).

^{13}C NMR (176 MHz, $CDCl_3$) δ (ppm) = 177.6, 143.6, 128.8, 127.4, 126.16, 48.6, 38.7, 27.7, 21.9

HRMS $[C_{13}H_{19}NO+H]^+$: Expected 206.1539, observed 206.1535

Melting point: 118–119 °C

***tert*-Butyl-(*R*)-2-(benzylcarbamoyl)pyrrolidine-1-carboxylate (*ent*-28)**

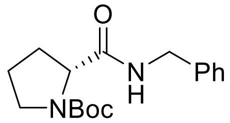

Synthesised according to the general procedure, from Boc-D-proline and benzylamine, reaction for 24 hours. Purified by flash column chromatography (EtOAc in cyclohexane, 0 → 30%). Yield = 1.19 g, white solid, 78%. Data is in accordance with previous reports.[23]

Enantiopurity >99:1 measured by HPLC Chiralpak AD-H column, 10% IPA in hexane, 1 ml/min, 40 mbar, 30 °C. Retention time = 6.6 minutes

Raw NMR data: https://doi.org/10.14469/hpc/14738

^{1}H NMR (600 MHz, DMSO) δ (ppm) = 8.40 (t, *J* = 5.9 Hz, NH, 0.6H, major), 8.36 (t, *J* = 5.8 Hz, NH, 0.4H, minor), 7.33 – 7.19 (m, 5H), 4.37 – 4.30 (m, 1H), 4.24 – 4.16 (m, 1H), 4.12 (dd, *J* = 8.6, 2.8 Hz, 0.4H, minor), 4.07 (dd, *J* = 8.5, 3.1 Hz, 1H, 0.6H, major), 3.44 – 3.37 (m, 1H), 3.32 – 3.25 (m, 1H), 2.17 – 2.03 (m, 1H), 1.89 – 1.71 (m, 3H), 1.41 (s, 3.3H, minor), 1.28 (s, 5.7H, major).

^{13}C NMR (151 MHz, DMSO) δ (ppm) = 172.5, 172.3, 153.7, 153.4, 139.7, 139.6, 128.2, 127.3, 126.9, 126.8, 126.6, 78.6, 78.5, 59.9, 59.8, 46.7, 46.5, 42.0, 41.8, 31.1, 30.1, 28.2, 27.9, 24.0, 23.2.

HRMS $[C_{17}H_{24}N_2O_3+H]^+$: Expected 305.1859, observed 305.1853.

ν_{max} (solid/cm^{-1}): 3331, 3052, 2977, 2911, 2872, 1738, 1682, 1527, 1478, 1453, 1423.

Melting point: 131–133 °C

***tert*-Butyl (S)-2-(benzylcarbamoyl)pyrrolidine-1-carboxylate (28)**

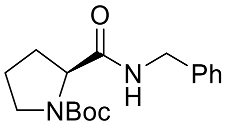

Synthesised according to the general procedure, from Boc-L -proline and benzylamine, reaction for 17 hours. Purified by flash column chromatography (EtOAc in cyclohexane, 25 → 100%). Yield = 1.37 g, white solid, 90%. NMR data identical to R enantiomer.

Enantiopurity >99:1 measured by HPLC Chiralpak AD-H column, 10% IPA in hexane, 1 ml/min 40 mbar, 30 °C. Retention time = 10.7 minutes

Melting point: >250 °C

***tert*-Butyl (S)-2-(naphthalen-1-ylcarbamoyl)pyrrolidine-1-carboxylate (29)**

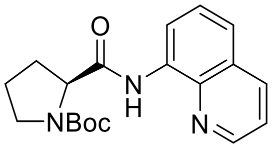

Synthesised according to the general procedure from Boc-L-proline and 8-amino quinoline, reaction time 44 hours. Purified by flash column chromatography (EtOAc in hexane 15→ 35 %) Yield = 351 mg, white solid, 21%. Data is in accordance with previous reports.[24]

Raw NMR data: https://doi.org/10.14469/hpc/14739

^{1}H NMR (700 MHz, $CDCl_3$) δ (ppm) = 10.61 (s, 0.4 H minor), 10.36 (s, 0.6 H major), 8.83 – 8.75 (m, 2H), 8.18 – 8.11 (m, 1H), 7.57 – 7.47 (m, 2H), 7.47 – 7.40 (m, 1H), 4.61 (s, 0.4H), 4.44 (s, 0.6H), 3.74-3.67 (m, 0.6H), 3.67-3.58 (m, 1H), 3.52 – 3.34 (m, 0.4H), 2.46 – 2.37 (m, 0.4H), 2.36 – 2.24 (m, 1.2H), 2.20-2.10 (m, 0.4H) 2.05-1.97 (m, 1H), 1.97-1.91 (m, 1H), 1.55 (s, 3.6H), 1.35 (s, 5.4H).

^{13}C NMR (176 MHz, $CDCl_3$) δ (ppm) = 171.7, 171.2, 155.5, 154.8, 148.6, 148.3, 138.9, 138.7, 136.4, 134.6, 134.2, 128.1, 127.4, 121.9, 121.8, 121.6, 116.8, 116.6, 80.8, 80.4, 62.6, 61.7, 47.4, 47.1, 31.5, 29.6, 28.6, 28.4, 24.6, 24.0.

ν_{max} (solid/cm^{-1}): 3285, 2973, 2928, 2870, 1702, 1673, 1524, 1484, 1414.

HRMS $[C_{19}H_{23}O_3N_3 + H]^+$: Expect 342.1812 Observe 342.1804

Melting point: 147–148 °C

Enantiopurity >99:1 measured by HPLC Chiralpak AD-H column, 20% IPA in hexane, 1 ml/min, 40 mbar, 30 °C. Retention time = 12.6 minutes

tert-Butyl-(R)-2-(quinolin-8-ylcarbamoyl)pyrrolidine-1-carboxylate (ent-29)

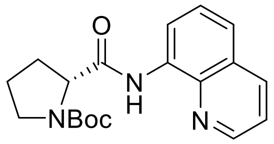

Synthesised according to the general procedure, from Boc-D-proline and 8-amino quinoline reaction for 40 hours. Purified by flash column chromatography (EtOAc in cyclohexane, 10 → 30%). Yield = 348 mg, white solid, 20%. Data is in accordance with previous reports[25]. NMR data is identical to S enantiomer.

Enantiopurity >99:1 measured by HPLC Chiralpak AD-H column, 20% IPA in hexane, 1 ml/min, 40 mbar, 30 °C. Retention time = 10.2 minutes

Melting point: 152–154 °C.

(S)-2-amino-N-benzyl-3-methylbutanamide (30)

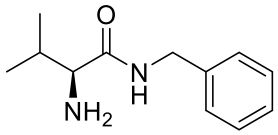

Prepared according to *in-situ* generation procedure B (see below), using 15% $BH_3.SMe_2$ and 30% (3,4,5)-trifluorophenol. Yield = 620 mg, white solid, 60%. Data is in accordance with previous reports[1]. Enantiomeric ratio was determined to be 90:10 by reaction with Marfey's reagent, Page 97).

Raw NMR data: https://doi.org/10.14469/hpc/14948

^{1}H NMR (700 MHz, $CDCl_3$) δ (ppm) = 7.66 (s, 1H), 7.32 (t, *J* = 7.5 Hz, 2H), 7.29 – 7.25 (m, 3H), 4.45 (qd, *J* = 14.9, 6.0 Hz, 2H), 3.31 (d, *J* = 3.8 Hz, 1 H) 2.35 (qqd, *J* = 7.0, 7.0, 3.8 Hz, 1H), 0.99 (d, *J* = 7.0 Hz, 3H), 0.84 (d, *J* = 7.0 Hz, 3H).

^{13}C NMR (176 MHz, $CDCl_3$) δ (ppm) = 174.2, 138.7, 128.8, 127.9, 127.5, 60.2, 43.3, 30.9, 19.9, 16.2.

Data in accordance with the literature.[1]

***N*-(Pyridin-2-yl)-2-(thiophen-3-yl)acetamide (31)**

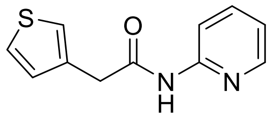

Synthesised according to the general procedure. Purified by flash column chromatography. Yield = 866 mg, off white solid, 79%. Data is in accordance with previous reports.[26]

Raw NMR data: https://doi.org/10.14469/hpc/14740

^{1}H NMR (700 MHz, $CDCl_3$) δ (ppm) = 8.27 (s, 1H), 8.22 (m, 2H), 7.69 (ddd, *J* = 8.4 ,7.5, 1.9 Hz, 1H), 7.36 (dd, *J* = 4.9, 3.0 Hz, 1H), 7.20 (m, 1H), 7.06 (dd, *J* = 4.9, 1.3 Hz, 1H), 7.02 (ddd, *J* = 7.4, 4.9, 0.9 Hz, 1H), 3.78 (s, 2H).

^{13}C NMR (176 MHz, $CDCl_3$) δ (ppm) = 169.3, 151.4, 147.9, 138.6, 133.9, 128.5, 127.2, 124.0, 120.1, 114.2, 39.4.

HRMS $[C_{11}H_{10}N_2OS+H]^+$; expected : 219.0587, observed : 219.0585

Melting point: 108–109 °C

***tert*-Butyl 4-(pyridin-2-ylcarbamoyl)piperidine-1-carboxylate (32)**

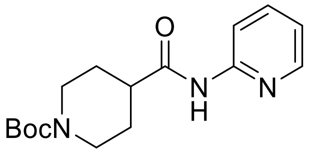

Synthesised according to the general procedure, reaction for 41 hours. Purified by flash column chromatography (EtOAc in cyclohexane, 15 → 85%). Yield = 1.20 g, white solid, 79%.

Raw NMR data: https://doi.org/10.14469/hpc/14741

^1H NMR (700 MHz, $CDCl_3$) δ (ppm) = 8.72 (s, 1H), 8.24 (dd, *J* = 4.9, 1.0 Hz, 1H), 8.20 (d, *J* = 8.3 Hz, 1H), 7.69 (ddd, *J* = 8.3, 7.3, 1.9 Hz, 1H), 7.03 (ddd, *J* = 7.3, 4.9, 0.8 Hz, 1H), 4.14 (br s, 2H), 2.72 (br s, 2H), 2.38 (m, 1H), 1.84 (br d, *J* = 11.4 Hz, 2H), 1.72 (qd, *J* = 12.3, 4.1 Hz, 2H), 1.44 (s, 9H).

^{13}C NMR (176 MHz, $CDCl_3$) δ (ppm) = 173.4, 154.8, 151.7, 147.7, 138.7, 120.0, 114.6, 79.8, 44.3, 43.6, 42.9, 28.6.

HRMS $[C_{17}H_{23}N_3O_3+H]^+$: Calculated 306.1812 Observed 306.1809

ν_{max} (solid/cm^{-1}): 3312, 2978, 2947, 2925, 2847, 1672, 1578, 1528, 1458, 1420.

Melting point: 156–157 °C

***N*-(Pyridin-2-yl)pivalamide (33)**

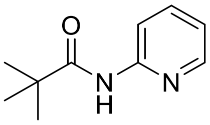

Synthesised according to the general procedure, reaction for 45 hours. Purified by flash column chromatography, (EtOAc in cyclohexane, 10 → 50 %). Yield = 535 mg, white solid, 60 %.

Raw NMR data: https://doi.org/10.14469/hpc/14742

^1H NMR (700 MHz, $CDCl_3$) δ (ppm) = 8.26 – 8.24 (m, 1H), 8.24 – 8.22 (m, 1H), 8.02 (s, 1H), 7.69 (ddd, J = 8.3, 7.3, 1.9 Hz, 1H), 7.02 (ddd, J = 7.3, 4.9, 0.9 Hz, 1H), 1.32 (s, 9H).

^{13}C NMR (176 MHz, $CDCl_3$) δ (ppm) = 177.2, 151.7, 147.8, 138.5 119.8, 114.0, 39.9, 27.6.

HRMS $[C_{10}H_{14}N_2O + H]^+$: Expected 179.1179, observed 179.1181

Melting point: 71–72 °C

***N*-(Pyridin-2-yl)benzamide (34)**

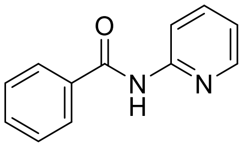

Synthesised according to the general procedure, reaction for 44 hours. Purified by flash column chromatography (EtOAc in hexanes, 10% → 70%). Yield = 402 mg, crystalline white solid, 40%. Analytical data is in accordance with literature reports.[27]

Raw NMR data: https://doi.org/10.14469/hpc/14743

^{1}H NMR (700 MHz, $CDCl_3$) δ (ppm) = 8.91 (s, 1H), 8.40 (dt, J = 8.4 Hz, 1 Hz, 1H), 8.20 (dd, J = 5 Hz, 1 Hz), 1 H), 7.91-7.93 (m, 2H), 7.75 (ddd, J = 8.4, 7.4, 1.9 Hz, 1H), 7.56 (tt, J = 7.4, 1.2 Hz, 1H), 7.48 (t, 7.8 Hz, 2H), 7.04 (ddd, J = 7.3, 4.9, 1 Hz, 1H).

^{13}C NMR (176 MHz, $CDCl_3$) δ (ppm) = δ 166.1, 151.8, 147.9, 138.7, 134.5, 132.4, 128.9, 127.5, 120.0, 114.5.

HRMS $[C_{12}H_{10}NO_2+H]^+$: Calculated = 199.0866, found 199.0865

Melting point: 78–82 °C

***N*-(Pyridin-2-yl)picolinamide (35)**

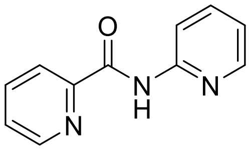

Synthesised according to the general procedure, reaction time 42 hrs. Purified by flash column chromatography (EtOAc in hexanes, 10% → 100%). Yield = 200 mg, white solid, 20%. Analytical data is in accordance with literature reports.[28]

Raw NMR data: https://doi.org/10.14469/hpc/14744

^{1}H NMR (700 MHz, $CDCl_3$) δ (ppm) = 10.55 (s, 1H), 8.64 (ddd, *J* = 4.7, 1.6, 0.9 Hz, 1H), 8.42 (dt, *J* = 8.3, 0.8 Hz, 1H), 8.37 (ddd, *J* = 4.9, 1.9, 0.9 Hz, 1H), 8.30 (dt, *J* = 7.8, 1 Hz, 1H), 7.91 (td, *J* = 7.7, 1.7 Hz, 1H), 7.76 (m, 1H), 7.49 (ddd, *J* = 7.5, 4.7, 1.2 Hz, 1H), 7.08 (ddd, *J* = 7.3, 4.9, 1.0 Hz, 1H).

^{13}C NMR (176 MHz, $CDCl_3$) δ (ppm) = 162.8, 151.3, 149.5, 148.4, 148.4, 138.4, 137.7, 126.9, 122.6, 120.0, 114.1.

HRMS $[C_{11}H_9N_3O+H]^+$: Expected 200.0818, observed 200.0817

Melting point : 101–104 °C

N-(Pyridin-2-yl)-1H-indazole-3-carboxamide (36)

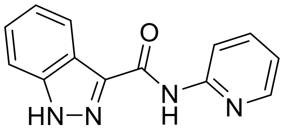

Synthesised according to the general procedure, reaction for 72 hours. Purified by flash column chromatography (MeOH in DCM, 0 → 5%), and further recrystallised from hot methanol. Yield = 238 mg, pale yellow/orange crystalline solid, 1 mmol, 20%.

Raw NMR data: https://doi.org/10.14469/hpc/14745

^{1}H NMR (700 MHz, $CDCl_3$) δ (ppm) = 13.92 (s, 1H), 9.77 (s, 1H), 8.37 (ddd, *J* = 4.8 Hz, 1.7 Hz, 0.7 Hz, 1H), 8.26 (d, *J* = 8.3 Hz, 1H), 8.22 (d, *J* = 8.1 Hz, 1H), 7.87 (ddd, *J* = 8.3 Hz, 7.4 Hz, 1.7 Hz, 1 H), 7.67 (d, 8.4 Hz, 1H), 7.47 (ddd, *J* = 8.4 Hz, 6.9 Hz, 0.9 Hz, 1 H), 7.32 (t, *J* = 7.4 Hz, 1H), 7.17 (ddd, *J* = 7.4 Hz, 4.8 Hz, 0.8 Hz, 1H).

^{13}C NMR (176 MHz, $CDCl_3$) δ (ppm) = 160.6, 151.1, 148.4, 141.5, 138.5, 137.3, 127.0, 122.9, 121.5, 121.2, 119.9, 113.5, 111.2.

HRMS $[C_{13}H_{10}N_4O+H]^+$: Expect 239.0927 observe 239.0921

ν_{max} (solid/cm^{-1}): 3336, 2726, 1678, 1595, 1574, 1538, 1512, 1488, 1454, 1433

Melting point > 250 °C

In-Situ generation of catalyst

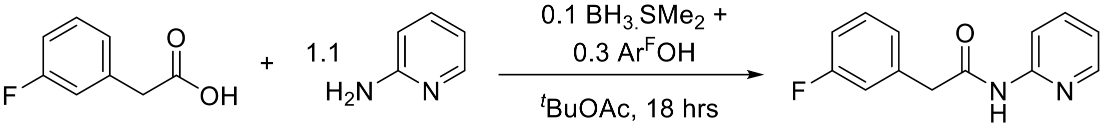

4, 81%

Procedure A: Under an atmosphere of argon, 3,4,5-triflurophenol (222 mg, 1.5 mmol) was dissolved in 0.6 ml anhydrous diethyl ether and cooled to 0 °C. Borane dimethylsulfide (50 µl, 0.5 mmol) was added and the reaction stirred at 0 °C for 5 minutes before heating to reflux for 90 minutes. After this time, 3-fluorophenylacetic acid (771 mg, 5 mmol) was added (hydrogen evolution observed), followed by 2-amino pyridine (518 mg, 5.5 mmol) and 5 ml *tert*-butyl acetate. The reaction was heated to reflux under a Dean-Stark distillation apparatus for 18 hours when the reaction was cooled and quenched by addition of water. The aqueous layer was basified with 15 ml 1 M NaOH, extracted with DCM (3 * 15 ml), dried over Na_2SO_4, filtered and concentrated under reduced pressure. The pure product was isolated by flash column chromatography (EtOAc in cyclohexane, 15% → 40%), to give 929 mg of a white solid 4.03 mmol, 81%.

A similar procedure was followed for reactions using 2 equivalents of trifluorophenol:

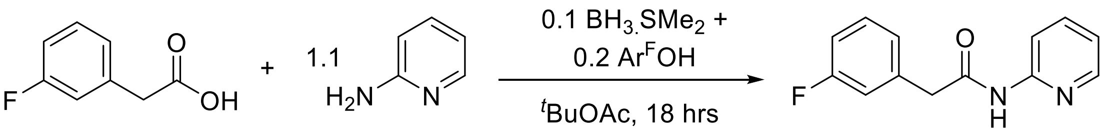

4, 80%

Purified by column chromatography, yield = 80%

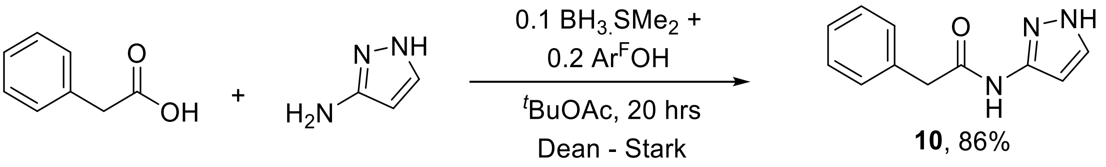

10, 86%

Purified by recrystallisation from MeOH/water (5:4), 861 mg, 86%

Procedure B: The reactions were carried out as for procedure A, however 3,4,5-trifluorophenol and borane dimethylsulfide were stirred in 5 ml ^{t}BuOAc at room temperature, and the carboxylic acid and amine were added directly to this mixture, before heating to reflux under a Dean-Stark trap.

2-Phenyl-*N*-(quinolin-8-yl)acetamide (6)

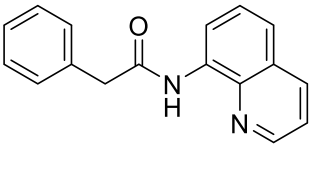

Synthesised according to the *in situ* catalyst generation procedure B, using 0.2 eq. of 3,4,5-trifluorophenol and stirring at rt for 2 h before adding the acid and amine. Yield = 996 mg, white solid, 76%. Analytical data is in accordance with compound **6** synthesised using general procedure.

N-(3-Bromophenyl)-1H-pyrazole-3-carboxamide (12)

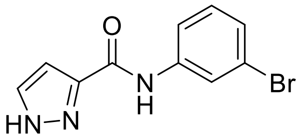

Synthesised according to the *in situ* catalyst generation procedure B, using 0.2 eq. of 3,4,5-trifluorophenol and stirring at rt for 2 h before adding the acid and amine. Yield = 891 mg, white solid, 67%. Analytical data is in accordance with compound **12** synthesised using general procedure.

Varying the ratio of phenol to borane

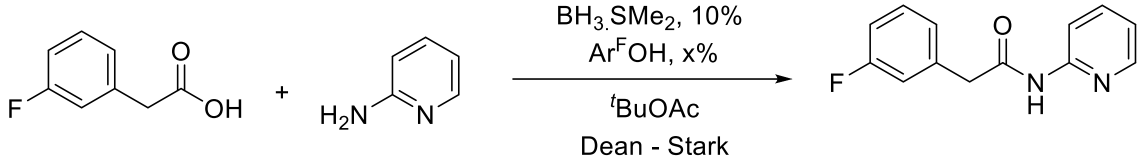

Reactions were carried out according to the *in-situ* generation procedure, using varying amounts of trifluorophenol. For the reaction with no added phenol, the acid and amine were mixed in 5 ml *tert*-butyl acetate, heated to reflux and borane dimethylsulfide was added under a flow of argon, before the reaction was heated in a Dean-Stark apparatus.

Phenol Loading, x%	Yield / %
30 %	81
20 %	80
10 %	71
0 %	70
$B(OH)_3$	48

Borane dimethylsulfide catalyst control reaction

As the *in-situ* generation reaction experiments (above) showed high reactivity with borane dimethylsulfide alone, we investigated whether this would be a more general catalyst. We hypothesised that a part of the reactivity was derived from stoichiometric activation of the carboxylic acid through 3 B-H bonds on the catalyst, so tested the reaction using picolinic acid and benzylamine, which gave 96% yield with only 5% of the $B(OAr^F)_3$ catalyst, and offered a co-ordinating substrate. The reaction was carried out as described above, and provided only 6% isolated yield of amide.

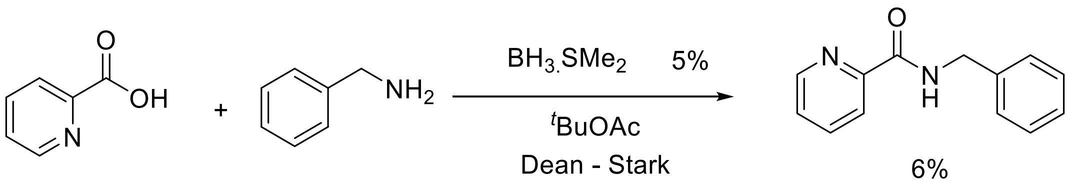

Larger scale amidation reactions

***N*-(Pyridin-2-yl)-2-(thiophen-3-yl)acetamide (31)**

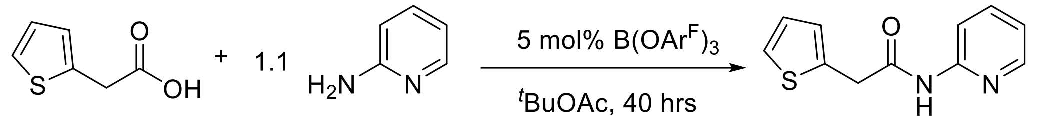

12.98 g, 59%, PMI = 10.1

3-thiopheneacetic acid (14.2g, 100 mmol) and 2-aminopyridine (10.4g, 110 mmol) were suspended in 70 ml *tert*-butyl acetate and heated to 85 °C. A solution of $B(OAr^F)_3$ (2.26g, 5 mmol, in 10 ml ^tBuOAc) was added, followed by a further 20 ml *tert*-butyl acetate totalling 100 ml reaction solvent. The suspension did not fully dissolve and was heated to reflux under a Dean-Stark apparatus for 40 hours then filtered whilst hot and washed with 10 ml hot ^tBuOAc. A precipitate formed in the collection flask and was redissolved by heating and addition of 1 ml EtOH, then cooled slowly, resulting in a crop of 10.28 g large brown crystals. The mother liquor was concentrated to approx. 30 ml, the resulting precipitate was warmed back into solution and on cooling produced a second crop of crystals, which were collected and washed with 10 ml cold ^tBuOAc giving 2.70 g for a total yield of 12.98 g (59.5 mmol, 60%).

PMI = total mass of substances used (reagents, solvents)/mass of product

PMI = (14.2 + 10.4 + 2.26 + ((100 + 10 + 10) * 0.866)) / 12.98

PMI = 10.1

2-(3-Fluorophenyl)-N-(pyridin-2-yl)acetamide(4)

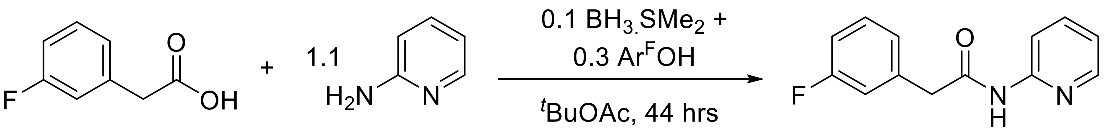

16.2 g, 71%, PMI = 11.8

Under an atmosphere of argon, 3,4,5-trifluorophenol (4.44 g, 30 mmol) was dissolved in anhydrous diethyl ether (10 ml) and cooled to 0 °C. Borane dimethylsulfide (950 µl, 10 mmol) was added dropwise (evolution of hydrogen observed), and the solution stirred at this temperature for 20 minutes before heating to reflux for 2 hours. After this time the ether was removed by distillation and the product redissolved in 10 ml *tert*-butyl acetate. In a separate flask (taking no precautions to exclude air) 3-fluorophenylacetic acid (15.4 g, 100 mmol) and 2-amino pyridine (10.4 g, 110 mmol) were suspended in 80 ml *tert*-butyl acetate and heated to reflux under a Dean-Stark condenser. The catalyst solution was added, followed by a further 10 ml *tert*-butyl acetate. The reaction remained heterogenous throughout. After 44 hours, the reaction was made homogenous by the addition of 15 ml EtOH (whilst still hot). On cooling a crop of colourless crystals formed, these were collected and washed twice with ^tBuOAc (10 ml then 5 ml) to yield 8.28 g. The mother liquor produced two further batches of crystals, each washed with 10 ml tBuOAc (2.7g). The mother liquor was concentrated and 2 ml EtOH was added to aid dissolution. The recrystallisation and washing cycle was repeated for a total of 6 crops of crystals, each washed with 10 ml tBuOAc, excluding the final two batches which were each washed with 10 ml diethyl ether. Final yield = 16.25g, 70.6 mmol, 71%.

PMI = (15.4+10.4+0.76+4.4+((10+10+10)*0.706) + ((100+10+5+10+10+10)*0.866)+ (17*0.789)) /16.25
PMI = 11.8

Inhibition with 2-amino pyridine

In order to explore the reactivity difference between boronic acid and borate catalysts for reactions involving 2-amino pyridine, we tested it as an additive in reactions between benzoic acid and benzylamine.

In a 50 ml round bottomed flask, benzoic acid (610 mg, 5 mmol) and 1,3,5-trimethoxybenzene (for use as internal standard, 84.1 mg, 0.5 mmol) were dissolved in 10 ml *tert*-Amyl methyl ether (TAME), with the addition of additives as listed in the table. Benzylamine (600 µl, 5.5 mmol) was added and the reaction mixture was heated to 70 °C. 2-chlorophenylboronic acid was added then the flask equipped with a Dean-Stark distillation apparatus with a filled side arm and the reaction was brought to reflux for 18 hours. The reaction was quenched with $NaHCO_3$ solution, extracted into DCM, washed with 1M HCl, dried over Na_2SO_4, filtered and concentrated in vacuo. The yield was determined by ^{1}H NMR analysis vs. the internal standard.

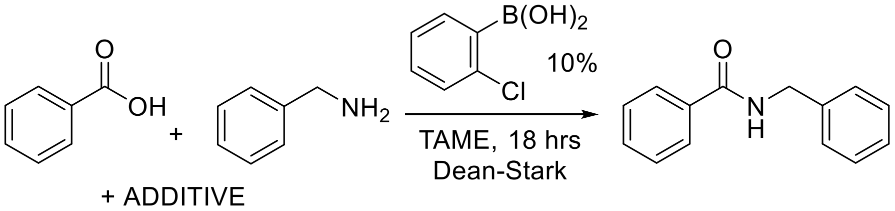

Additive	Amount (equivs.)	Yield
None		79
2-amino pyridine	0.1	67
	1	41
	2	21
	4	10
4-amino pyridine	1	70
4-DMAP	1	68
NBu_3	1	89

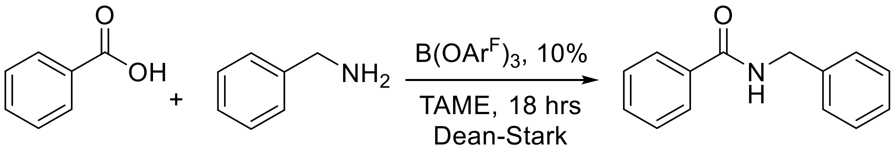

Additive	Amount (equivs.)	Yield
None		71
2-amino pyridine	1	55
	2	32
	4	23

Adduct formation between 2-amino pyridine and phenylboroxine

Triphenylboroxine (31 mg, 0.1 mmol) and 2-aminopyridine (9 mg, 0.1 mmol) were dissolved in 500 µl $CDCl_3$, and the resulting solution was analysed by ^{1}H and ^{11}B NMR spectroscopy. Crystals suitable for analysis by single crystal x-ray diffraction were grown by vapour diffusion of pentane into this solution. NMR data is available from [10.14469/hpc/14848](https://doi.org/10.14469/hpc/14848)

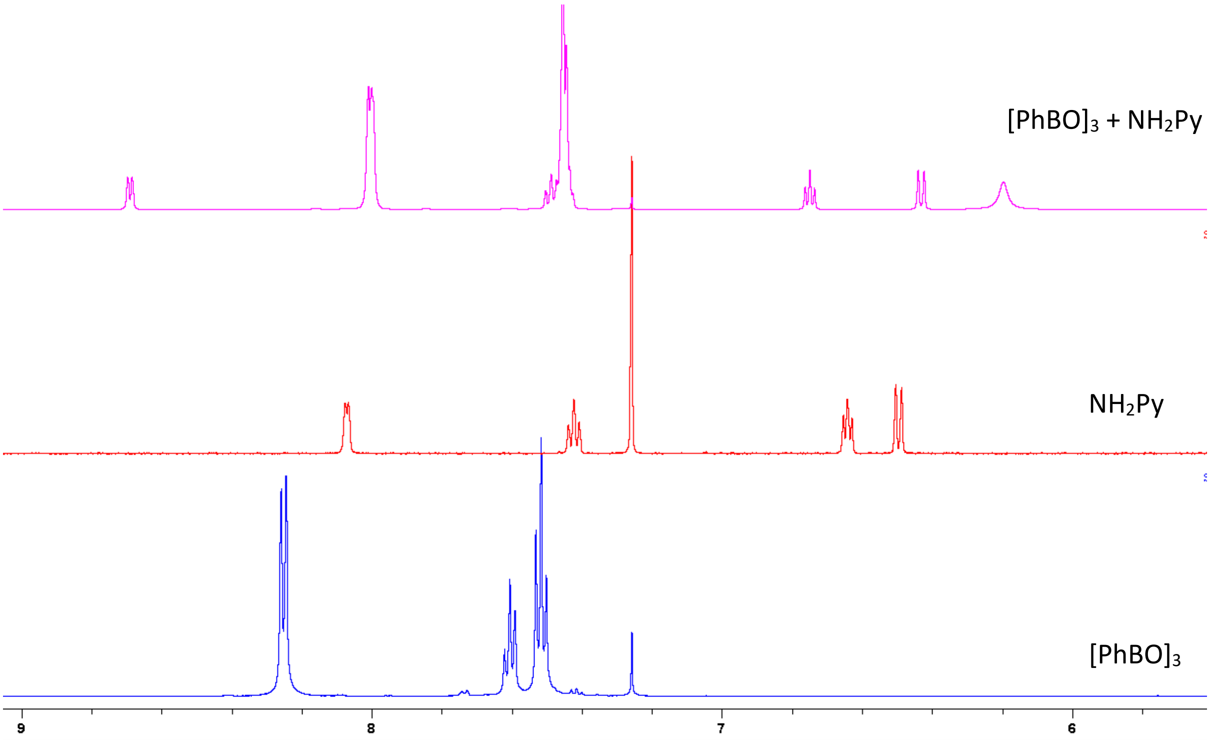

Aromatic region of the NMR spectra of phenylboroxine, 2-amino pyridine, and a 1:1 mixture, in $CDCl_3$.

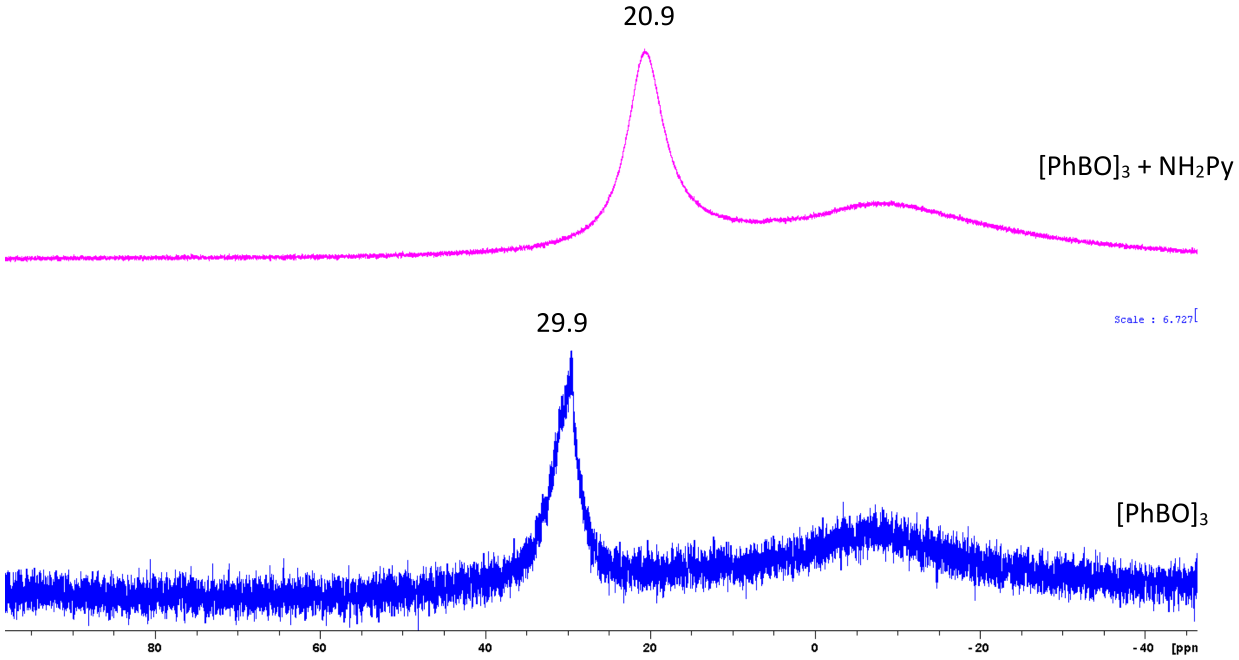

^{11}B NMR spectra of triphenylboroxine and a mixture of triphenylboroxine with 2-amino pyridine, each in $CDCl_3$

Interaction of 2-amino pyridine with 2-chlorophenylboroxine

Synthesis of 2-Chlorophenylboroxine

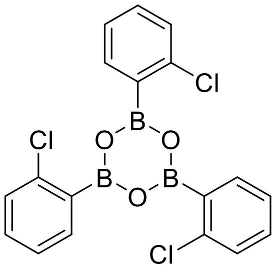

Under an atmosphere of argon, 2-chlorophenyl boronic acid (3g, 19 mmol) was suspended in toluene (60 ml) and heated to reflux under a dean-stark apparatus for 4 hours. On cooling, a white precipitate formed. The reaction mixture was filtered under air and washed twice with toluene (10 ml), redissolving most of the solid. The solution was poured into ice cold hexane (150 ml) and a white precipitate formed, the solid was collected by filtration, washed with twice with hexane (20 ml), and dried under reduced pressure. Yield = 1.17 g white needle crystals, (2.8 mmol, 44 %).

NMR data is available from 10.14469/hpc/14849

^{1}H NMR (700 MHz, $CDCl_3$) δ (ppm) = 8.26 (d, J = 7.5 Hz, 3H), 7.49 – 7.45 (m, 6H), 7.39 – 7.35 (m, 3H).

^{13}C NMR (176 MHz, $CDCl_3$) δ (ppm) = 141.5, 138.9, 133.6, 130.5, 128.9, 126.4.

^{11}B NMR (225 MHz, $CDCl_3$) δ (ppm) = 29.2.

Reaction of 2-amino pyridine with 2-chlorophenylboroxine

2-Chlorophenylboroxine (41.5 mg, 0.1 mmol) and 2-amino pyridine (9 mg, 0.1 mmol) were dissolved in 600 µl $CDCl_3$ and analysed by ^{1}H and ^{11}B NMR spectroscopy.

NMR data is available from 10.14469/hpc/14850

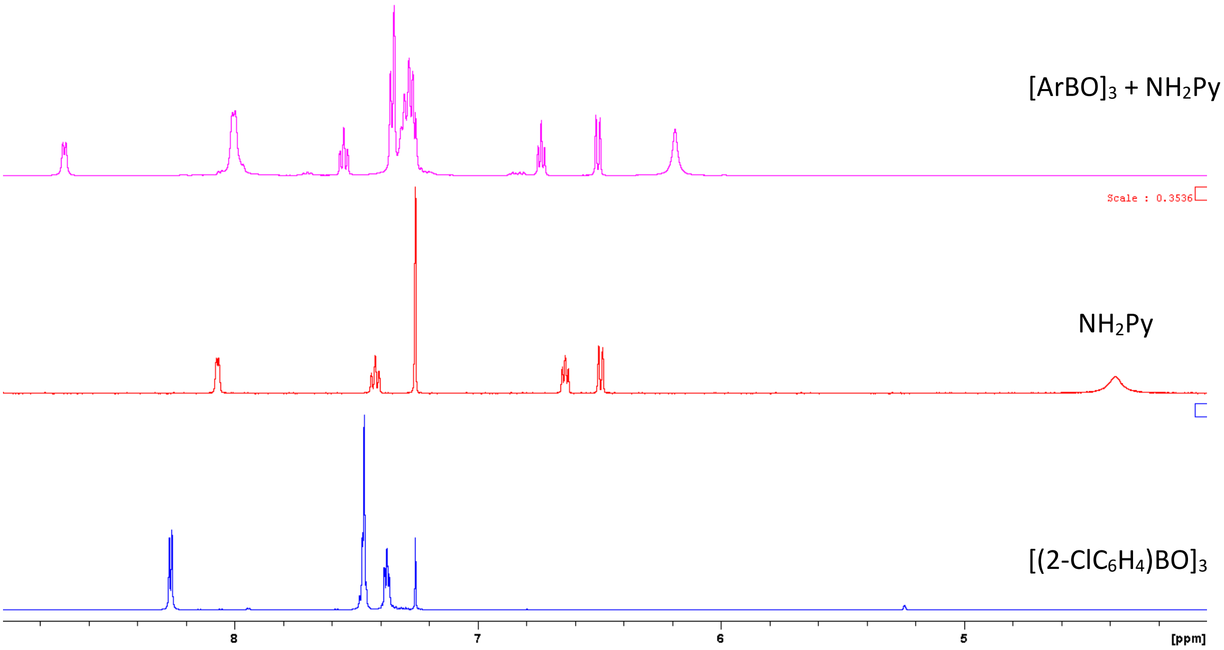

Window of the ^{1}H NMR spectrum ($CDCl_3$) of the interaction of 2-chlorophenylboroxine with 2-aminopyridine

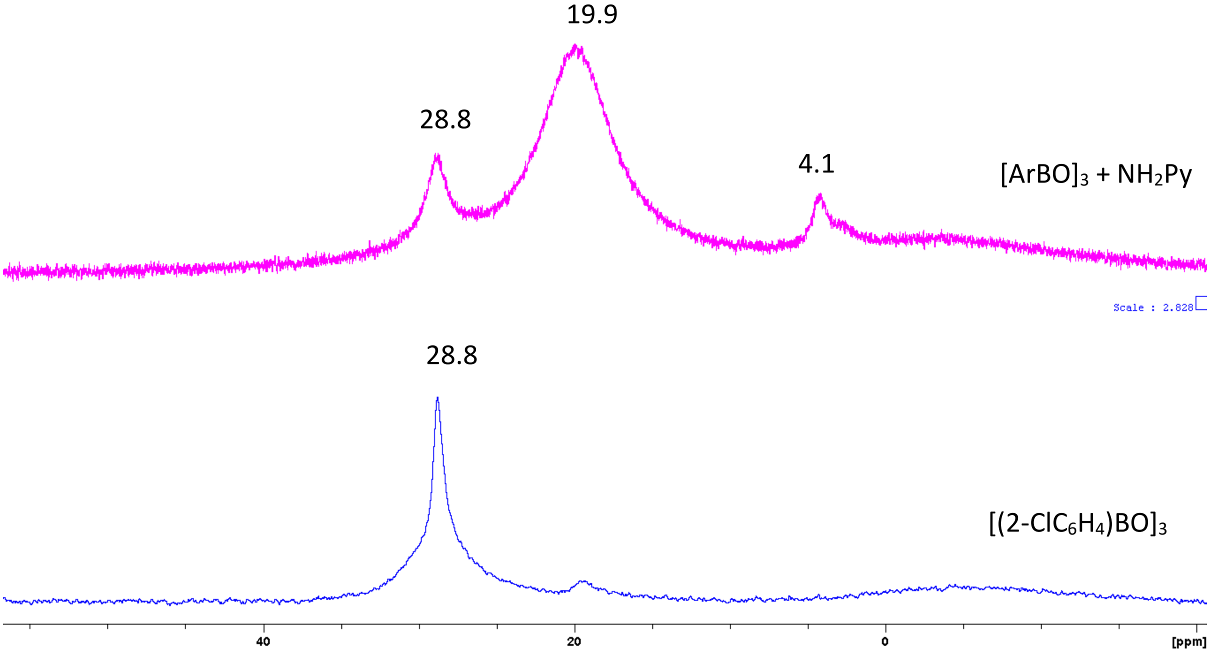

^{11}B NMR spectrum ($CDCl_3$) of the interaction between 2-chlorophenylboroxine and 2-amino pyridine

Reaction of 2-amino pyridine with 2-chlorophenylboronic acid

Under an atmosphere of argon, 2-chlorophenyl boronic acid (47 mg, 0.3 mmol, 3 eq.) and 2-aminopyridine (10 mg, 0.1 mmol, 1 eq.) were suspended in $CDCl_3$ (1.2 ml), and activated 4Å molecular sieves (1.2 g) were added. The reaction was stirred for 3 days, after which time it was filtered and ^{1}H and ^{11}B NMR spectra were measured, revealing a product identical to that obtained using the pre-formed boroxine. NMR data is available from [10.14469/hpc/14847](10.14469/hpc/14847)

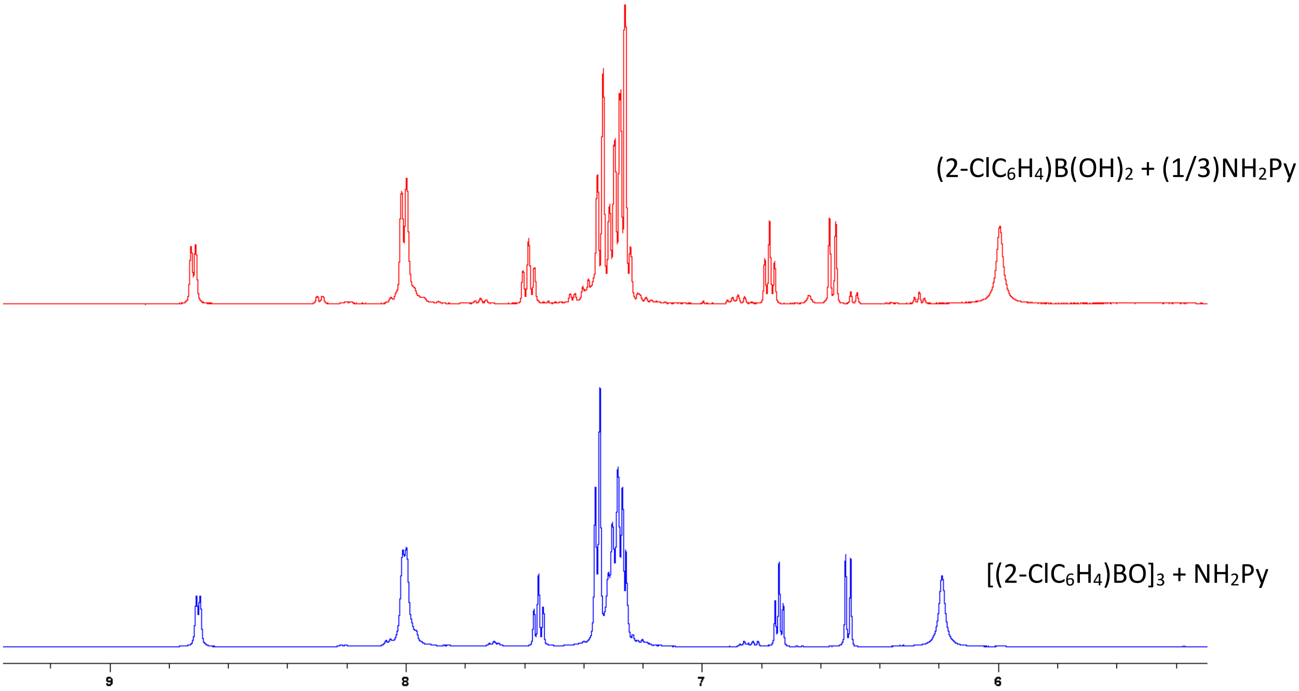

^{1}H NMR spectrum of the reaction between 2-chlorophenylboronic acid and 2-amino pyridine in the presence of 4Å molecular sieves in $CDCl_3$.

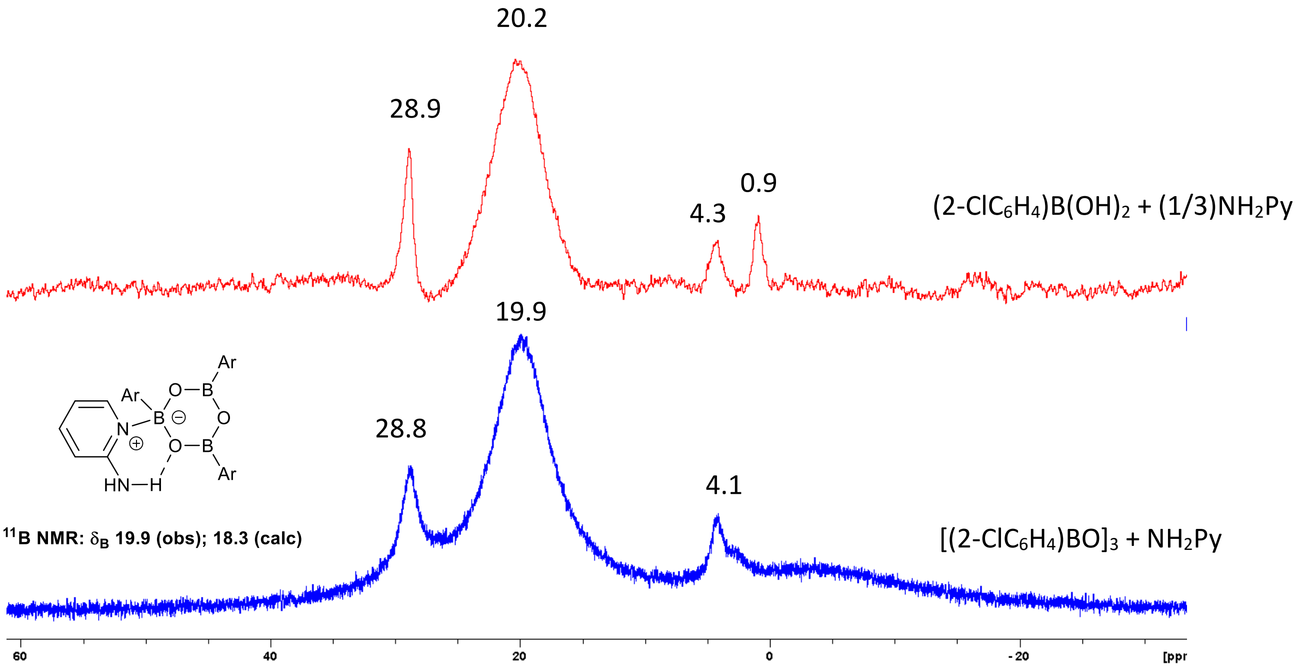

^{11}B NMR spectrum ($CDCl_3$) of (top) the reaction between 2-chlorophenylboronic acid and 2-amino pyridine, and (bottom) the interaction between 2-chlorophenylboroxine and 2-amino pyridine. The chemical shift of 19.9 ppm is consistent with a boroxine-pyridine complex in which the pyridine is in rapidly exchanging between the three boron atoms on the NMR timescale. For calculated NMR data, see: https://doi.org/10.14469/hpc/14850

Interaction of 2-aminopyridine with catalysts in ^{t}BuOAc

As the amidation is carried out in ^{t}BuOAc, NMR investigations were carried out into the interaction between 2-amino pyridine and 2-chlorophenylboronic acid, $B(OCH_2CF_3)_3$, and $B(OAr^F)_3$ in tBuOAc.

2-chlorophenylboronic acid (28 mg, 0.18 mmol) and 2-aminopyridine (17 mg, 0.18 mmol) were dissolved in 600 µl *tert*-butyl acetate and transferred to an NMR tube for NMR analysis. Analysis by ^{11}B NMR showed that the major species was unreacted $ArB(OH)_2$ (δ = 29 ppm), with formation of two further species (δ = 19 ppm, amine-boroxine adduct, and δ = 4 ppm, minor). Over 42 hours no changes were observed, after which time the reaction was heated to 70 °C for 18 hours, which led to further conversion to the amine-boroxine adduct. The signal at δ = 19 ppm was also seen in the reaction of 2-chlorophenylboroxine (83 mg, 0.2 mmol) with 2-aminopyridine (19 mg, 0.2 mmol) in the presence of activated 3 Å molecular sieves.

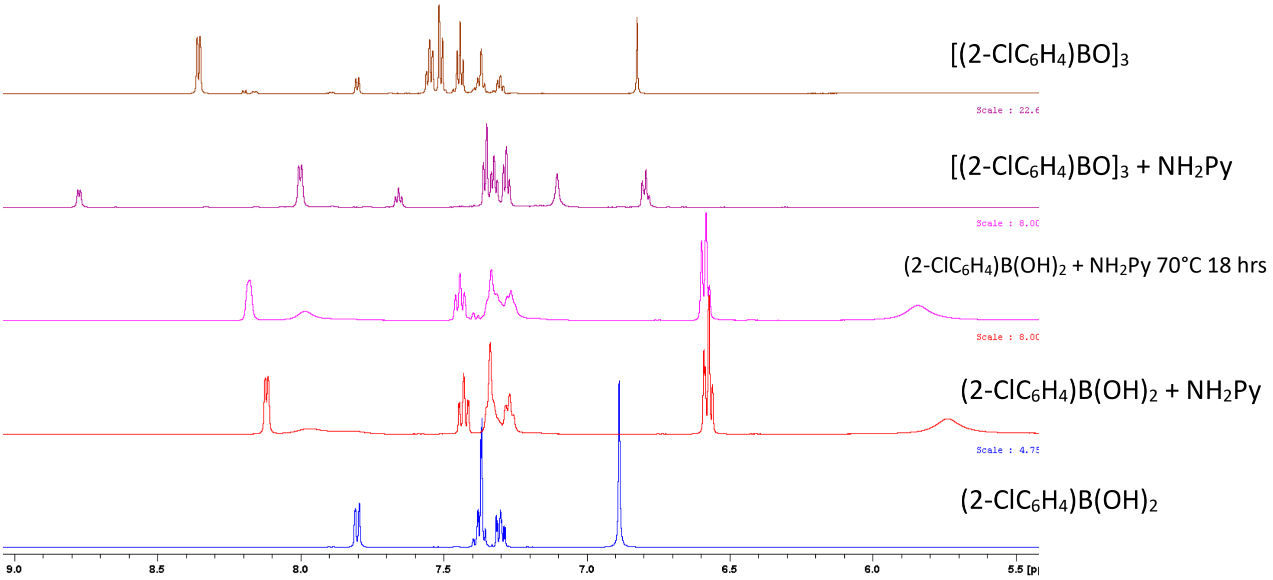

Aromatic region of the ^{1}H NMR spectra of the indicated samples in ^{t}BuOAc.

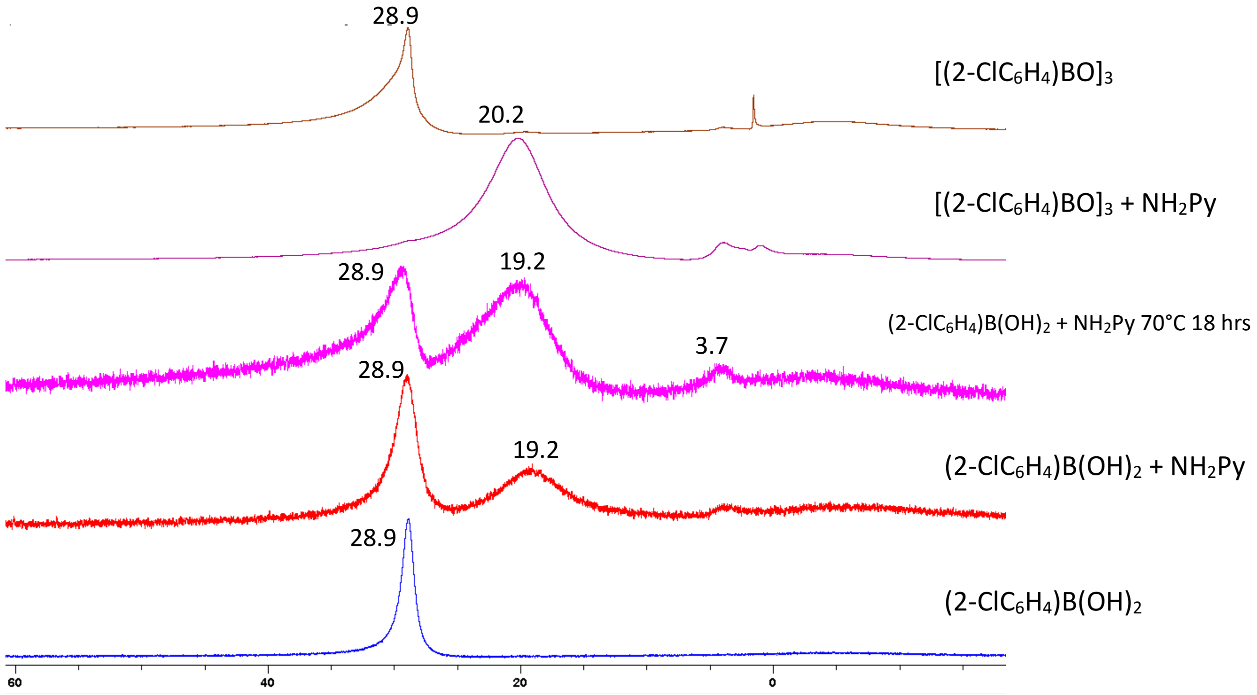

^{11}B NMR spectra of the indicated samples in ^{t}BuOAc

Tris(trifluoroethyl)borate (39 µl, 0.18 mmol) was added to two solutions of 2-amino pyridine (17 mg, 0.18 mmol, and 5.6 mg, 0.06 mmol) in 600 µl ^{t}BuOAc, and the samples analysed by NMR spectroscopy. The titration shows the formation of an adduct which is in fast equilibrium with excess borate on the NMR timescale. In a separate reaction, tris(trifluoroethyl)borate (39 µl, 0.18 mmol), trifluoroethanol (13.1 µl, 0.18 mmol) and N,N-diisopropyl-N-ethylamine (31.4 µl, 0.18 mmol) were added to 600 ul ^{t}BuOAc and analysed by NMR spectroscopy, to provide a reference for $[HNR_3]^+[B(OR)_4]^-$ salt.

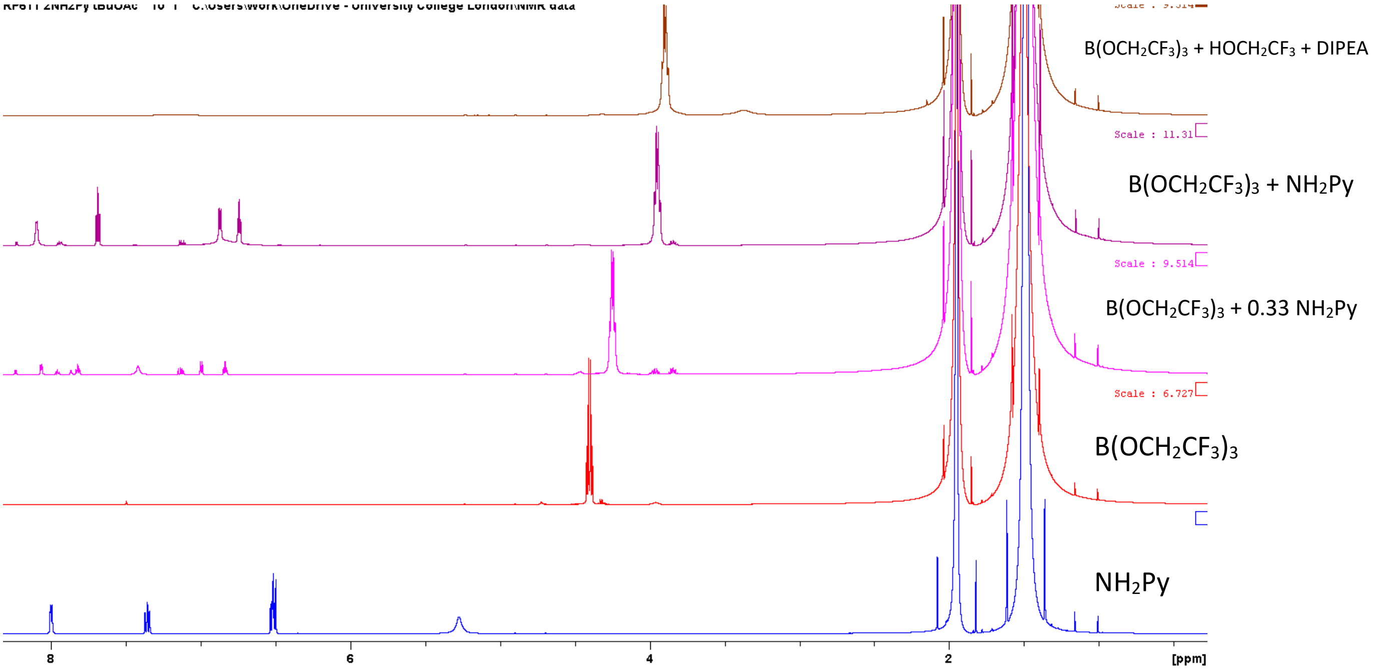

^{1}H NMR spectra of the indicated samples in tBuOAc

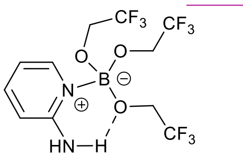

^{11}B NMR: δ_B **4.9 (obs); 3.8 (calc)**

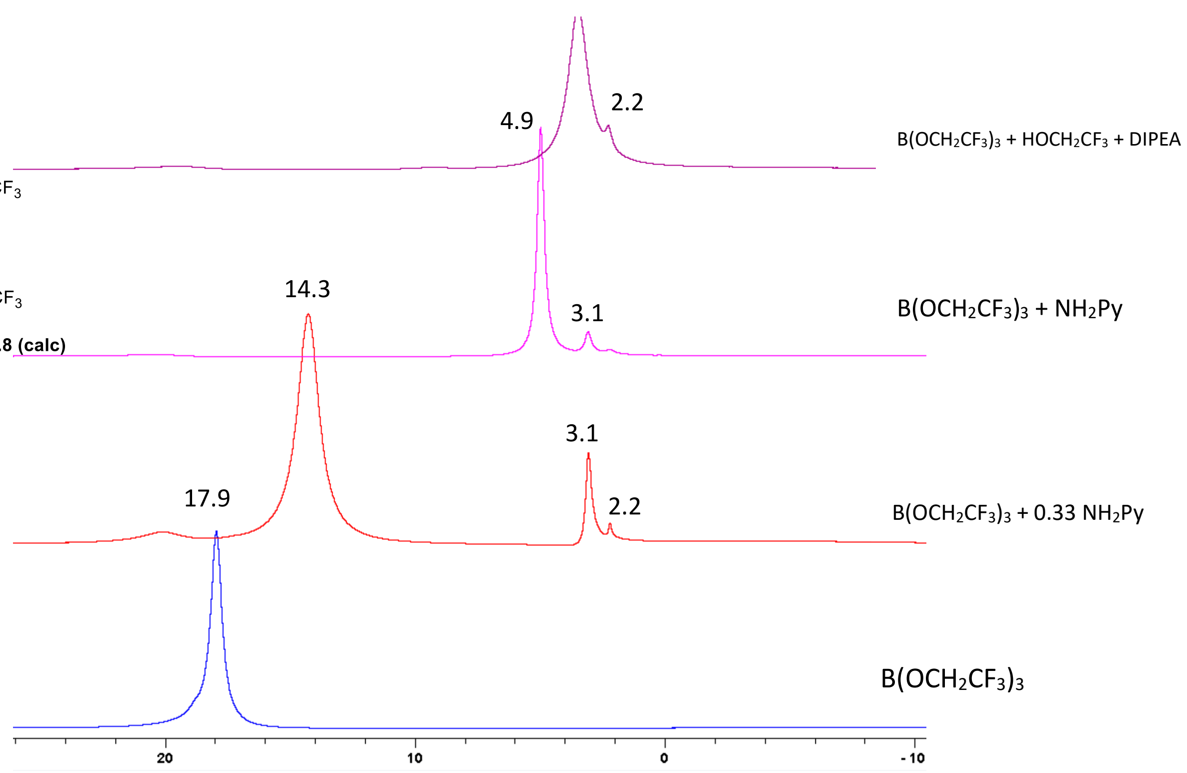

^{11}B NMR spectra of the indicated samples in ^{t}BuOAc.

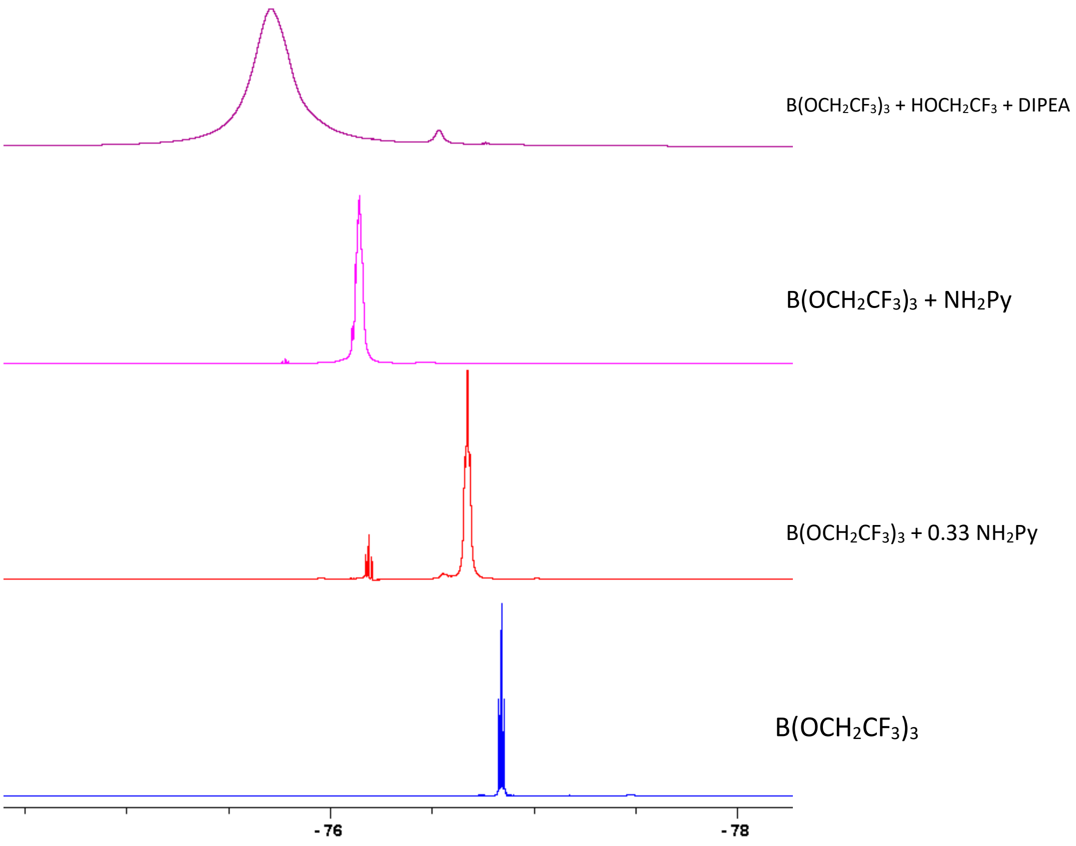

^{19}F NMR spectra of the indicated samples in ^{t}BuOAc.

Tris(3,4,5-triflurorophenyl)borate (81 mg, 0.18 mmol) and 2-aminopyridine (17 mg, 0.18 mmol) were dissolved in 600 µl ^{t}BuOAc and analysed by ^{1}H, ^{11}B, and ^{13}C NMR spectroscopy. Dissolution of $B(OAr^F)_3$ in ^{t}BuOAc leads to formation of 2 species in ^{11}B NMR – one trigonal (δ = 15.6 pm) and one tetrahedral (δ = 2.8 ppm). This is replicated when a sample of borate is dissolved in 500 µl $CDCl_3$ (one peak at δ = 15.7 ppm) and 300 µl ^{t}BuOAc is added (two peaks, δ = 15.4 ppm, and δ = 2.6 ppm). In a separate reaction, Tris(3,4,5-triflurorophenyl)borate (81 mg, 0.18 mmol), 3,4,5-trifluorophenol (27 mg, 0.18 mmol) and *N*,*N*-diisopropyl-*N*-ethylamine (31.4 µl, 0.18 mmol) were added to 600 µl ^{t}BuOAc and analysed by NMR spectroscopy, to provide a reference for $[HNR_3]^+[B(OR)_4]^-$ salt. ^{1}H and ^{11}B NMR spectra indicate that several species are formed from the interaction of 2-amino pyridine with the borate, including formation of $[B(OR)_4]^-$ salt.

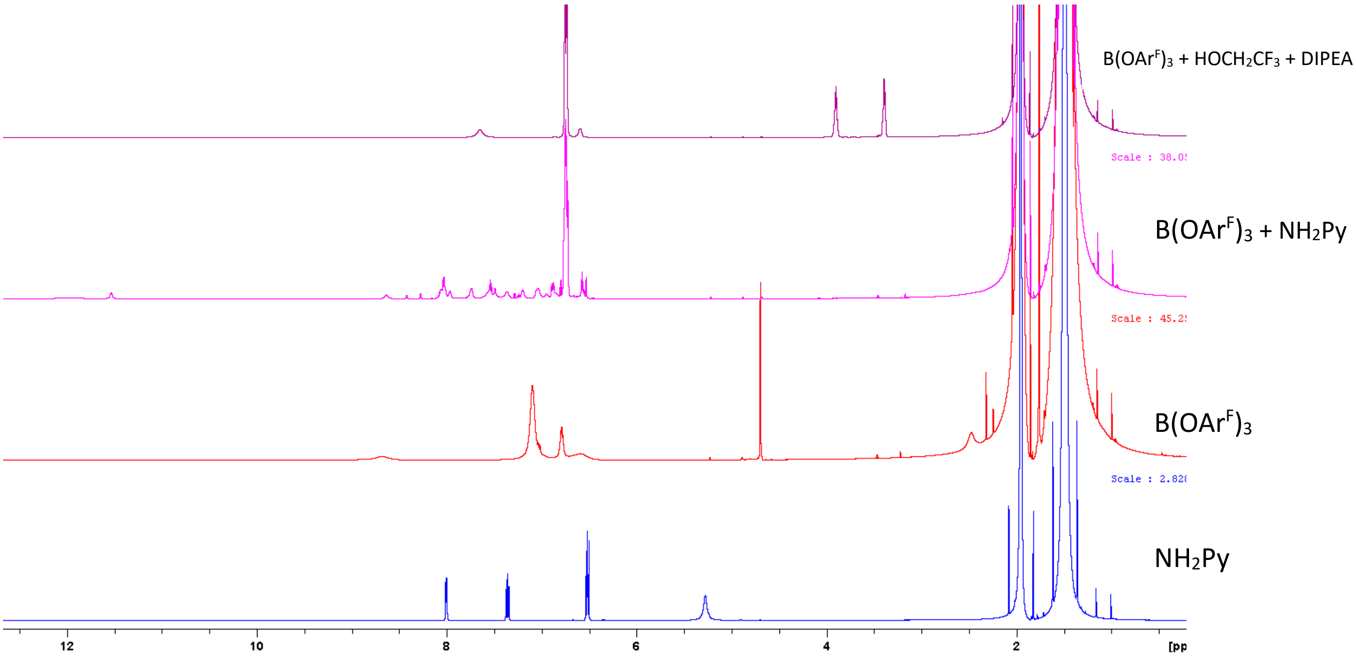

^{1}H NMR spectra of the indicated samples in ^{t}BuOAc.

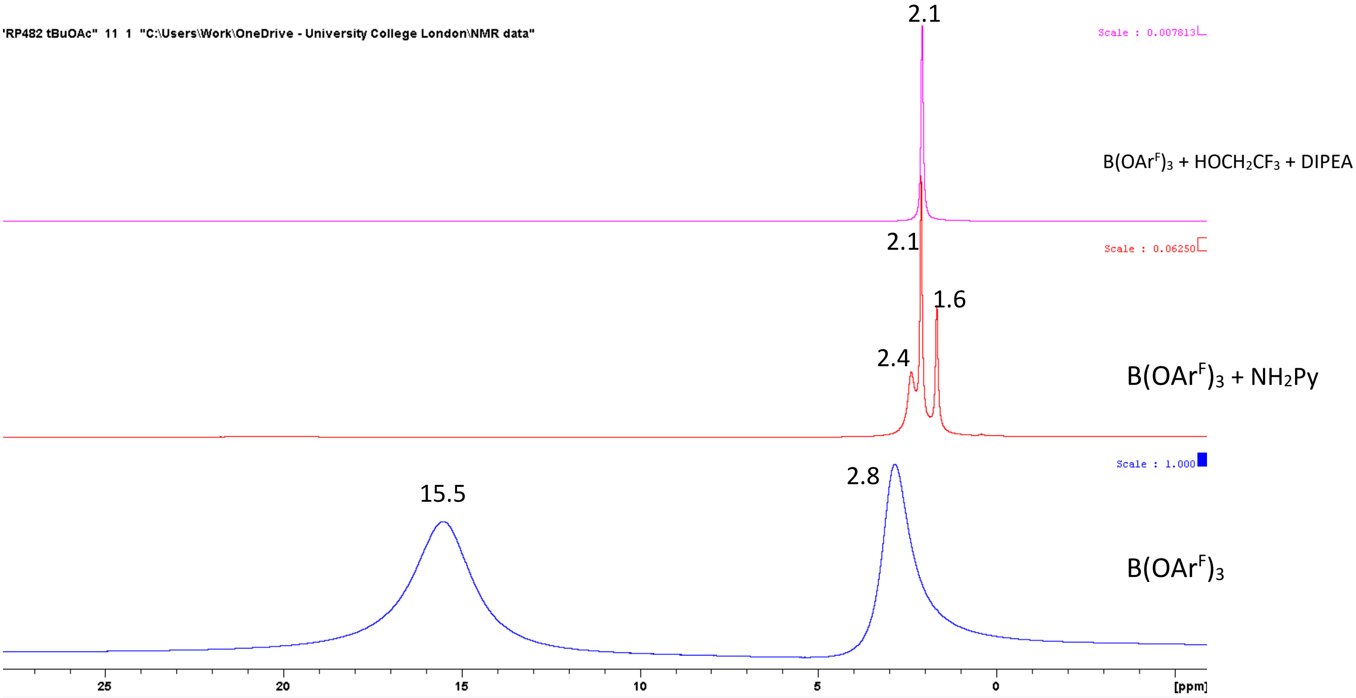

^{11}B NMR spectra of the indicated samples in ^{t}BuOAc.

While analysis is complicated by the formation of multiple species, these reactions reveal that the type of adducts formed between 2-aminopyridine and borates differ significantly to those with 2-chlorophenylboronic acid as the boroxine- amine adduct is favoured for the boronic acid whereas this is less feasible for the borates. The ^{11}B NMR shifts observed for interactions of 2-aminopyridine with both $B(OCH_2CF_3)_3$ and $B(OAr^F)_3$ are consistent with the formation of tetrahedral $R_2N\text{-}B(OR)_3$ complexes. Calculations at the B3LYP+GD3+BJ/Def2-TZVPP/SCRF level suggest the tetrahedral complex (below, left) is 6.15 kcal mol^{-1} lower in free energy (ΔG_{298}) than the borate/amine components using chloroform as a continuum solvent (https://doi.org/10.14469/hpc/14634).

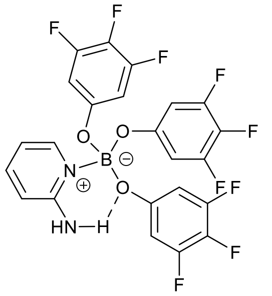

^{11}B NMR: δ_B 1.6-2.4 (obs); 3.8 (calc)

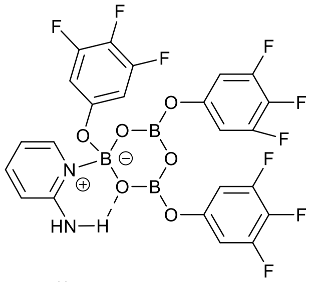

^{11}B NMR: δ_B 13.2 (calc)

Raw NMR data for all of the above experiments can be found at https://doi.org/10.14469/hpc/14846

Analysis of reaction mixtures for catalyst protodeborylation

A series of amidation experiments were performed using 2-chloro-4-fluorophenylboronic acid as catalyst to give a ^{19}F NMR handle for reaction monitoring. In each case, no protodeborylation was observed, though no remaining boronic acid could be detected either. There is no single clear ^{19}F containing species, and many are fairly broad. These are likely to be various adducts of (dehydrated) 2-chloro-4-fluorophenylboronic acid with the amines, carboxylates, and inhibitors present in the reaction.

Benzoic acid (610 mg, 5 mmol) and 1,3,5-trimethoxybenzene (84 mg, 0.5 mmol) were dissolved in TAME (10 ml). Benzylamine (600 µl, 5.5 mmol) was added and the reaction heated to 80 °C, at which point 2-chloro-4-fluorophenylboronic acid (87 mg, 0.5 mmol) was added, a filled Dean-Stark adapter was attached and the reaction heated to reflux. After 1 hour, two aliquots of 250 µl were withdrawn, and 4-fluorobromobenzene (2.5 µl) was added to each as an internal standard for NMR analysis. In one of the samples, 3-flurorochlorobenzene was added to aid identification of any protodeborylation of the catalyst. Each sample was diluted with 400 µl $CDCl_3$ and analysed by ^{19}F NMR spectroscopy. After 18 hours, a second set of samples was taken for similar analysis.

A second reaction was performed in an analogous procedure with addition of 2-aminopyridine (471 mg, 5 mmol, 1 eq.) in the initial reaction mixture.

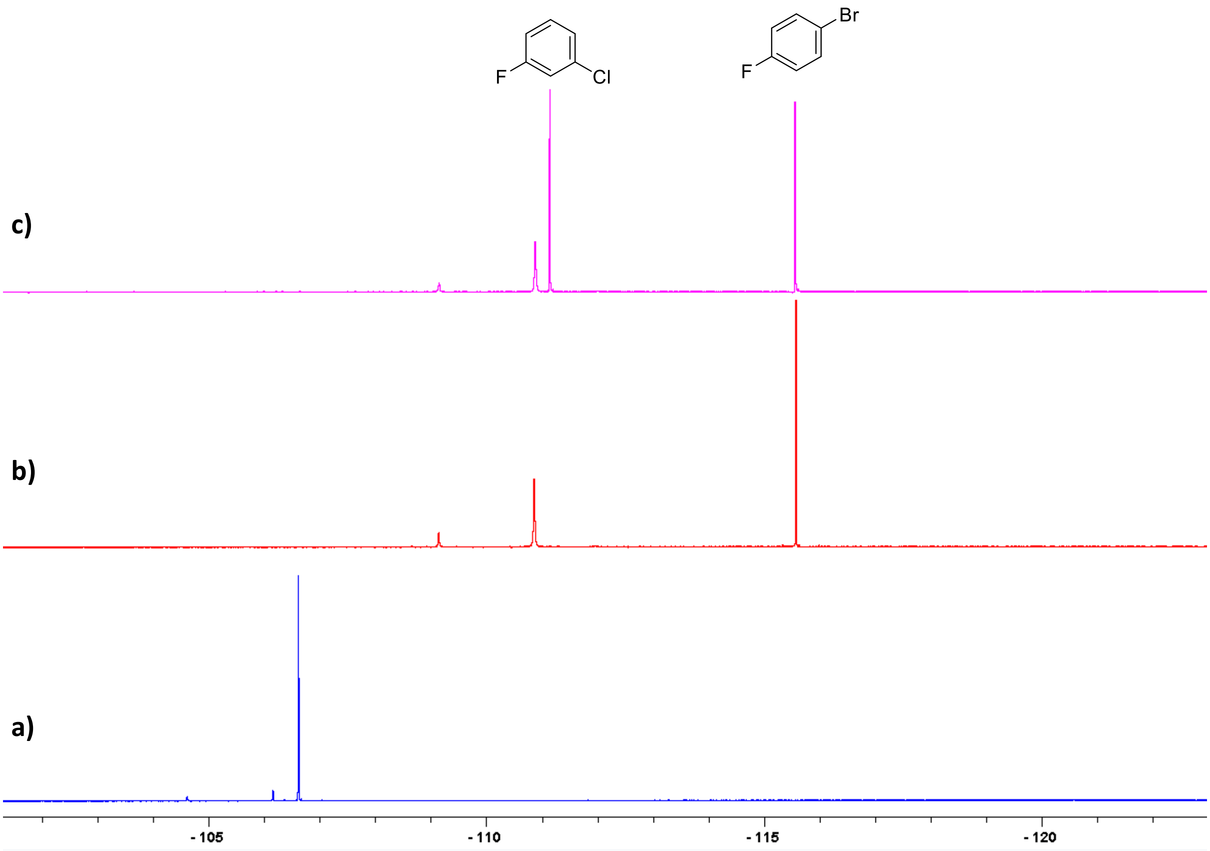

^{19}F NMR spectra of a) 2-chloro-4-fluorophenylboronic acid, b) the reaction mixture after 1 hour, c) the reaction mixture after 1 hour with 3-fluorochlorobenzene added

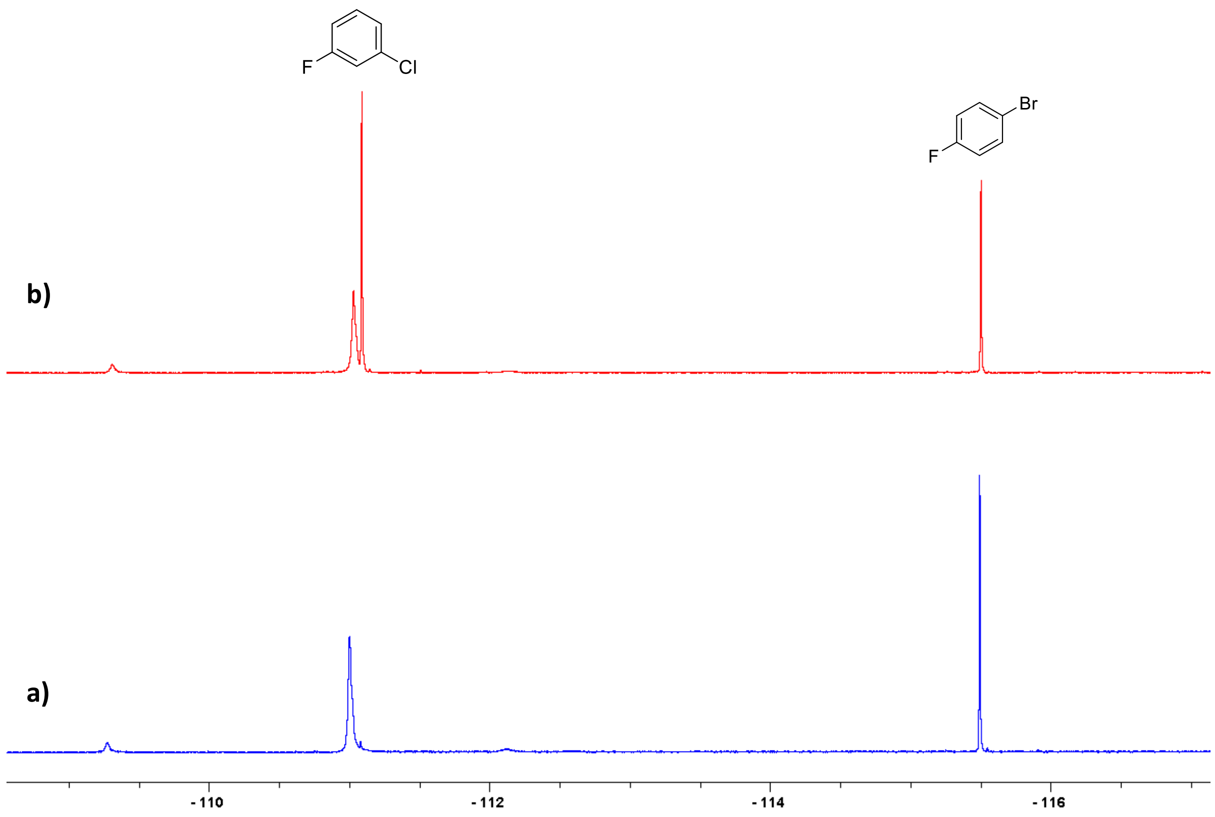

^{19}F NMR spectra of a) the reaction mixture after 18 hours, b) with 3-fluorochlorobenzene added

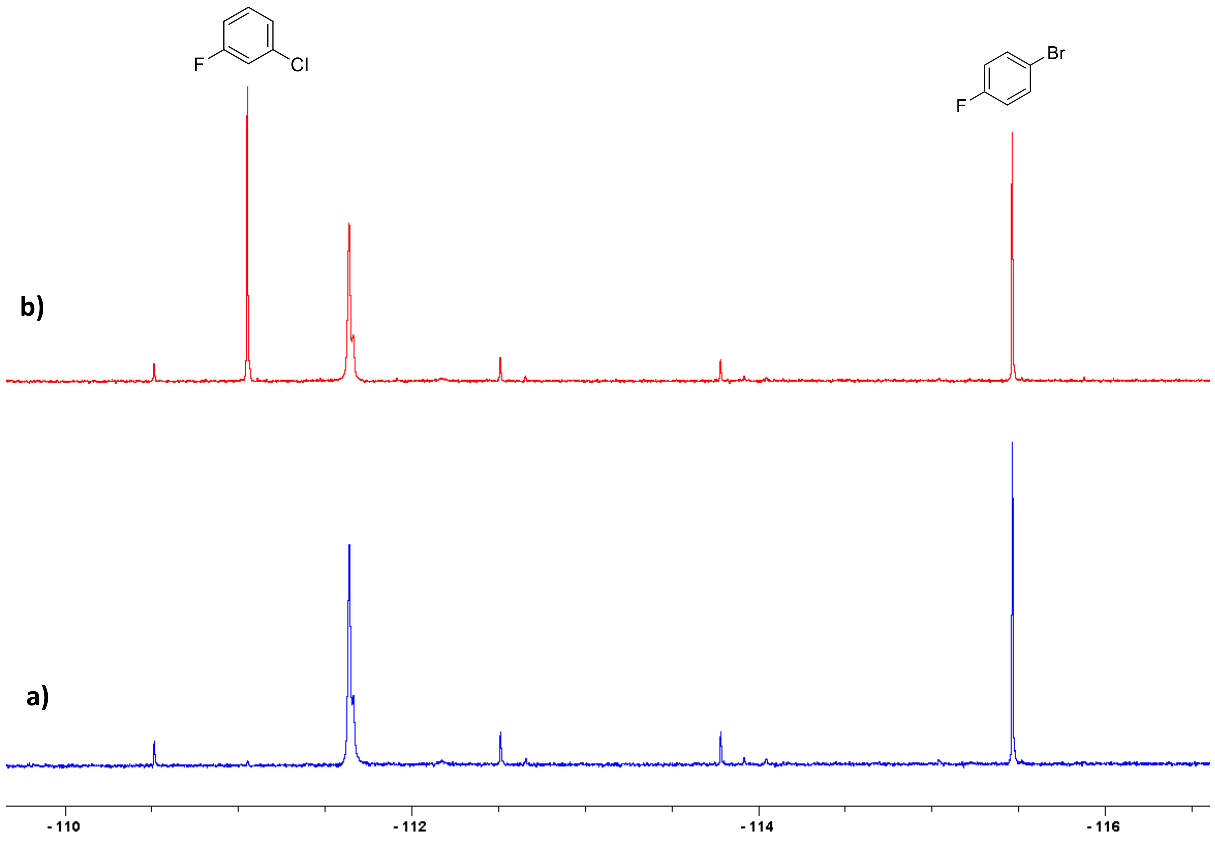

^{19}F NMR spectra of the reaction with 2-amino pyridine as an inhibitor after 18 hours, a) without and b) with 3-fluorochlorobenzene

Phenylacetic acid (681 mg, 5 mmol) and 2-aminopyridine (518 mg, 5.5 mmol) were suspended in TAME (10 ml), heated to 80°C (dissolves on heating), and 2-chloro-4-fluorophenyl boronic acid (87 mg, 0.5 mmol) was added. A filled Dean-Stark adapter was attached and the reaction heated to reflux for 18 hours. After this time, 4-fluorobromobenzene (54.9 µl, 0.5 mmol) was added and an aliquot of 250 µl was taken from the reaction mixture. 3-fluorochlorobenzene (53.5 µl, 0.5 mmol) was added to the remaining reaction mixture, and a further 250 µl aliquot was taken. Each sample was diluted with 400 µl $CDCl_3$ and analysed by ^{19}F NMR spectroscopy.

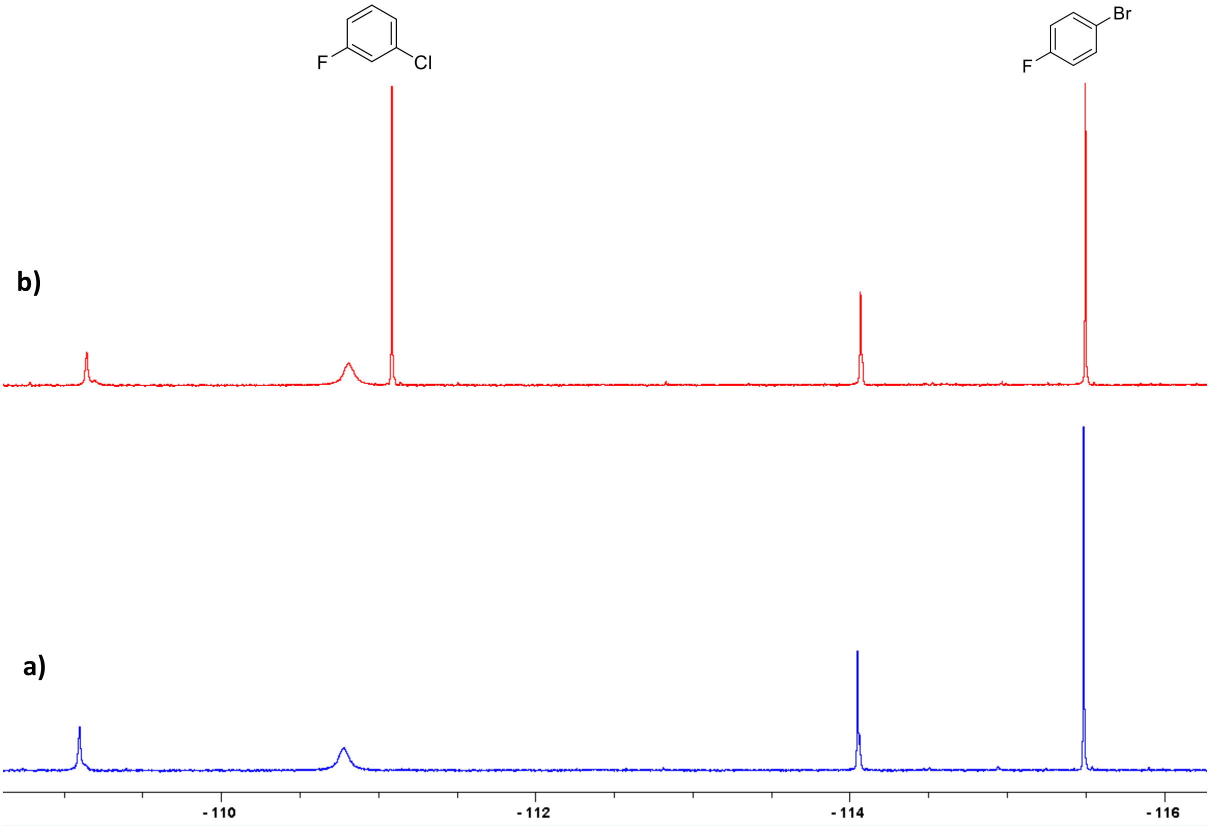

^{19}F NMR spectra of the reaction mixture after 18 hours a) without and b) with 3-fluorochlorobenzene

2-aminopyridine (518 mg, 5.5 mmol) and 2-chloro-4-fluorophenylboronic acid (87 mg, 0.5 mmol) were dissolved in TAME (10 ml), and heated to reflux under a filled Dean-Stark apparatus for 18 hours. After this time, 4-fluorobromobenzene (54.9 µl, 0.5 mmol) was added and an aliquot of 250 µl was taken from the reaction mixture. 3-fluorochlorobenzene (53.5 µl, 0.5 mmol) was added to the remaining reaction mixture, and a further 250 µl aliquot was taken. Each sample was diluted with 400 µl $CDCl_3$ and analysed by ^{19}F NMR spectroscopy. N.B. in this reaction there is significant solid precipitate.

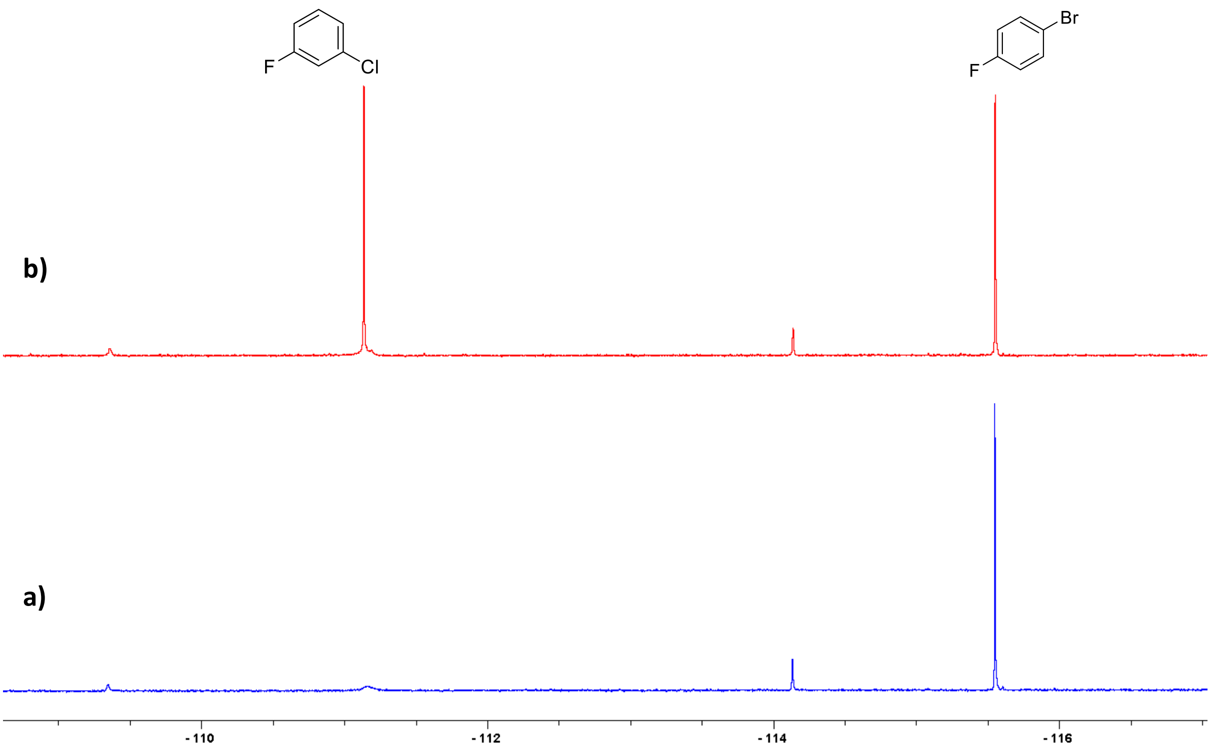

^{19}F NMR spectra of the reaction between excess 2-amino pyridine and 2-chloro-4-fluorophenyl boronic acid, a) without and b) with 3-fluorochlorobenzene

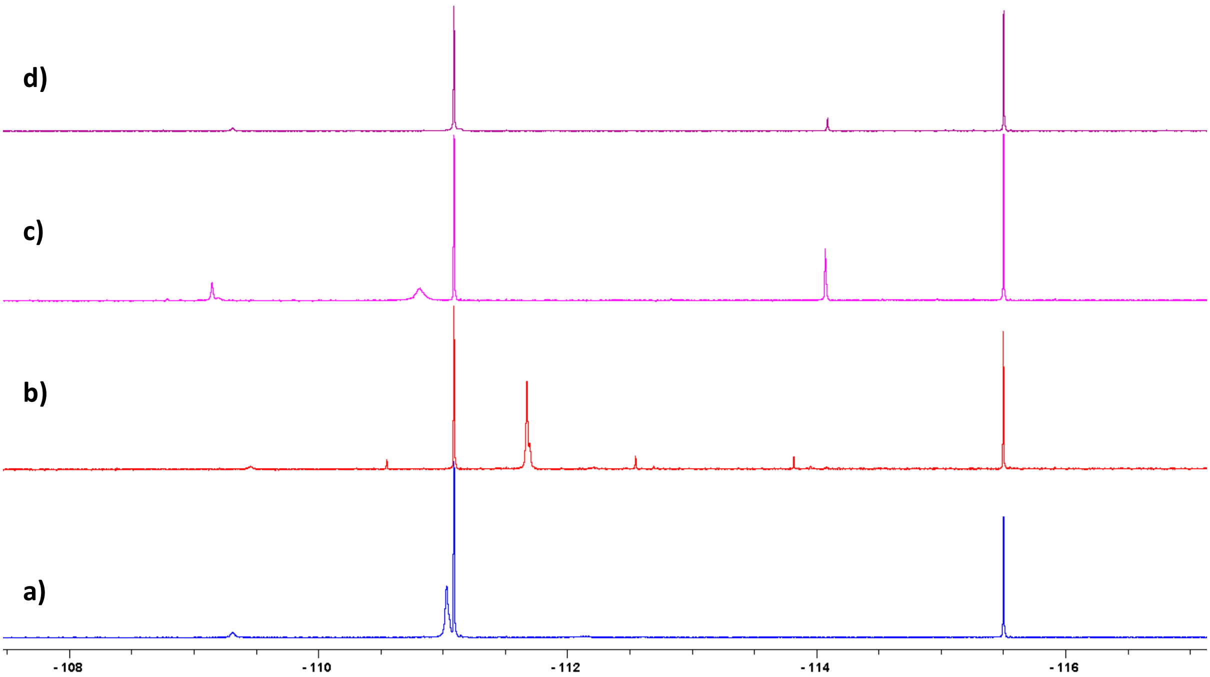

^{19}F NMR spectra, with added 4-fluorobromobenzene and 3-fluorochlorobenzene, of the reactions catalysed by 2-chloro-4-fluorophenylboronic acid a) Benzoic acid + benzylamine, b) benzoic acid + benzylamine + 2-amino pyridine (inhibitor), c) phenylacetic acid + 2-aminopyridine, and d) the reaction of 2-chloro-4-fluorophenylboronic acid with excess 2-aminopyridine.

Characterisation data

***Tris*-(3,4,5-trifluorophenyl) borate**

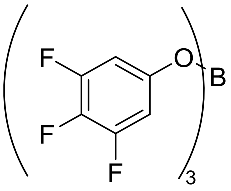

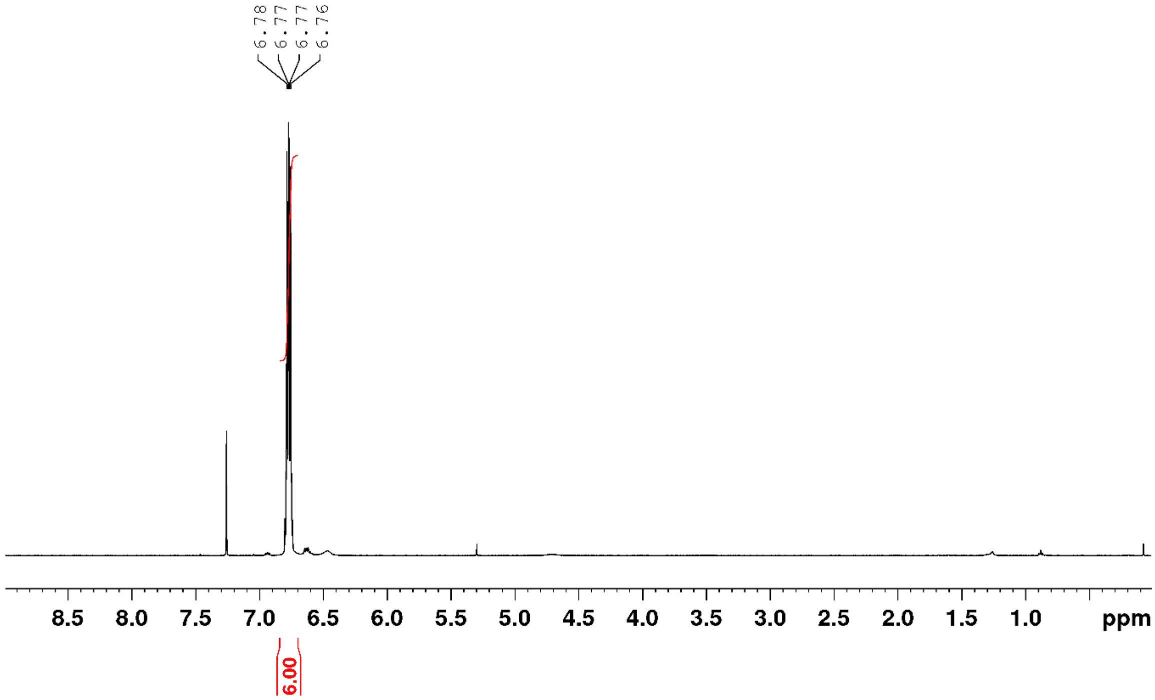

^{1}H NMR spectrum of $B(OAr^F)_3$ in $CDCl_3$

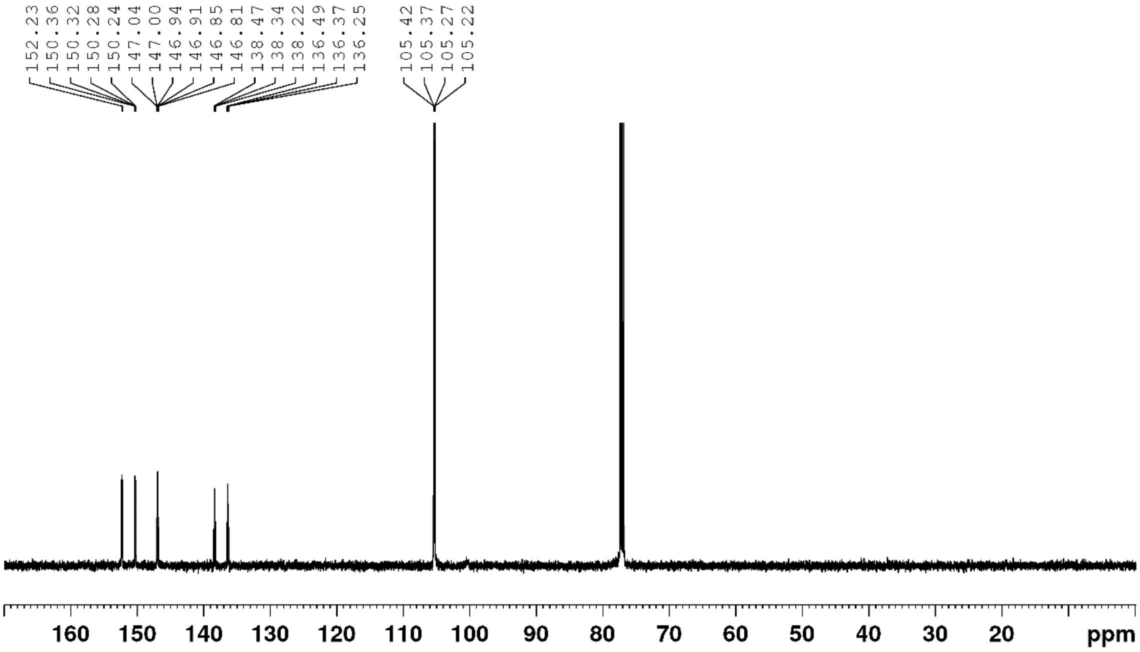

^{13}C NMR spectrum of $B(OAr^F)_3$ in $CDCl_3$

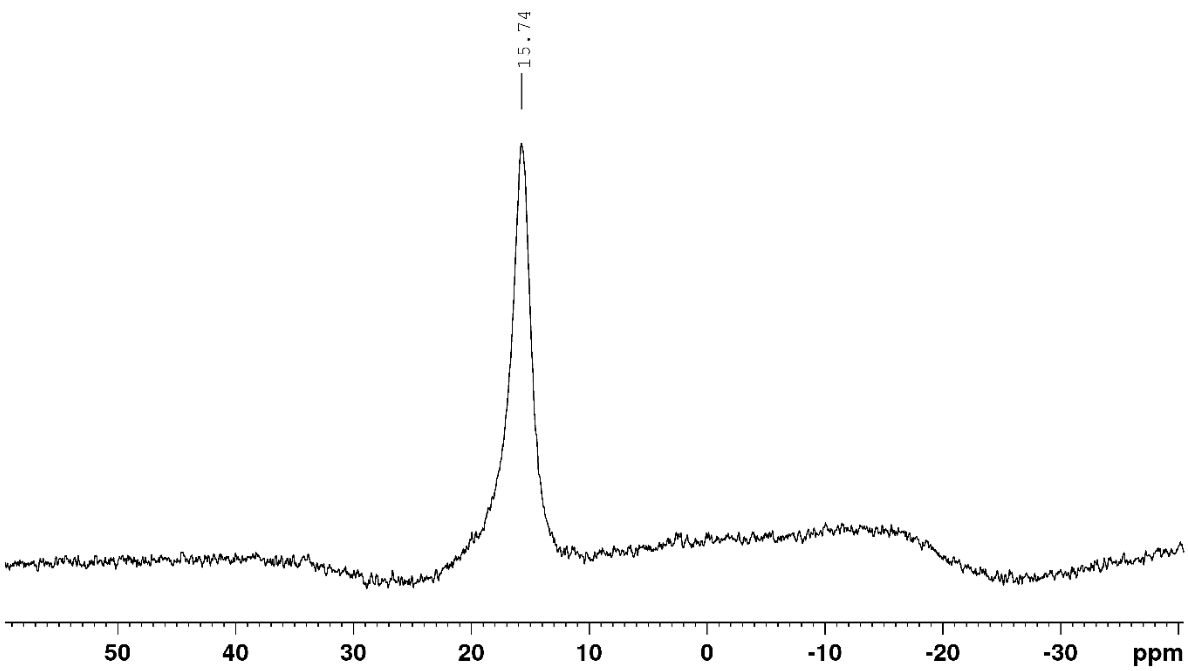

^{11}B NMR spectrum of $B(OAr^F)_3$ in $CDCl_3$

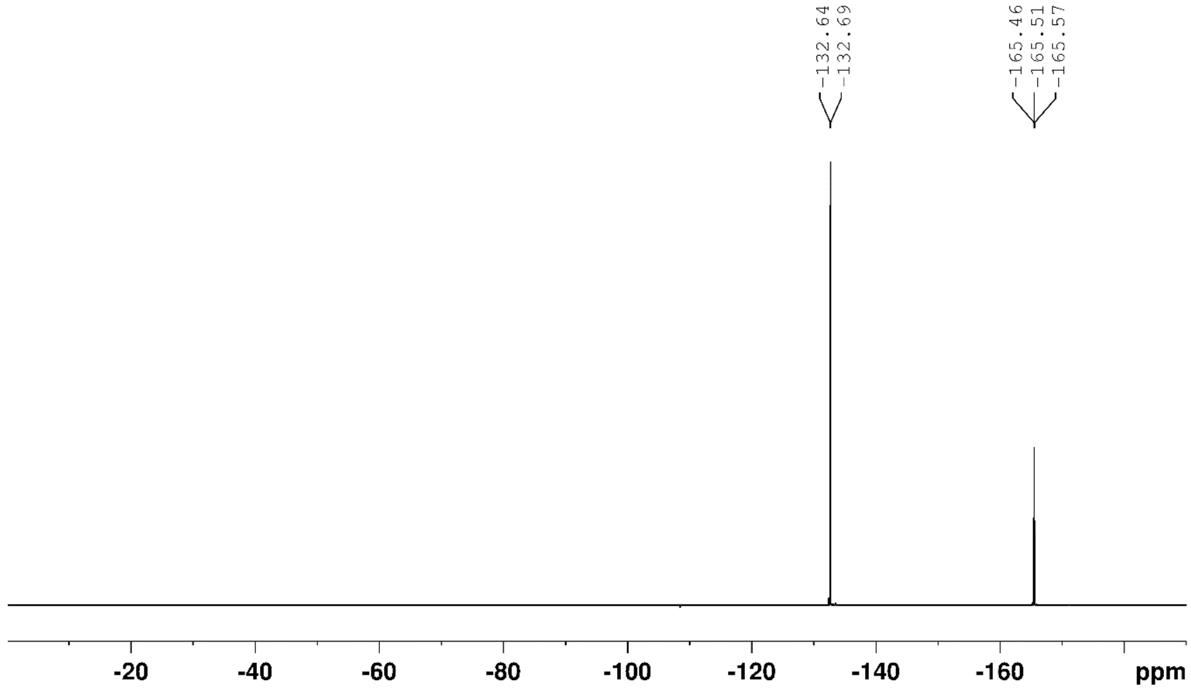

^{19}F NMR spectrum of $B(OAr^F)_3$ in $CDCl_3$.

***Tris*-(2,2,3,3,4,4,5,5-octafluoropentyl) borate**

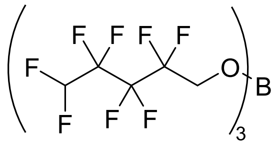

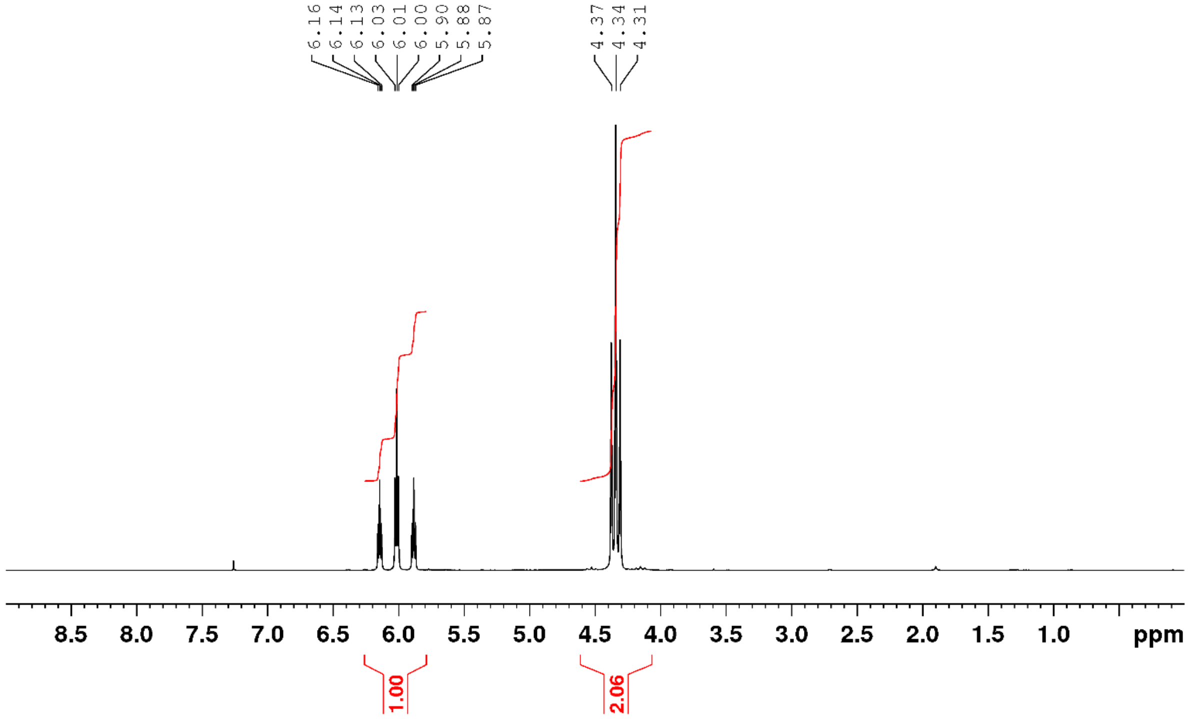

^1H NMR spectrum of $B(OCH_2(CF_2)_3CF_2H)_3$ in $CDCl_3$.

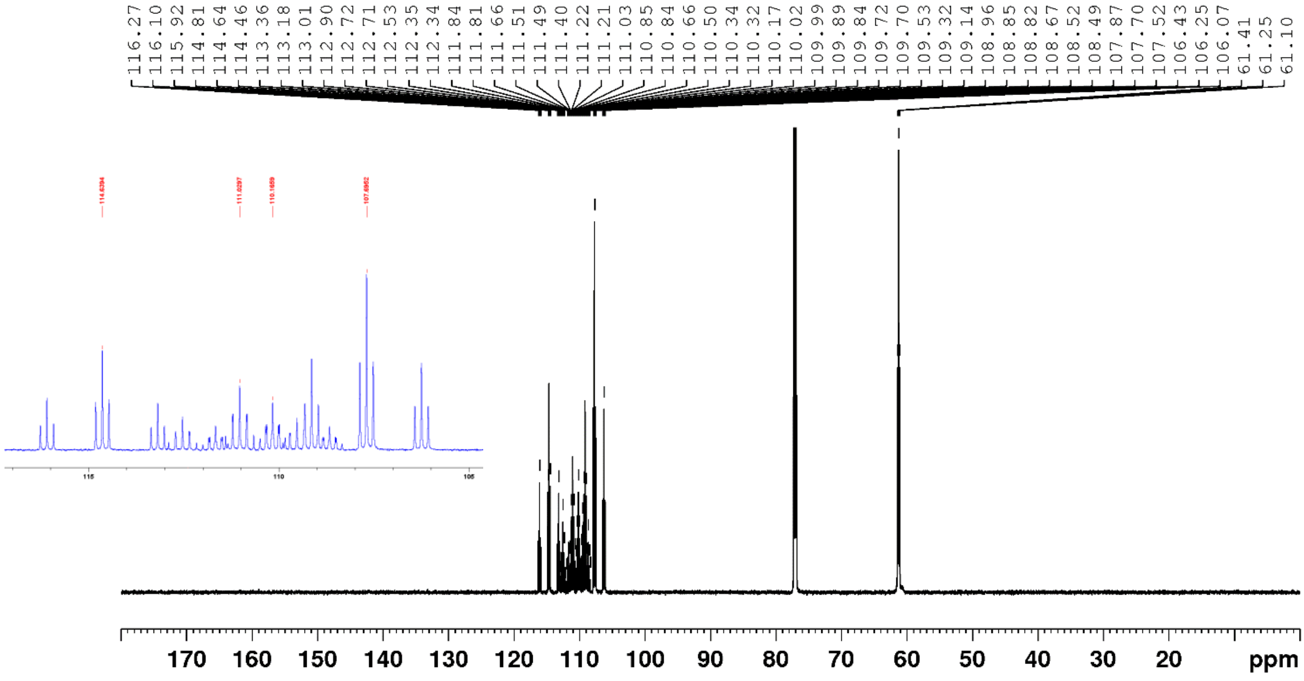

^{13}C NMR spectrum of $B(OCH_2(CF_2)_3CF_2H)_3$ in $CDCl_3$.

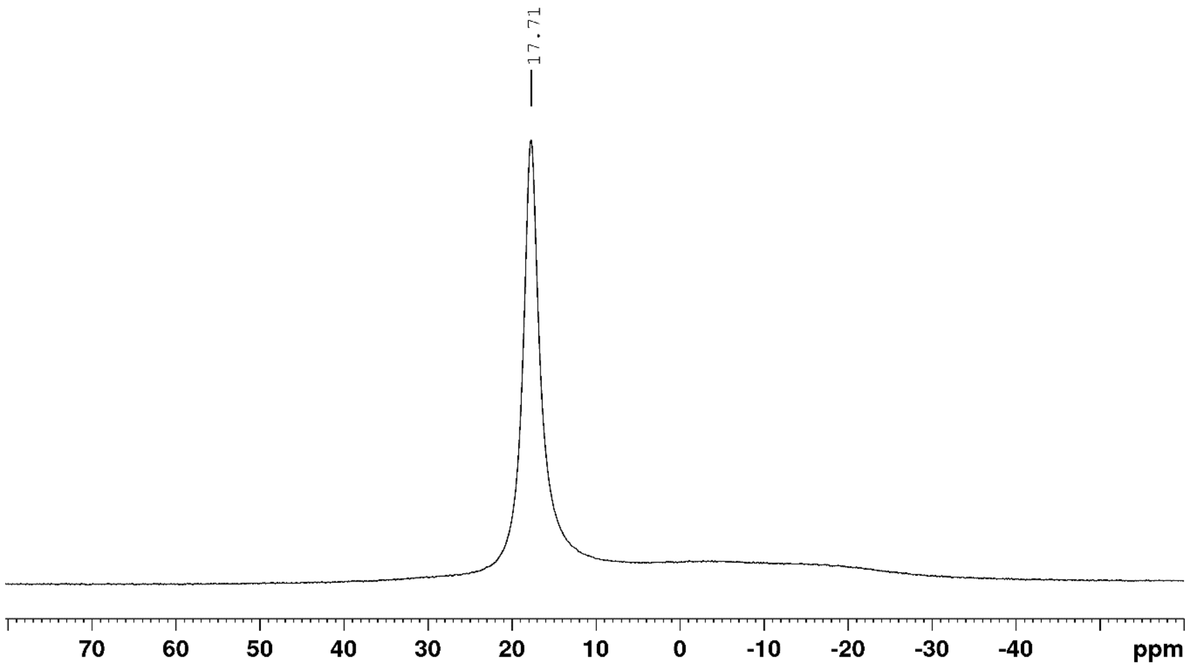

^{11}B NMR spectrum of $B(OCH_2(CF_2)_3CF_2H)_3$ in $CDCl_3$.

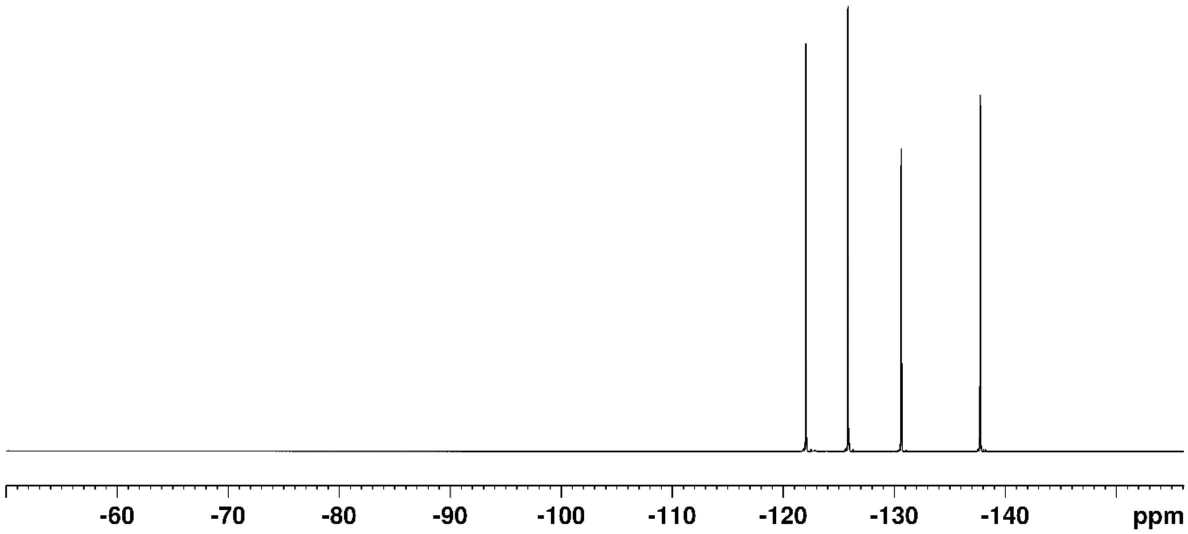

^{19}F NMR spectrum of $B(OCH_2(CF_2)_3CF_2H)_3$ in $CDCl_3$.

***Tris*-(2,2,2-trichloroethyl) borate**

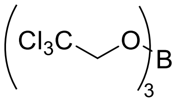

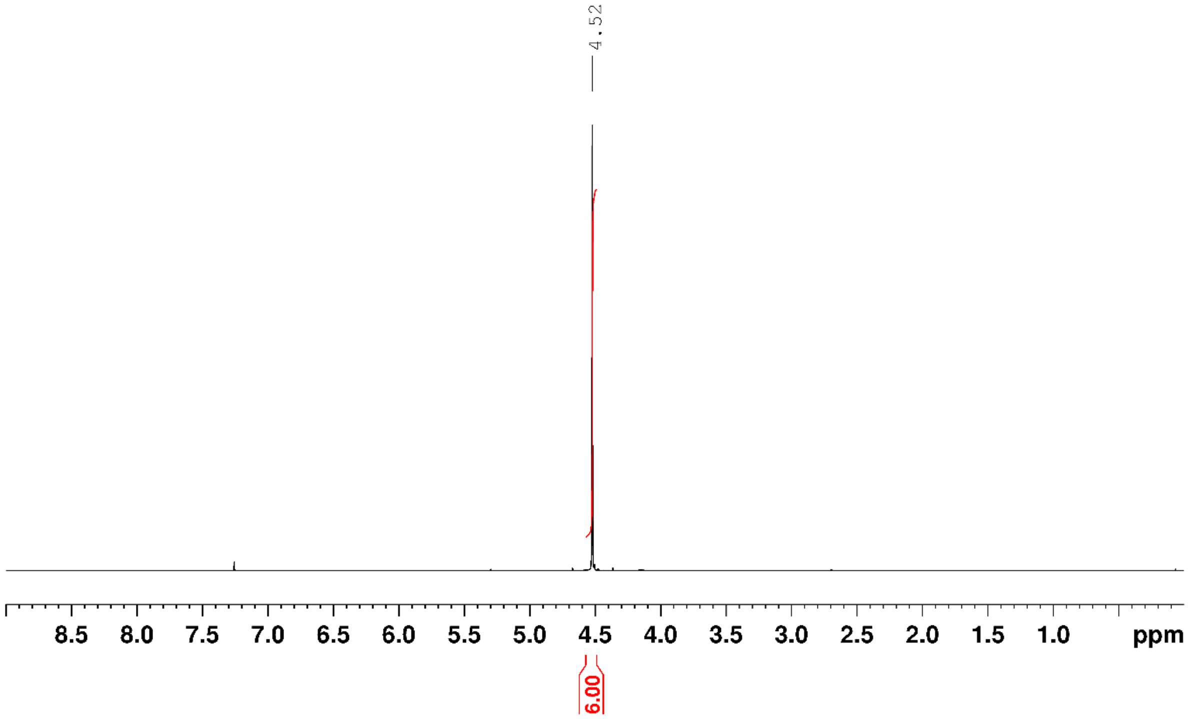

^{1}H NMR spectrum of trichloroethylborate in $CDCl_3$.

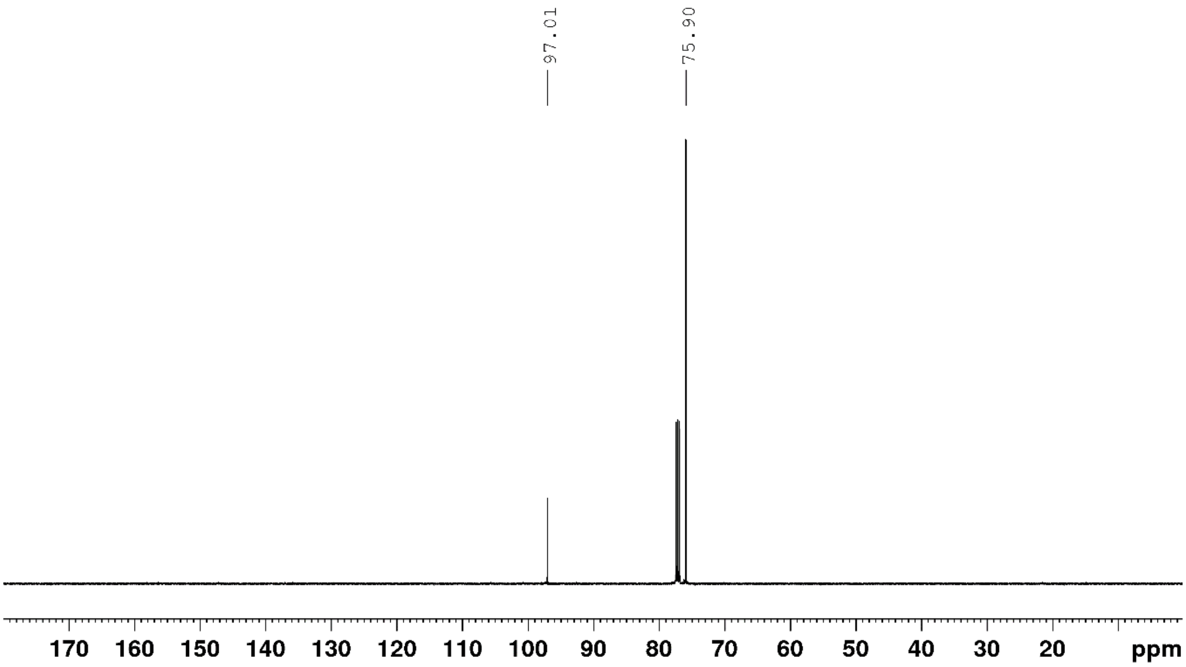

^{13}C NMR spectrum of trichloroethylborate in $CDCl_3$.

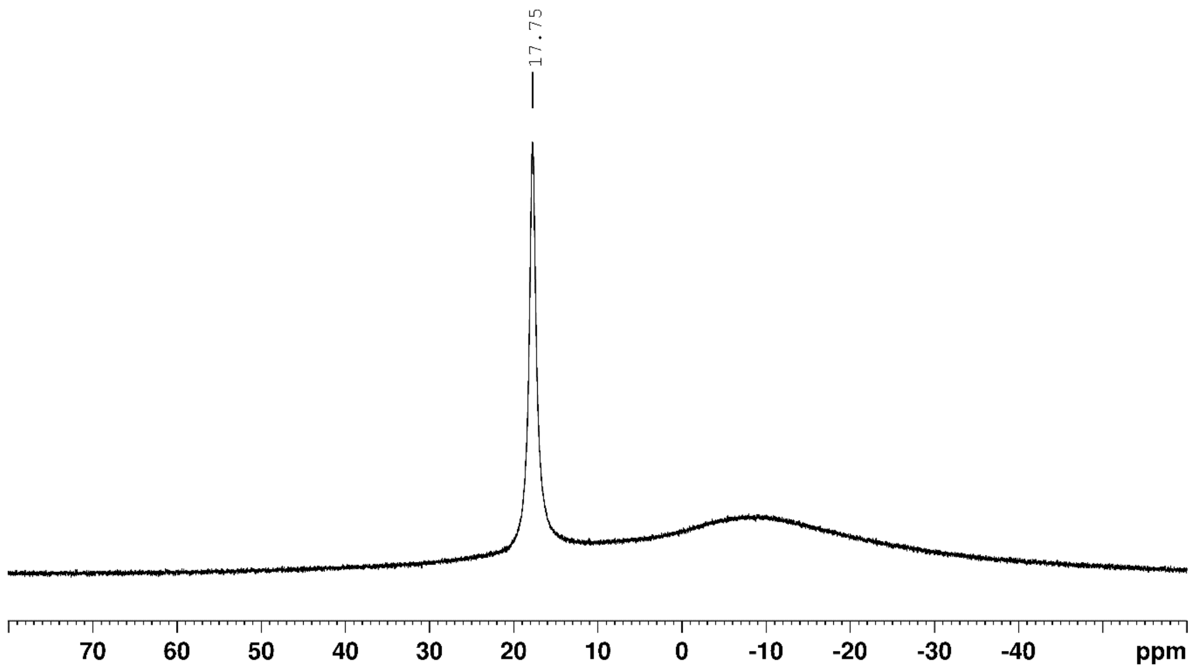

^{11}B NMR spectrum of trichloroethylborate in $CDCl_3$.

Tris-(2,4,6-trichlorophenyl) borate

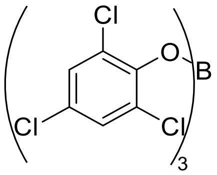

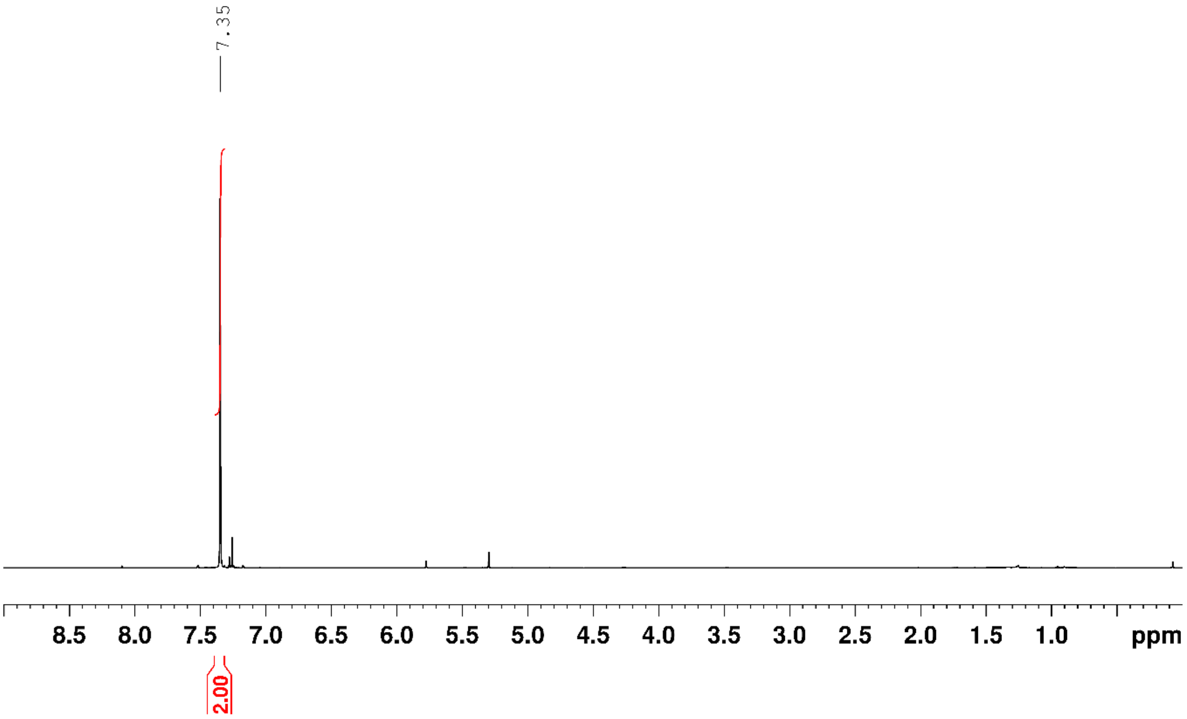

^{1}H NMR spectrum of **D** in $CDCl_3$

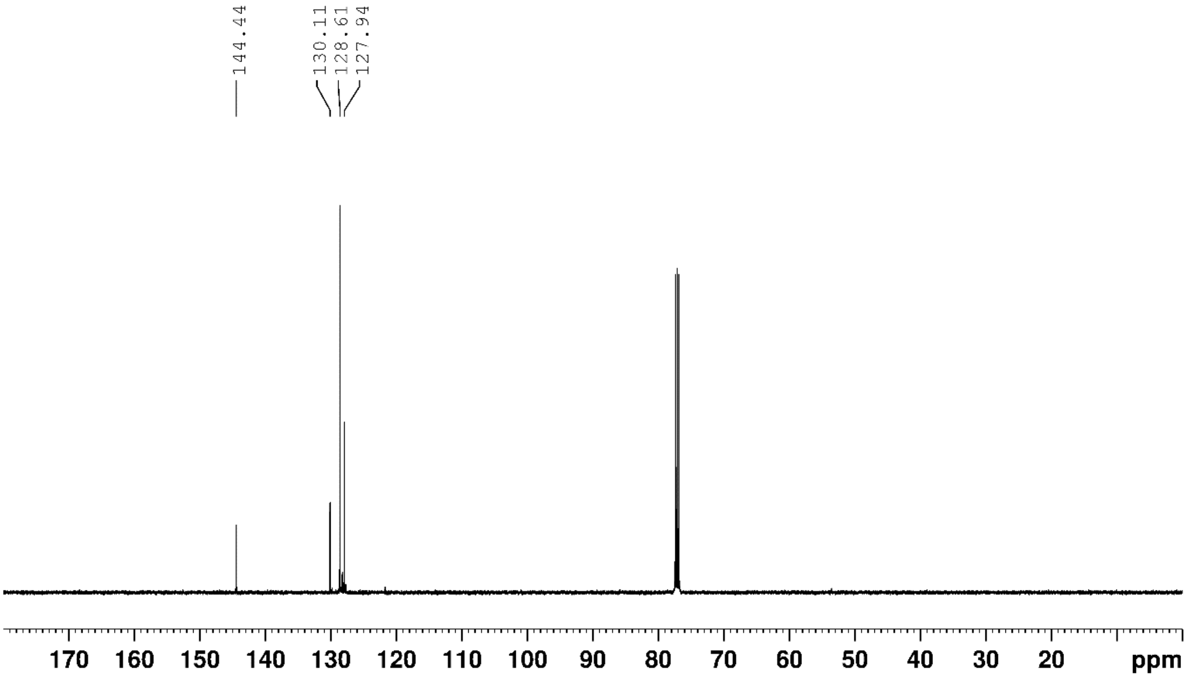

^{13}C NMR spectrum of **D** in $CDCl_3$

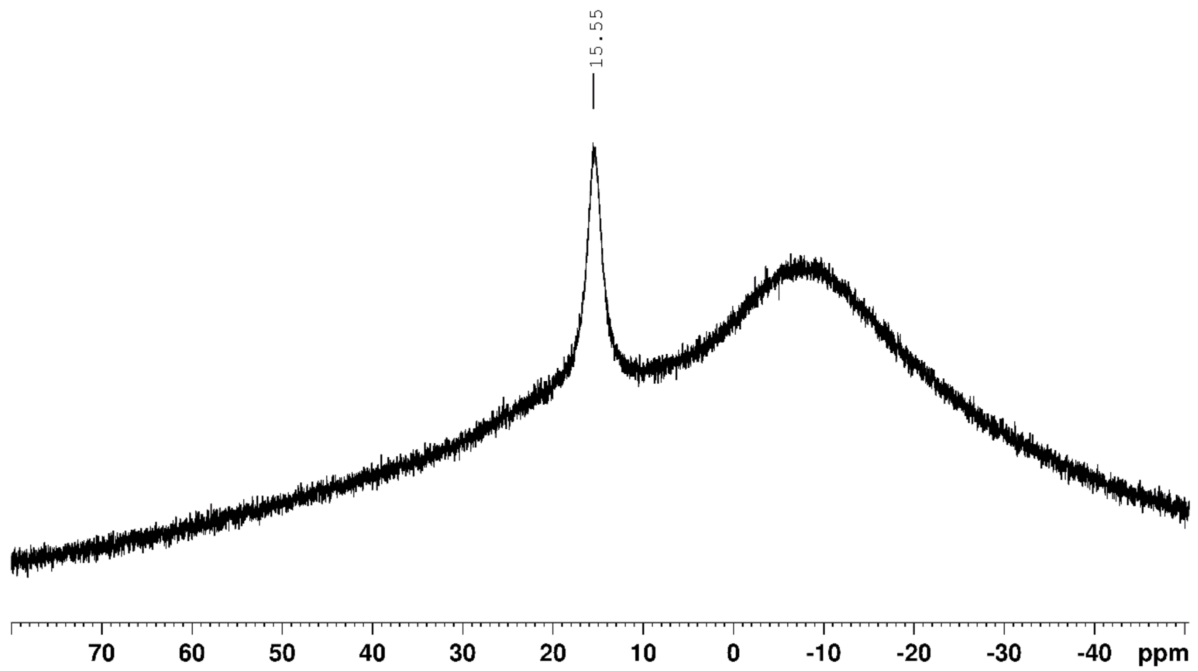

^{11}B NMR spectrum of **D** in $CDCl_3$

***N*-Benzylbenzamide (3)**

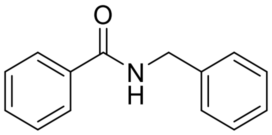

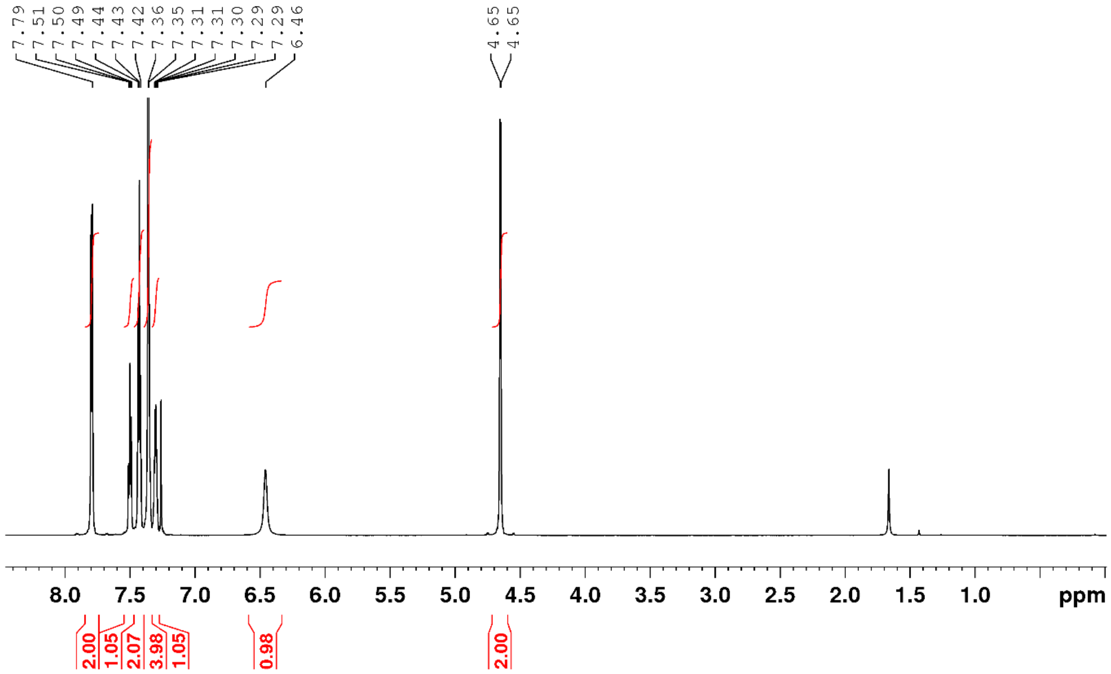

^{1}H NMR spectrum of **3** in $CDCl_3$

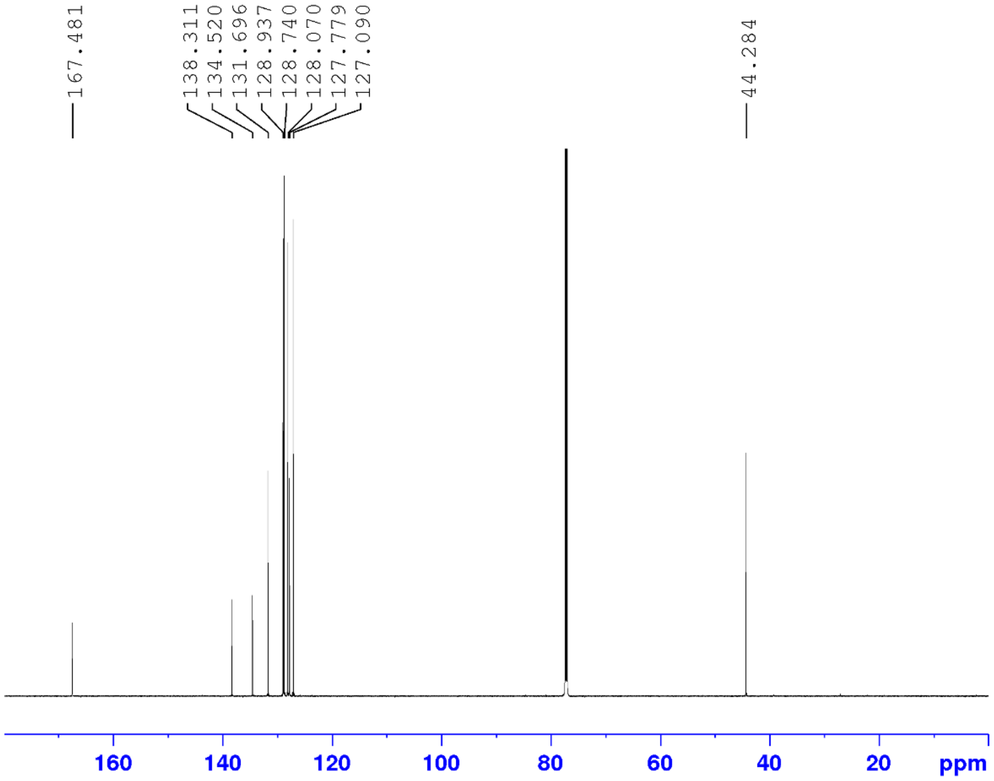

^{13}C NMR spectrum of **3** in $CDCl_3$

2-(3-Fluorophenyl)-N-(pyridin-2-yl)acetamide (4)

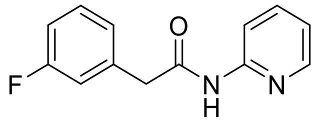

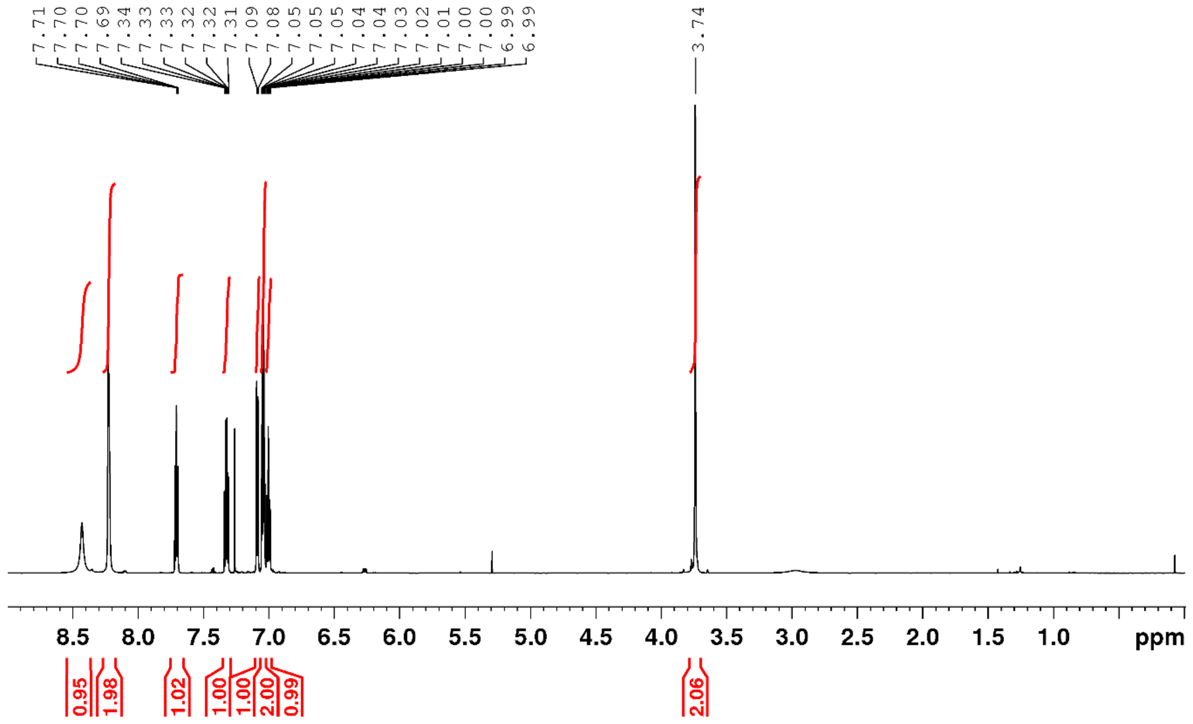

^{1}H NMR spectrum of **4** in $CDCl_3$

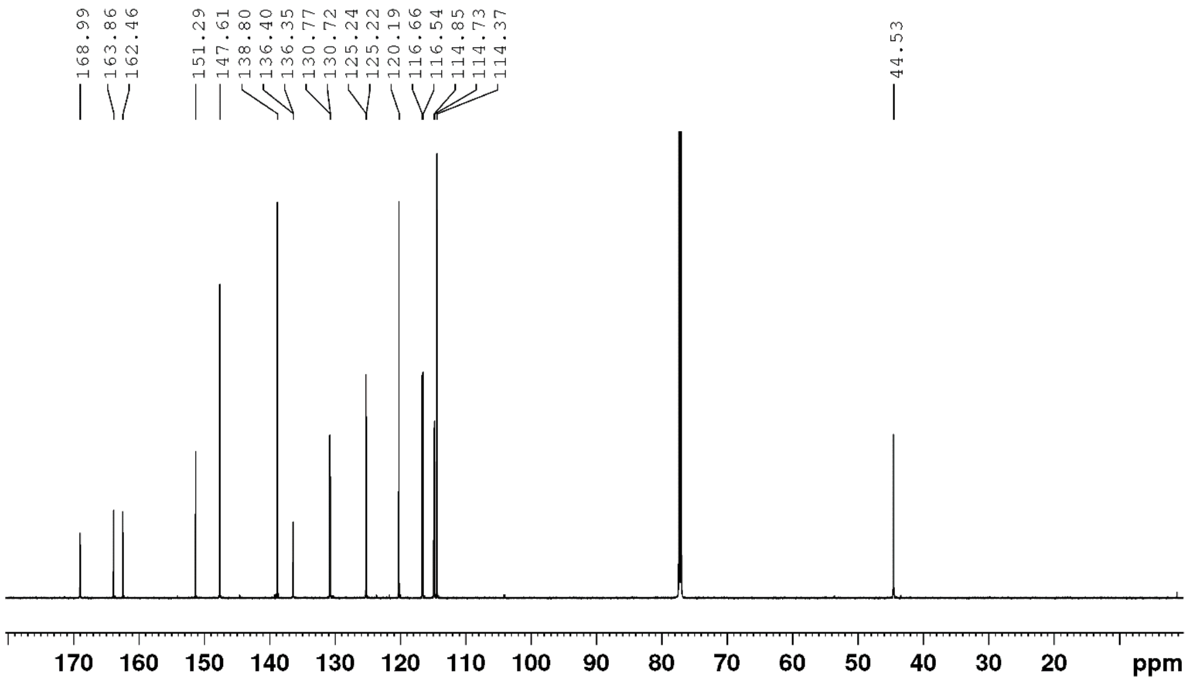

^{13}C NMR spectrum of **4** in $CDCl_3$

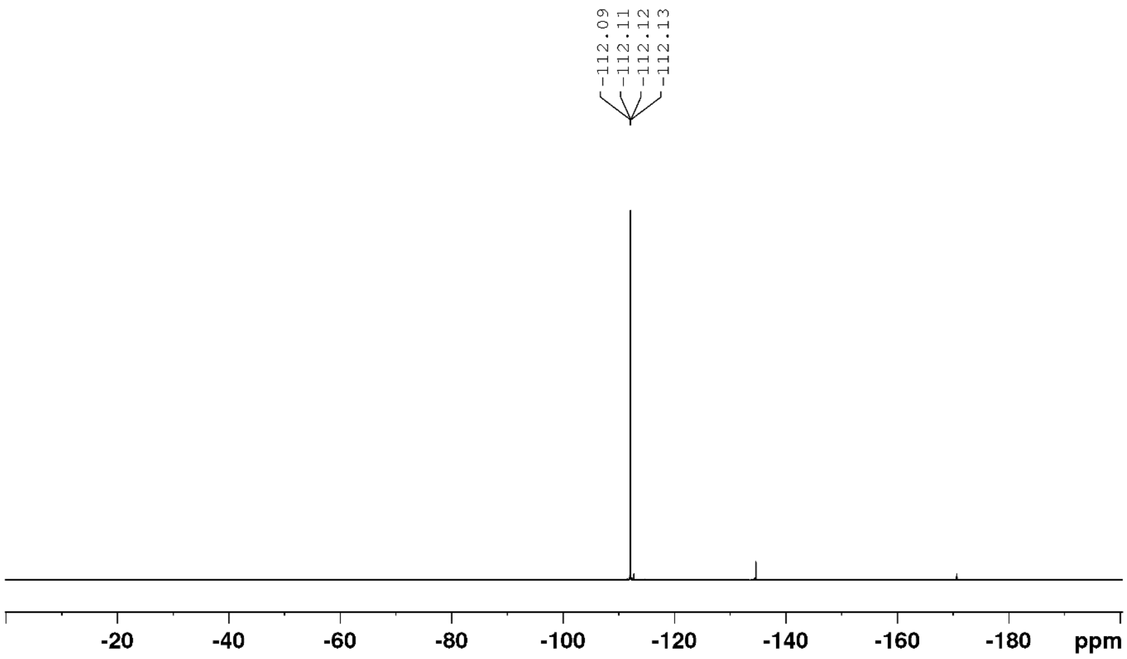

^{19}F NMR spectrum of **4** in $CDCl_3$

N-(2-(Dimethylamino)-5-(trifluoromethyl)phenyl)-2-phenylacetamide (5)

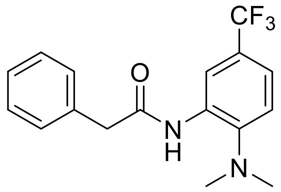

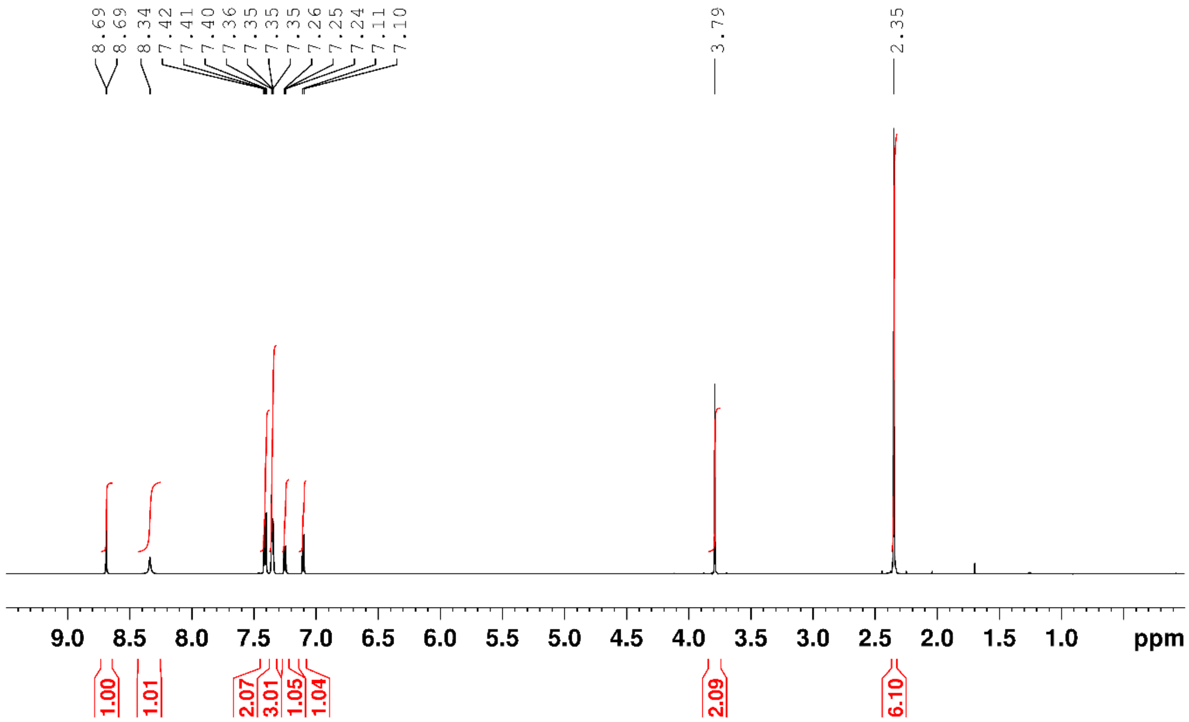

^{1}H NMR spectrum of **5** in $CDCl_3$

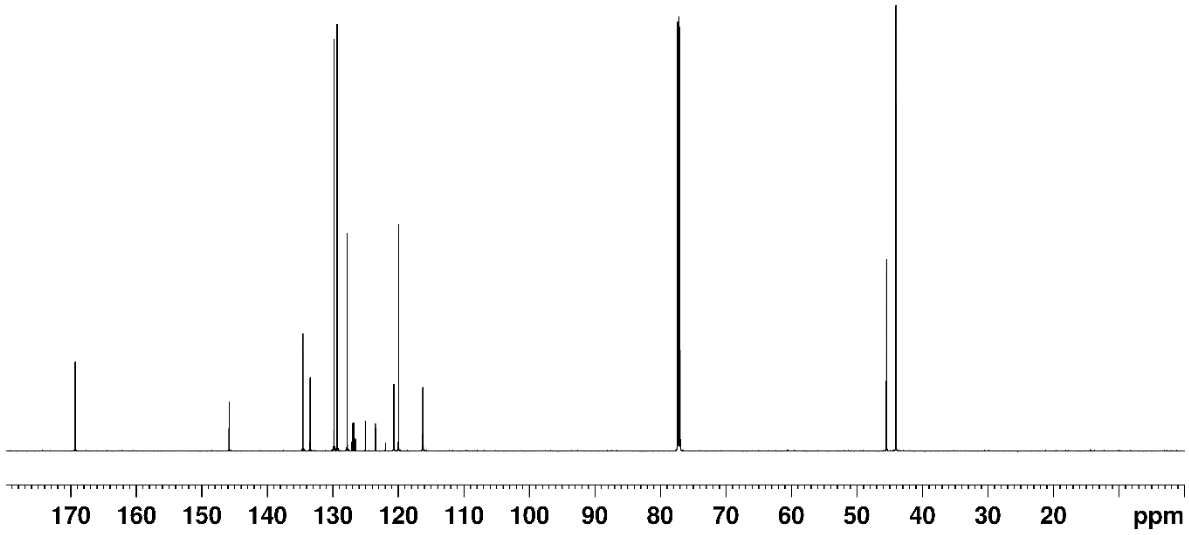

^{13}C NMR spectrum of **5** in $CDCl_3$

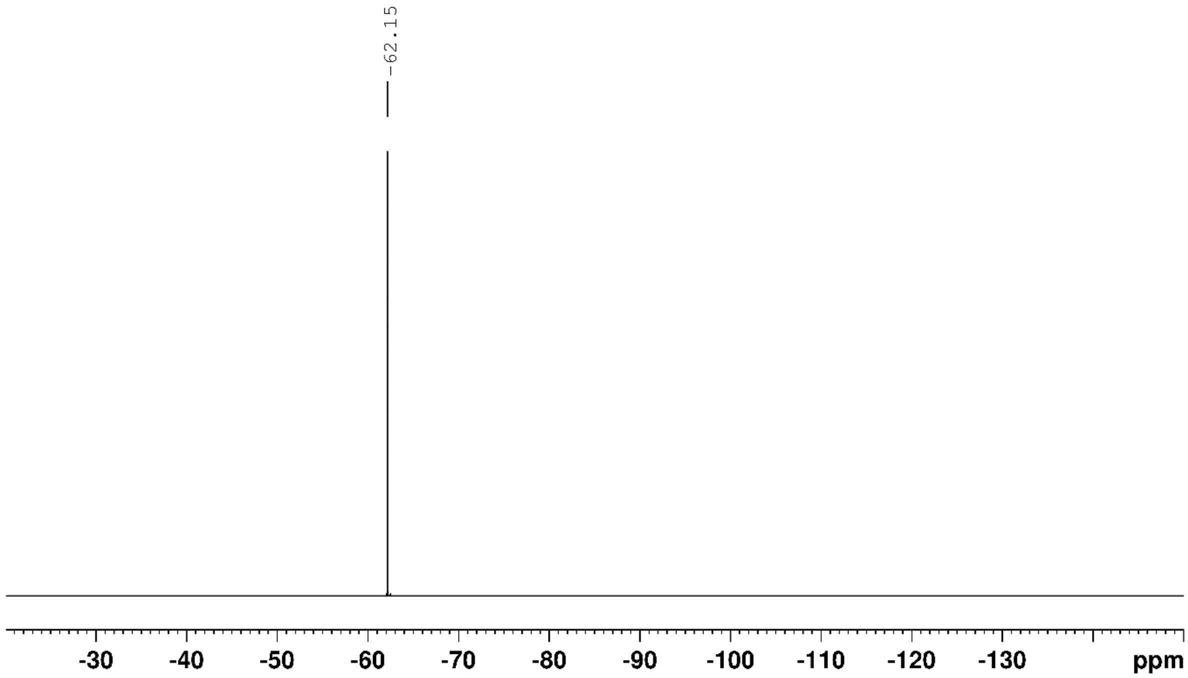

^{19}F NMR spectrum of **5** in $CDCl_3$

2-Phenyl-N-(quinolin-8-yl)acetamide (6)

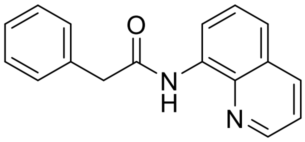

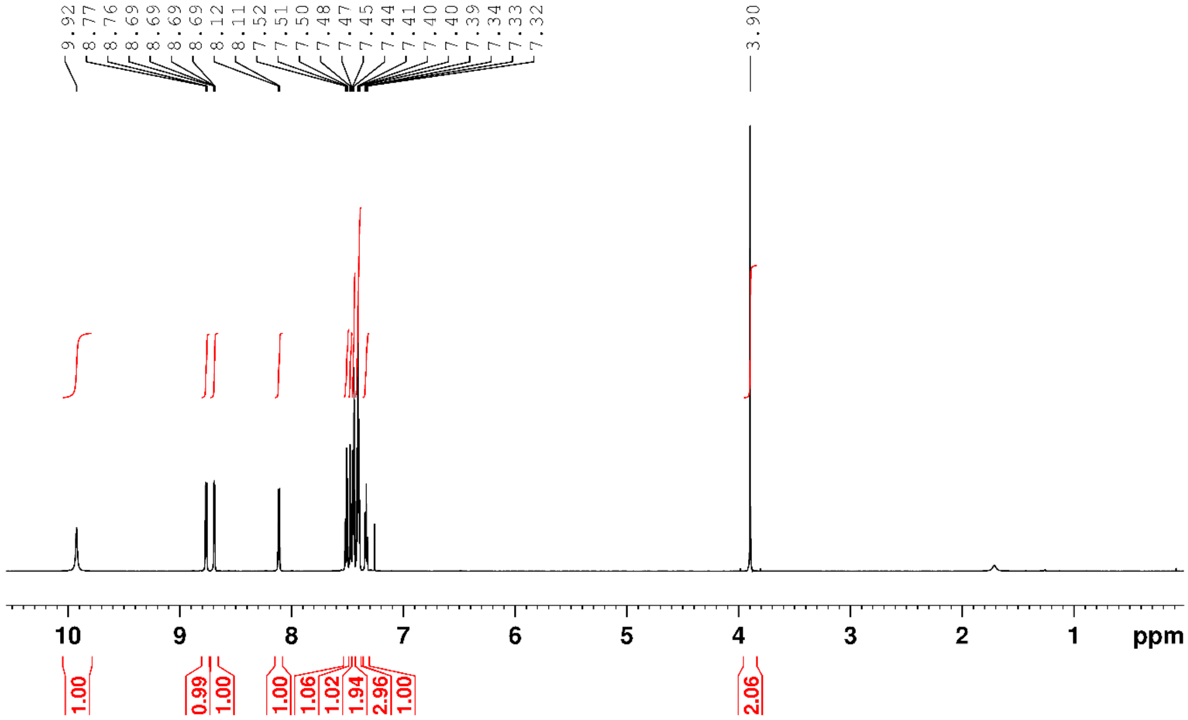

^{1}H NMR spectrum of **6** in $CDCl_3$

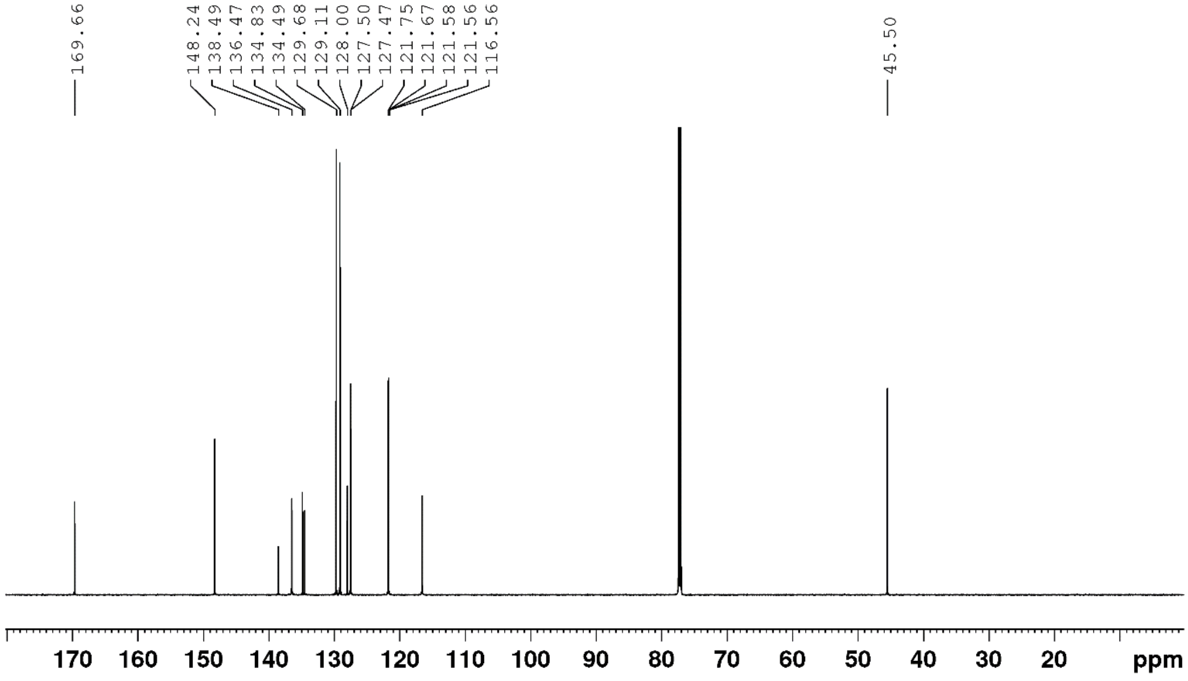

^{13}C NMR spectrum of **6** in $CDCl_3$

***N*-Benzyl-1H-indazole-3-carboxamide (7)**

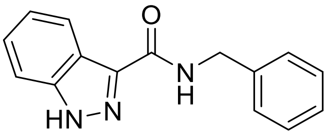

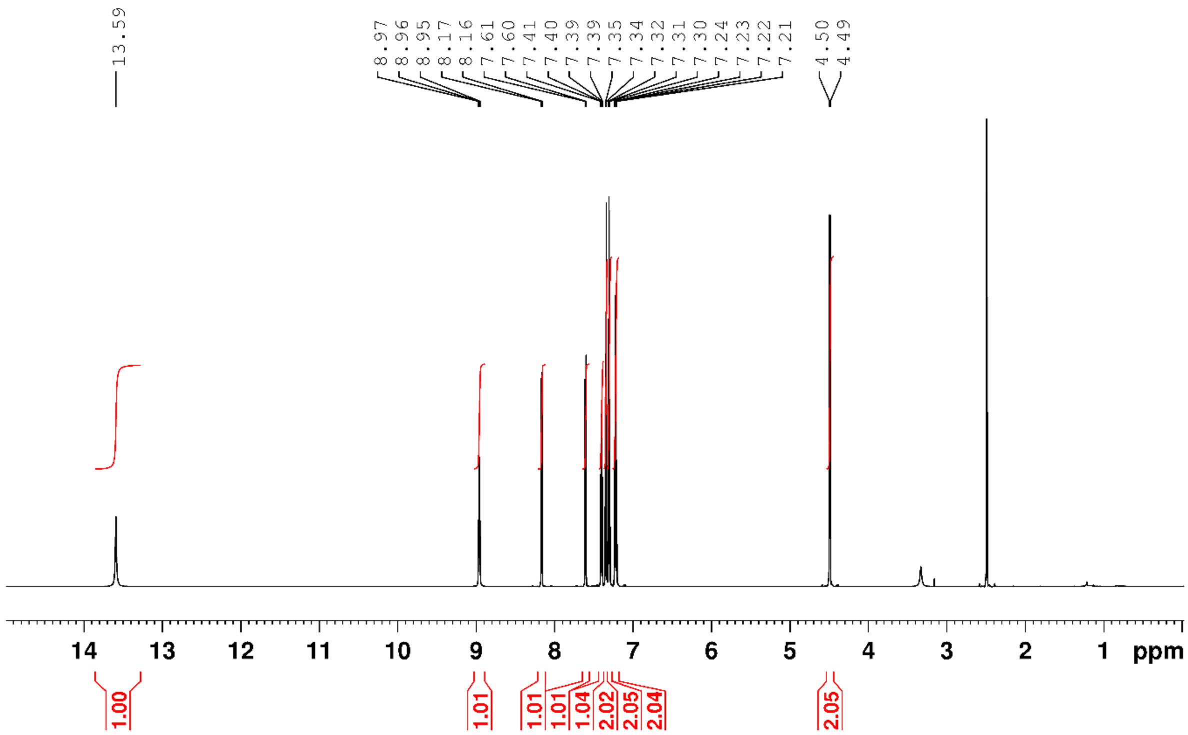

^{1}H NMR spectrum of **7** in DMSO-d6

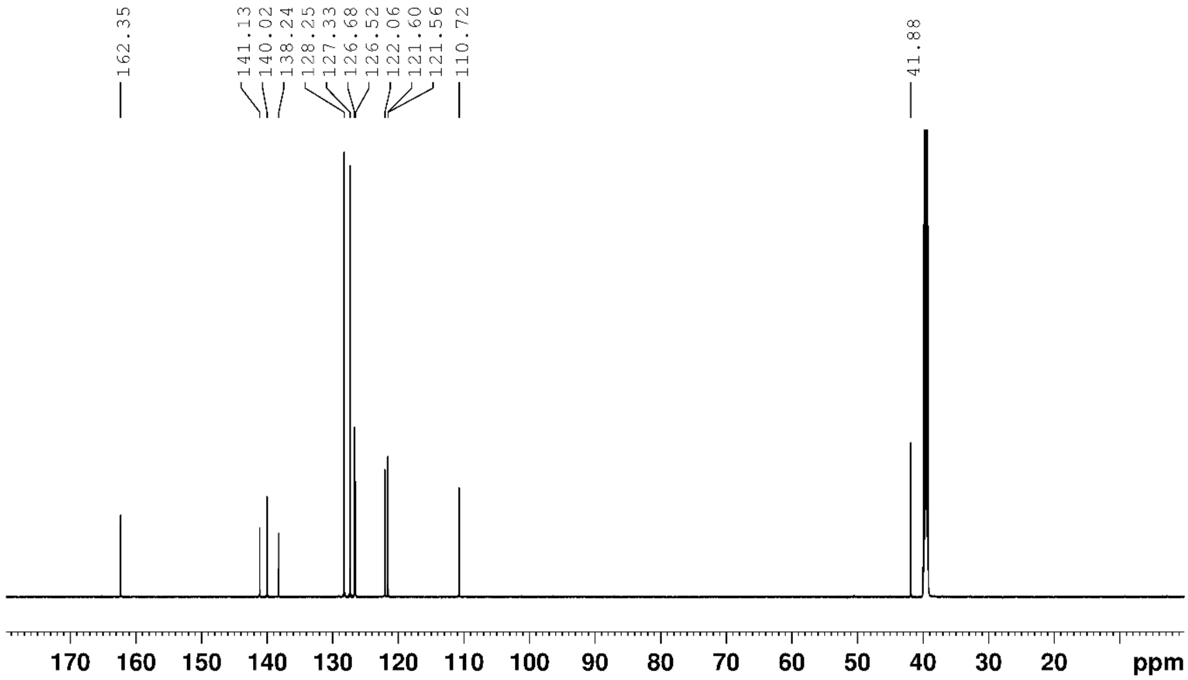

^{13}C NMR spectrum of **7** in DMSO-d6.

tert-Butyl 4-(quinolin-8-ylcarbamoyl)piperidine-1-carboxylate (8)

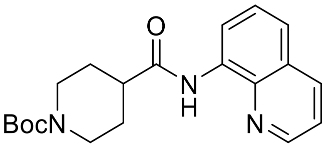

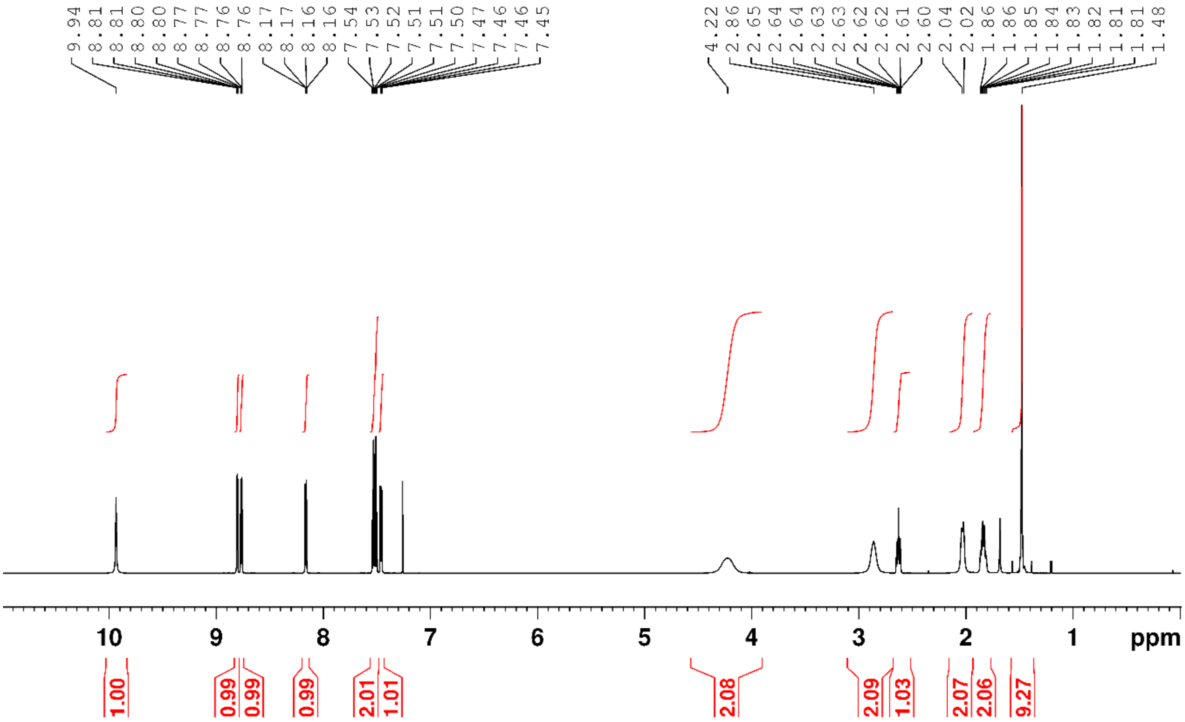

^{1}H NMR spectrum of **8** in $CDCl_3$

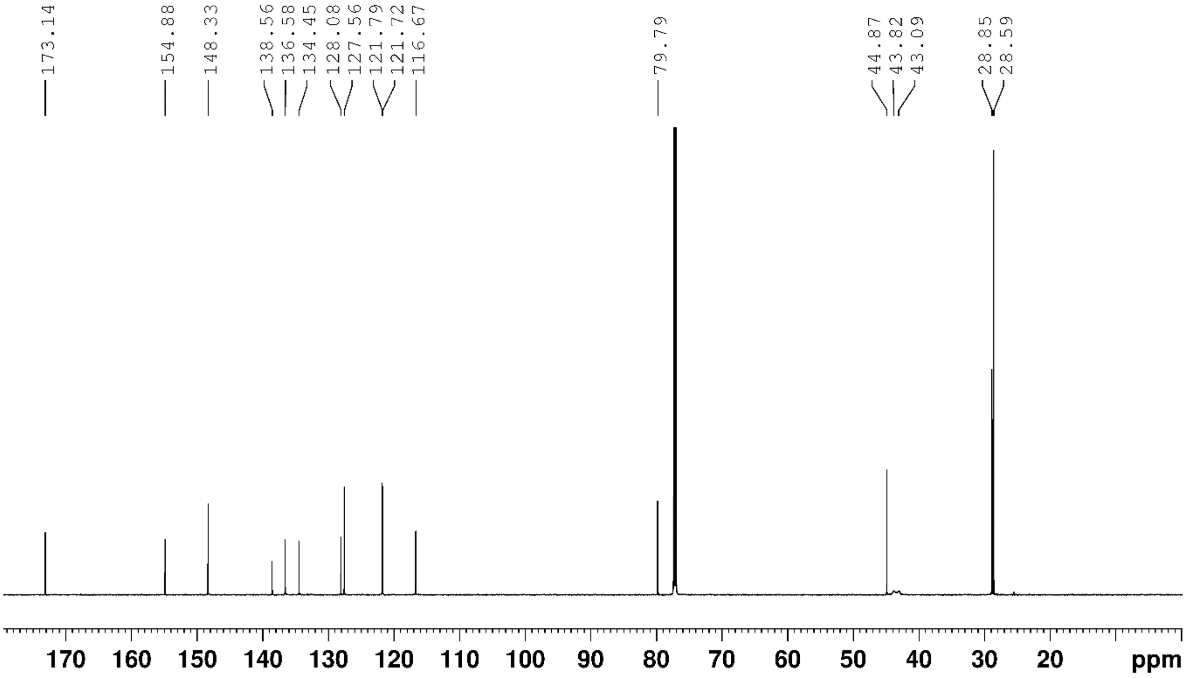

^{13}C NMR spectrum of **8** in $CDCl_3$

_N_-(2-(Dimethylamino)ethyl)-2-phenylacetamide (9)

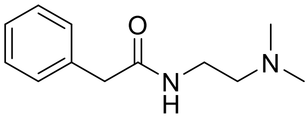

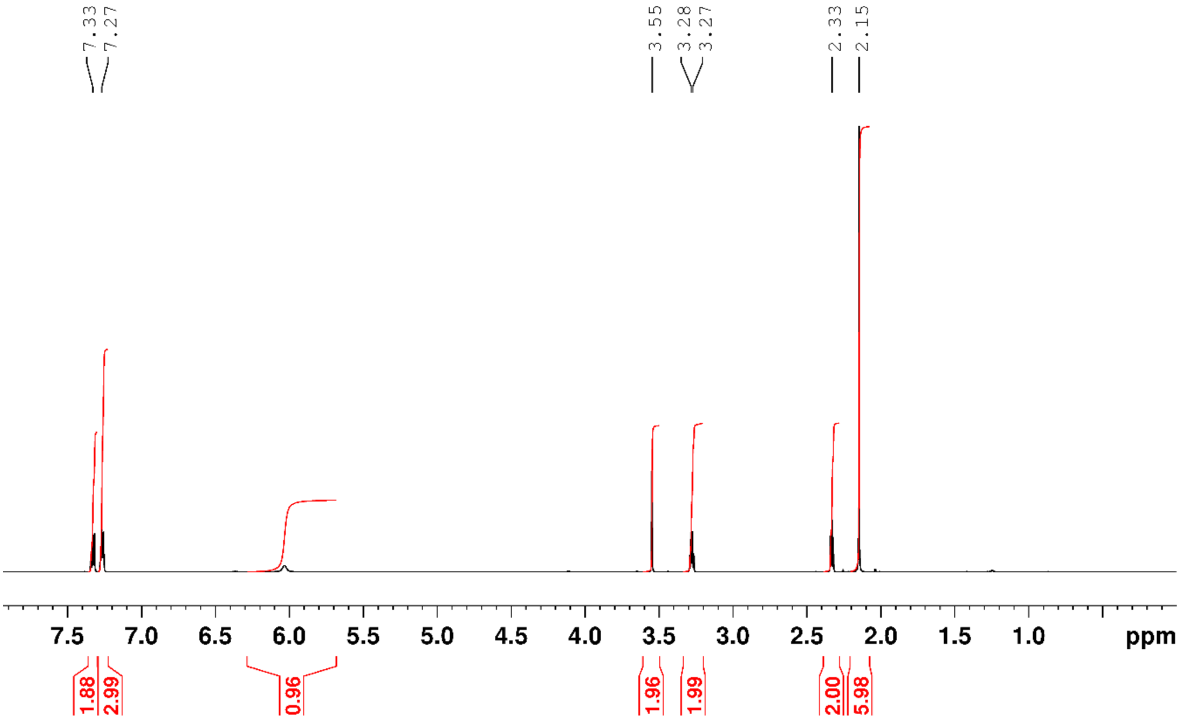

^{1}H NMR spectrum of **9** in $CDCl_3$

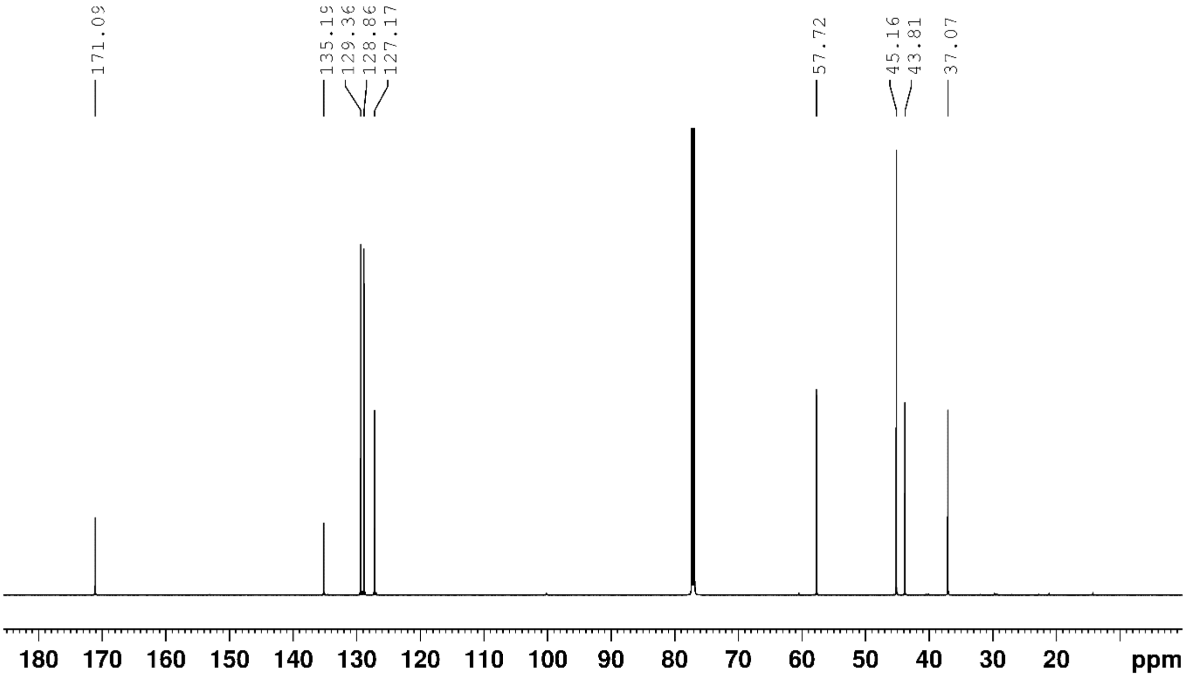

^{13}C NMR spectrum of **9** in $CDCl_3$.

2-Phenyl-*N*-(1H-pyrazol-3-yl)acetamide (10)

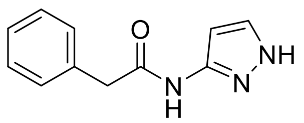

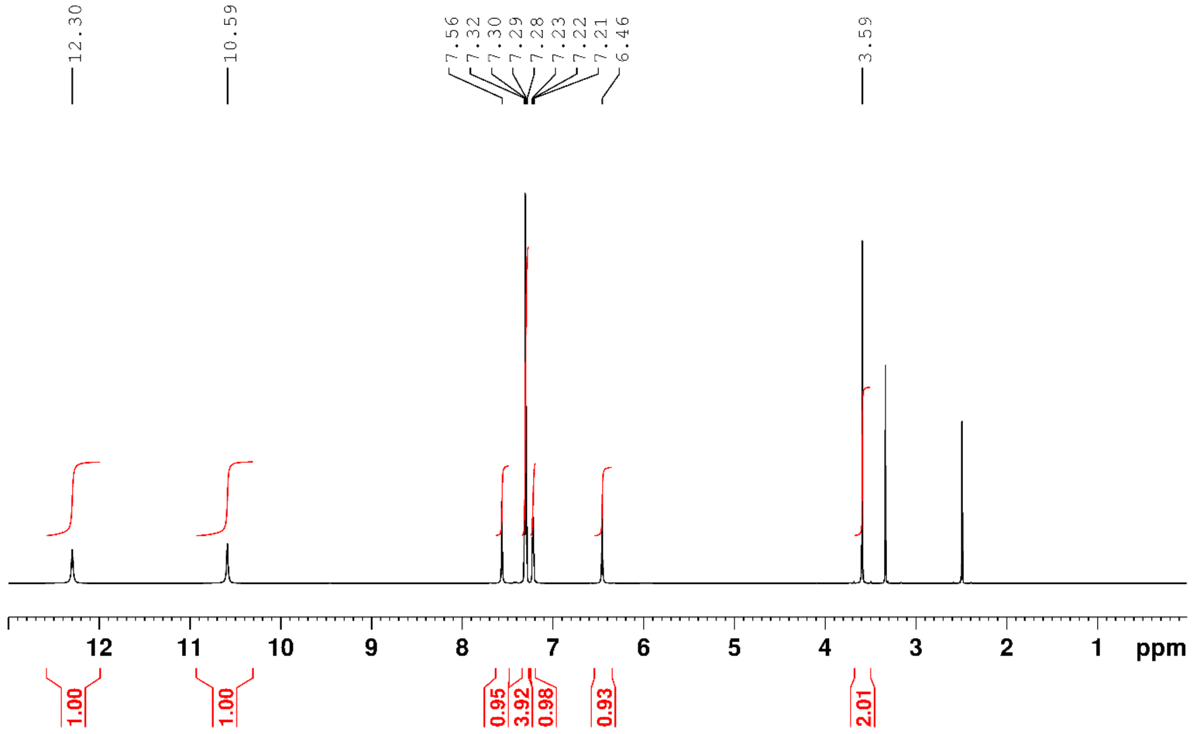

^{1}H NMR spectrum of **10** in DMSO-d$_6$

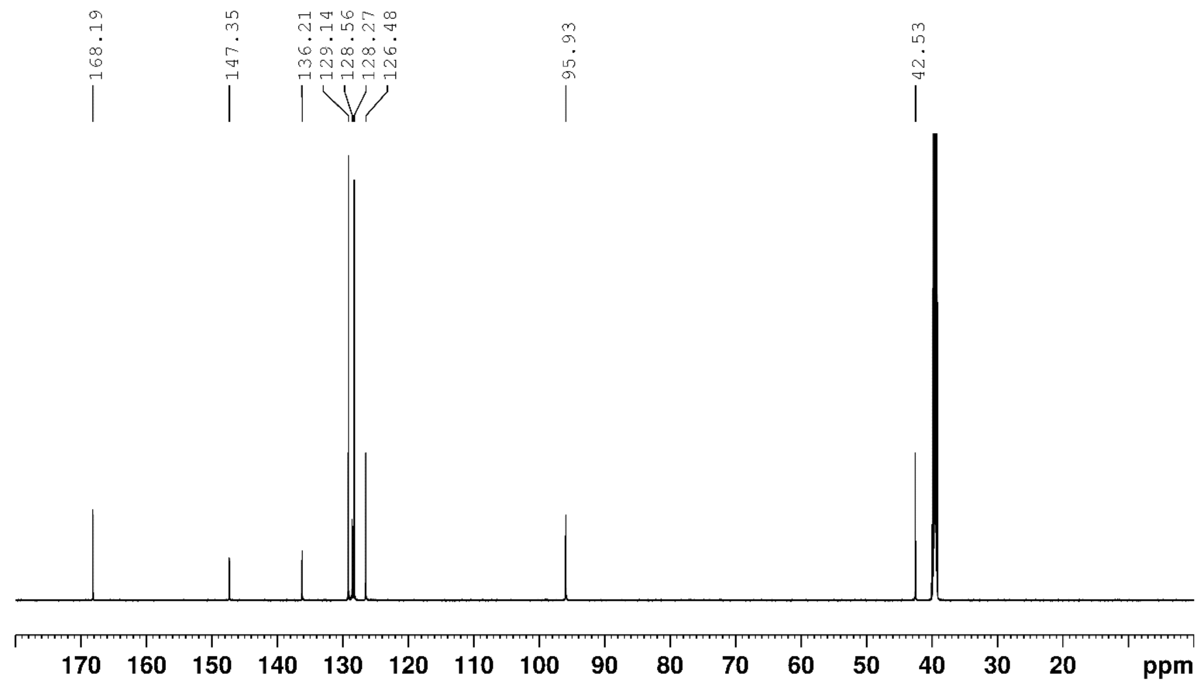

^{13}C NMR spectrum of **10** in DMSO-d$_6$

N-Phenylpicolinamide (11)

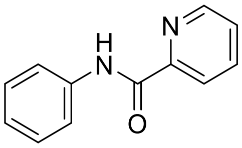

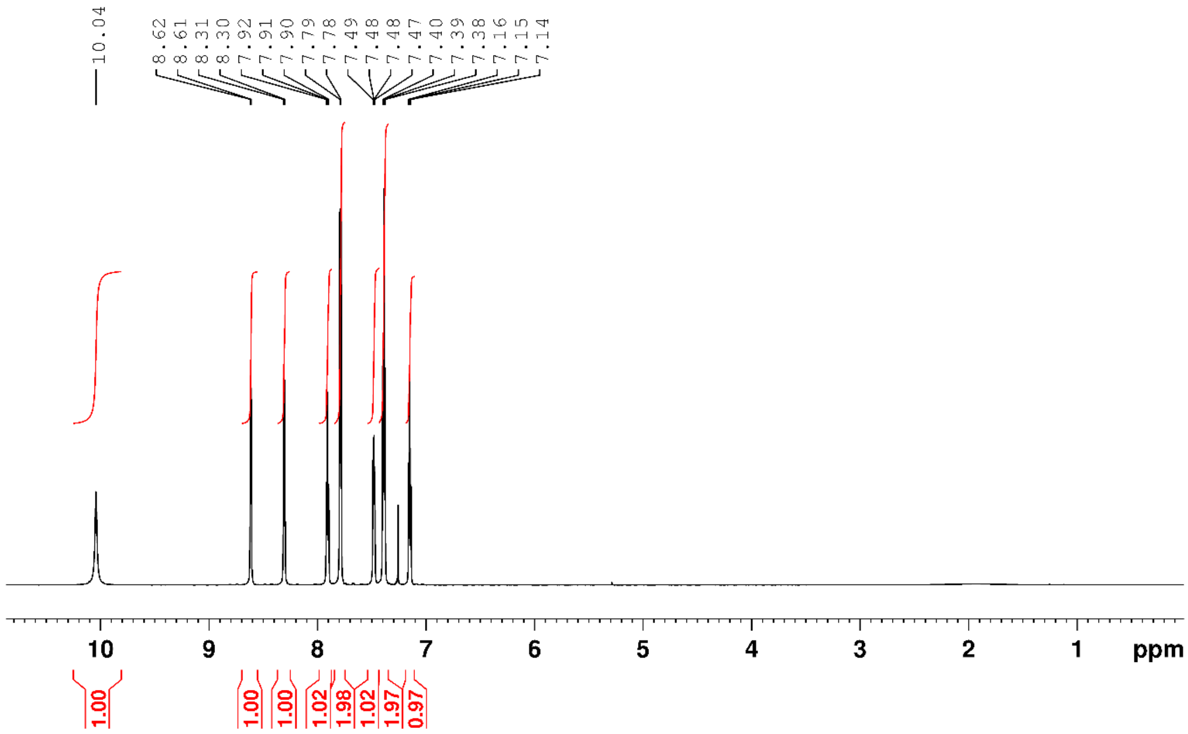

^{1}H NMR spectrum of **11** in $CDCl_3$

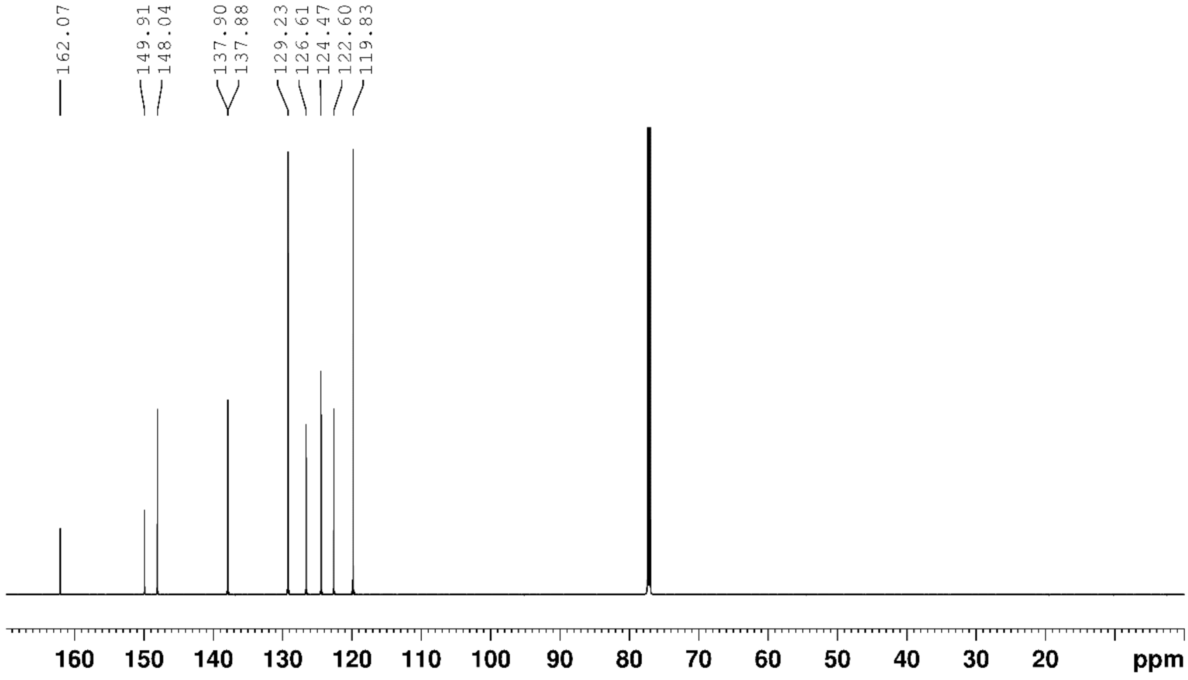

^{13}C NMR spectrum of **11** in $CDCl_3$

N-(3-Bromophenyl)-1H-pyrazole-3-carboxamide (12)

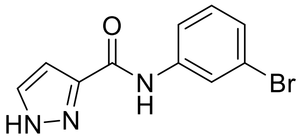

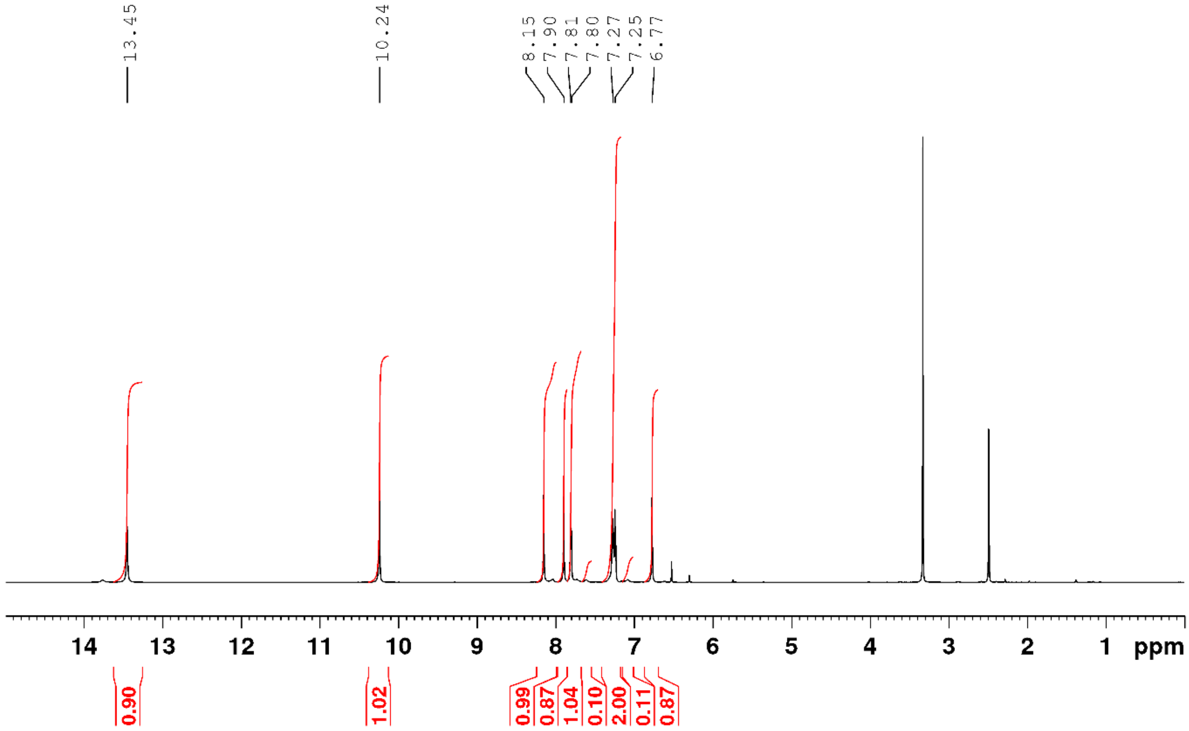

^{1}H NMR spectrum of **12** in DMSO-d$_6$

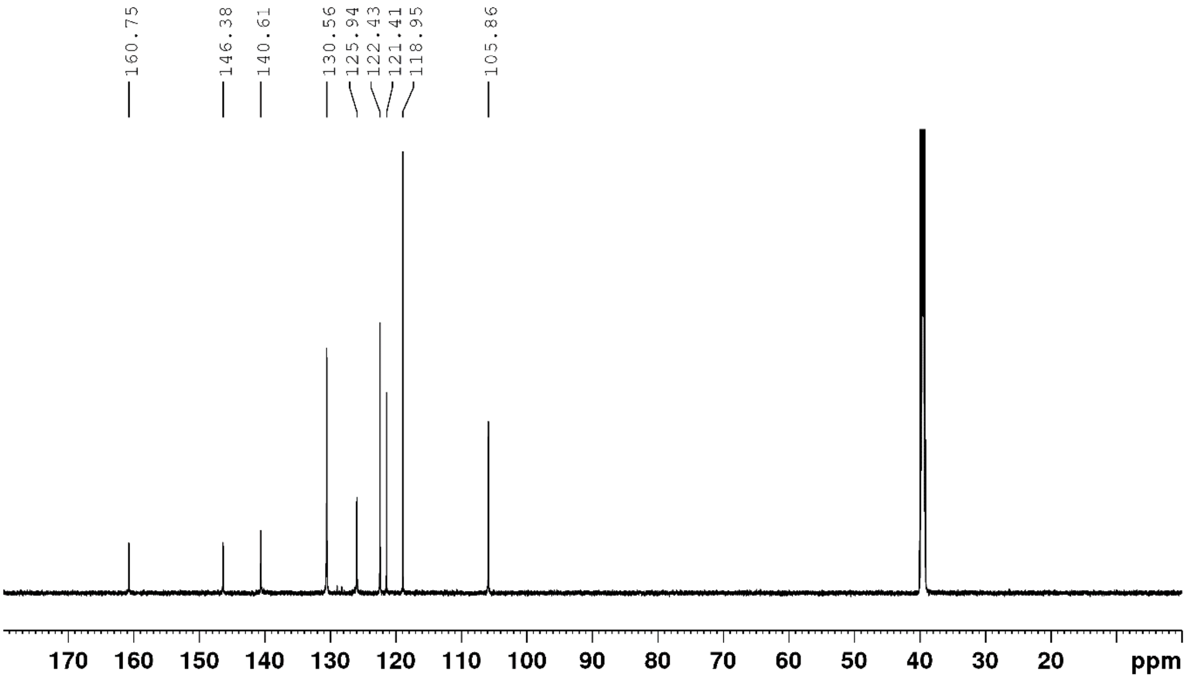

^{13}C NMR spectrum of **12** in DMSO-d$_6$.

N-Benzylpicolinamide (13)

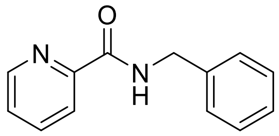

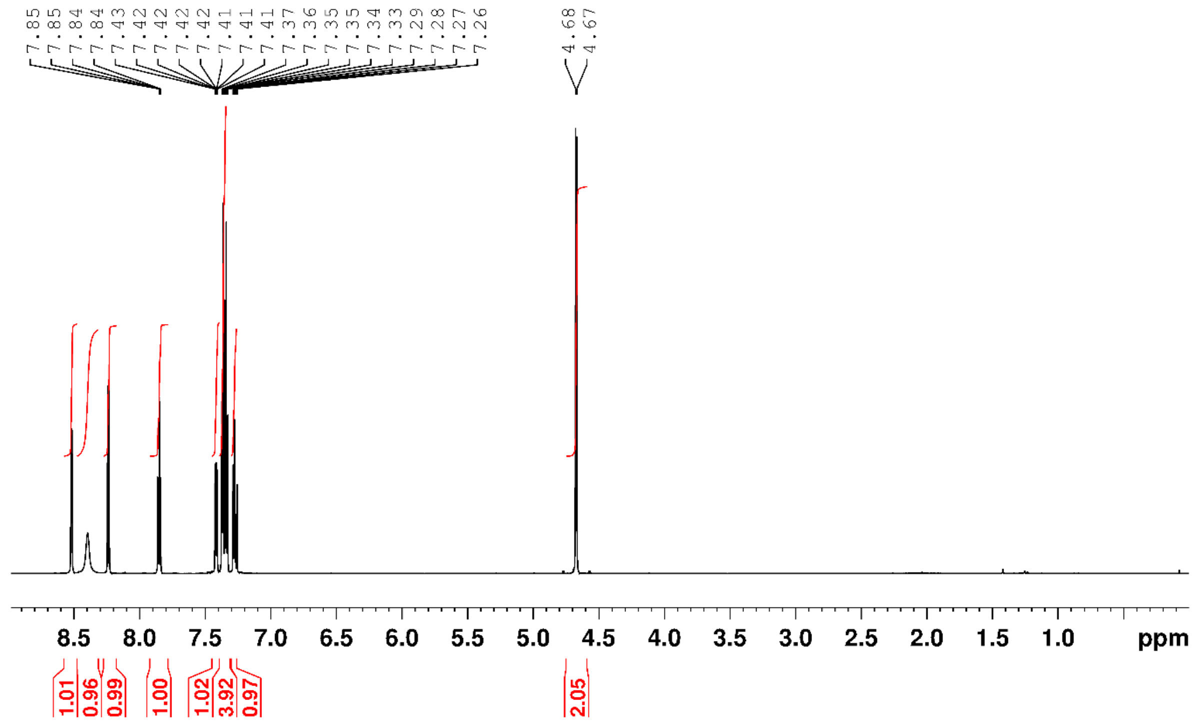

^{1}H NMR spectrum of **13** in $CDCl_3$

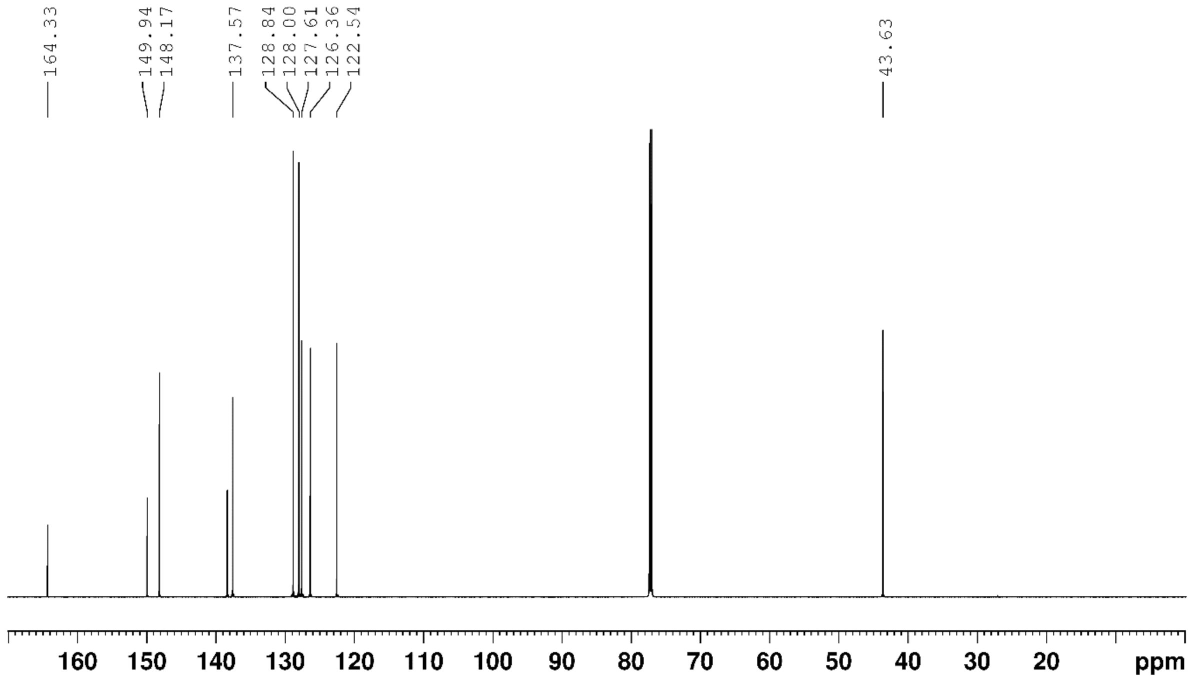

^{13}C NMR spectrum of **13** in $CDCl_3$

_N_-Benzyl-2-(2-chlorophenyl)-2-hydroxyacetamide (14)

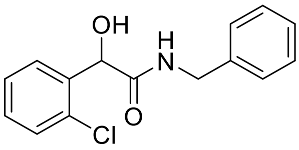

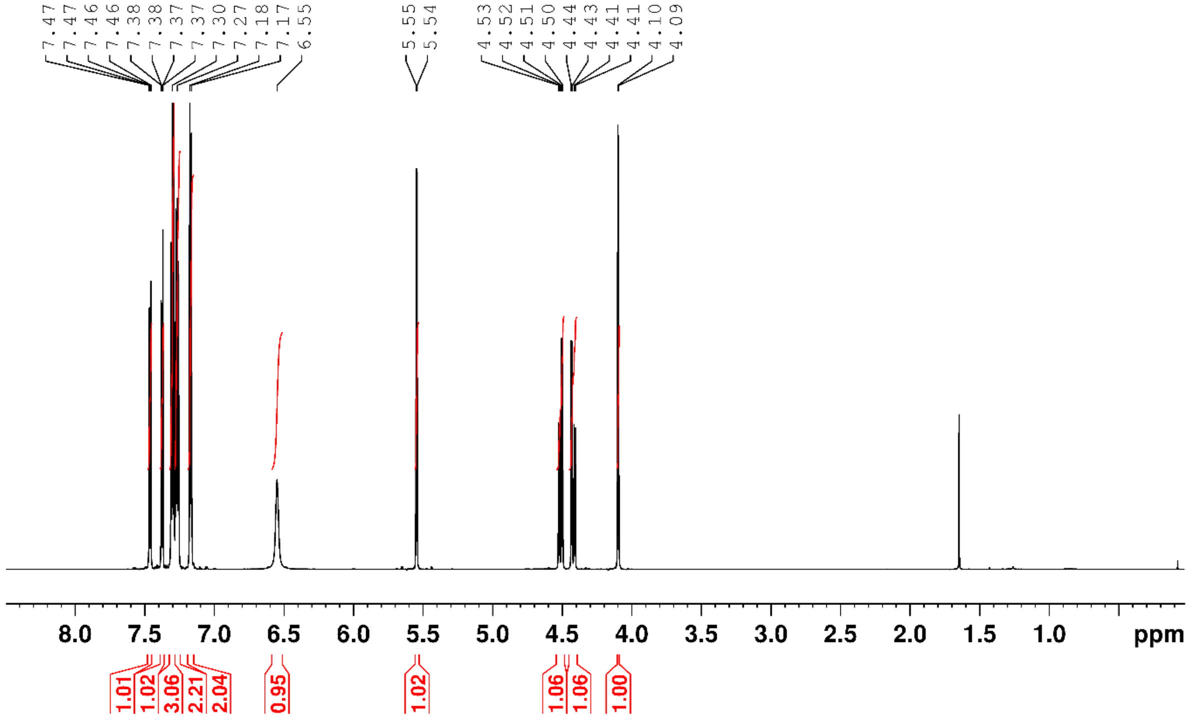

^{1}H NMR spectrum of **14** in $CDCl_3$

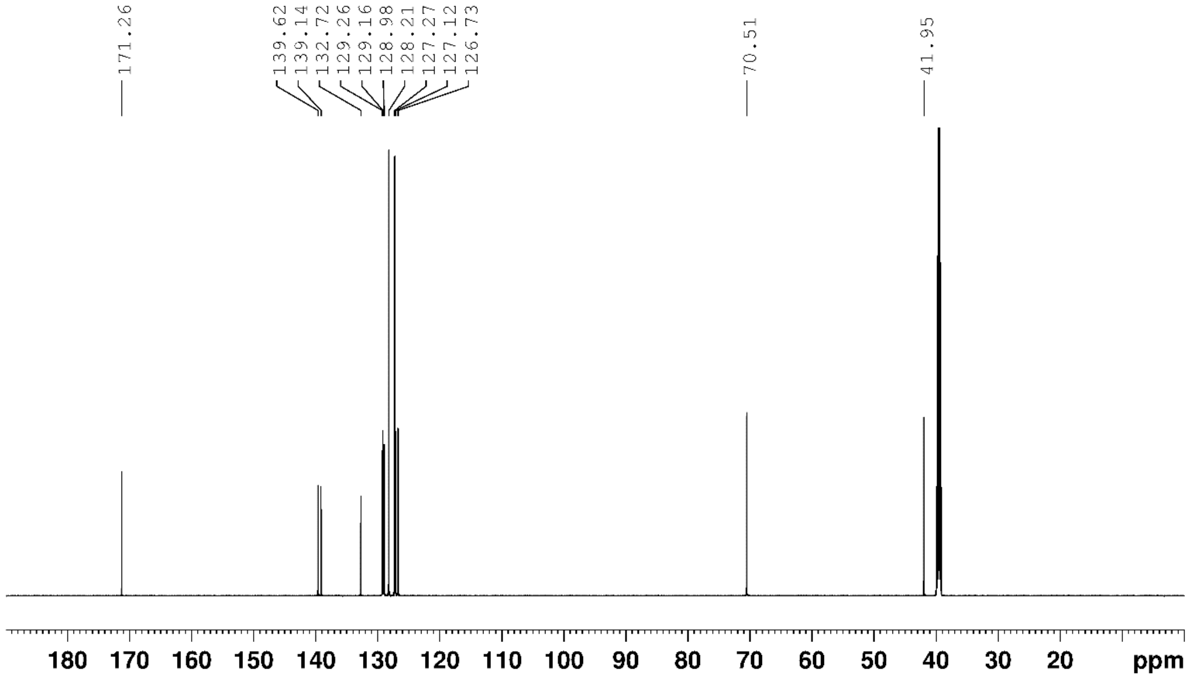

^{13}C NMR spectrum of **14** in DMSO-d_6.

N-Cyclohexylpicolinamide (15)

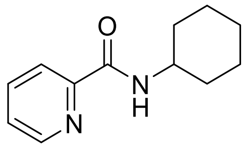

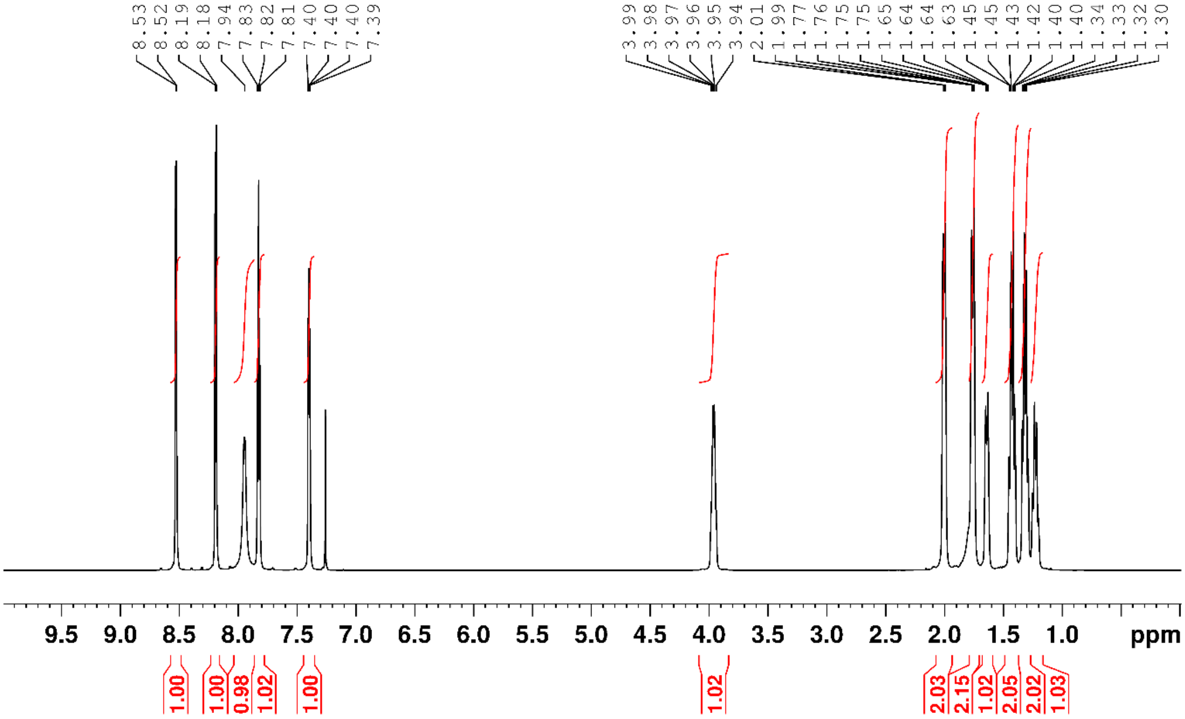

^{1}H NMR spectrum of **15** in $CDCl_3$

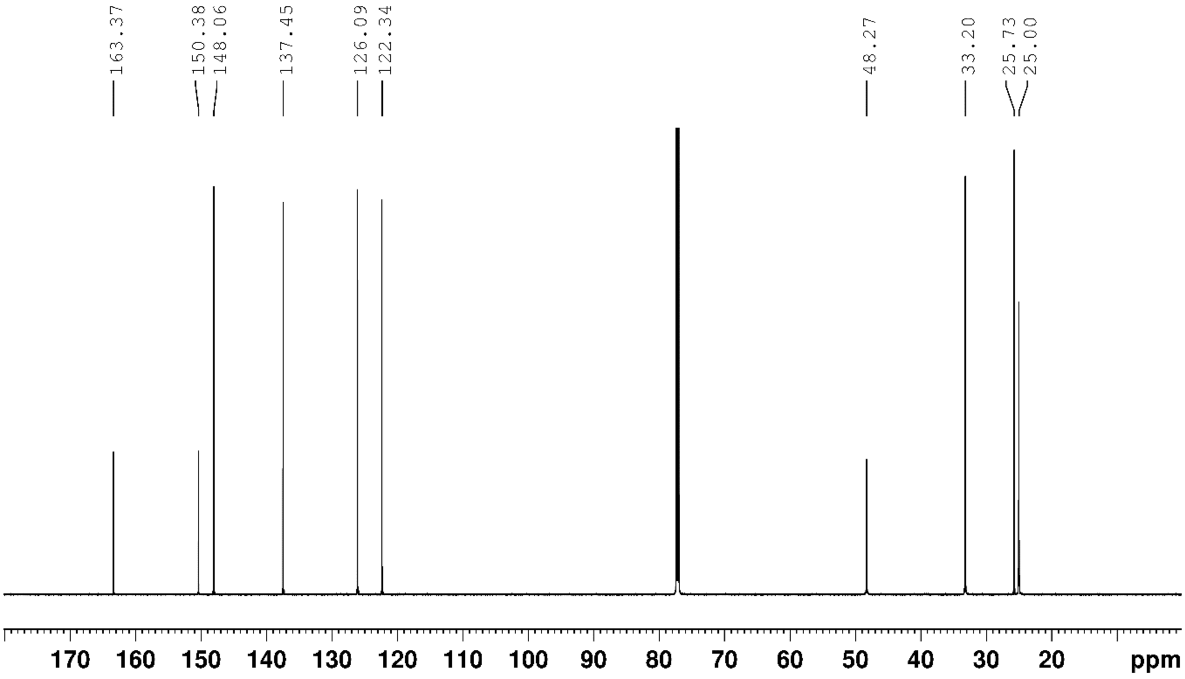

^{13}C NMR spectrum of **15** in $CDCl_3$.

***N*-Benzyl-1H-pyrazole-3-carboxamide (16)**

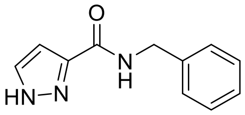

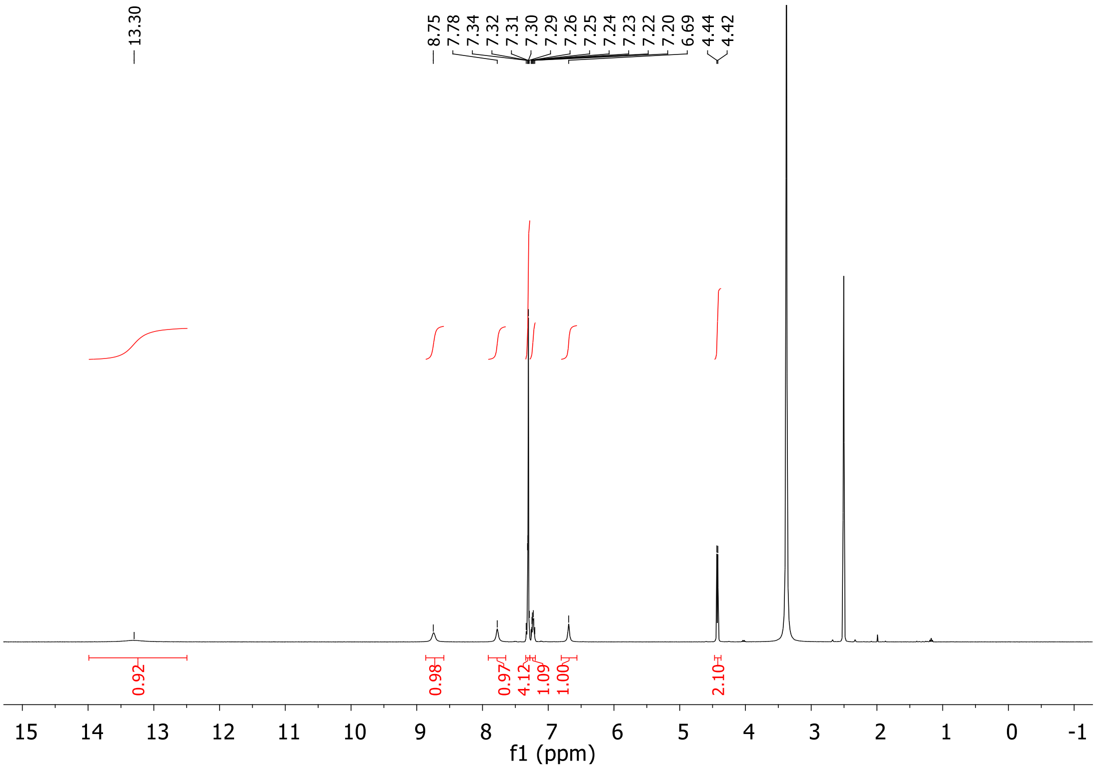

^{1}H NMR spectrum of **16** in DMSO.

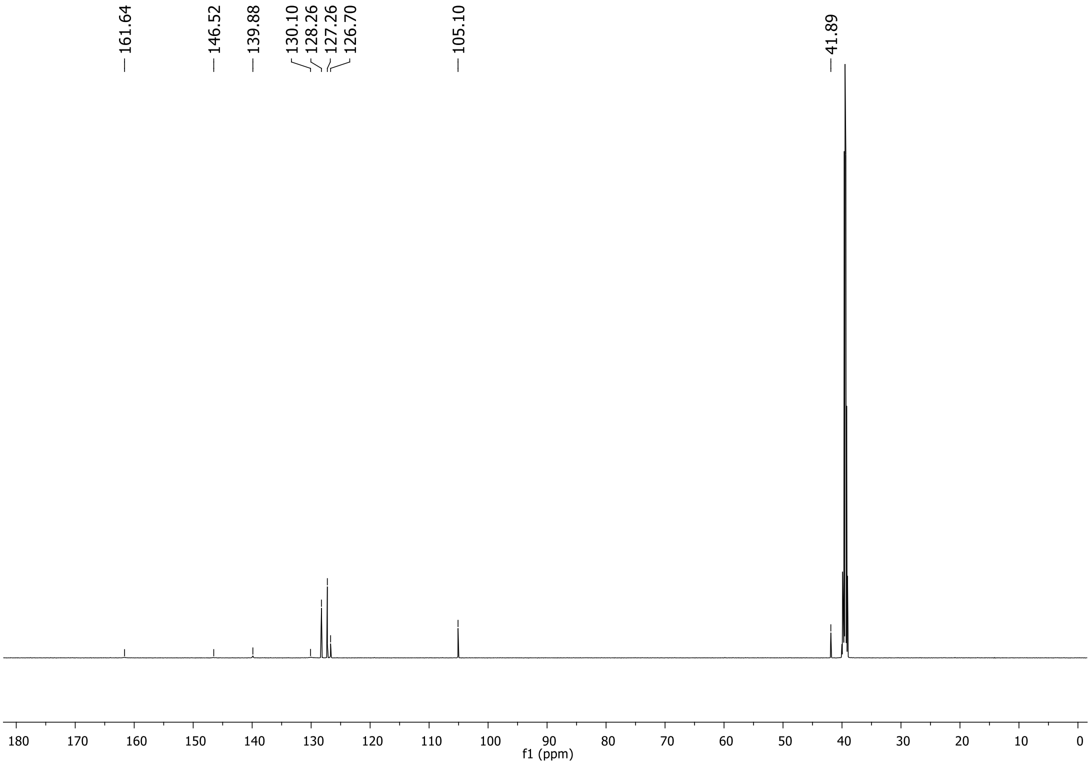

^{13}C NMR spectrum of **16** in DMSO.

_N_-(2-(Dimethylamino)phenyl)-2-phenylacetamide (17)

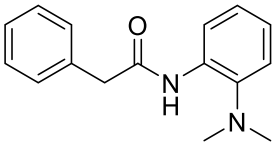

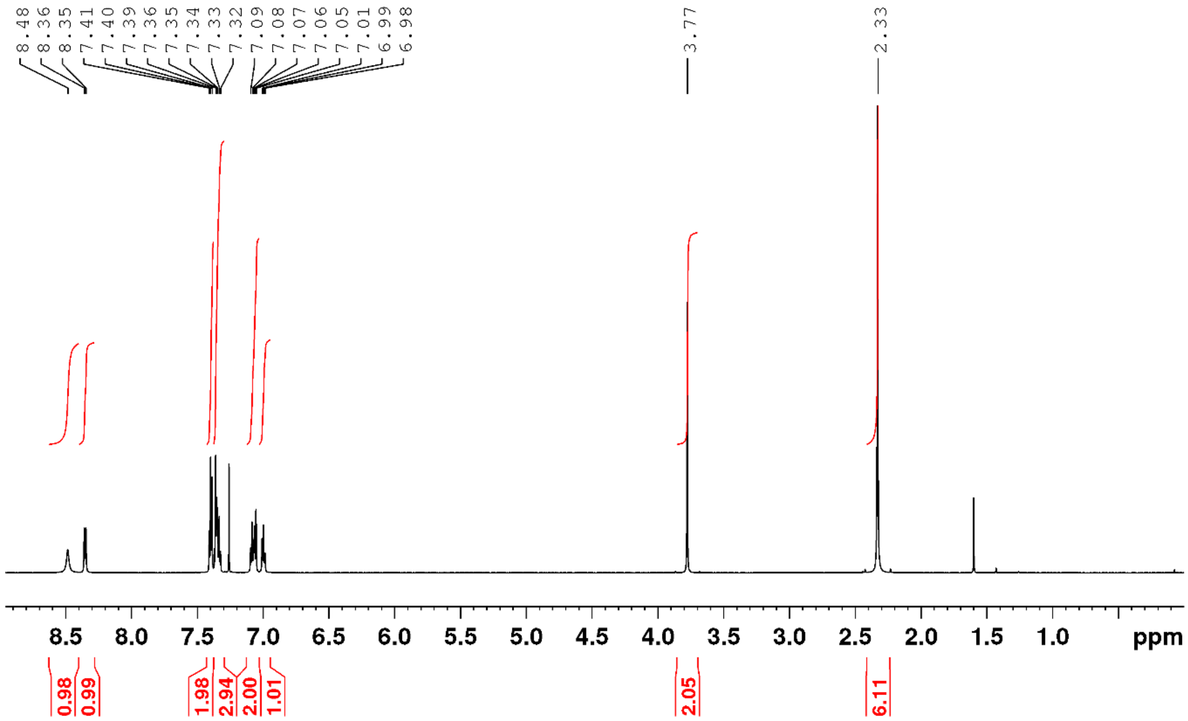

^{1}H NMR spectrum of **17** in $CDCl_3$

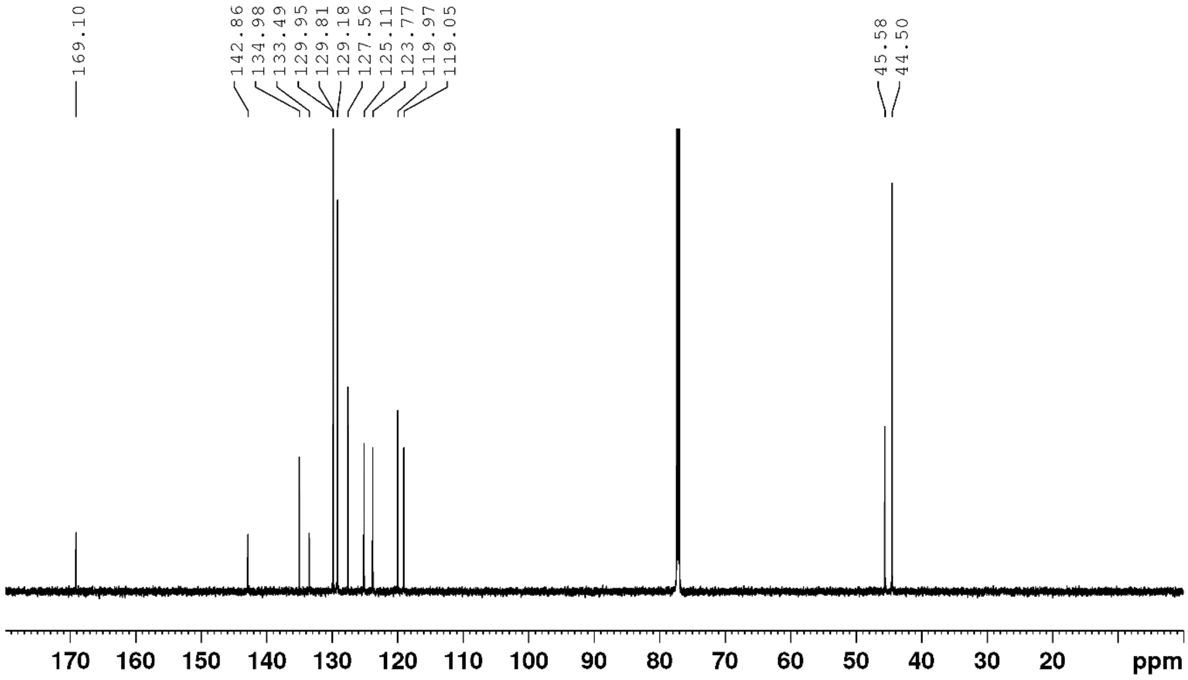

^{13}C NMR spectrum of **17** in $CDCl_3$

2-(3-Fluorophenyl)-*N*-(pyridin-3-yl)acetamide (18)

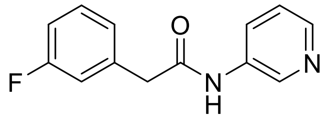

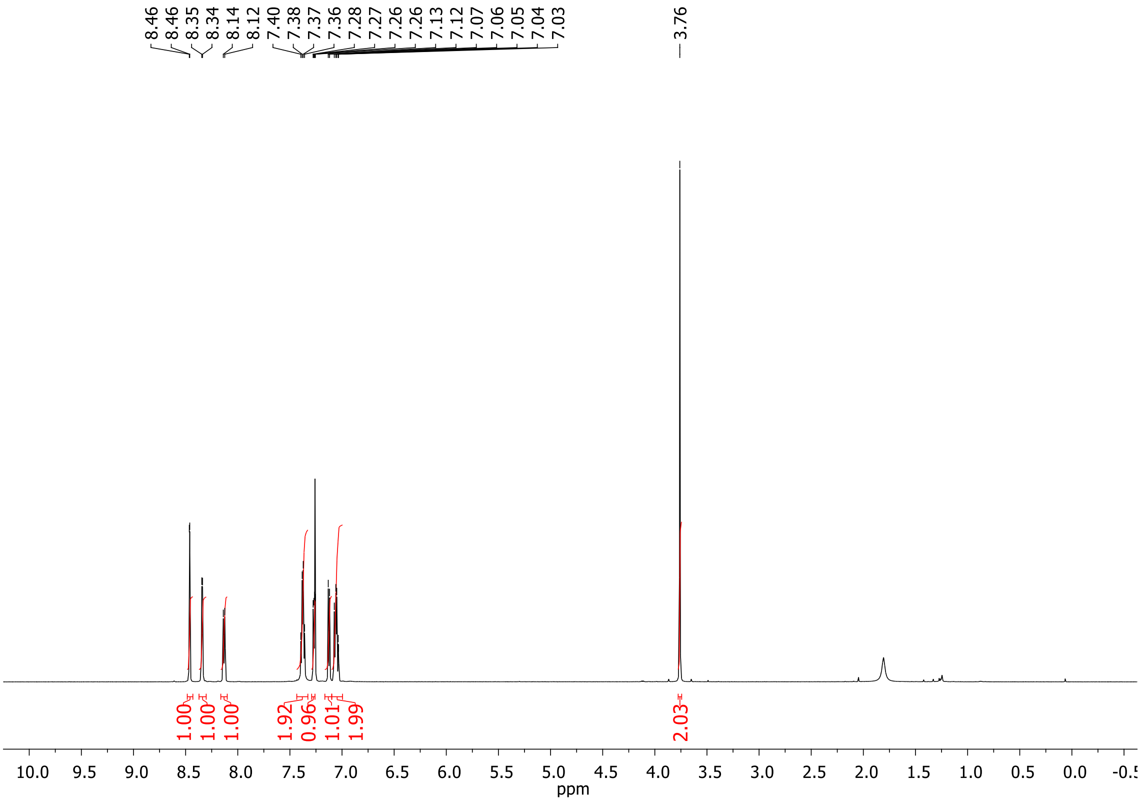

^{1}H NMR spectrum of **18** in $CDCl_3$

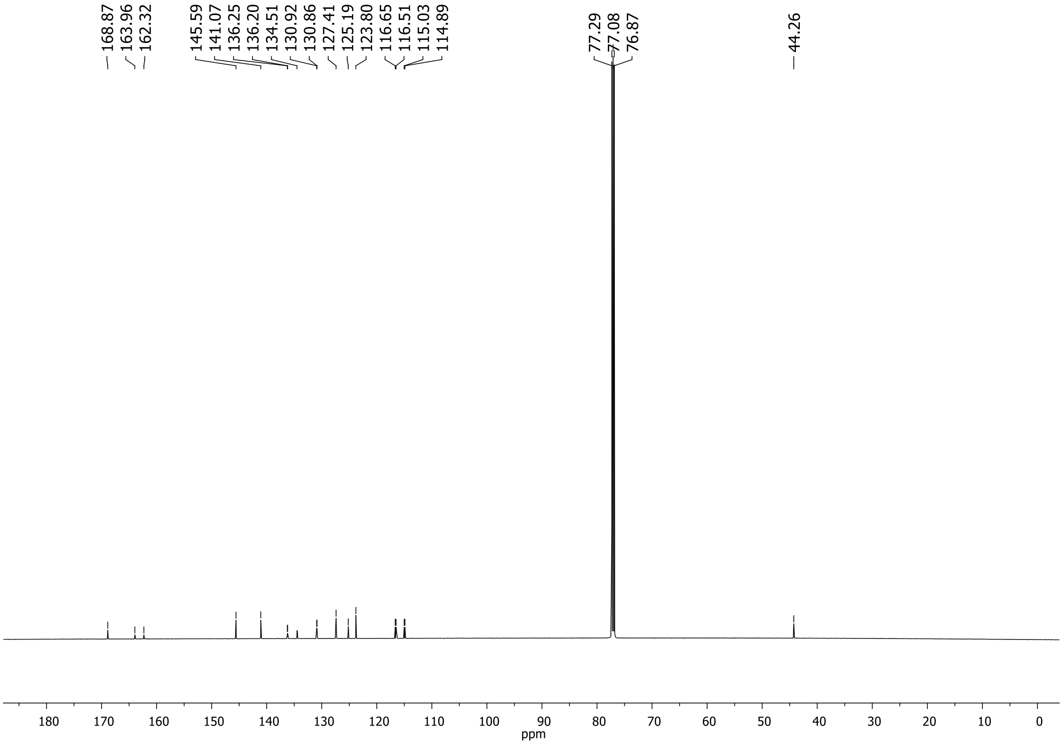

^{13}C NMR spectrum of **18** in $CDCl_3$.

tert-Butyl 4-(pyridin-3-ylcarbamoyl)piperidine-1-carboxylate (19)

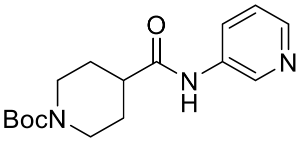

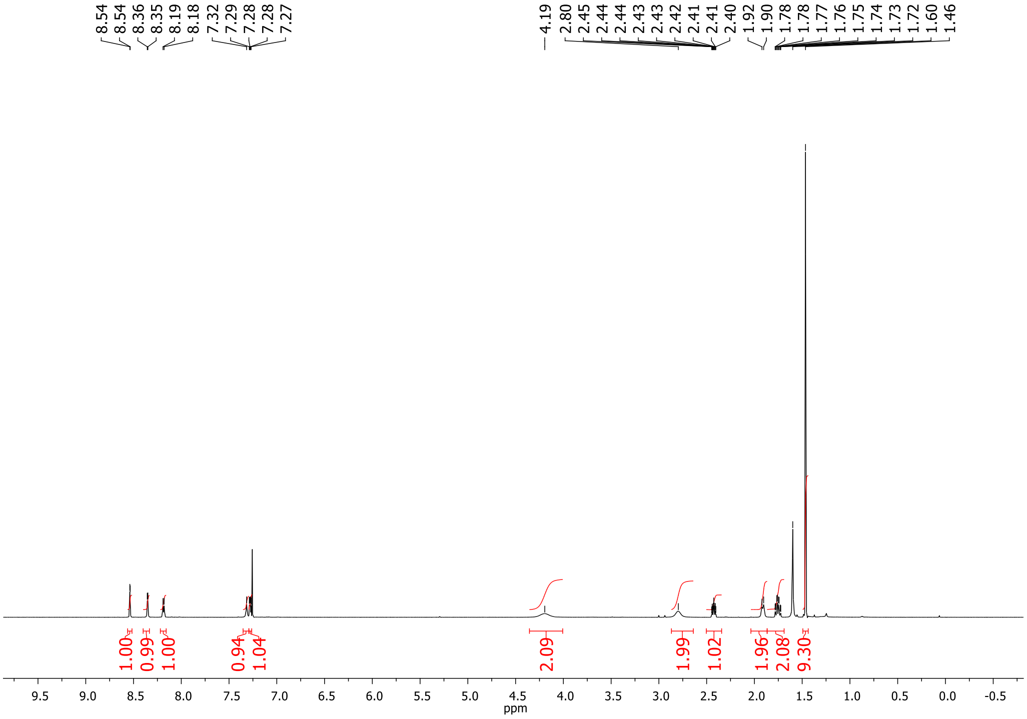

^{1}H NMR spectrum of **19** in $CDCl_3$

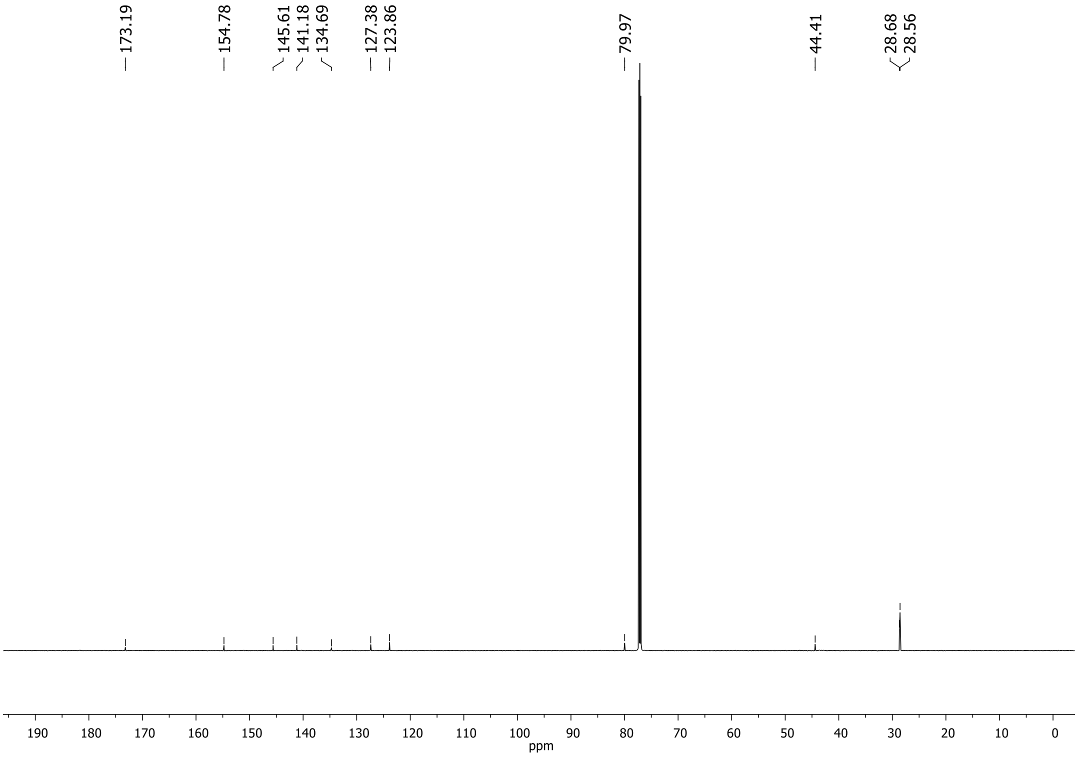

^{13}C NMR spectrum of **19** in $CDCl_3$.

_N_-(3,5-Bis-(trifluoromethyl)phenyl)-5-bromofuran-2-carboxamide (2)

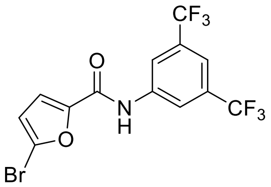

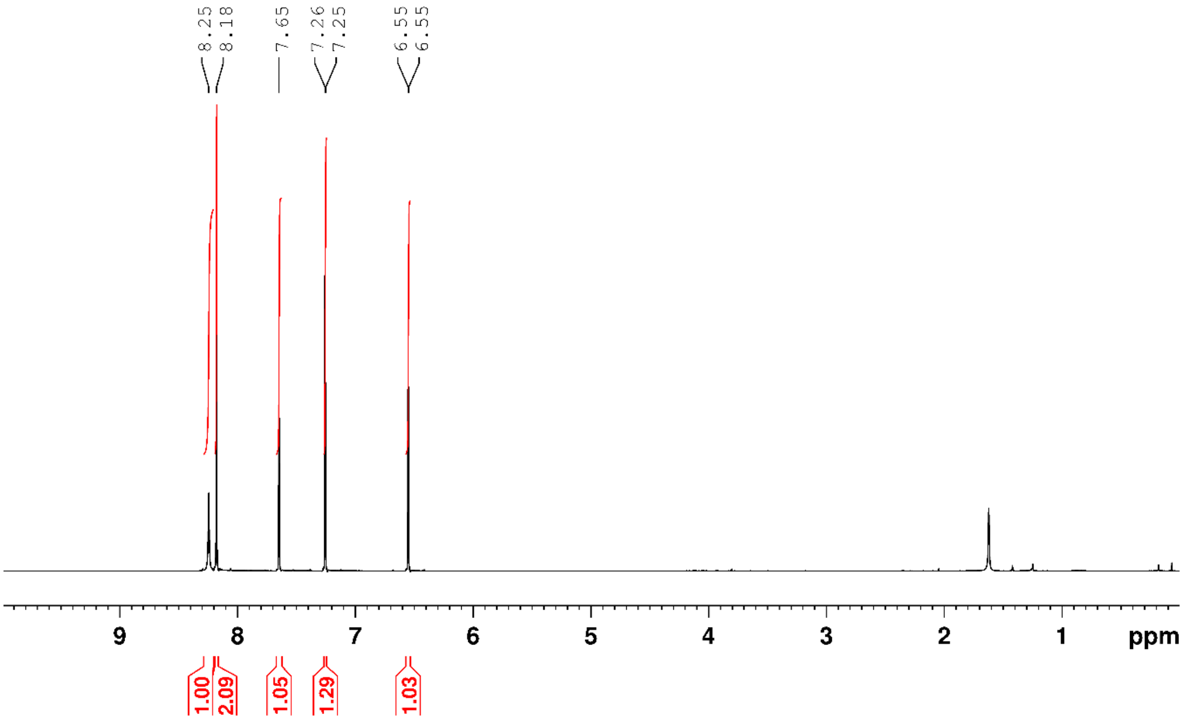

^{1}H NMR spectrum of **20** in $CDCl_3$

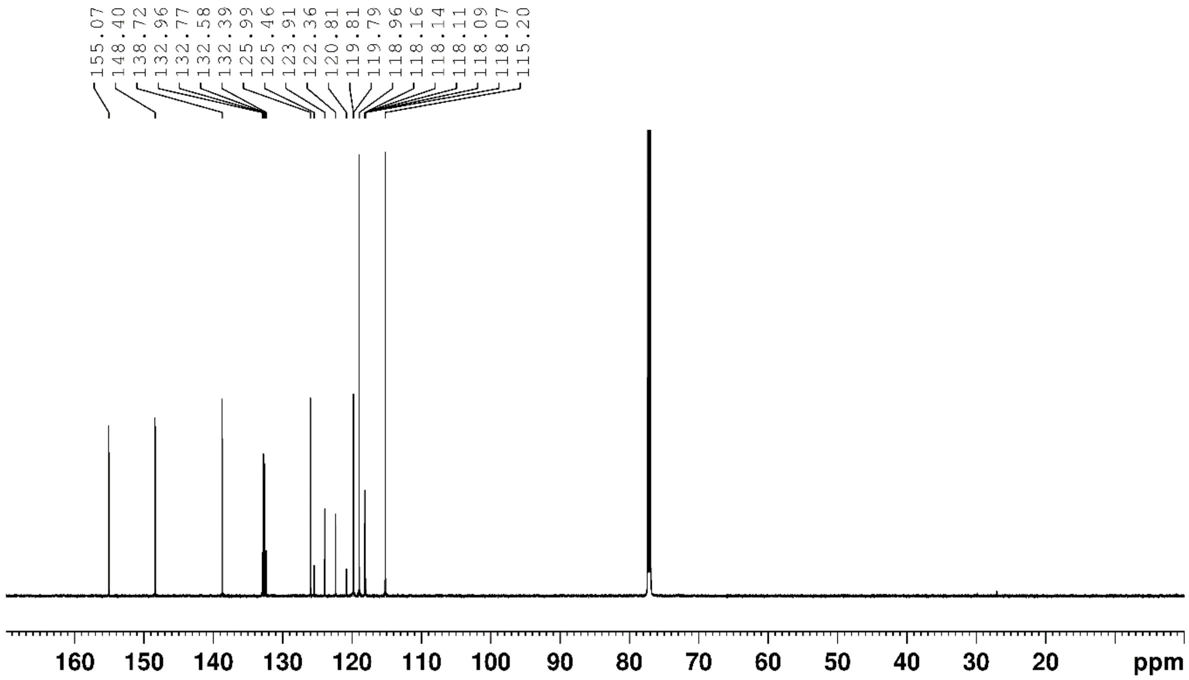

^{13}C NMR spectrum of **20** in $CDCl_3$

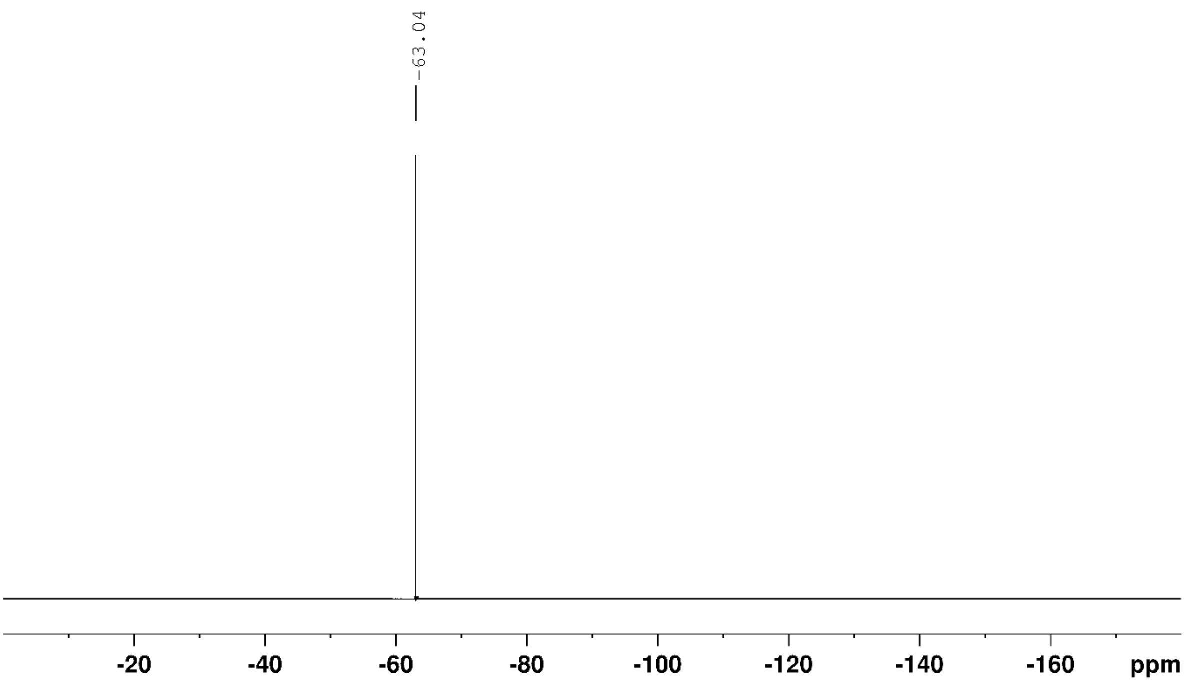

^{19}F NMR spectrum of **20** in $CDCl_3$

N-(3-Bromophenyl)benzamide (21)

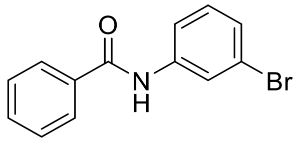

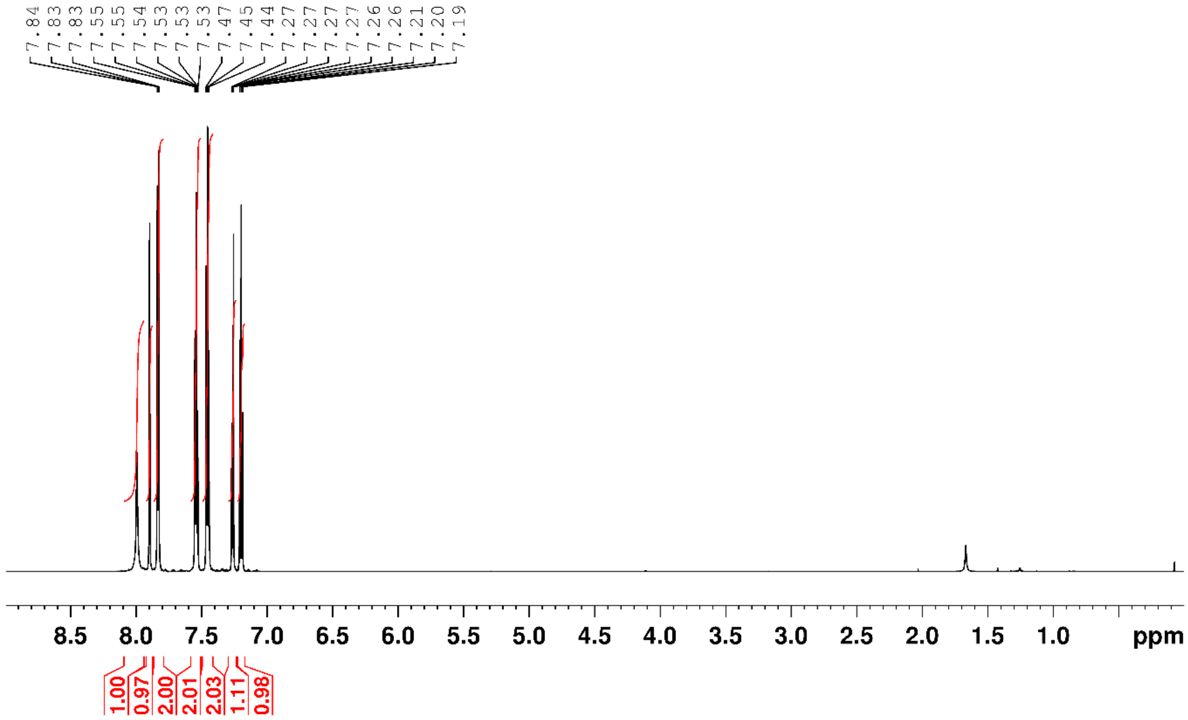

^{1}H NMR spectrum of **21** in $CDCl_3$

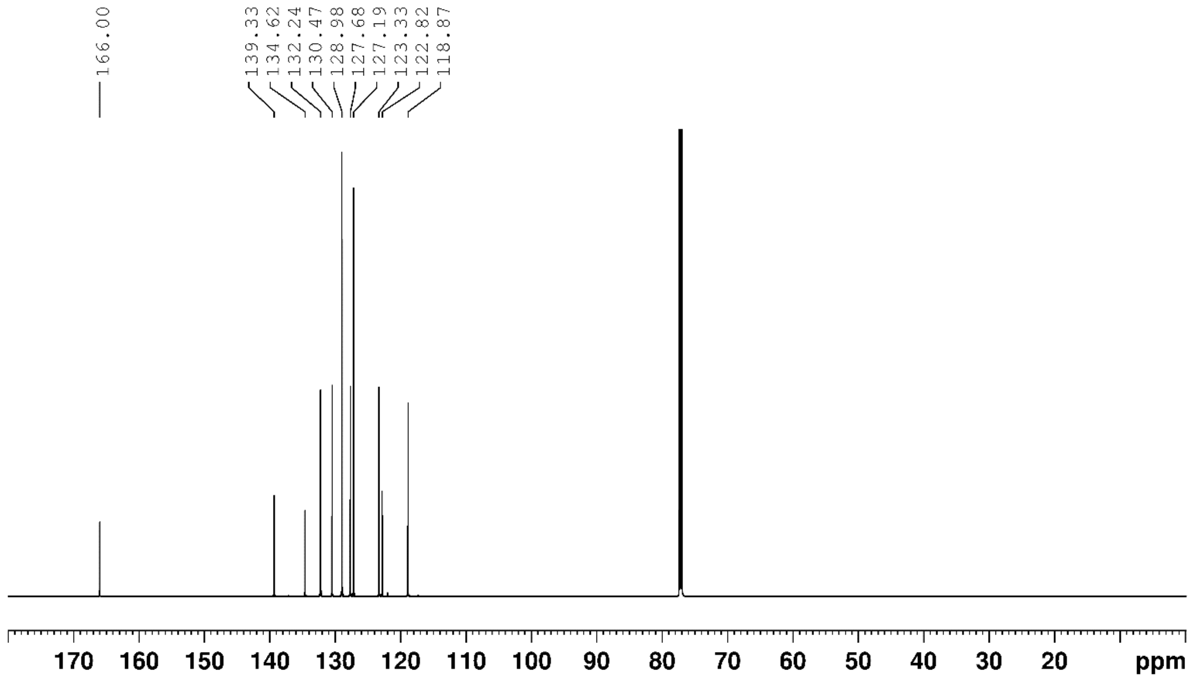

^{13}C NMR spectrum of **21** in $CDCl_3$

_N_-(3,5-Bis-(trifluoromethyl)phenyl)-2-phenylacetamide (22)

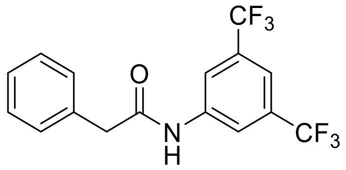

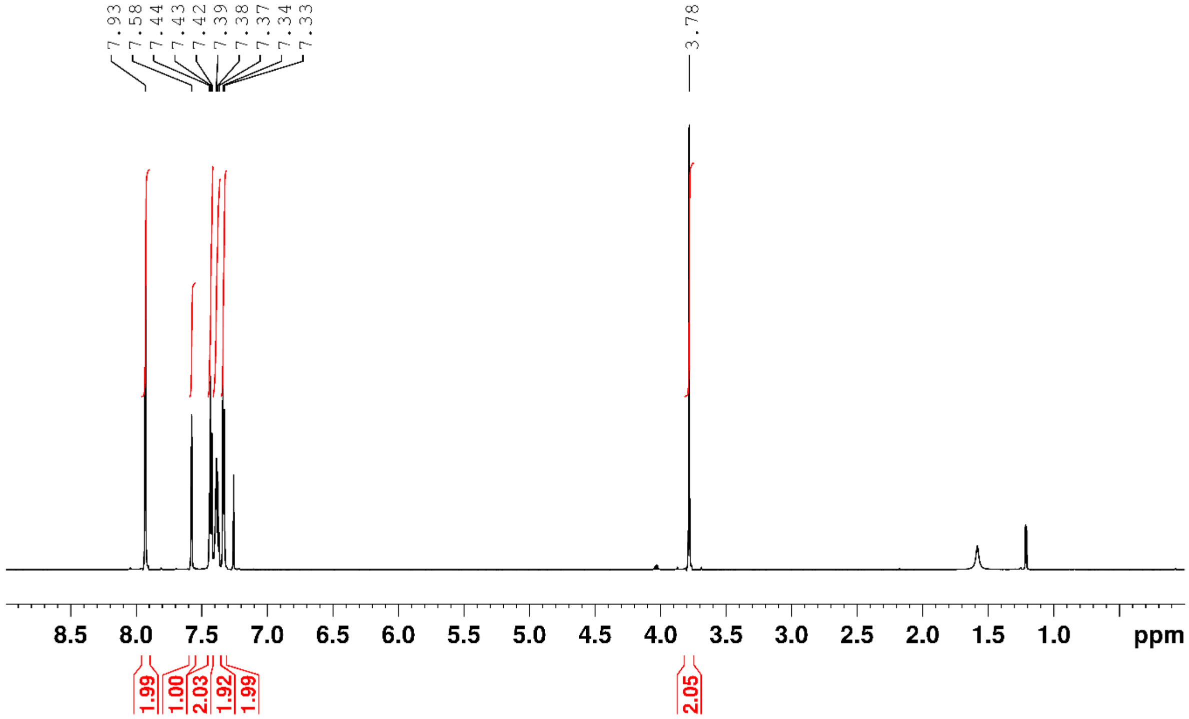

^{1}H NMR spectrum of **22** in $CDCl_3$

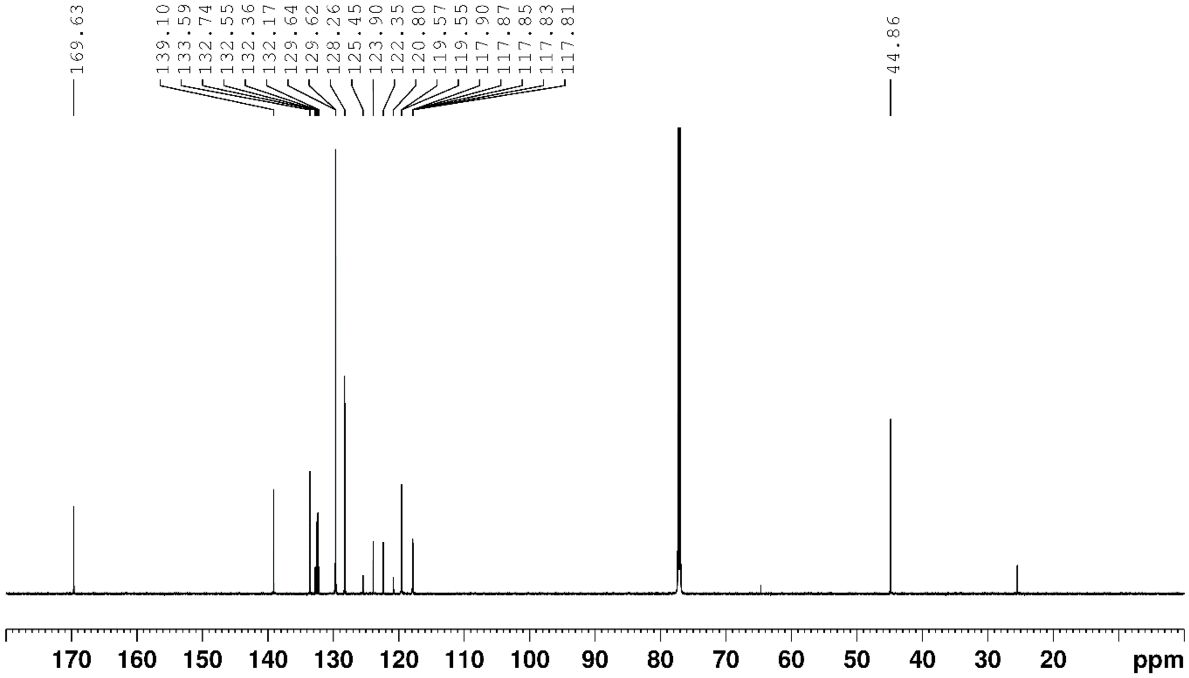

^{13}C NMR spectrum of **22** in $CDCl_3$

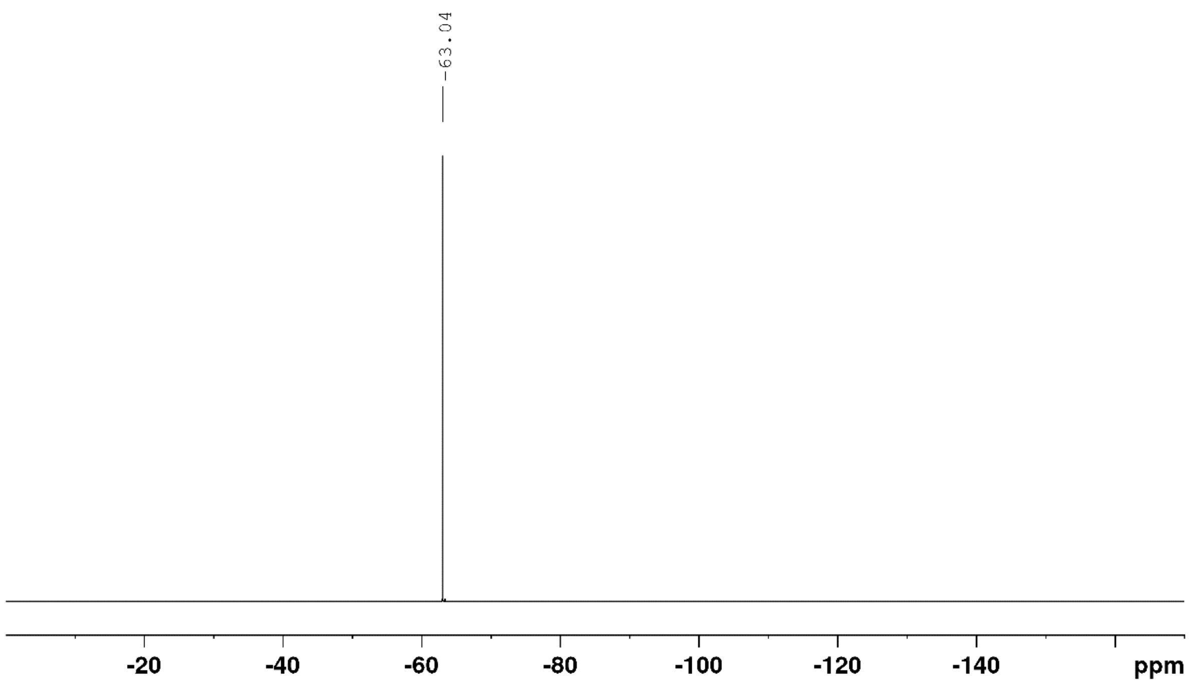

^{19}F NMR spectrum of **22** in $CDCl_3$

_N_-Allyl-_N_-phenyl,2-phenylacetamide (23)

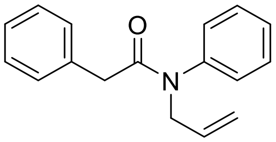

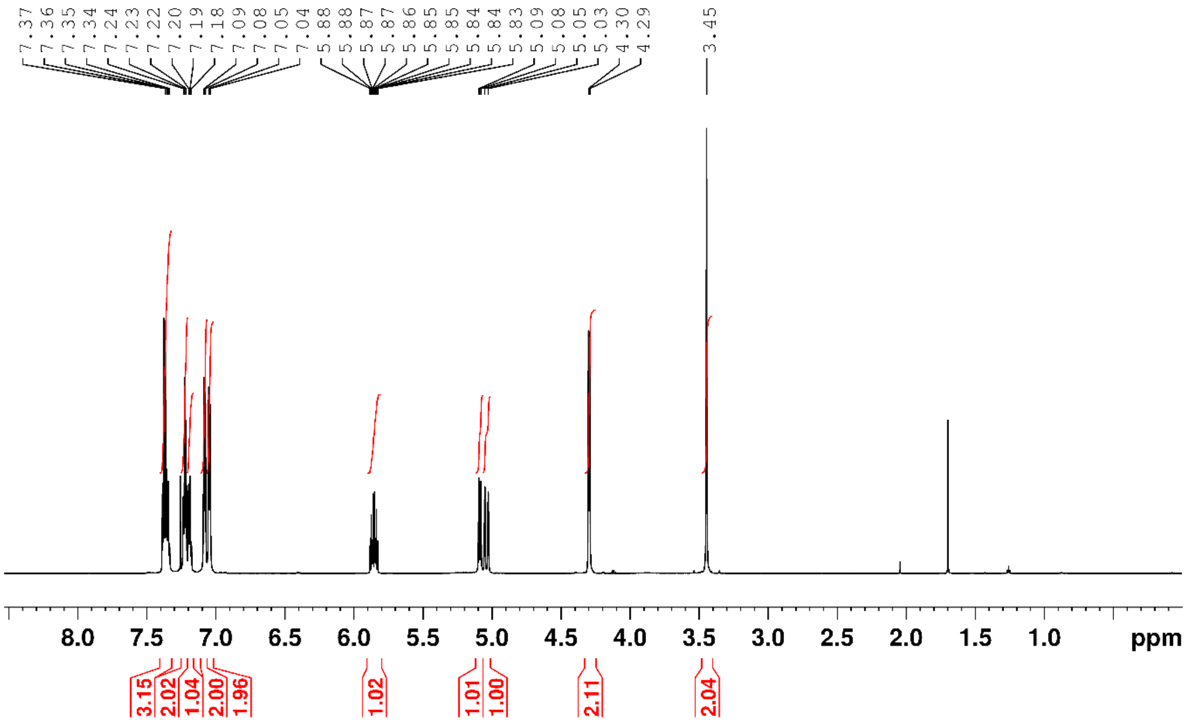

^{1}H NMR spectrum of **23** in $CDCl_3$

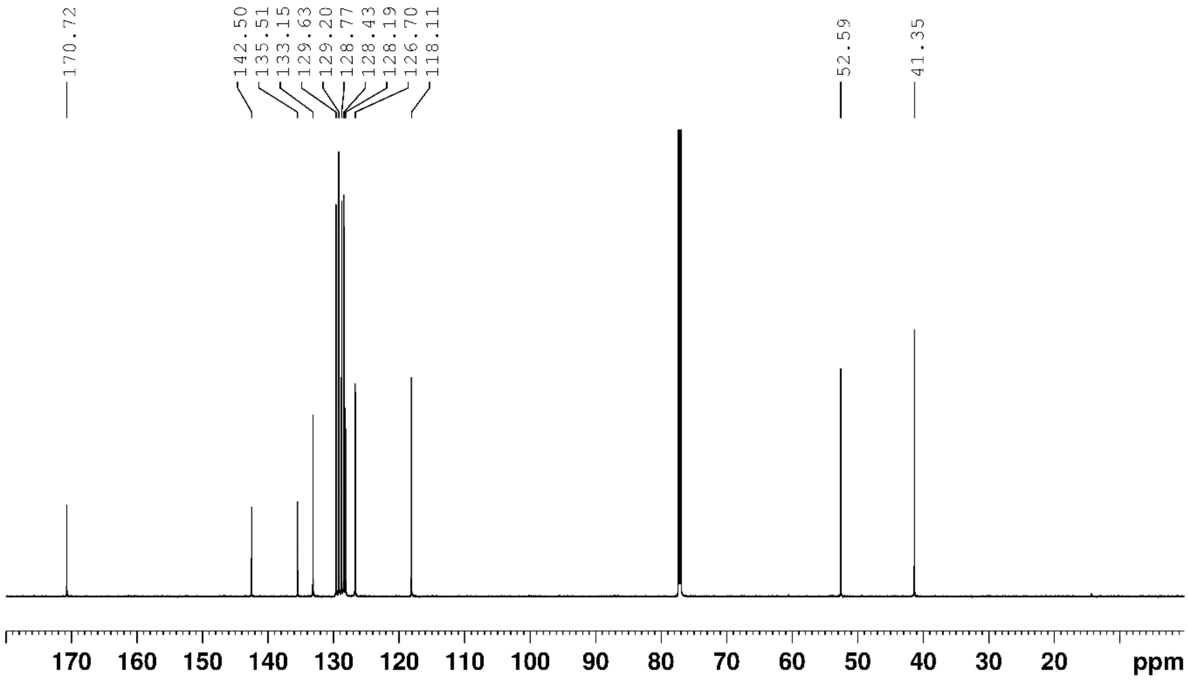

^{13}C NMR spectrum of **23** in $CDCl_3$

***N*-Methyl-*N*-phenylbenzamide (24)**

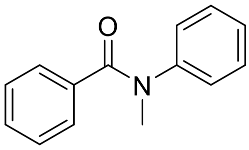

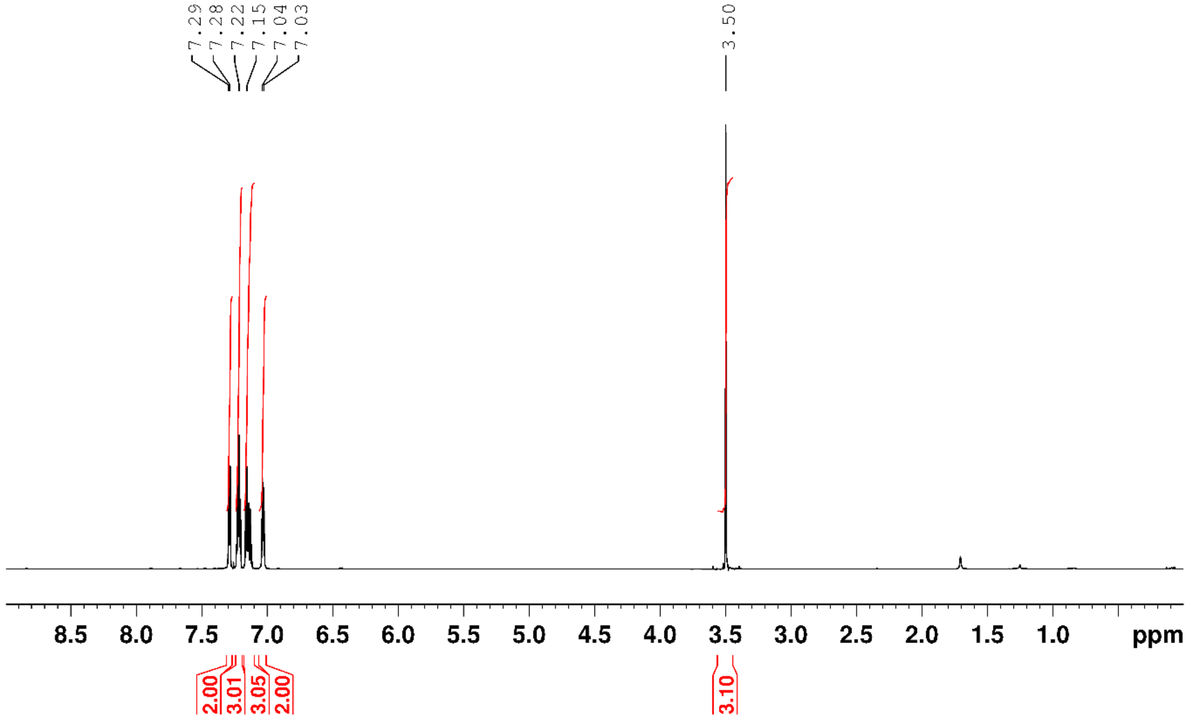

^{1}H NMR spectrum of **24** in $CDCl_3$

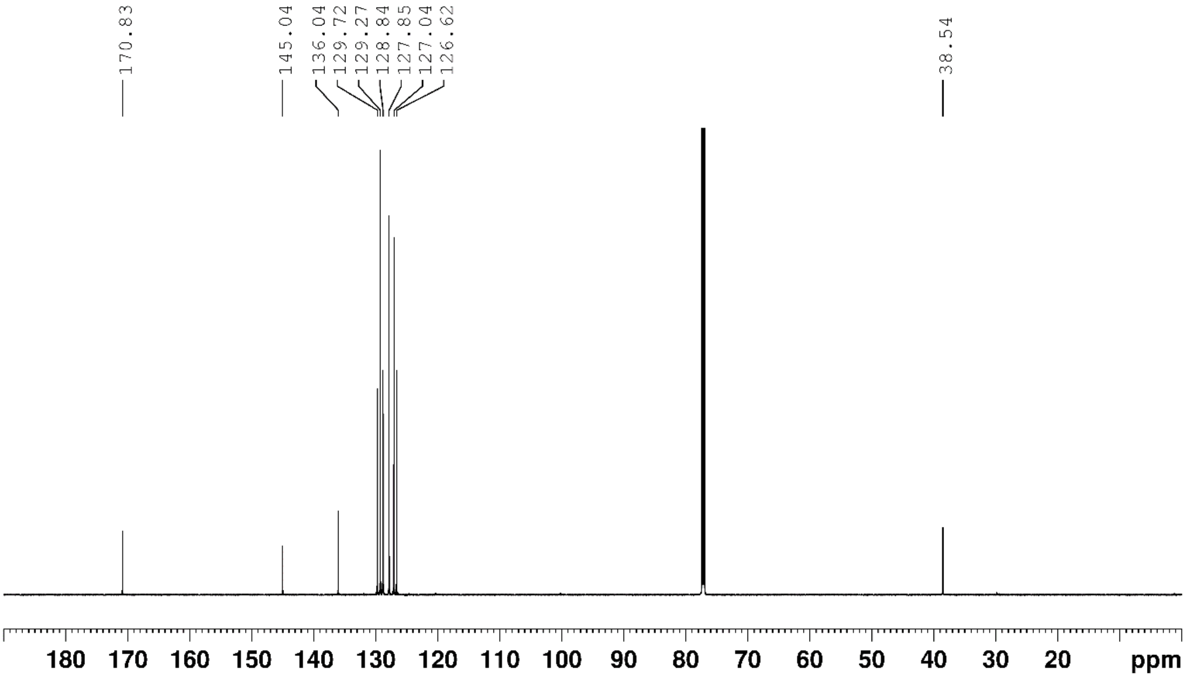

^{13}C NMR spectrum of **24** in $CDCl_3$

N-Cyclohexylcyclopropanecarboxamide (25)

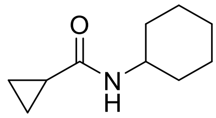

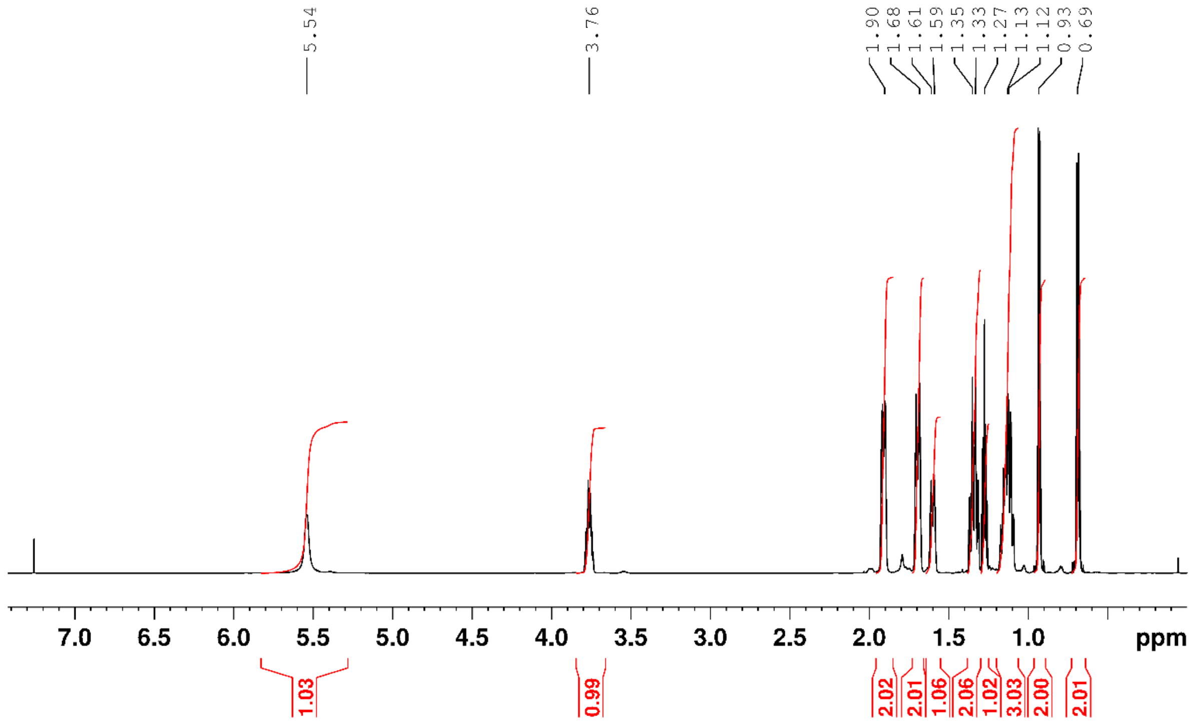

^{1}H NMR spectrum of **25** in $CDCl_3$

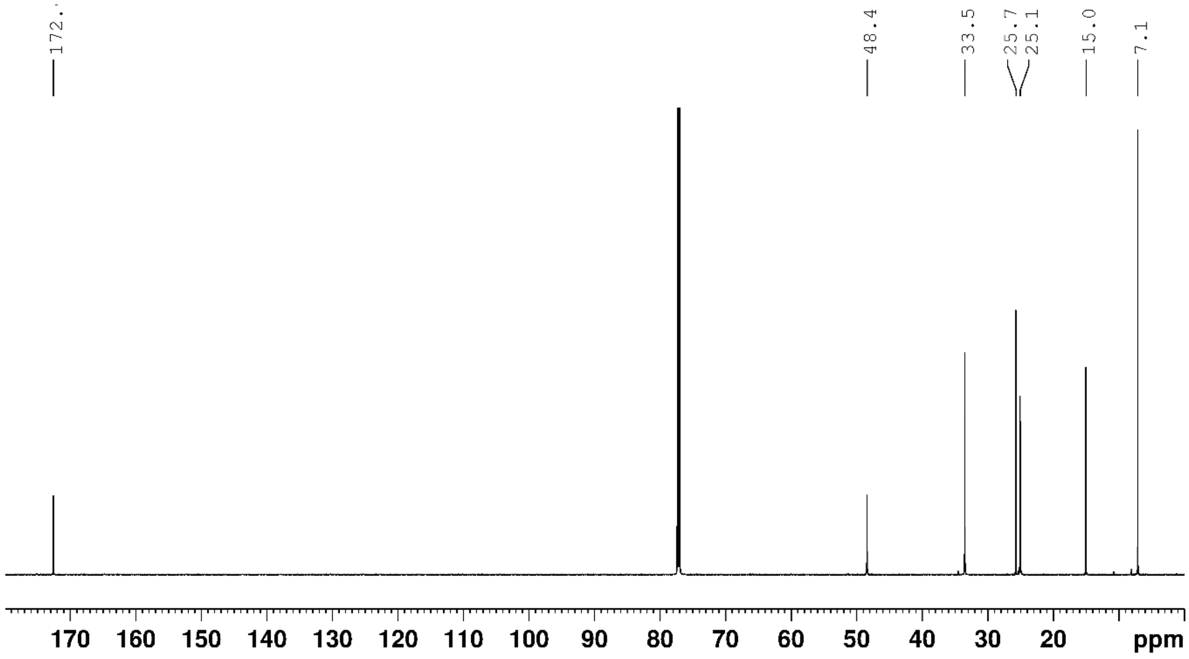

^{13}C NMR spectrum of **25** in $CDCl_3$.

N-(Adamantan-1-yl)-2-phenylacetamide (26)

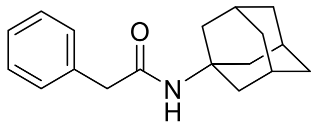

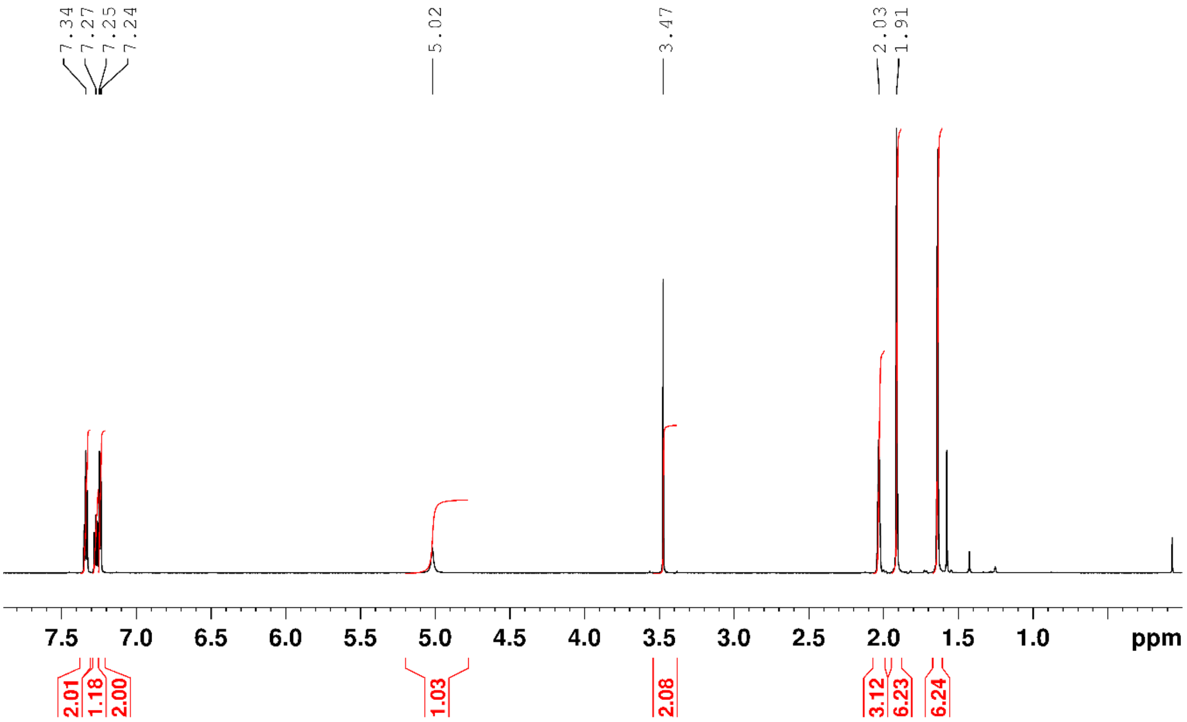

^{1}H NMR spectrum of **26** in $CDCl_3$

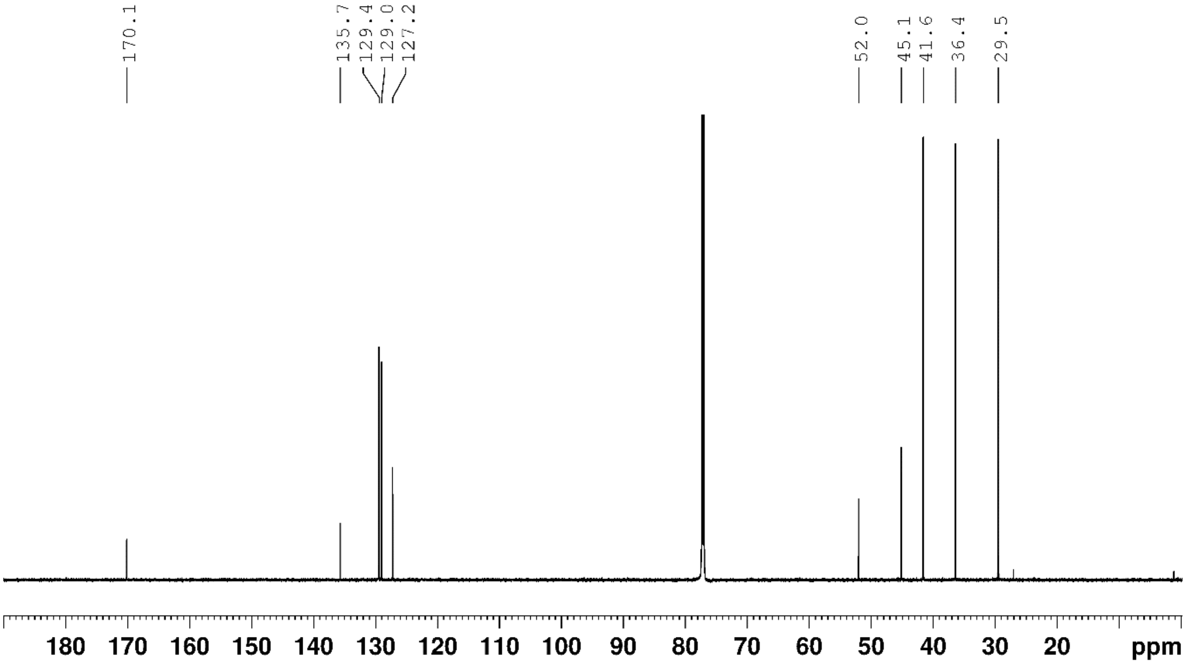

^{13}C NMR spectrum of **26** in $CDCl_3$.

(S)-*N*-(1-Phenylethyl)pivalamide (27)

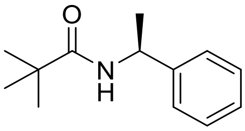

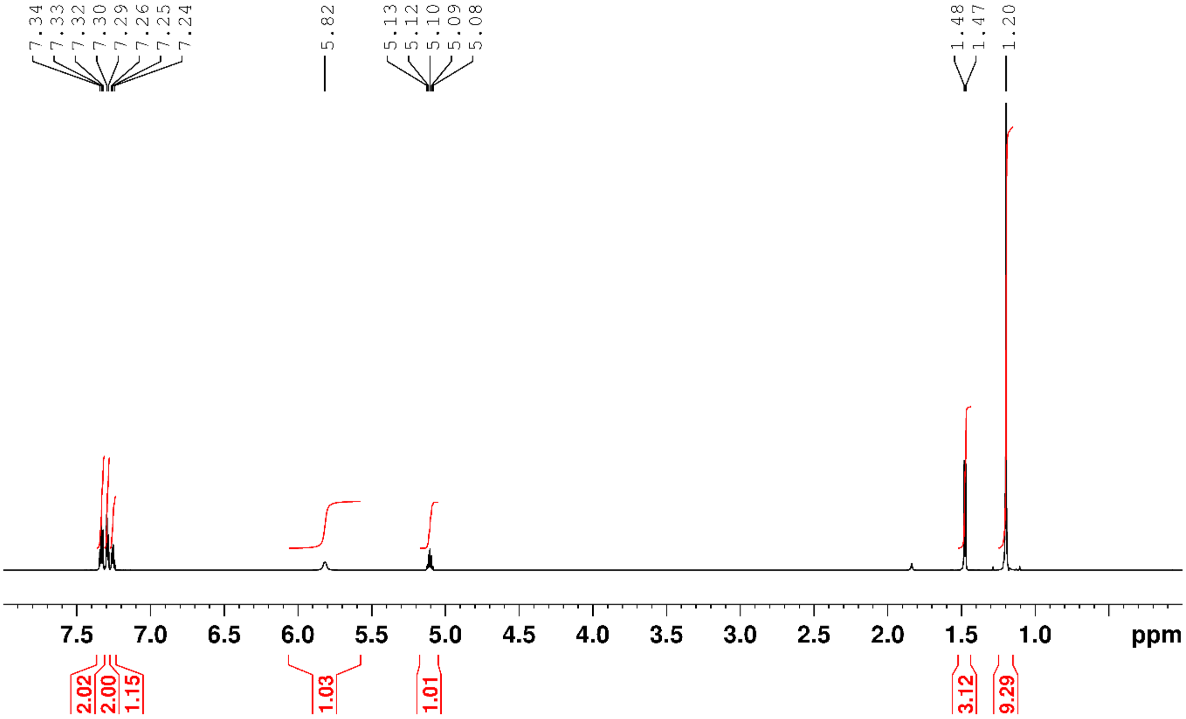

^{1}H NMR spectrum of **27** in $CDCl_3$

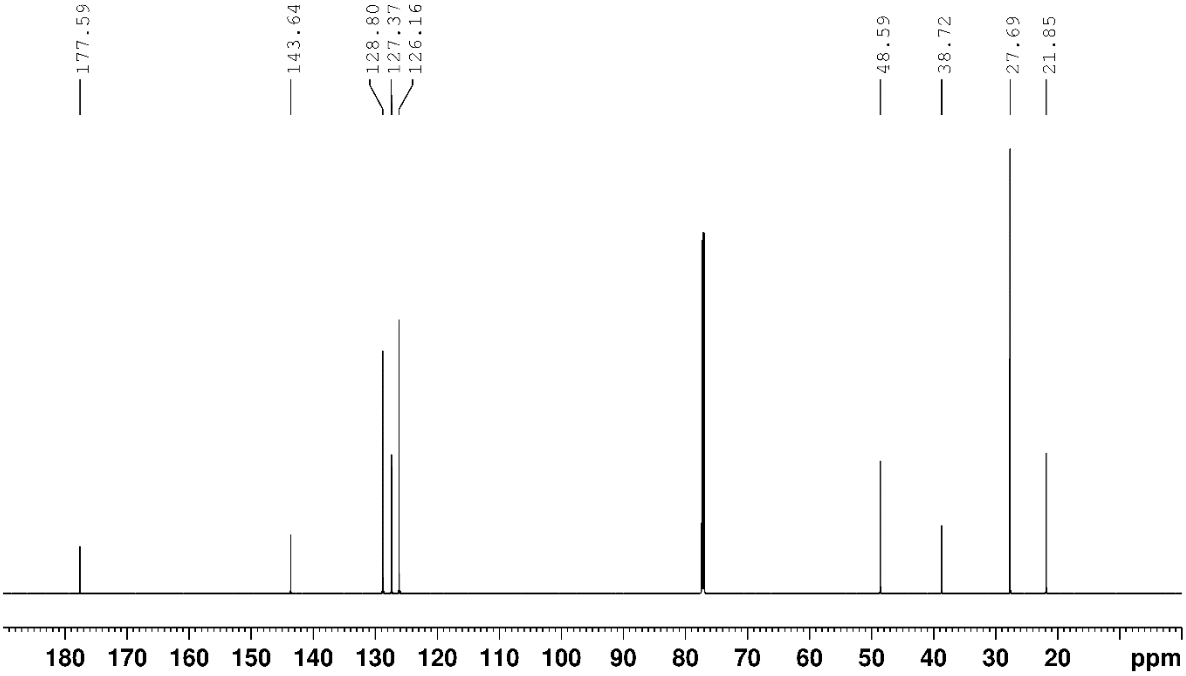

^{13}C NMR spectrum of **27** in $CDCl_3$.

tert-Butyl-(R)-2-(benzylcarbamoyl)pyrrolidine-1-carboxylate (28)

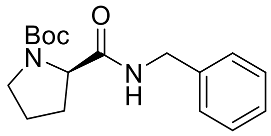

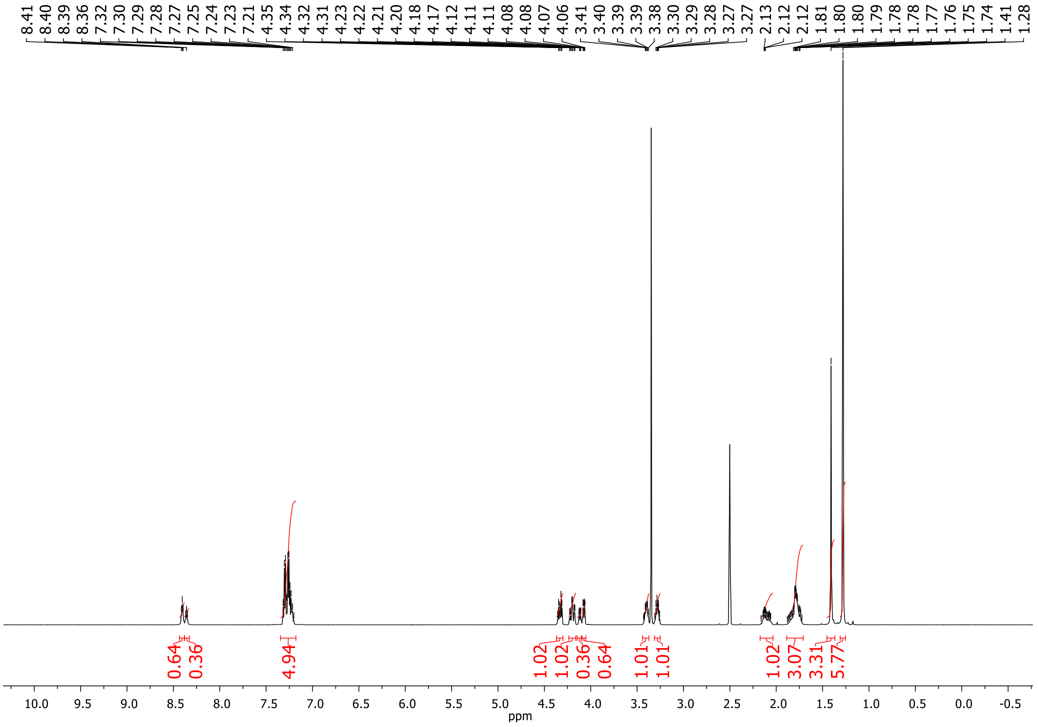

^{1}H NMR spectrum of **28** in DMSO-d_6.

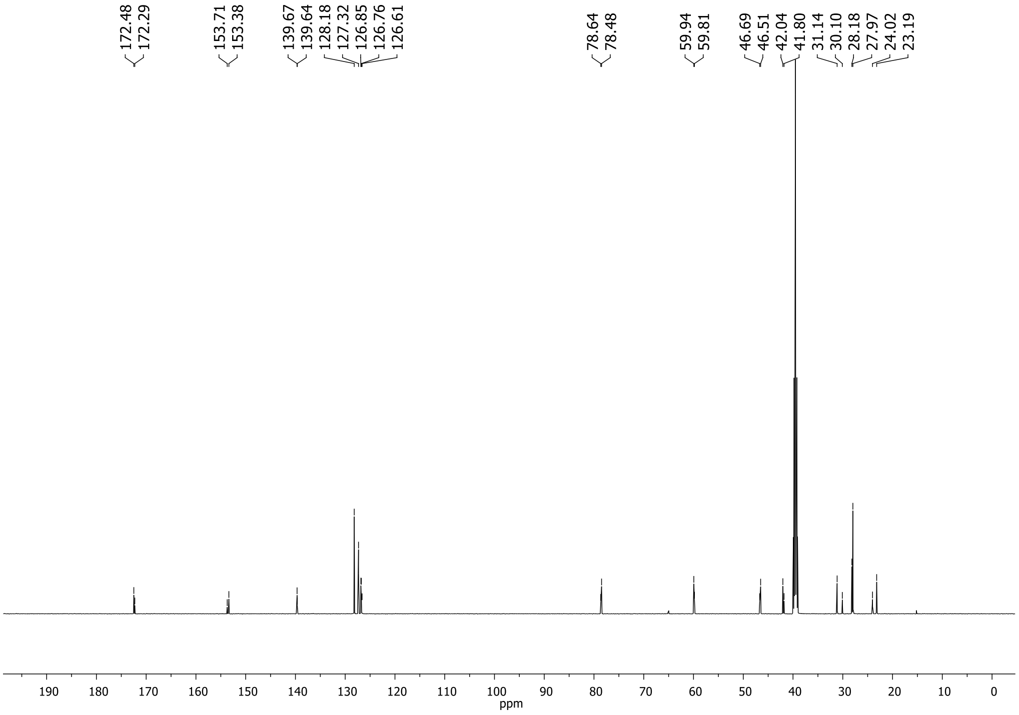

^{13}C NMR spectrum of **28** in DMSO-d_6

tert-Butyl (S)-2-(naphthalen-1-ylcarbamoyl)pyrrolidine-1-carboxylate (29)

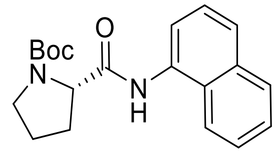

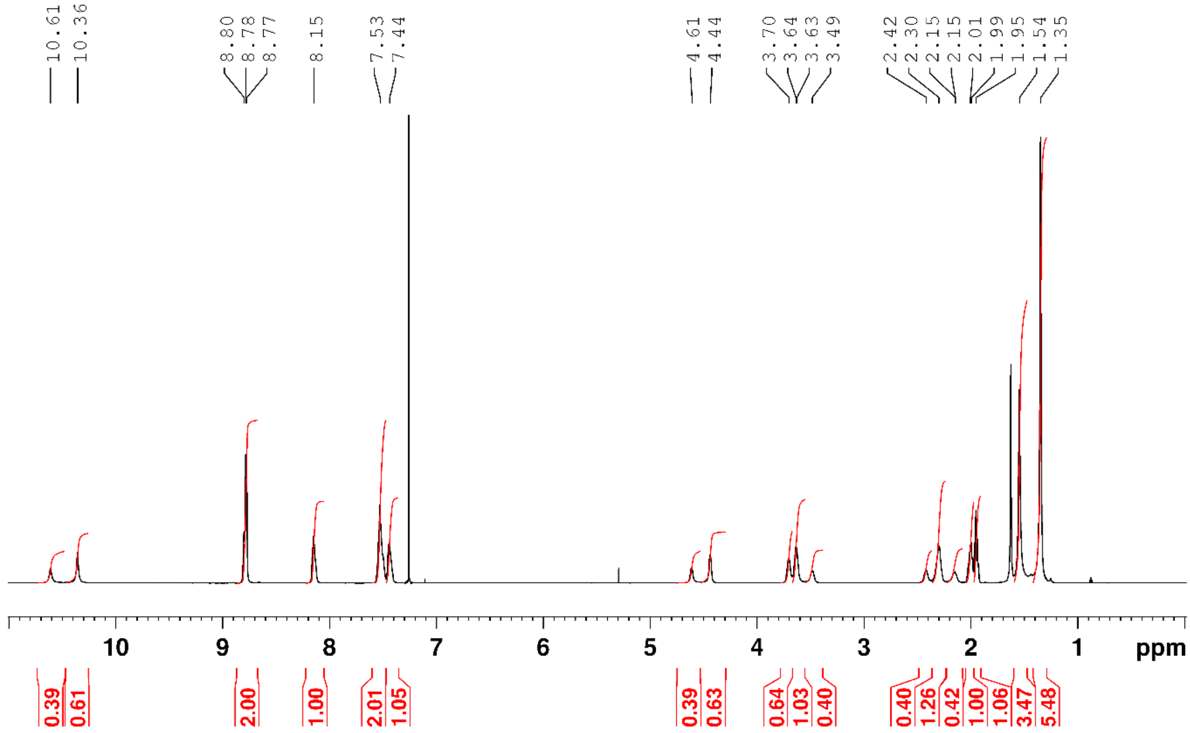

^{1}H NMR spectrum of **29** in $CDCl_3$

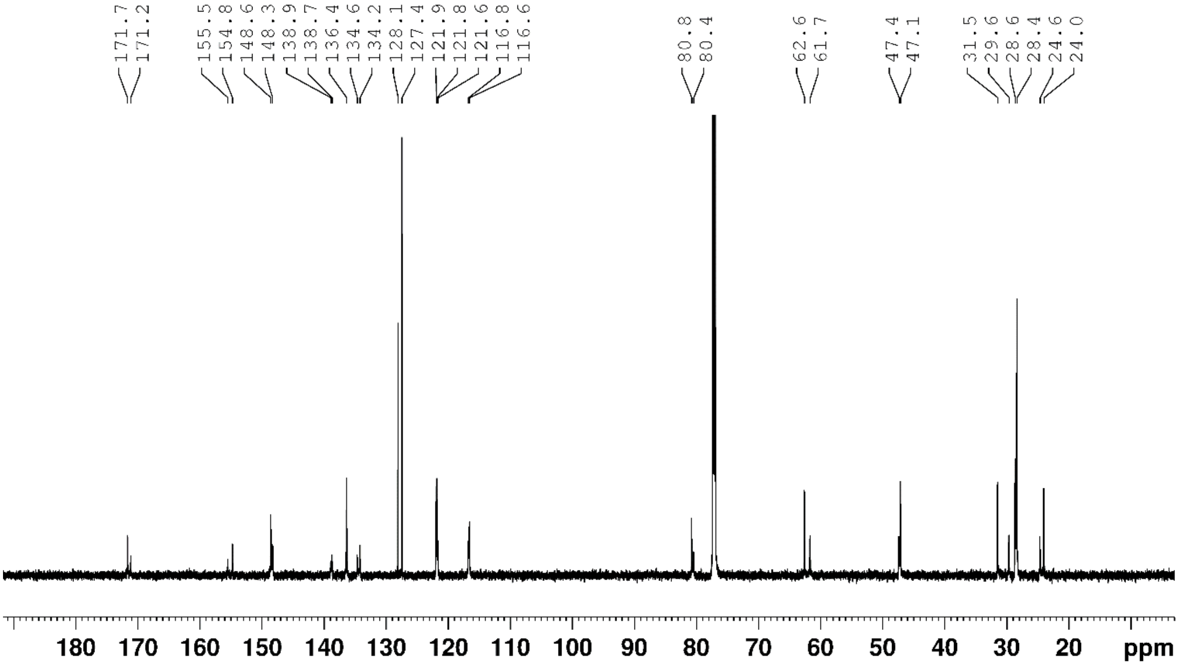

^{13}C NMR spectrum of **29** in $CDCl_3$

(*S*)-2-Amino-N-benzyl-3-methylbutanamide (30)

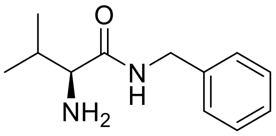

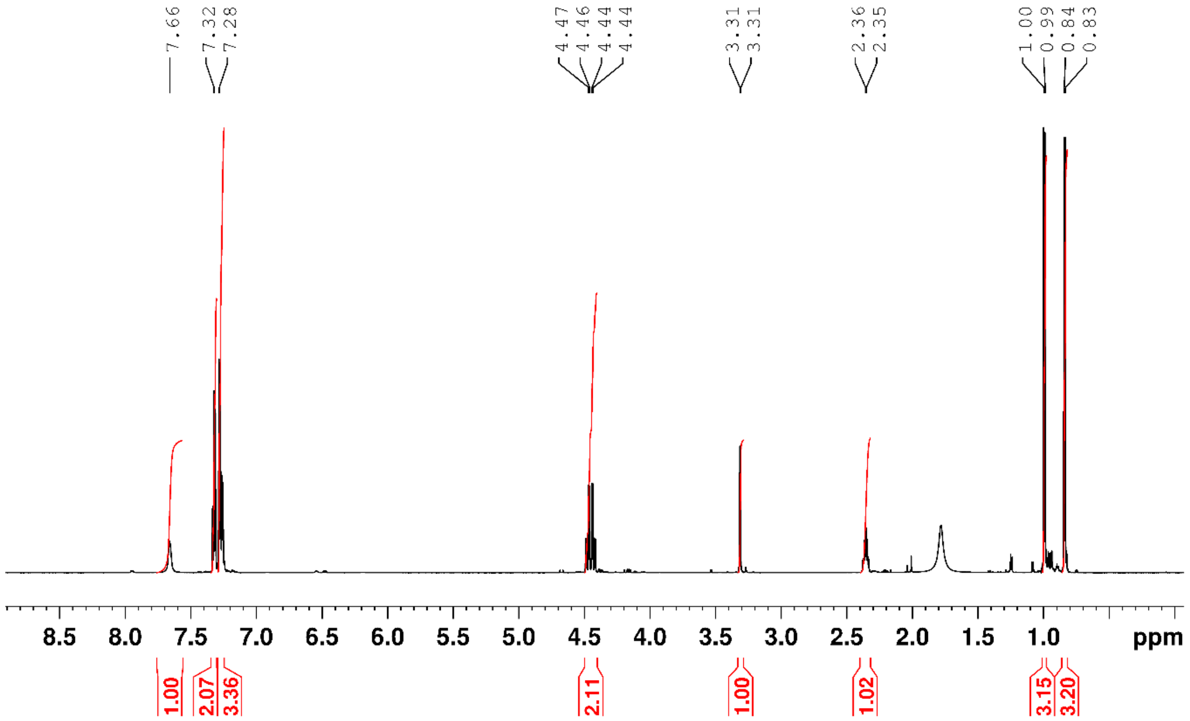

^{1}H NMR spectrum of **30** in $CDCl_3$

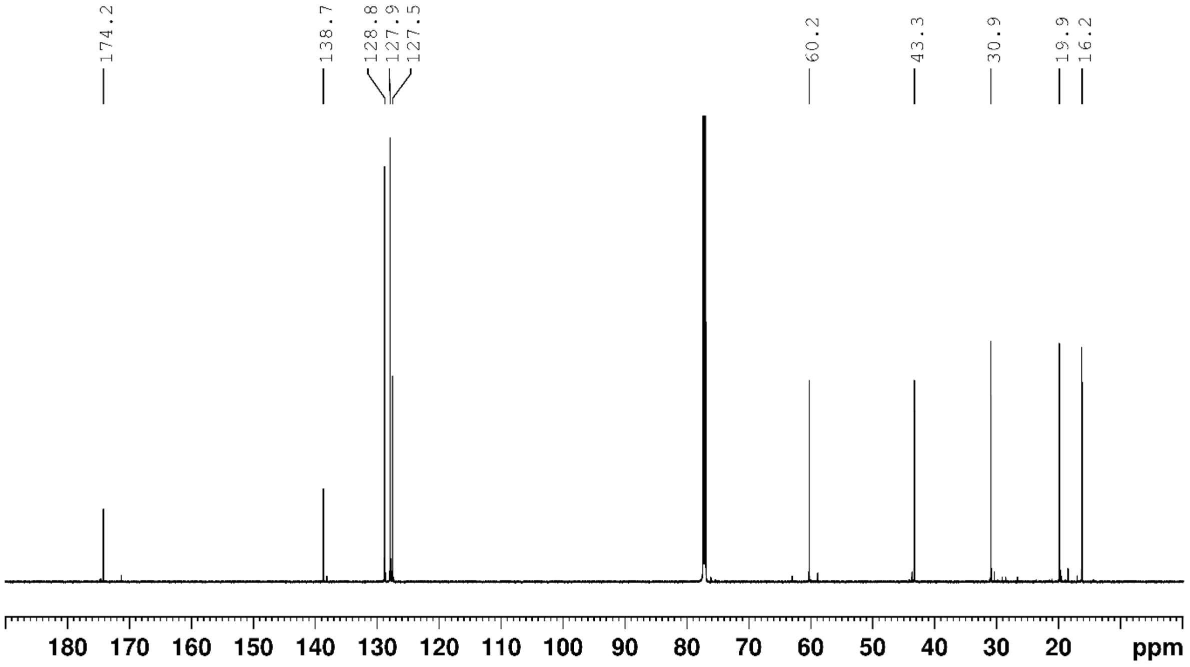

^{13}C NMR spectrum of **30** in $CDCl_3$

N-(Pyridin-2-yl)-2-(thiophen-3-yl)acetamide (31)

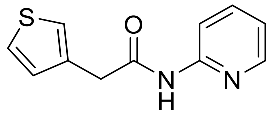

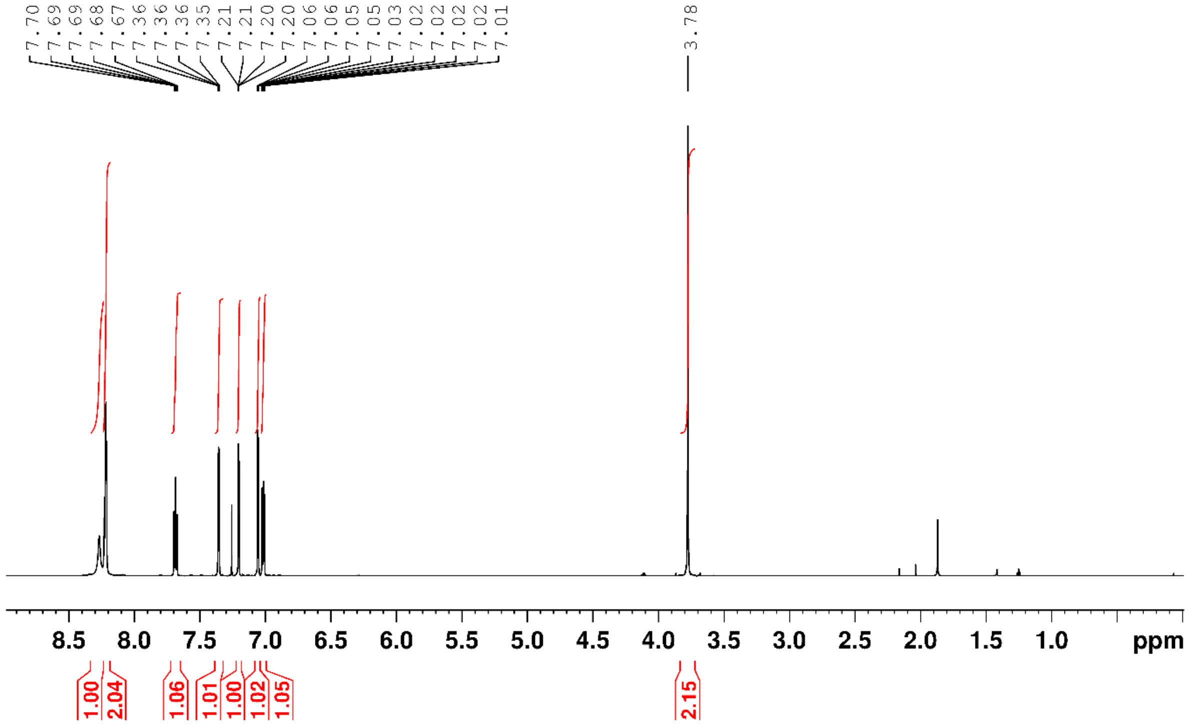

^{1}H NMR spectrum of **31** in $CDCl_3$

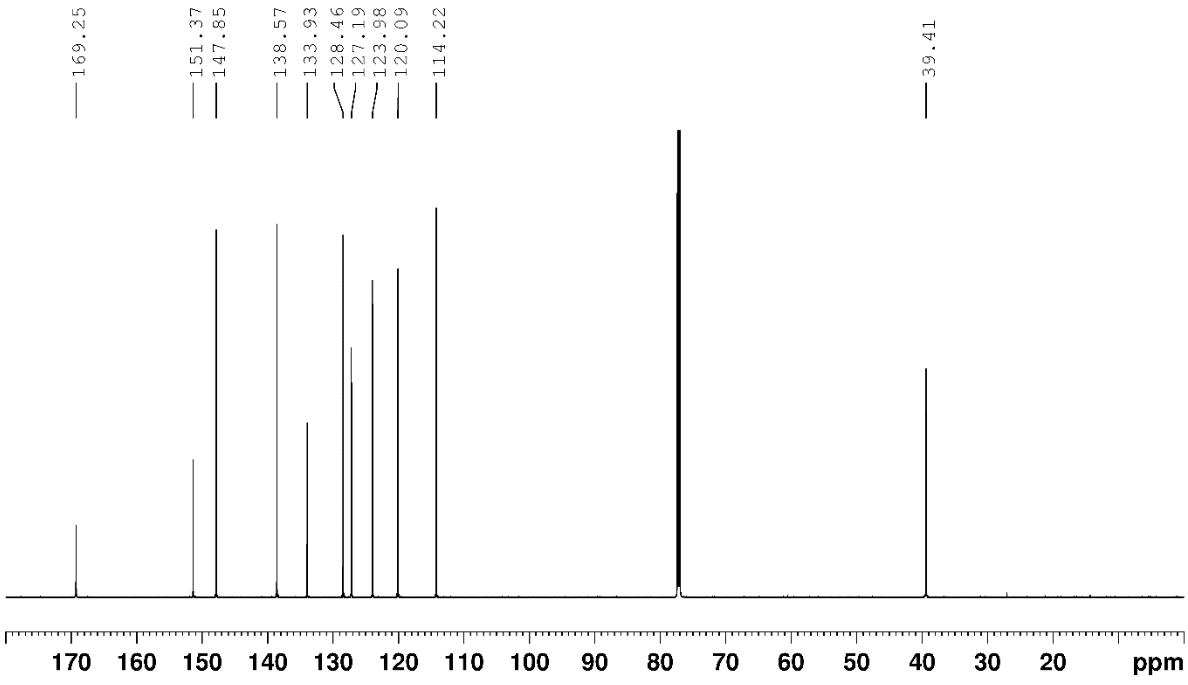

^{13}C NMR spectrum of **31** in $CDCl_3$.

***tert*-Butyl 4-(pyridin-2-ylcarbamoyl)piperidine-1-carboxylate (32)**

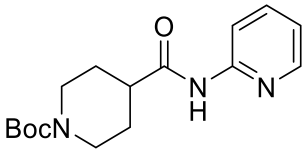

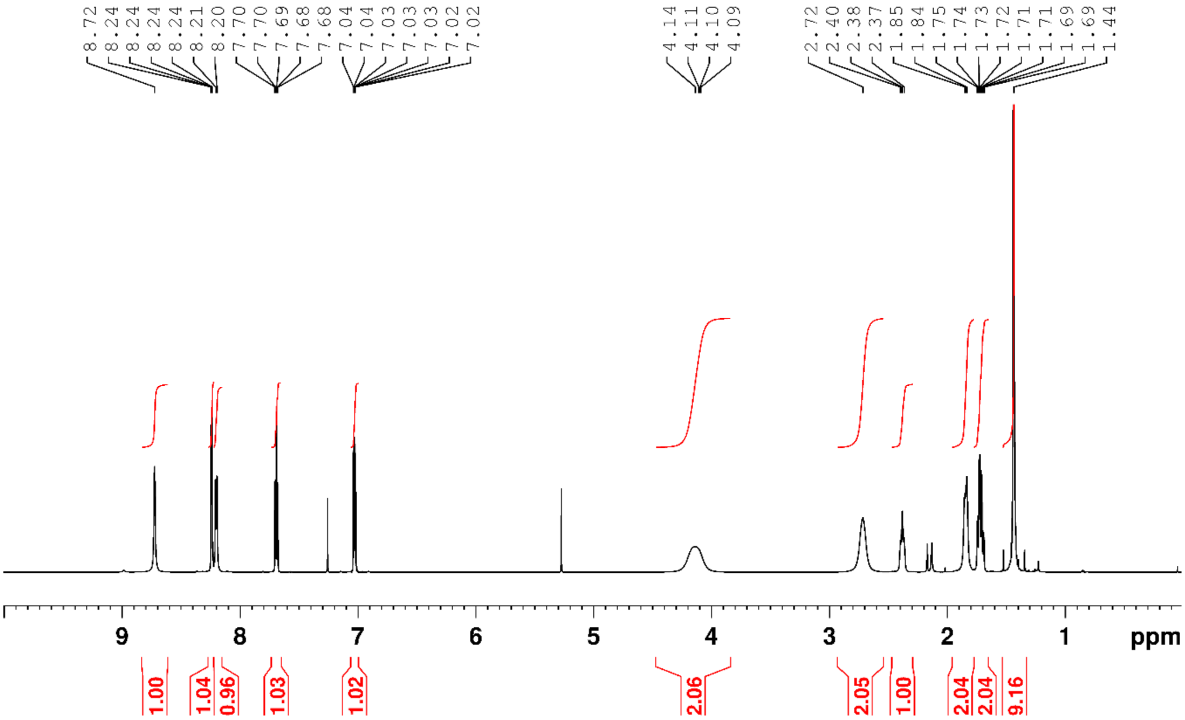

^{1}H NMR spectrum of **32** in $CDCl_3$.

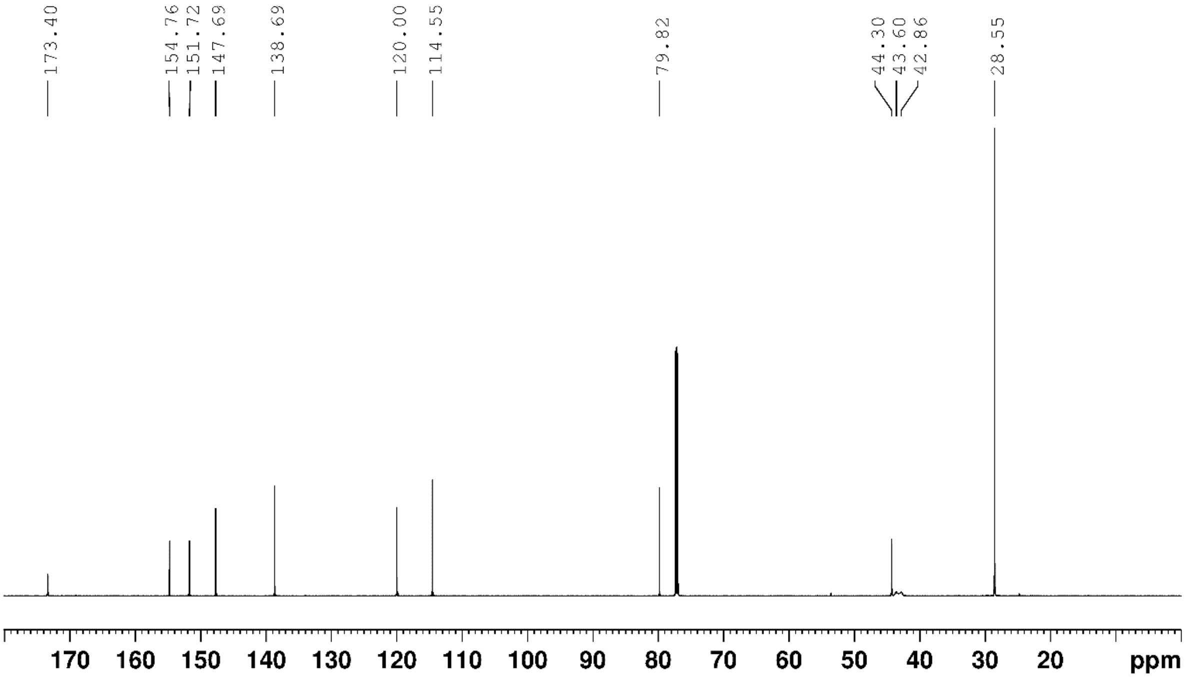

^{13}C NMR spectrum of **32** in $CDCl_3$.

N-(Pyridin-2-yl)-pivalamide (33)

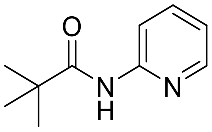

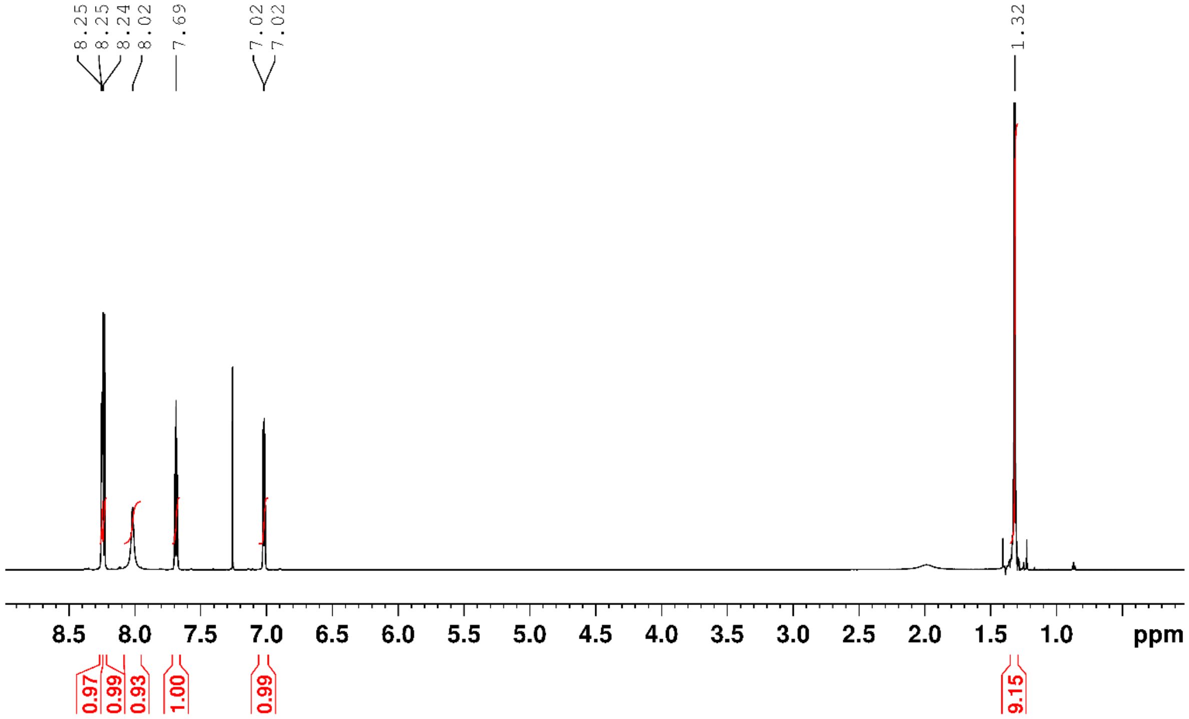

^{1}H NMR spectrum of **33** in $CDCl_3$

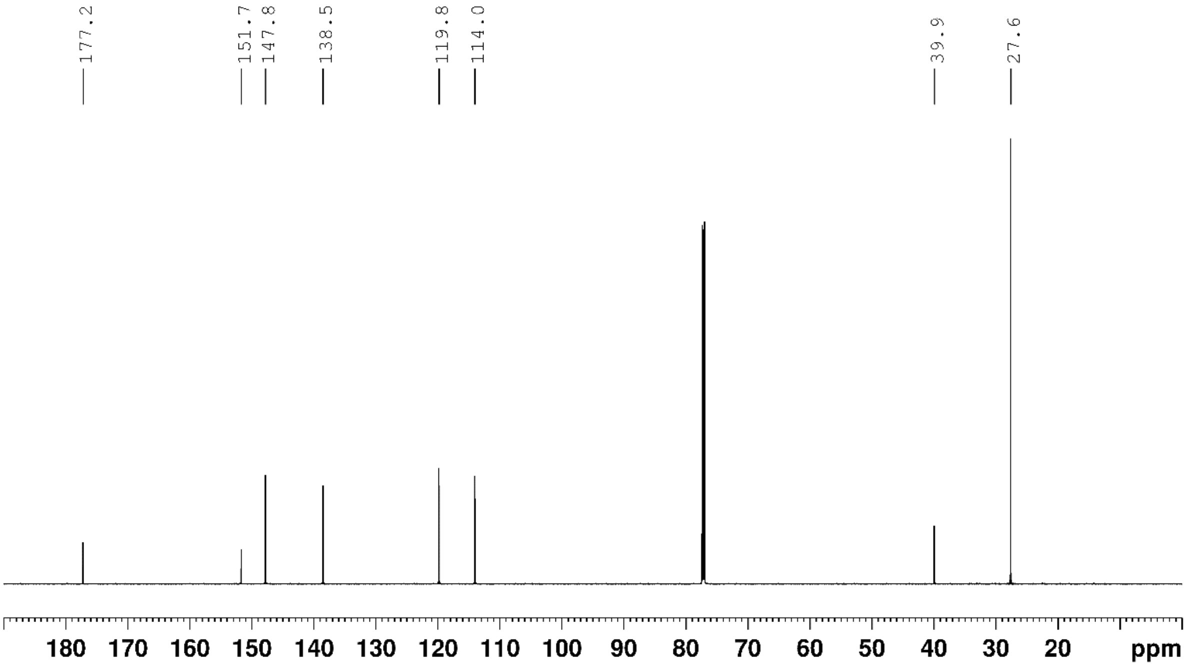

^{13}C NMR spectrum of **33** in $CDCl_3$

***N*-(Pyridin-2-yl)benzamide (34)**

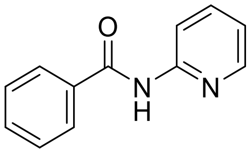

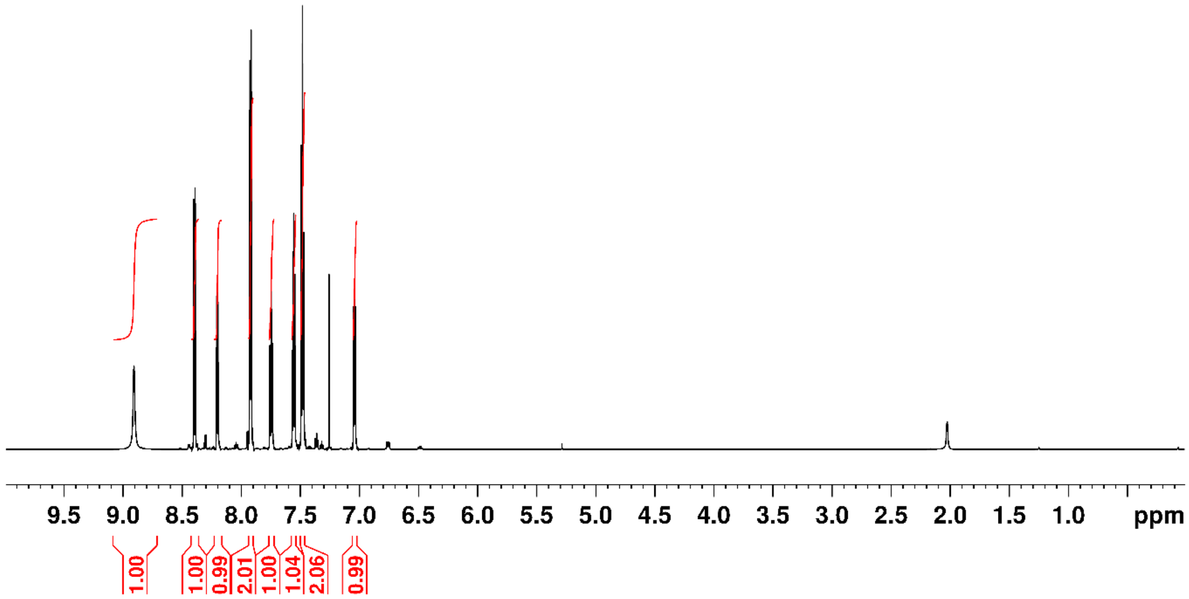

^{1}H NMR spectrum of **34** in $CDCl_3$

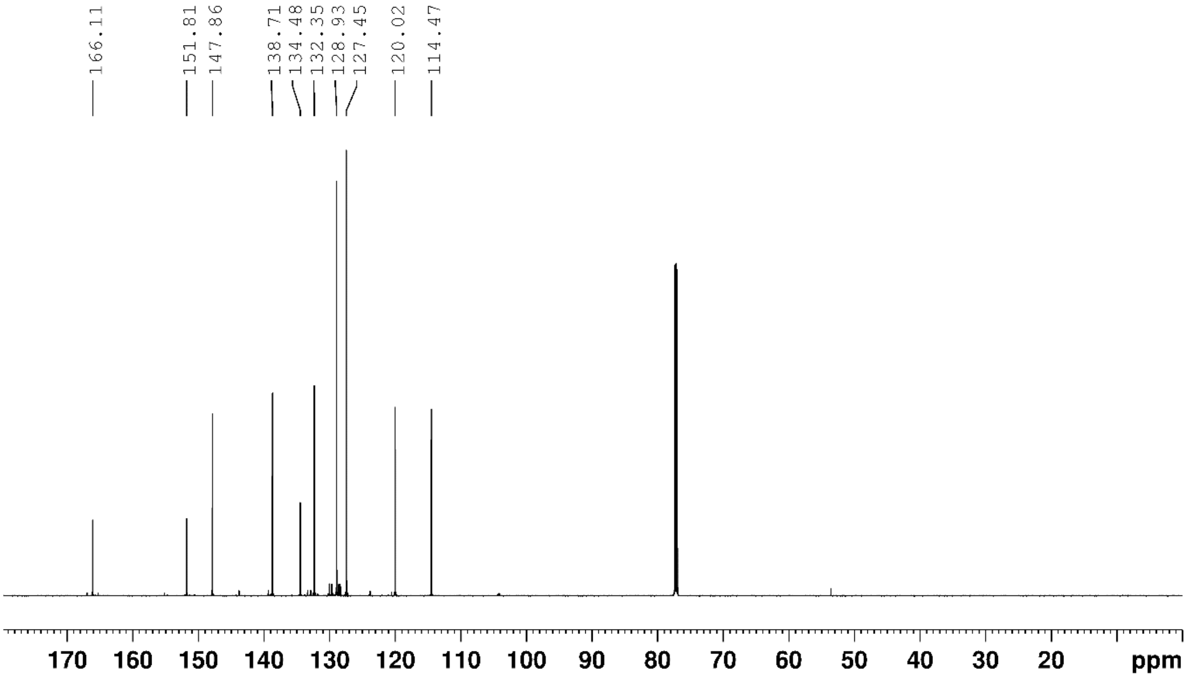

^{13}C NMR spectrum of **34** in $CDCl_3$

N-(Pyridin-2-yl)picolinamide (35)

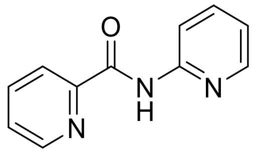

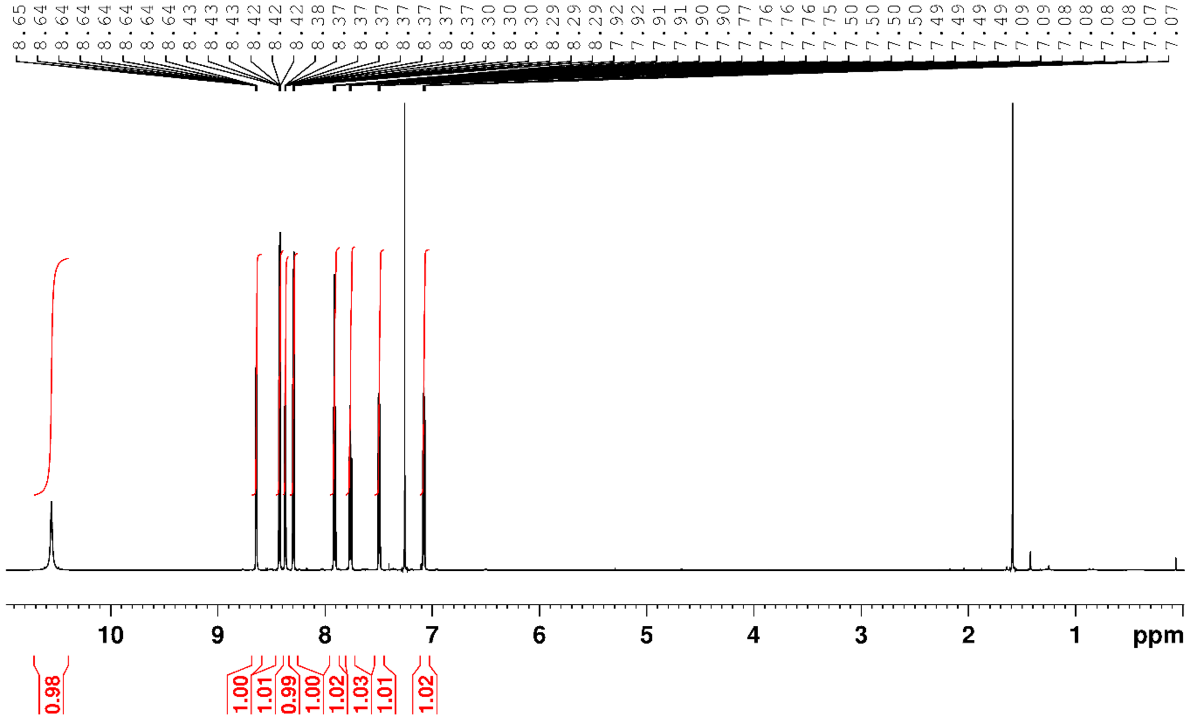

^{1}H NMR spectrum of **35** in $CDCl_3$

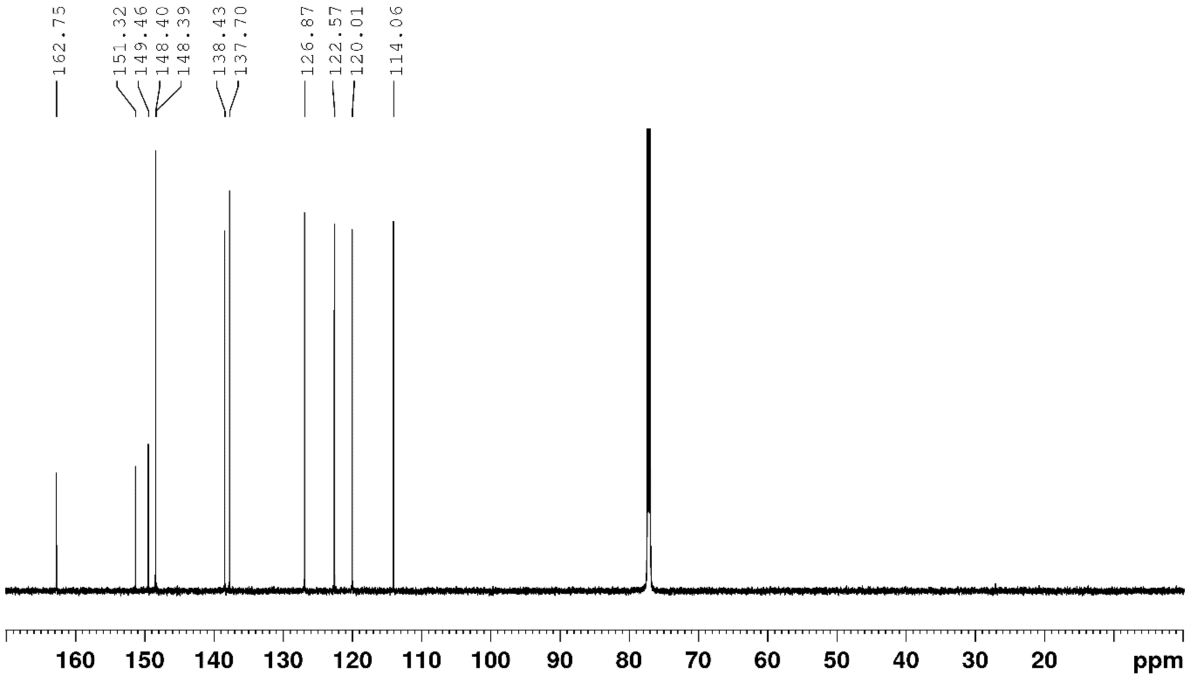

^{13}C NMR spectrum of **35** in $CDCl_3$.

N-(Pyridin-2-yl)-1H-indazole-3-carboxamide (36)

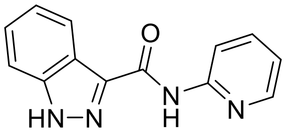

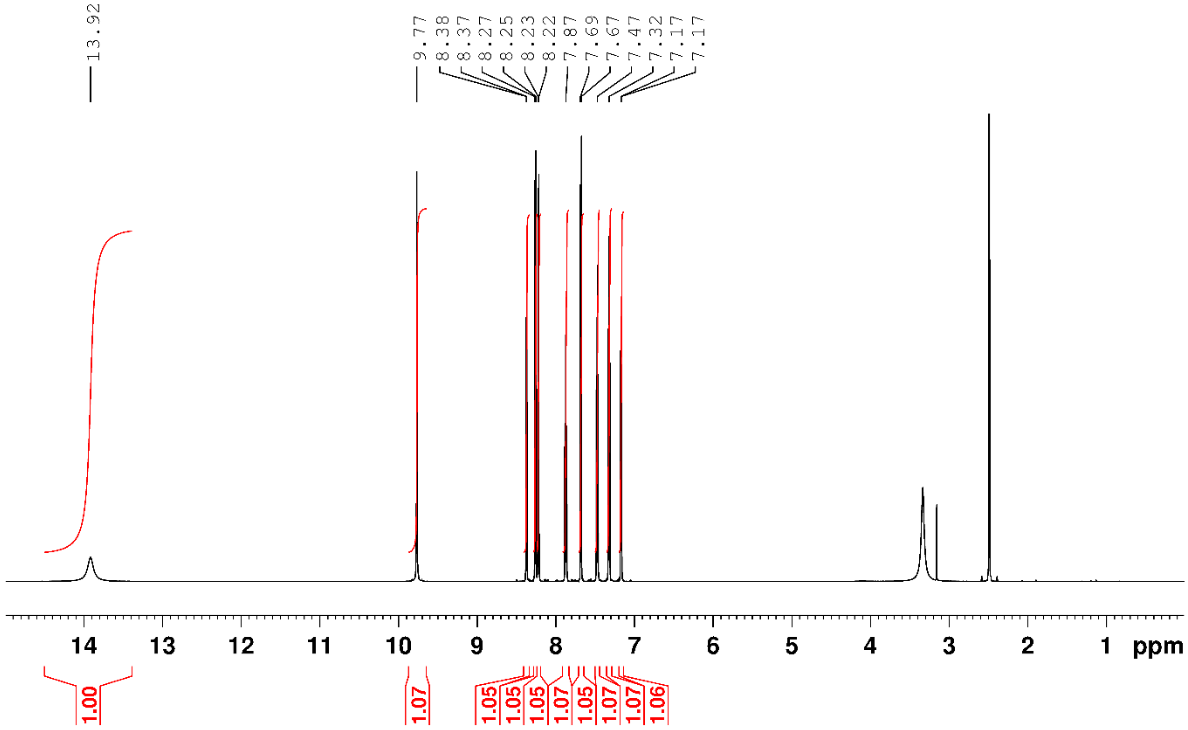

^{1}H NMR spectrum of **36** in DMSO – d_6

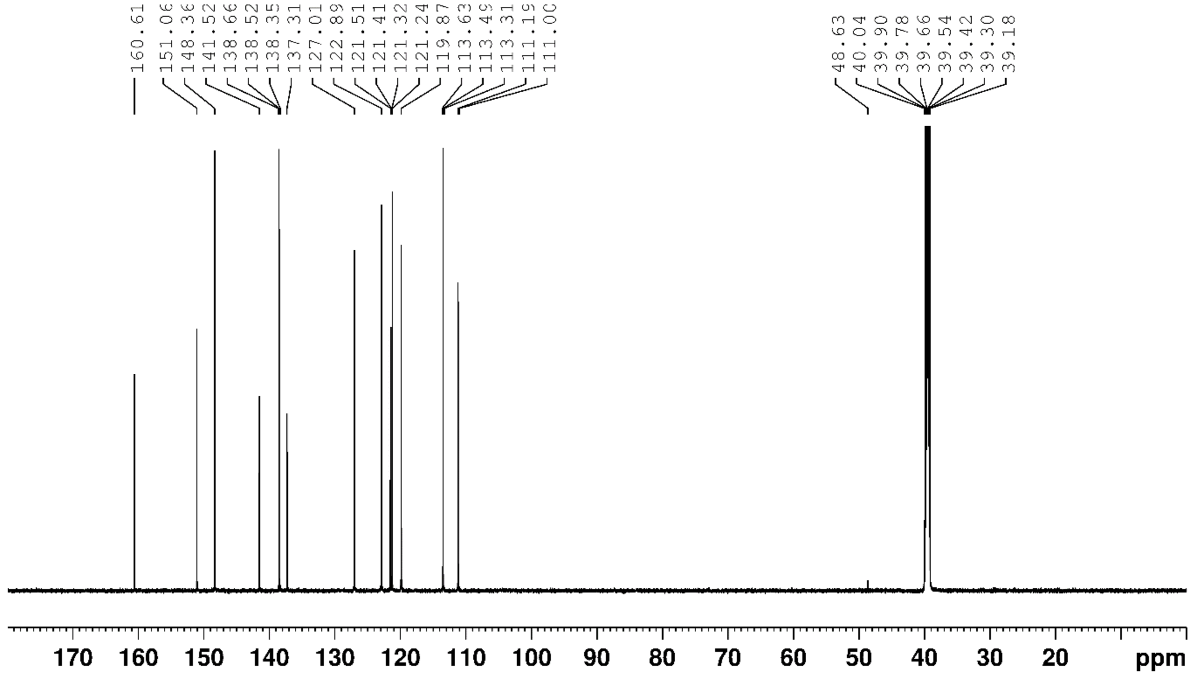

^{13}C NMR spectrum of **36** in DMSO – d_6.

Chiral HPLC traces

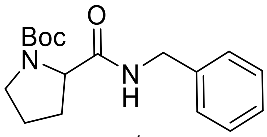

+/-

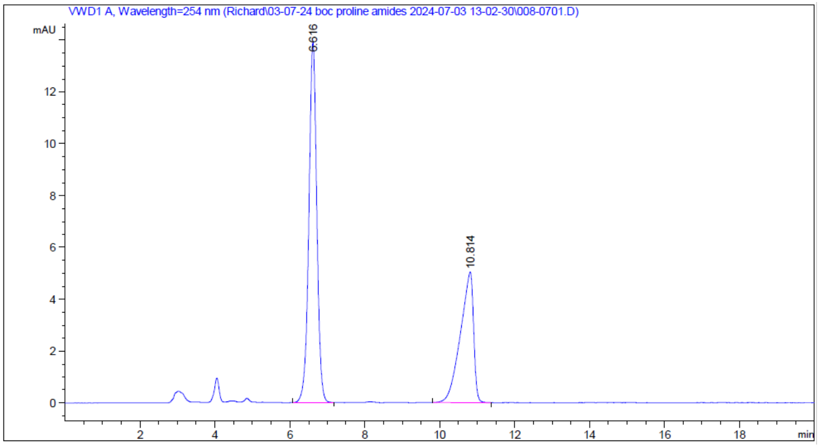

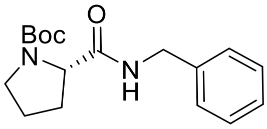

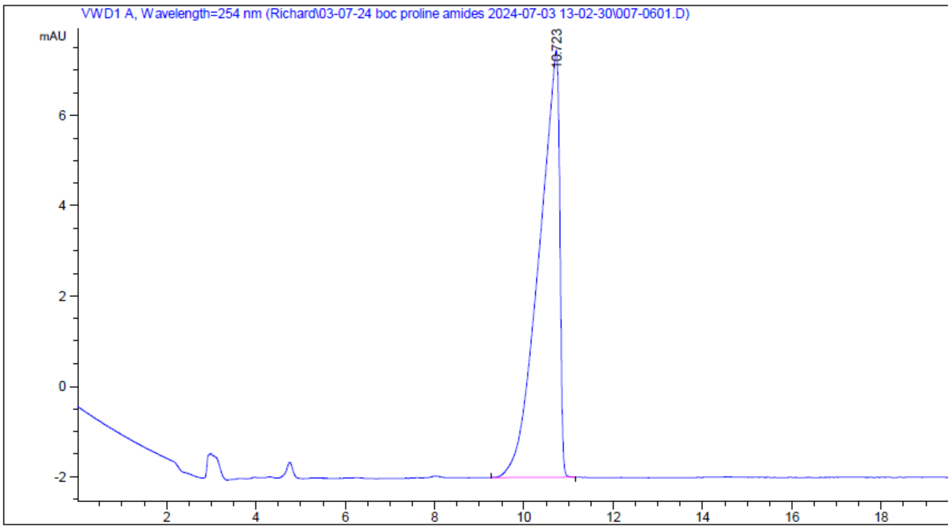

===

Area Percent Report

===

Sorted By : Signal
Multiplier : 1.0000
Dilution : 1.0000
Do not use Multiplier & Dilution Factor with ISTDs

Signal 1: VWD1 A, Wavelength=254 nm

Peak #	RetTime [min]	Type	Width [min]	Area [mAU*s]	Height [mAU]	Area %
1	10.723	BB	0.4207	301.90167	9.45162	100.0000

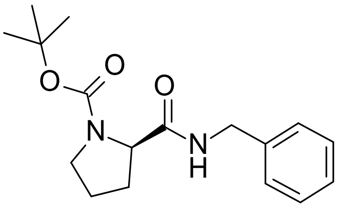

(modified after loading)

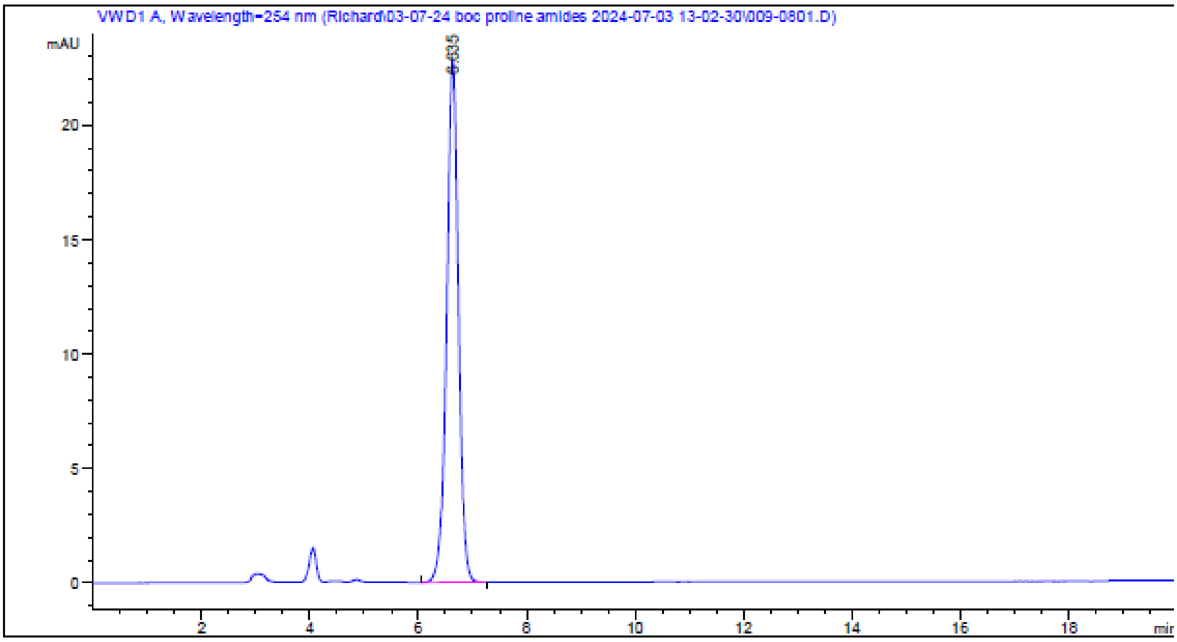


```
=====================================================================
                        Area Percent Report
=====================================================================

Sorted By             :      Signal
Multiplier            :      1.0000
Dilution              :      1.0000
Do not use Multiplier & Dilution Factor with ISTDs


Signal 1: VWD1 A, Wavelength=254 nm

Peak RetTime Type  Width     Area      Height     Area
  #   [min]        [min]   [mAU*s]     [mAU]        %
----|-------|----|-------|----------|----------|--------|
   1   6.635 BB    0.2327  346.97598   22.78194 100.0000

Totals :                   346.97598   22.78194
```

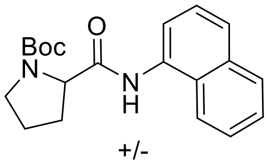

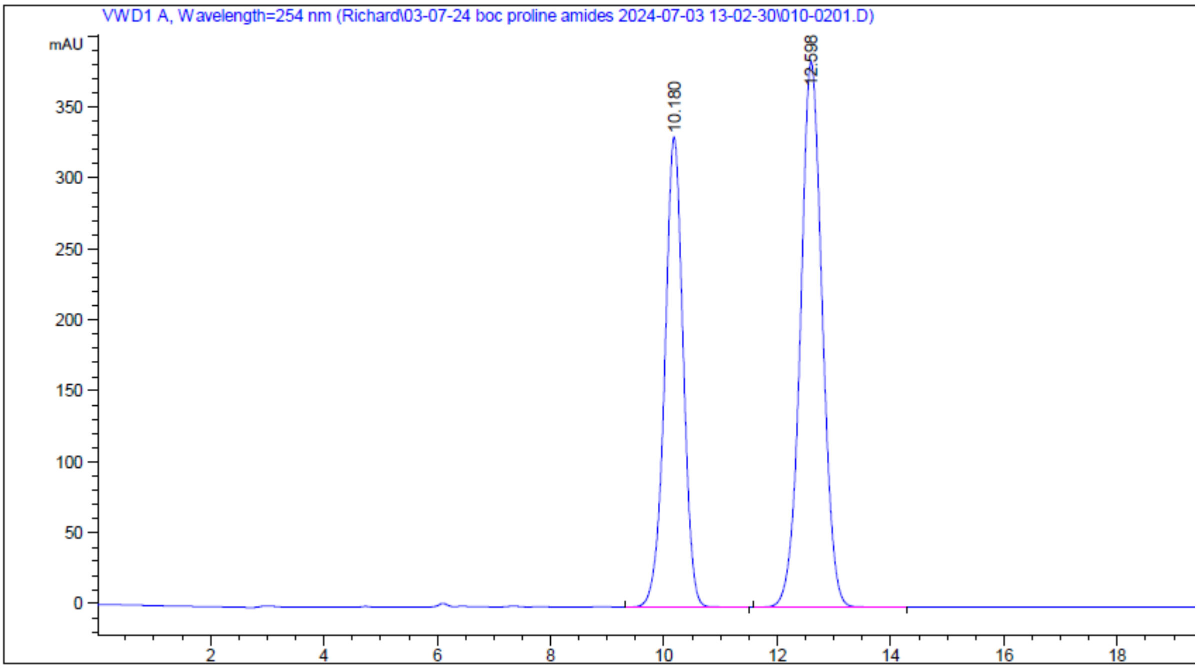

===

Area Percent Report

===

Sorted By : Signal
Multiplier : 1.0000
Dilution : 1.0000
Do not use Multiplier & Dilution Factor with ISTDs

Signal 1: VWD1 A, Wavelength=254 nm

Peak #	RetTime [min]	Type	Width [min]	Area [mAU*s]	Height [mAU]	Area %
1	10.180	BB	0.3449	7461.55469	331.06152	42.1006
2	12.598	BB	0.4096	1.02616e4	384.23862	57.8994

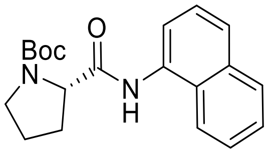

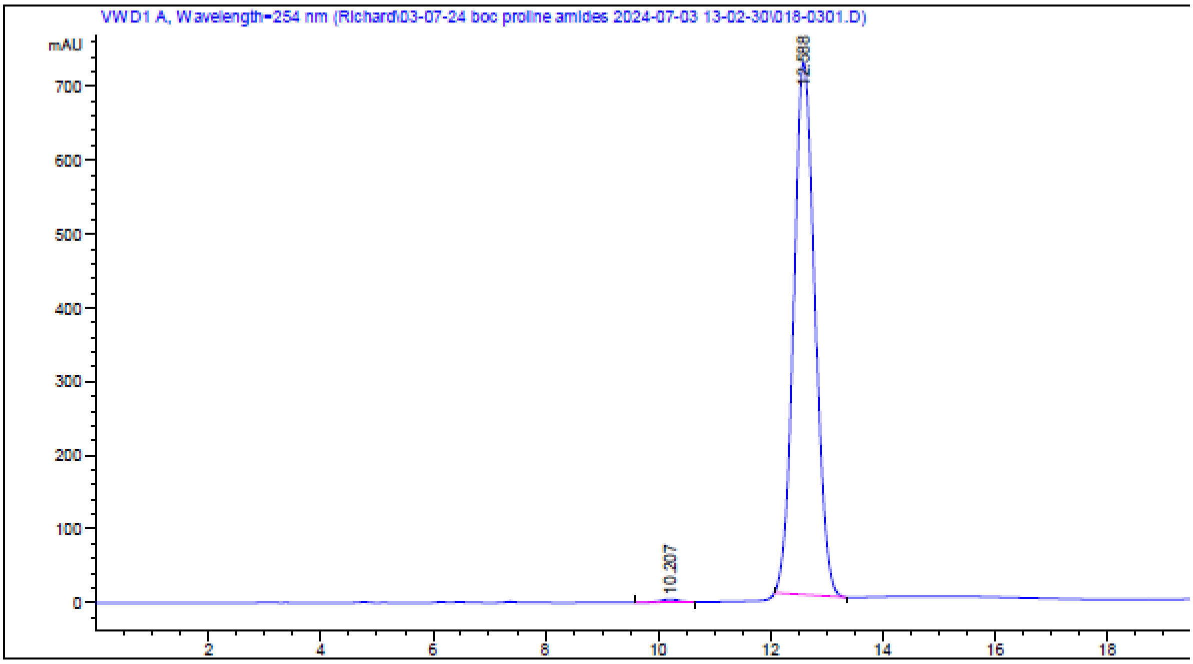

===

Area Percent Report

===

Sorted By : Signal
Multiplier : 1.0000
Dilution : 1.0000
Do not use Multiplier & Dilution Factor with ISTDs

Signal 1: VWD1 A, Wavelength=254 nm

Peak #	RetTime [min]	Type	Width [min]	Area [mAU*s]	Height [mAU]	Area %
1	10.207	BB	0.3281	90.79762	4.23518	0.4763
2	12.588	BB	0.3927	1.89711e4	721.64771	99.5237

Totals : 1.90619e4 725.88288

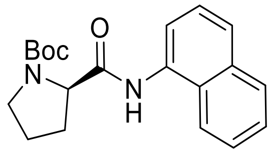

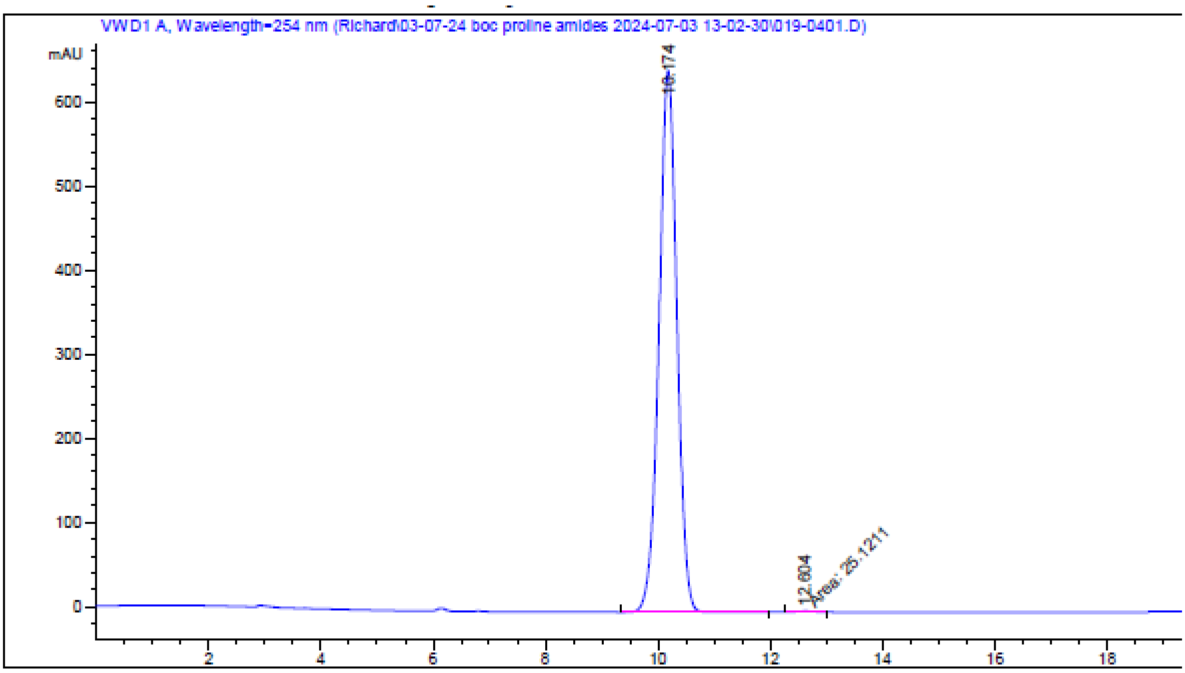


```
=====================================================================
                         Area Percent Report
=====================================================================

Sorted By             :      Signal
Multiplier            :      1.0000
Dilution              :      1.0000
Do not use Multiplier & Dilution Factor with ISTDs


Signal 1: VWD1 A, Wavelength=254 nm

Peak RetTime Type  Width     Area      Height     Area
  #   [min]        [min]   [mAU*s]     [mAU]        %
----|-------|----|-------|----------|----------|--------|
   1  10.174 BB    0.3414 1.42840e4  642.53271  99.8244
   2  12.604 MM T  0.3749   25.12111    1.11675   0.1756
```

Enantiopurity determination with Marfey's reagent

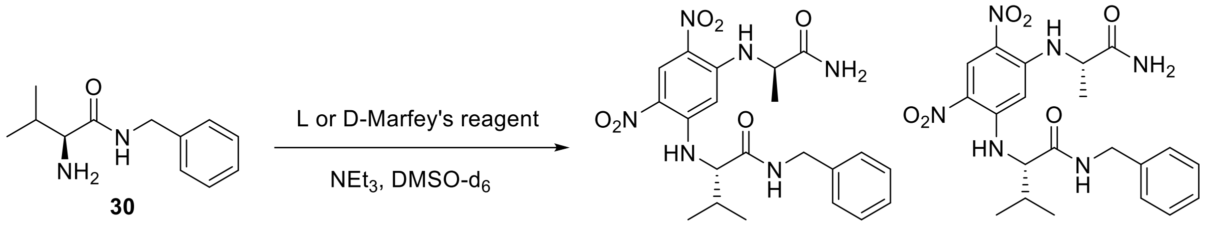

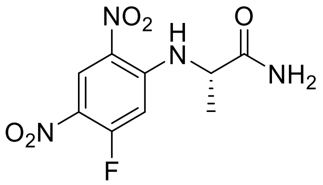

L-Marfey's reagent

In two separate reactions, compound **30** (10 mg, 0.05 mmol) and R- or S- Marfey's reagent (20 mg, 0.075 mmol) were dissolved in DMSO-d_6 (500 µl), and triethylamine (7 µl, 0.05 mmol) was added. The reactions were heated to 40 °C for 1 hour then analysed by NMR spectroscopy.

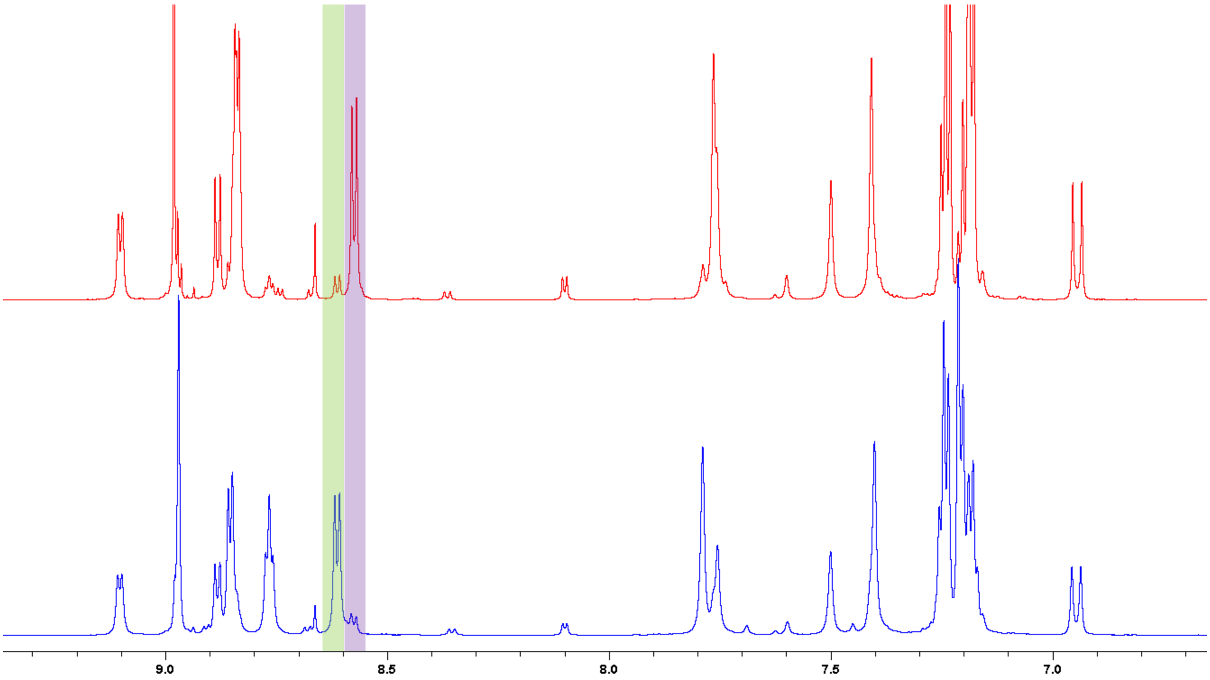

Aromatic section of the ^{1}H NMR spectra of the reactions of **30** with Marfey's reagents in DMSO-d_6. The peaks used for analysis of enantiomeric purity are highlighted

Single crystal x-ray diffraction studies

The diffraction data was collected on a four-circle *Agilent SuperNova* (Dual Source) single crystal X-ray diffractometer using a micro-focus CuK_α X-ray beam (λ = 1.54184 Å) and a *HyPix-Arc 100°* hybrid pixel array detector. The sample temperatures were controlled with an *Oxford Instruments* cryojet. The data were processed using the *CrysAlis*^{Pro} software[29].

The crystal structure was solved with the *SHELXT* programme[30], used within the *Olex2* software suite[31], and refined by least squares on the basis of F^2 with the *SHELXL*[32] programme using the *ShelXle* graphical user interface[33]. All non-hydrogen atoms were refined anisotropically by the full-matrix least-squares method. Hydrogen atoms associated with carbon atoms were refined isotropically [U_{iso}(H) = 1.2U_{eq}(C)] in geometrically constrained positions. The positions of hydrogen atoms affiliated with the amino group were located in the difference Fourier map and refined isotopically [U_{iso}(H) = 1.5U_{eq}(N)] using the DFIX and DANG commands in *SHELXL*.

Relevant crystallographic information and refinement parameters are shown in the table below.

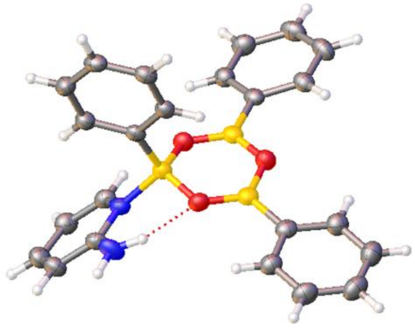

Crystallographic information and details of refinement parameters.

empirical formula	$C_{23}H_{21}B_3N_2O_3$
M_r / g mol^{-1}	405.85
crystal system	orthorhombic
space group	*Pbca*
a / Å	6.81856(4)
b / Å	18.79523(11)
c / Å	32.85532(19)
α / °	90
β / °	90
γ / °	90
V / Å^3	4210.62(4)
Z	8
ρ_{calc} / g cm^{-3}	1.280
T / K	150.0(1)
μ / mm^{-1}	0.657
F(000)	1696
crystal size / mm^3	0.23 × 0.12 × 0.04
radiation	CuK_α (λ = 1.54184 Å)
2θ range for data collection / °	9.410−133.180
index ranges	$-8 \le h \le 8$
	$-22 \le k \le 22$
	$-39 \le l \le 39$
number of collected reflections	94888
unique reflections	3713
number of unique reflections	3408 [$I > 2\sigma(I)$]
R_{int}	0.0349
$R(F)$, $F > 2\sigma$(F)	0.0306
$wR(F^2)$, $F > 2\sigma$(F)	0.0804
$R(F)$, all data	0.0333
$wR(F^2)$, all data	0.0824
Δ_r (max., min.) e Å^{-3}	0.165/−0.180
CCDC deposition number	2391496

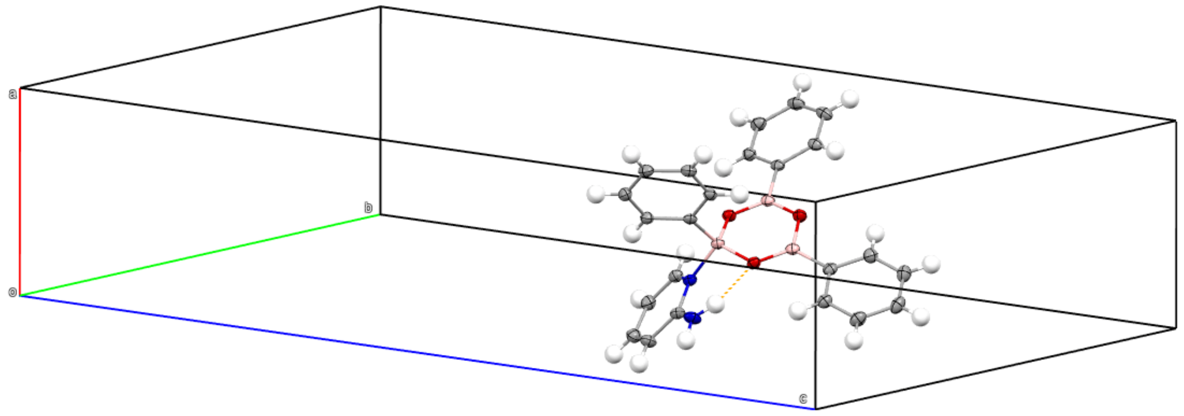

The asymmetric unit. The thermal ellipsoids are drawn at the 50% probability level. Colour scheme: carbon – grey, nitrogen – blue, oxygen – red, boron – pink.

Raw data is available at [10.14469/hpc/14694](10.14469/hpc/14694)

Computational methods

Co-ordinate files can be found at Imperial College Research Data Repository, DOI: 10.14469/hpc/12218

Treatment of 2-chlorophenylboronic acid with 2-aminopyridine led to the formation of a stable boroxine complex. Calculations at the B3LYP+GD3+BJ/Def2-TZVPP/SCRF level[34] suggest this complex is 5.1 kcal mol^{-1} lower in free energy (DG298) than the free components using chloroform as a continuum solvent. The corresponding values for complexes with benzylamine and aniline are respectively -5.0 and +1.2 kcal mol^{-1}. An NCI (non-covalent interaction) analysis[35,36] of the total electron density suggests that the 2-aminopyridine complex is significantly stabilised both by an intramolecular hydrogen bond from NH to O and by stacking interactions between the chlorine of one o-Cl aryl substituent and the boroxine ring, along with a number of smaller effects. The benzylamine complex is predominantly stabilised by intramolecular p-p stacking, something which is not possible with the aniline complex due to its more rigid nature.

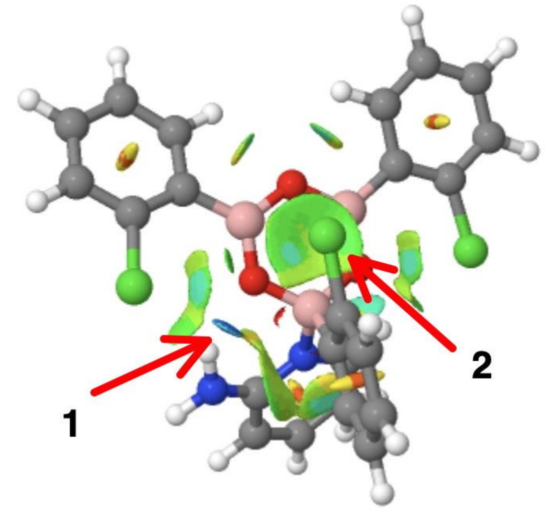

2-aminopyridine

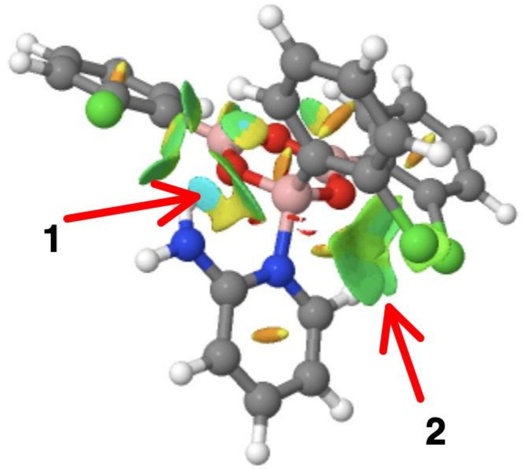

2-aminopyridine conformer

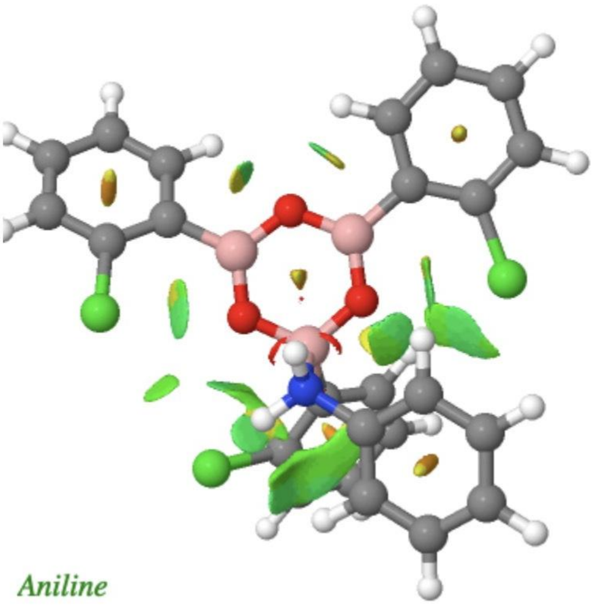

Aniline

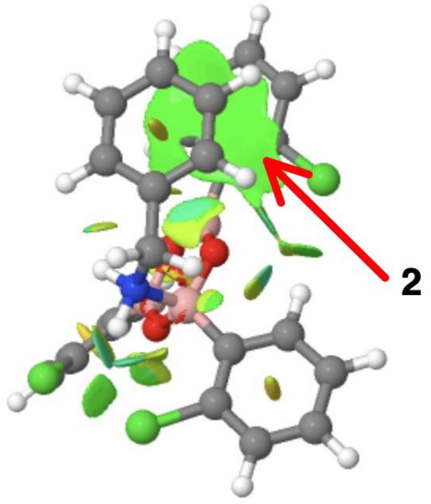

Benzylamine

References

1. M. T. Sabatini, L. T. Boulton and T. D. Sheppard, *Sci. Adv.*, 2017, **3**, e1701028.
2. H. S. Lee, X. Q. Yang, C. L. Xiang, J. McBreen and L. S. Choi, *J. Electrochem. Soc.*, 1998, **145**, 2813.
3. H. Böhrer, N. Trapp, D. Himmel, M. Schleep and I. Krossing, *Dalton Trans.*, 2015, **44**, 7489-7499.
4. T. Maki, K. Ishihara and H. Yamamoto, *Org. Lett.*, 2006, **8**, 1431-1434.
5. R. Tsutsumi, N. Kashiwagi and N. Kumagai, *J. Org. Chem.*, 2023, **88**, 6247-6251.
6. T. Colclough, W. Gerrard and M. F. Lappert, *J. Chem. Soc.*, 1956, DOI: 10.1039/JR9560003006, 3006-3010.
7. F. A. LeBlanc, A. Decken, T. S. Cameron, J. Passmore, J. M. Rautiainen and T. K. Whidden, *Inorg. Chem.*, 2017, **56**, 974-983.
8. T. T. T. Nguyen, V. D. Duong, T. N. N. Pham, Q. T. Duong and T. B. Nguyen, *Org. Biomol. Chem.*, 2022, **20**, 8054-8058.
9. W.-H. Rao, B.-B. Zhan, K. Chen, P.-X. Ling, Z.-Z. Zhang and B.-F. Shi, *Org. Lett.*, 2015, **17**, 3552-3555.
10. A.-S. Piticari, D. Antermite, J. I. Higham, J. H. Moore, M. P. Webster and J. A. Bull, *Adv. Synth. Catal.*, 2022, **364**, 1488-1497.
11. V. Karaluka, R. M. Lanigan, P. M. Murray, M. Badland and T. D. Sheppard, *Org. Biomol. Chem.*, 2015, **13**, 10888-10894.
12. C. Li and H.-L. Qin, *Org. Lett.*, 2019, **21**, 4495-4499.
13. Á. M. Martínez, N. Rodríguez, R. Gómez Arrayás and J. C. Carretero, *Chem. Commun.*, 2014, **50**, 6105-6107.
14. S. B. Salunke, N. S. Babu and C.-T. Chen, *Adv. Synth. Catal.*, 2011, **353**, 1234-1240.
15. C. E. Coomber, V. Laserna, L. T. Martin, P. D. Smith, H. C. Hailes, M. J. Porter and T. D. Sheppard, *Org. Biomol. Chem.*, 2019, **17**, 6465-6469.
16. M. Kissane, S. E. Lawrence and A. R. Maguire, *Org. Biomol. Chem.*, 2010, **8**, 2735-2748.
17. Y. Zhao, L. Xu, J. Zhang, M. Zhang, J. Lu, R. He, J. Xi, R. Zhuang, J. Li and Y. Zhou, *Biorg. Med. Chem.*, 2021, **29**, 115867.
18. E. Qu, S. Li, J. Bai, Y. Zheng and W. Li, *Org. Lett.*, 2022, **24**, 58-63.
19. J. d. M. Muñoz, J. Alcázar, A. de la Hoz, Á. Díaz-Ortiz and S.-A. Alonso de Diego, *Green Chem.*, 2012, **14**, 1335-1341.
20. J. Britton, J. M. Chalker and C. L. Raston, *Chem. Eur. J.*, 2015, **21**, 10660-10665.
21. A. R. Bayguzina, A. R. Lutfullina and R. I. Khusnutdinov, *Russ. J. Org. Chem.*, 2018, **54**, 1127-1133.
22. D. C. Braddock, J. J. Davies and P. D. Lickiss, *Org. Lett.*, 2022, **24**, 1175-1179.
23. C. L. Allen, A. R. Chhatwal and J. M. J. Williams, *Chem. Commun.*, 2012, **48**, 666-668.
24. B. V. Subba Reddy, K. Bhavani, A. Raju and J. S. Yadav, *Tetrahedron: Asymmetry*, 2011, **22**, 881-886.
25. S. Kayser, J. C. Hansen, M. Staudt, A. Moroz, Y. Larsen, P. Temperini, F. Yi, J. T. Syrenne, N. Krogsgaard-Larsen, S. Iliadis, B. Nielsen, K. B. Hansen, D. S. Pickering and L. Bunch, *ACS Chemical Neuroscience*, 2020, **11**, 674-701.
26. J. Kumar, A. K. Singh, A. Gupta and S. Bhadra, *J. Org. Chem.*, 2022, **87**, 6330-6335.
27. S. J. Underwood and C. J. Douglas, *Org. Lett.*, 2023, **25**, 146-151.
28. W. I. Nicholson, F. Barreteau, J. A. Leitch, R. Payne, I. Priestley, E. Godineau, C. Battilocchio and D. L. Browne, *Angew. Chem. Int. Ed.*, 2021, **60**, 21868-21874.
29. CrysAllisPro 1.171.42.60a, Rigaku, 2022.

30. G. Sheldrick, *Acta. Cryst. A*, 2015, **71**, 3-8.
31. O. V. Dolomanov, L. J. Bourhis, R. J. Gildea, J. A. K. Howard and H. Puschmann, *J. Appl. Crystallogr.*, 2009, **42**, 339-341.
32. G. Sheldrick, *Acta. Cryst. C*, 2015, **71**, 3-8.
33. C. B. Hubschle, G. M. Sheldrick and B. Dittrich, *J. Appl. Crystallogr.*, 2011, **44**, 1281-1284.
34. R. Procter, C. Alamillo-Ferrer, U. Shabbir, P. Britton, D.-K. Bučar, A. S. Dumon, H. S. Rzepa, J. Bures, A. Whiting, and T. D. Sheppard, Imperial College Research Data Repository, 2024, DOI: 10.14469/hpc/12218
35. E. R. Johnson, S. Keinan, P. Mori-Sánchez, J. Contreras-García, A. J. Cohen and W. Yang, *J. Am. Chem. Soc.*, 2010, **132**, 6498-6506.
36. For an interactive version of these 3D models, see R. Procter, C. Alamillo-Ferrer, U. Shabbir, P. Britton, D.-K. Bučar, A. S. Dumon, H. S. Rzepa, J. Bures, A. Whiting, and T. D. Sheppard, Imperial College Research Data Repository, 2024, DOI:10.14469/hpc/13825
